# Citizen engagement in public services in low‐ and middle‐income countries: A mixed‐methods systematic review of participation, inclusion, transparency and accountability (PITA) initiatives

**DOI:** 10.1002/cl2.1025

**Published:** 2019-08-02

**Authors:** Hugh Waddington, Ada Sonnenfeld, Juliette Finetti, Marie Gaarder, Denny John, Jennifer Stevenson

**Affiliations:** ^1^ International Initiative for Impact Evaluation; ^2^ Education Endowment Foundation; ^3^ Campbell Collaboration

## Abstract

**Background:**

How do governance interventions that engage citizens in public service delivery planning, management and oversight impact the quality of and access to services and citizens’ quality of life? This systematic review examined high quality evidence from 35 citizen engagement programmes in low‐ and middle‐income countries that promote the engagement of citizens in service delivery through four routes: *participation* (participatory priority setting); *inclusion* of marginalised groups; *transparency* (information on rights and public service performance), and/or citizen efforts to ensure public service *accountability* (citizen feedback and monitoring); collectively, PITA mechanisms. We collected quantitative and qualitative data from the included studies and used statistical meta‐analysis and realist‐informed framework synthesis to analyse the findings.

**Results:**

The findings suggest that interventions promoting citizen engagement by improving direct engagement between service users and service providers, are often effective in stimulating active citizen engagement in service delivery and realising improvements in access to services and quality of service provision, particularly for services that involve direct interaction between citizens and providers. However, in the absence of complementary interventions to address bottlenecks around service provider supply chains and service use, citizen engagement interventions alone may not improve key wellbeing outcomes for target communities or state‐society relations. In addition, interventions promoting citizen engagement by increasing citizen pressures on politicians to hold providers to account, are not usually able to influence service delivery.

**Conclusions:**

The citizen engagement interventions studied were more likely to be successful: (1) where the programme targeted a service that citizens access directly from front‐line staff, such as healthcare, as opposed to services accessed independently of service provider staff, such as roads; (2) where implementers were able to generate active support and buy‐in for the intervention from both citizens and front‐line public service staff and officials; and (3) where the implementation approach drew on and/or stimulated local capacity for collective action. From a research perspective, the review found few studies that investigated the impact of these interventions on women or other vulnerable groups within communities, and that rigorous impact evaluations often lack adequately transparent reporting, particularly of information on what interventions actually did and how conditions compared to those in comparison communities.

## 1 PLAIN LANGUAGE SUMMARY

### 1.1 Citizen engagement improves access to public services in low‐ and middle‐income countries, but evidence on development outcomes is limited

Interventions promoting citizen engagement in public service management involve participation, inclusion, transparency and accountability (PITA) mechanisms. In low‐ and middle‐income countries (LMICs), these interventions are effective in improving active citizenship and service delivery, and may improve the responsiveness of service provider staff for services provided directly by public servants (for example, in health).

In contrast, interventions providing information to stimulate pressure on politicians are not usually effective in improving provider response or service delivery. There is insufficient evidence to conclude whether these interventions are effective in improving wellbeing or the relationship between citizens and the state.

### 1.2 What is this review about?

Failures in governance lead to the exclusion of large portions of society from public services and to waste, fraud and corruption. This review assesses evidence for interventions promoting better governance of public services: participation (participatory planning), inclusion (involvement of marginalised groups), transparency (information about citizen rights or performance of public officials), and accountability (citizen feedback) mechanisms, known collectively as PITA mechanisms.

### 1.3 What is the aim of this review?

This Campbell systematic review examines the effects of interventions to promote citizen engagement in public service management. The review synthesises evidence from 35 impact evaluations and 36 related studies of interventions promoting participation, inclusion, transparency and accountability (PITA) mechanisms.

### 1.4 What studies are included?

The review includes impact evaluations relating to 35 PITA programmes from 20 LMICs. In addition, 36 qualitative and programmatic documents were included to strengthen understanding of implementation context and programme mechanisms.

### 1.5 What are the main findings of this review?

Citizen engagement interventions (i) are usually effective in improving intermediate user engagement outcomes, for example, meeting attendance and contributions to community funds; (ii) improve access to and quality of services but not service use outcomes; (iii) can lead to improvements in some wellbeing outcomes such as health and productive outcomes; (iv) may improve tax collection; but (v) do not usually lead to changes in provider action outcomes such as public spending, staff motivation and corruption. There may be an exception where there is direct interaction between citizens and service providers in the regular delivery of services. Interventions providing performance information do not generally improve access or lead to improvements in service quality.

Only interventions focused on services delivered by front‐line staff (e.g., in health) achieve positive outcomes. Those delivered without public interaction (e.g., roads) do not. However, engagement with civil society organisations and interest groups may lead to better outcomes for services accessed independently of providers. Inclusive citizen engagement programmes have at least as big an effect on user engagement and access to services as less inclusive approaches.

Many interventions experienced challenges stemming from a lack of positive engagement with supply‐side actors, whose power the interventions often sought to diminish. Interventions implemented with the strong support of the targeted service providers were better able to realise positive impacts.

Approaches to citizen‐service provider engagement appear to work more effectively when implemented through phased, facilitated collaborative processes rather than one‐off accountability meetings that are seen as confrontational.

Only four studies present any data on intervention costs. This limited the potential for any analysis of comparisons across programmes and settings.

In interpreting the findings, it must be noted that each individual outcome is reported in only a few studies and that included studies have important methodological weaknesses with risks of bias arising from weak design, analysis and reporting.

### 1.6 What do the findings of this review mean?

#### 1.6.1 For policy and programme managers

A collaborative rather than confrontational approach with the service providers whose services are under scrutiny is more likely to be effective. Engaging communities may require using civil society organisations to facilitate the community's participation. Programme design should ensure positive engagement with supply‐side actors within the intervention setting.

#### 1.6.2 For researchers

More high‐quality studies are needed, comparing different approaches to improving service delivery, paying attention to complete description of the different approaches being compared. Since implementation is a crucial factor, mixed methods studies should be the norm, and will help focus on equity considerations which have been neglected. Finally, there should be standardisation of indicators in PITA studies.

#### 1.7 How up‐to‐date is this review?

The review authors searched for studies up to March 2018. This Campbell systematic review was published in June 2019.

## 2 EXECUTIVE SUMMARY

### 2.1 Background

Public services – health care, social protection, justice and physical infrastructure – are critical to enabling the large‐scale development of populations, and thus the focus of significant development aid. Sustainable Development Goal 16 recognises the centrality of effective, accountable and transparent institutions that engage in inclusive and participatory decision‐making to ensure the sustainability of global development investments.

The repercussions of failures of governance – the processes and manners in which people implement policies and programs – are well documented: exclusions of large portions of society from public services is correlated with violent conflict, while fraud and corruption lead not only to wasted investments but to negative impacts on people's quality of life. Interventions aiming to strengthen governance operate across three domains: between the state and citizens; between the state and service providers; and between citizens and service providers.

In this review, we examined interventions that strengthened governance through the “short route” between citizens and service providers, and interventions that improved governance by shortening the “long route” by providing information about the performance of elected officials to improve service provision. Following an Evidence Gap Map study on interventions to promote State‐Society Relations (Phillips et al., 2017), the Centre of Excellence on Democracy, Human Rights and Governance (DRG) at USAID commissioned this systematic review to answer the question, “to what extent are programmes that incorporate PITA characteristics into their design effective, as compared to otherwise similar programmes that do not?”

#### 2.2 Objectives

This systematic review includes projects and programmes that aim to change the ways citizens engage in the planning, running and oversight of public services, and investigates the subsequent impact of these efforts on the quality of and access to public services, and ultimately on people's quality of life and satisfaction with the State. The review applied an innovative approach that sought to understand the mechanisms and processes through which change happens, and to systematically identify the key factors that influence whether an intervention may be effective in a given context.

The review aimed to answer the following five questions:

1. What are the effects of interventions that aim to strengthen PITA mechanisms on *social and economic wellbeing* of participants (intermediate and final outcomes)?
2. What are the effects of interventions that aim to strengthen PITA mechanisms on *participatory, inclusive, transparent or accountable processes* (immediate outcomes)?
3. To what extent *do effects vary* by population group and location?
4. What *factors* relating to programme design, implementation, context, and mechanism are associated with better or worse outcomes along the causal chain?
5. What evidence is available on *programme costs and incremental cost effectiveness* in included studies of effects?

### 2.3 Search methods

The systematic review was carried out according to a protocol that was peer reviewed and published in the Campbell Collaboration library. To identify all potential relevant published and unpublished evaluations to include in the review, the authors carried out a systematic search of key academic databases, donor and practitioner websites, including potential results in all languages, and from any low‐ or middle‐income country, drawing also on an evidence gap map on state‐society relations (Phillips et al., 2017). The searches were carried out between February and April 2018.

### 2.4 Selection criteria

To identify the direct contribution of interventions promoting citizen engagement on service delivery improvements, the review included evaluations in low‐ and middle‐income countries (L&MICs) that compared the impact on service delivery access and quality in participating communities against similar communities where citizens received “standard public services,” which did not have access to the same opportunities or support for citizen engagement in the planning or oversight of those services. The review included quantitative causal studies (randomised and non‐randomised impact evaluations) and also drew on information about mechanisms from the programmes contained in the included impact evaluations (e.g. programme documents, qualitative studies).

### 2.5 Data collection and analysis

Authors conducted a detailed critical appraisal (risk of bias) and external validity assessment of the included studies, to assess the credibility of the findings. To answer Review Questions 1‐3, the authors extracted effect size data measuring the change in outcomes in consistent units from each included impact evaluation. We used statistical meta‐analysis to synthesise the findings. To structure the meta‐analysis, authors used a conceptual model for outcomes along the results chain relating to immediate outcomes (citizen and service provider engagement), intermediate outcomes (access to services and service uptake), and final outcomes (citizen welfare and state‐society relations).

To answer Review Question 4, a framework synthesis of all included studies plus supplemental qualitative and programmatic documents was conducted, to systematically identify the key barriers, facilitators and moderating factors that could explain why an intervention was more likely to achieve its expected results in a given context. Authors identified five intervention groups for the analysis; interventions promoting rights information, performance information, participatory planning, community feedback mechanisms and community‐based natural resource management. Finally, evidence on costs from the included impact evaluations and supplemental documentation was collected to answer Review Question 5.

### 2.6 Results

The search returned over 10,000 papers, from which 50 impact evaluation reports corresponding to 35 programmes that met the criteria for being included in the review, alongside an additional 11 on‐going studies, were identified. For the 35 programmes identified, authors undertook a targeted search and identified 36 qualitative and programmatic documents that were used to strengthen understanding of the context and implementation of the programmes. Authors identified five specific intervention types across the 35 programmes.

Sixteen citizen engagement programs evaluated citizen *participation* in the design and implementation of public services, grouped into two intervention sub‐groups:

- nine participatory priority setting, planning or budgeting interventions, wherein citizens participated in setting the priorities for and/or planning of local services. These include support for participatory budgeting in municipal governments in Brazil, Mexico and Russia, and support for participatory planning in India, Pakistan, Guinea and Kenya. It also included requirements for inclusive participation in two fragile contexts, Afghanistan and DRC.
- seven community‐based natural resource management (CBNRM) interventions, wherein citizens form local collectives and take over the management of a shared resource, for forest management in Nepal, Madagascar and Tanzania, and water user associations in Brazil, China and the Philippines, and Namibia.

Eleven citizen engagement programs evaluated *transparency* mechanisms, which specifically aimed to disclose and/or disseminate information that would shift the power balance between service providers and users, comprising two intervention sub‐groups:

- five evaluations of rights information interventions, which enable users to demand minimum standards for access to services, such as for social protection services in Indonesia (food subsidies) and India (public works), maternal and child health care in India and freedom of information in Pakistan.
- six evaluations of public official or service provider performance information interventions, such as the dissemination of municipal government performance scorecards in Afghanistan, Brazil, the Philippines and Uganda, and monitoring information provided in police stations in India.

Ten evaluations of *accountability* mechanisms were included, which specifically comprised citizen feedback or monitoring mechanism interventions, i.e. those that solicited feedback regarding and/or actively engaged citizens in the monitoring of service delivery, to hold public service providers and institutions responsible for executing their powers and mandates according to appropriate standards. These included community report cards in infrastructure (Afghanistan, Indonesia and Colombia), health (Ghana, Malawi and Uganda), agriculture (Uganda) and the security sector (DRC), and individual citizen “feedback loops” in Guinea, Kenya and Uganda.

Finally, nine of these citizen engagement programs also addressed *inclusion* of marginalised groups. Studies in Afghanistan and DRC focused exclusively on the mandated incorporation of women into community groups. Other programs targeted inclusion of women or poorer groups in Brazil, Indonesia, Malawi, Mexico, Pakistan and Uganda.

Risk of bias assessment was done of all included impact evaluations at the outcome level. Around 35 per cent of the findings for the 19 randomised evaluations were found to be of low risk of bias, 20 per cent had some concerns, and 35 per cent were of high risk of bias. Of the 16 non‐randomised evaluations, almost 90 per cent of outcomes had high risk of bias, 11 per cent had some concerns and only 1 outcome was assessed as being of low risk.

### 2.6.1 Review question 1

Authors found, on average, that citizen engagement interventions **improved** **access to and quality of services** by an overall average pooled effect size of 0.10 standard deviations (95% confidence interval=0.04, 0.16), compared to standard service delivery. Outcomes tended to be of similar magnitude when service access was measured in physical terms or quality of service. Only outcomes relating to reducing staff absenteeism and embezzlement were not systematically different for citizen engagement interventions, as compared to standard public service delivery. When disaggregating by intervention sub‐groups, the pooled effects for rights information and citizen feedback mechanisms were of similar magnitude. However, interventions providing performance information about public officials or service providers did not tend to lead to changes in access to services or improvements in service quality.

Turning to the rest of the causal chain, the results indicated that citizen engagement interventions incorporating PITA mechanisms did not systematically improve **service use**, whether measured as use of health services (e.g. immunisation, antenatal care), social protection services (employment services), or attitudes to services (user satisfaction and complaints). These findings were consistent for intervention sub‐groups (participatory priority setting, CBNRM, performance information, rights information, and citizen feedback mechanisms).

The findings also indicated that citizen engagement interventions can lead to improvements in some **wellbeing outcomes**, where wellbeing is measured using health outcomes (morbidity, mortality, nutrition) or productive outcomes (agriculture yields, income/expenditure, asset ownership). However, these overall changes tended to be small in magnitude (around 0.10 standard deviations increase in the outcome) and were not observable consistently across all outcomes analysed. The outcomes measured were diverse, and sample sizes for each outcome small, hence it is not possible to draw strong conclusions. In addition, interventions providing performance information about public officials or service provision did not increase wellbeing outcomes.

Outcome measures of **state‐society relations** included public confidence in institutions, institutional sustainability and taxes paid. Some study results suggested citizen engagement interventions may improve tax collection. There were no improvements for the other state‐society relations outcomes (corruption perceptions or confidence in institutions), although only two studies were identified for each. It is therefore not possible to draw strong conclusions.

### 2.6.2 Review question 2

In order to examine effects on immediate outcomes, service user and service provider engagement were analysed separately. The main finding from the analysis of **user engagement outcomes** is that interventions incorporating PITA mechanisms are usually effective in engaging service users, for example by improving meeting attendance, contributions to community funds and general knowledge about services. The average pooled effect on user engagement was an increase of 0.23 standard deviations across all outcome measure. When the findings were disaggregated by citizen engagement intervention, the results indicated that pooled effect magnitudes were similar and statistically significant for some intervention sub‐groups (participatory priority setting, CBNRM, rights information, citizen feedback mechanisms). Hence, in general, this review found that interventions usually lead to improved citizen engagement outcomes, as compared to standard public service delivery.

However, the effects of interventions promoting citizen engagement on **provider action outcomes** are very limited. Overall, provider responsiveness to the intervention tended to be small, by a small pooled effect which was statistically insignificant. No significant pooled effects were found for specific outcome measures such as public spending, staff motivation, corruption or responses as perceived by service users. Nor were there significant pooled effects for intervention sub‐groups (participatory priority setting, CBNRM, performance information, rights information, or citizen feedback mechanisms). In sum, this review found that citizen engagement in public services interventions do not usually trigger service provider actions.

### 2.6.3 Review question 3

Diversity and equity of impacts differ across population groups in three ways. Overall, few of the studies reported disaggregated intervention approaches and/or analysis of results for different population groups. Nine programmes incorporated specific measures within the intervention to extend the engagement to vulnerable groups. These **inclusive citizen engagement** programmes tended to have as big or bigger effects on user engagement and access to services as other citizen engagement programmes. Across the whole pool of included studies, 12 conducted sub‐group analysis to differentiate impacts for different population groups, most commonly by socio‐economic status and by sex of participant, yet these were spread widely across intervention type and geography. This review identified only one mixed‐methods study that conducted equity‐oriented causal chain analysis to differentiate impacts for women. Analysis by global region was not able to find consistent differences by intervention or outcomes along the results chain. Ultimately, due to the small sample of studies across a wide range of interventions and outcomes, it was difficult to conclude anything systematically for different population or geographic groups.

### 2.6.4 Review question 4

Through the realist‐informed framework synthesis, this review assessed the key contextual and implementation factors, along with the barriers, facilitators and moderating factors that were influential in shaping the results chains for each of the five intervention sub‐groups. The initial results chain framework was further developed for each intervention sub‐group, moving towards “best‐fit” framework synthesis, to better reflect the different factors and steps in the intervention causal chains.

Amongst *participatory planning* interventions, three facilitating factors were identified that may improve the likelihood of achieving results along the causal chain. First, strong local buy‐in for the participatory process, particularly where communities self‐selected into the intervention may encourage more active and effective engagement. Second, the incorporation of specific, culturally appropriate measures that address local barriers to the participation of vulnerable groups may be key to ensuring that decisions taken reflect pro‐poor values and outcomes. Finally, participatory planning processes that engaged and/or stimulated the growth of local civil society and capacity for collective action may be more sustainable and more likely to achieve long‐term results.

Four contextual factors were identified that mediated results chains amongst *community‐based natural resource management (CBNRM)* interventions. Where interventions required large shifts in control over the resource, representing a relinquishment of power from local officials to community groups, lack of engagement and buy‐in from local officials was a frequent barrier to the full implementation of the CBNRM policy. Critically, this barrier often resulted in situations in which community groups took on additional responsibilities for resource management, but did not gain access to the corresponding promised benefits. A related factor is the clarity of the national CBNRM policy context; where there were multiple vague and overlapping policies governing natural resource use, officials were more able to adjust or block full implementation of CBNRM in a way that preserved their power and control over resource benefit access. External support to change resource use was a key facilitating factor: even in the absence of full policy implementation, access to alternative livelihoods such as tourism may still enable communities to realise the joint socio‐economic and environmental objectives of CBNRM. Finally, the type and intensity of local resource use were key moderating factors influencing the effectiveness of CBNRM; community management may not be appropriate in contexts prone to illegal logging or poaching, where attempts to enforce regulations may endanger community members.

Across *citizen feedback and monitoring mechanism* interventions, four key facilitating factors were identified. First, there was a strong distinction between projects targeting services delivered directly to citizens by front‐line providers, such as healthcare, versus those that citizens access independently of staff who implement and manage the services, such as infrastructure. The social sanction threat of individual citizens’ voices was not strong enough to spur improvements amongst providers who do not interact with users on a regular basis. Building on the criticality of sustained, direct engagement, the findings suggest that interventions that took a phased, facilitated approach to engaging citizens and service providers jointly in the monitoring of service delivery may be able to trigger intrinsic motivation within service providers and create a sense of working towards a common goal, which may be more effective compared to more confrontational town‐hall‐style meetings. The incorporation of performance benchmarks was also a frequent facilitating factor to enable community monitors to identify realistic opportunities for local improvements in service delivery. Finally, ensuring the creation of common knowledge around feedback or monitoring results, and working through local community organisations were identified as further facilitating factors that strengthened the weight of citizens’ voices and their power to hold service providers to account.

*Rights information* interventions were more likely to be successful where they targeted the provision of services that citizens access through interactions with service provider staff; created a sense of common knowledge about people's rights to the service amongst both citizens and providers; and created an appropriate level of social sanction risk for providers. An initial critical factor is whether citizens’ lack of knowledge of their rights was the key barrier preventing them from accessing services, as opposed to an issue on the “supply” side of service delivery, such as a lack of capacity amongst service providers to deliver the service. Because rights information interventions rarely engage with service providers, even where service use may change as citizens effectively bargain for access, improvements in service delivery quality are unlikely.

Finally, amongst *performance information* interventions, a key facilitating factor was the extent to which implementers secured the support of and buy‐in from the individuals whose performance was being analysed and disseminated. Without such support, the findings suggest that the targeted individuals may be able to avoid accountability by either preventing full implementation of the intervention, or by successfully undermining the credibility of the performance information disseminated. Most of these interventions targeted political actors’ performance (as opposed to sector‐specific public services), in attempts to “shorten the long route” of citizen‐state accountability by increasing citizen engagement with politicians outside of elections. While interventions were at times successful in eliciting some improvements in politician performance, the findings suggest that, ultimately, this route remains too long to identify short‐term effects on service delivery. Politicians may claim plausible deniability of their individual capacity to influence service delivery change, and such interventions do not engage many key actors involved along the public service delivery supply chain.

A key factor influencing progression along the causal chains for accountability and transparency‐for‐accountability interventions in the framework synthesis was found to be whether interventions targeted public services that were delivered to citizens directly by front‐line providers, typically merit good services such as healthcare, versus those that targeted purely public good services delivered indirectly to citizens, such as roads. Disaggregating the meta‐analysis amongst accountability interventions targeting merit versus pure public good services suggested that citizen engagement improved across all services. However, interventions targeting directly delivered merit‐good services were better able to elicit positive responses amongst service providers, a difference which appeared to trigger a break in the causal chain for interventions targeting indirectly‐delivered, pure public good services. The findings showed positive effects for outcomes of service quality and access amongst directly delivered services, but insignificant findings on outcomes amongst interventions targeting indirectly delivered pure public goods.

### 2.6.5 Review question 5

Cost effectiveness is a key question for decision making, yet it is rarely incorporated into impact evaluations. Only four studies presented any data on intervention costs, usually at highly aggregated level (e.g. total cost of intervention) and only the study of report cards in health in Uganda presented cost effectiveness information (cost per under‐5 death averted). This limited the potential for any analysis of comparisons across programmes and settings.

### 2.7 Authors’ conclusions

#### 2.7.1 Implications for policy makers and practitioners

The findings suggest interventions to improve governance through citizen engagement in public services may be effective in stimulating active citizen involvement and improving access to and the quality of public services. Sustained, direct engagement between citizens and service provider staff appears to be key: the biggest effects were seen for interventions that targeted public service governance through the “short route” of direct engagement between citizens and service providers, and which targeted services that citizens access directly from front‐line providers, typically merit goods such as healthcare.

However, citizen engagement interventions alone do not typically improve use of services and may not also lead to better wellbeing outcomes for citizens or state‐society relations. The authors hypothesise that this may be due to the absence of complementary interventions to address bottlenecks over which citizens can have limited access, such as service provider budgets and supply chains or technical capacities.

Citizen engagement interventions were less successful where there was less engagement between citizens and front‐line providers. This occurred where interventions aimed to shorten the “long route” of improving governance by increasing citizen pressures on politicians to improve public services through politician performance information, or where interventions targeted services such as infrastructure, which citizens access independently of service providers.

Interventions that work through local civil society groups and stimulate capacity for collective action, particularly amongst vulnerable groups, may be more effective than those that rely on engaging unorganised citizens. This is particularly critical for public good services such as infrastructure, wherein citizens must overcome the collective action issue.

Interventions that obtain and sustain buy‐in from local public service providers at the point of citizen engagement may be more effective at creating appropriate threats of social sanctions and stimulating intrinsic motivation to stimulate behaviour changes amongst service providers to improve service delivery quality. This is particularly critical in CBNRM, to ensure interventions do not do unintentional harm by increasing the burden on communities for resource management without enabling them to realise full access to the benefits in return.

Interventions that do not incorporate specific measures to facilitate the inclusion of vulnerable groups may not realise equitable outcomes for those groups in the short‐term. Barriers to vulnerable groups’ inclusion varies widely by context, and inclusion components should be adapted in response to local contexts and needs.

### 2.7.2 Implications for research

Impact evaluations need to “open intervention black boxes” by being more transparent in reporting of intervention design and implementation fidelity, as standard. This also includes clearer reporting of the comparison conditions received by groups outside of the intervention. Authors may draw on frameworks for intervention reporting guidelines, such as TIDieR in health sector research. Impact studies also need to engage more consistently with equity issues, either by evaluating intervention components specifically targeting equity, collecting outcomes relevant for certain vulnerable groups, or at the very least reporting outcomes subgroups for vulnerable groups. Most studies collected outcomes data shortly after intervention, usually within 5 years. There may be opportunities to examine outcomes over longer periods cost‐effectively, for example by conducting more follow‐up studies of existing trials, or by conducting ex post evaluations using natural experiments.

In this review, the authors used theory‐based mixed‐methods approaches to examine a wide range of interventions promoting citizen engagement in public services governance, taking the PITA mechanism as the unit of analysis. Further synthesis research adopting this broader approach may focus on interventions to improve other domains of governance (e.g. the compact between state and service provider), combinations of domains (e.g. citizen engagement plus compact), or by comparing citizen engagement with other approaches to increase state capacity such as through better monitoring of public service delivery agents. The authors also note that systematic reviews usually focus on the effectiveness of particular interventions, and new systematic reviews of specific interventions (e.g. participatory budgeting, water user associations) and updates of existing reviews (e.g. community monitoring, education sector govnernance) are needed.

## 3 BACKGROUND

### 3.1 The problem: unaccountable government systems and poor service delivery

The sustainability of global development investments depends on strong institutions, citizen engagement, accountable governments, and equitable economic growth (World Bank, 2017). Goal number 16 of the Sustainable Development Goals explicitly recognises the importance of the development of effective, accountable and transparent institutions at all levels, and of ensuring responsive, inclusive, participatory and representative decision‐making at all levels (UNDP, 2016). In the Paris Declaration on Aid Effectiveness, donor and partner countries committed to improving their mutual accountability and transparency in the use of development resources, with partner countries further committing to systematically involve diverse stakeholders in national development priority setting processes (OECD/DAC, 2005). Many development challenges, such as poor service delivery, corruption and slow growth, persist because of the political context around them; they are as much about power dynamics as they are technical challenges.

Improving the governance of public institutions and service delivery has long been a central tenet of strategies for achieving or supporting development; World Bank *World Development Reports* since the late 1990s have included elements of improving governance as central to their theories of change (Grindle, 2004). In the decades since, mainstream approaches to realising good governance have shifted in focus, away from privatisation of service delivery and towards a focus on increasing the engagement of constituents, particularly vulnerable groups, with public institutions and service providers in such ways to increase the effectiveness, appropriateness, and quality of service delivery. The 2004 *World Development Report* (WDR) highlighted the insight that public spending on service delivery in developing countries often primarily reached the better‐off minority of citizens; for example, in India, curative health subsidies were primarily going to the richest 20 per cent of the population, who received three times the subsidies of the poorest 20 per cent (World Bank, 2004). This insight remains pertinent. For example, a recent evaluation of an e‐governance intervention in India that aimed to improve transparency in a fiscal transfer system for a social benefits programme suggested that while the intervention was successful at reducing leakages, the savings did not translate into improved outcomes for beneficiaries (Banerjee, Duflo, Imbert, Mathew, & Pande, 2017). One of the authors later posited that this may have been because the intervention did not empower the ultimate beneficiaries to ensure that financial gains from reduced corruption were converted into increased outcomes for poor people (Page and Pande, 2018).

There are many definitions of governance. For the purposes of this review, we use the recent definition employed by the World Bank, where governance is defined as “the process through which state and non‐state actors interact to design and implement policies within a given set of formal and informal rules that shape and are shaped by power” (World Bank, 2017). Where characteristics of good governance are weak or absent from public processes and service delivery, the effectiveness and sustainability of development interventions is likely to suffer (World Bank, 2016). Barriers to access to public services for vulnerable groups exacerbate inequality, with potential long‐term repercussions for a society's development (Easterly, 2007). Fraud and corruption are pervasive across low‐ and middle‐income countries, and the negative consequences on quality of life and core development outcomes are well documented (Molina, Carella, Pacheco, Cruces, & Gasparini, 2016; Svensson, 2005). Where state and public actors cannot be effectively held accountable, a culture of impunity develops that normalises fraud and rent‐seeking practices. The World Bank's *World Development Report 2017* highlighted key repercussions of power asymmetries, including: exclusion of large portions of society from services, institutions or resources, which is correlated with violent conflict: elite and/or interest‐group capture of policies in order to serve interests, resulting in poor targeting and ineffective or inappropriate policies, which can lead to poor or stagnant growth, condemning economies to an under‐developed state; and clientelism, which often leads to rent‐seeking and poor service delivery, which have long‐term repercussions on societies’ growth (World Bank, 2017).

Despite the decades of acknowledgement of the importance of good governance, progress has been slow; the Worldwide Governance Indicators show limited to none or even negative progress on key governance indicators amongst aggregates of low and lower‐middle income countries from 2006 to 2016 (World Bank, 2018). The repercussions of continued governance failures are high, and well documented; for example, in Nigeria, unabated corruption led to the squandering of billions of dollars by the National Petroleum Company, jeopardizing the country's long‐term growth potential and financial stability (World Bank, 2017).

Approaches to improve governance have generally either focused on mechanisms to strengthen the effectiveness and institutionalisation of public institutions, or on external pressures to improve service delivery despite weak institutions. While each approach has yielded valuable insights, translating insights from theory into practice has been challenging. There is some evidence that at times, failures could be due to an over‐emphasis of the demand side of governance by service users, citizens and civil society, which ignores the constraints faced on the supply side by politicians, bureaucrats and service providers (Brinkerhoff & Wetterberg, 2015), or of the power of information (Wibbels & Keohane, 2018). More recently, insights are emerging into the value of system‐based approaches that look at both the supply and demand sides of governance as actors in a single system, drawing on power analyses and social network theories (Fox, 2014; Halloran, 2015; Mcloughlin & Batley, 2012; Wibbels & Keohane, 2018).

USAID's Democracy, Human Rights and Governance (DRC) Center identified *participation, accountability, transparency and inclusion* (PITA) as critical principles that could be incorporated into interventions within and across sectors to improve development outcomes, and in line with the Doing Development Differently global initiative (USAID, 2016). We define participation as efforts to involve citizens in the design, monitoring and delivery of policy and programmes upstream (Quick & Feldman, 2011). Transparency is a “characteristic of governments, companies, organisations and individuals of being open in the clear disclosure of information rules, plans, processes and actions” (Transparency International, 2009: 44). Accountability is the concept that individuals, agencies and organisations are held responsible for executing their powers according to a certain standard downstream (McGee & Gaventa, 2011). Finally, inclusion means a particular focus on marginalised and vulnerable citizens in policy and programming upstream or downstream (Quick and Feldman, ibid).

### 3.2 Interventions to strengthen good governance

A recent evidence gap map (EGM) on interventions to improve “state‐society relations” highlighted a number of interventions to improve governance (Phillips et al., 2017). These were broadly grouped into interventions for inclusive political processes and leadership (e.g. community‐driven development, electoral monitoring, and quotas for women and minority representation in political institutions), and interventions for responsive and accountable institutions and service delivery (e.g. audits, land reform and public servant performance incentives).

Drawing on Phillips et al. (2017), and also insights from the literature, we theorised good governance can come about through sustained improvements across three domains: within the political system; within the management and administration of public sector offices and institutions; and in the ways in which public officials and service providers engage with service users (external engagement) (Waddington, Stevenson, Sonnenfeld, & Gaarder, 2018). In this framing, good governance interventions attempt to influence the social contract that mediates the relationships between government and citizens, regarding who has access to what power and in return for what accountability for service provision, through three accountability domains:

- *Influencing how the broader political system functions:* The broader political system dictates access to and contestability of the policy arena (World Bank, 2017). This primarily comprises the checks and balances, or “horizontal accountability” between institutions, yet also includes political representation systems and thus, as an extension, elements of “vertical accountability” that are exercised through electoral systems (Transparency and Accountability Initiative TAI, 2017). Increasingly, good governance interventions seek to influence *how* this system functions, rather than the specific form it takes (World Bank, 2017).
- *Influencing how a specific public service or institution's system functions internally:* Many good governance interventions aim to improve service delivery through the institutionalisation of public services and institutions. These interventions foster “internal accountability” of institutions, and include, but are not limited to, strengthening human resources management, systems of upwards accountability of staff and management or between different levels of government, and supply chains for infrastructure, goods, and financial flows (Finan, Olken, & Pande, 2015).
- *Influencing how a specific public service or institution engages externally with constituents:* These interventions aim to mediate the ways that citizens engage with government and public service providers outside of the “long route” of electoral processes (World Bank, 2004). They work to improve service delivery through “external accountability”, by increasing the engagement between service providers and service users to improve the responsiveness and effectiveness of public services. This comprises the informal processes of vertical accountability, through which citizens, CSOs and the media may attempt to influence political and public service actors directly, as well as efforts towards “diagonal accountability,” formalised processes in which citizens are engaged in horizontal accountability efforts (Transparency and Accountability Initiative TAI, 2017). In addition, it may include approaches which aim to “shorten the long route” by providing information on performance of public servants.

**Figure 1:**
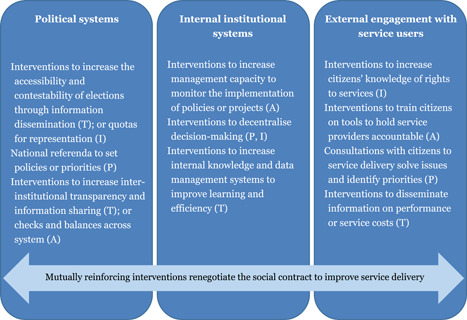
PITA throughout the three domains of good governance Notes: P: Participation | I: Inclusion | T: Transparency | A: Accountability. *Source*: Authors

Many good governance interventions are designed to improve service delivery for citizens. This is often done through interventions that embody one or multiple PITA characteristics, which seek to address power dynamics between the state, civil society and citizens to make service delivery more effective and equitable (USAID, 2016). PITA characteristics influence the functioning of the social contract and its systems throughout each of the three accountability domains, and thus, good governance interventions may target one or more of these (Figure 1). For example, within the political system domain, the PITA characteristics have a direct impact on who has access to the electoral systems and who can contest the policy arena. Elected officials must exercise some basic level of downwards accountability towards the constituents who elected them (or, in non‐democratic states, who grant them legitimacy), and sideways accountability to their fellow statesmen through the checks and balances built into the system. Interventions targeting PITA mechanisms in this domain tend to focus on *creating a fair system*. Within the internal system domain, interventions tend to focus on *creating an efficient system*, such as through improving the upwards accountability of officials and service providers to management, or through improving the relevance of service provision at local levels through decentralisation. Finally, in the external engagement domain, the PITA characteristics of a service or institution mediate the means through which it engages with citizens, civil society, and business/interest groups. These interventions aim to address a more diverse set of system attributes, primarily the *relevance, effectiveness and inclusivity of the service delivery system*, and are further differentiated from those in the previous domains through their reliance on soft power. The following figure (Figure 1) provides some examples of interventions which target the different domains of good governance.

The effectiveness of interventions that target the PITA characteristics within one domain will be mediated by the context of the other domains as well, the power relations and constraints, and also by other interventions aiming to improve good governance and service delivery, particularly those that target service delivery supply chains. There is increasing scholarship that suggests that while interventions improving the PITA characteristics of public services and institutions, particularly in the external engagement domain, may be *necessary* for achieving sustainable improvements in service delivery and a stable social contract, they may not be *sufficient* (e‐Pact Consortium, 2016). On the other hand, while interventions that target strengthening PITA characteristics within internal institutional systems may be sufficient for improving governance within the system, the impact of those governance improvements may not reach the ultimate beneficiaries (citizens/service users) without the incorporation of interventions strengthening the system's external PITA characteristics (Page & Pande, 2018).

### 3.2.1 The focus of this review on citizen engagement interventions

While recognising the interactions of interventions promoting PITA mechanisms across each domain and with complementary good governance and service delivery initiatives, it has been pointed out that to attempt to cover the entirety of good governance interventions in a single review would be “exceedingly ambitious” (Sáez, 2013). Thus, this review analysed the value‐addition of interventions in the third domain, external PITA interventions targeting public service and institution engagement with citizens.

Interventions promoting PITA mechanisms can be implemented as stand‐alone interventions or as part of a larger programme working to strengthen governance and service delivery. They may be implemented either on the supply or demand side of service delivery, or may target both simultaneously, such as a public audit process that trains community members on tools to hold public officials accountable, and works with public officials to increase their understanding of the importance of downwards accountability. An intervention may strengthen one or multiple PITA characteristics of the ways public services and institutions engage with their constituents.

For the purposes of this review, the definitions of PITA were operationalised as follows:

- *Participation:* The intervention promotes or formalises continuous citizen input in the design and implementation of public services, processes or policies. Participation interventions create specific opportunities or processes for citizens to provide meaningful input into public policy or strategy design and planning. An example of a participation intervention is the introduction of participatory budgeting so that citizens may directly contribute to the development of a budget proposal (Touchton & Wampler, 2014). A community‐level example could be the creation and capacity building of a representative community‐based natural resource management committee that is mandated to develop and monitor locally agreed standards and regulations for the use of common property.
- *Accountability:* The intervention encompasses monitoring and soft/social accountability mechanisms to encourage or actively hold individuals, public service providers and institutions responsible for executing their powers and mandates according to a certain standard. Accountability interventions create opportunities or processes for constituents to monitor the government and public service providers. An example is a project to encourage and build the capacity of civil society to hold government accountable for the sustainable and equitable management of natural resources (USAID, 2016), or a citizen report card intervention, in which a community group is taught the quality standards to which they are entitled and how to monitor the quality and performance of service delivery, and then to work with the service providers to address any identified issues through a mutually agreed action plan.
- *Transparency:* The intervention involves the disclosure and/or dissemination of information about rights of public service users, to promote participation, and/or performance of public service providers, to promote accountability. Transparency interventions included in our review have the explicit aim of changing the way that citizens and service providers or public officials interact and the power relations between service providers and users. An example is local clinics posting information about patient rights, service fees and standards, and budget execution (USAID, 2016), which restricts the scope for service providers to charge bribes.
- *Inclusion:* The intervention includes particular strategies to promote the opportunities and capacities of marginalised and vulnerable groups such as women, ethnic minorities or lesbian, gay bisexual, transgender and intersex (LGBTI) people to engage with the management of public institutions and service providers. Hence, we define inclusion specifically as a component of an intervention that targets a change in participation, transparency or accountability. An example of an intervention to promote inclusion is ensuring that a certain proportion of places in a community governance group are reserved for women (Humphreys, de la Sierra, & van der Windt, 2012).

The intervention categories are described in more detail below (see Table 4 in the Methodology section). While most interventions contribute primarily to a single PITA mechanism as described above, there is often significant interplay between the PITA characteristics to which an intervention contributes. Though efforts have been made to make the definitions mutually exclusive, a single intervention may contribute to strengthening multiple PITA characteristics. The obvious cases here are interventions for transparency and inclusion. For example, a transparency intervention that improves access to information about users’ rights may aim ultimately to improve user participation, while one aiming to improve information about public service performance ultimately aims to improve accountability. Further, interventions included in the review that are designed to improve the access of a marginalised group of citizens (inclusion) to a decision‐making process aim, at an intermediate outcome level, to improve the group's input into the process by providing increased opportunities for consultation (participation), or service delivery monitoring (accountability).

### 3.3 How citizen engagement interventions might work

**Figure 2:**
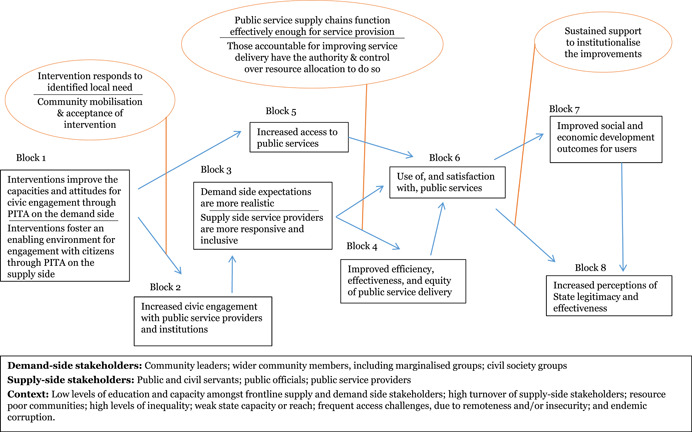
Indicative theory of change

We developed a stylised model showing an indicative theory of change for how the interventions may work at the protocol stage (Figure 2). The theory of change is represented as a series of “blocks,” though the authors recognise that change is not always linear and may be multi‐directional. The numbers represent typical hypothesised progression, and enable signposting to the key stages of the change process in the text. Circles are used to represent underlying assumptions and key factors that facilitate, moderate or create bottlenecks along the casual chain. This preliminary theory of change developed in the systematic review protocol (Waddington et al., 2018) drew on insights from the literature and programmatic best practices. In particular, the framework built on the 2004 *World Development Report* (World Bank, 2004) theory of change, which articulated the importance of pro‐poor governance practices that actively engage end users for effective outcomes, and Rahman and Robinson (2006) who articulated the importance of local ownership and long‐term support. The assumptions and moderating factors drew on insights from Fox (2014), Page and Pande (2018), and the 2017 WDR (World Bank, 2017), among others. We have not taken a “rights‐based approach” that views improvements in PITA characteristics as the end objective. While recognising the value of PITA characteristics in and of themselves, the focus of this review is on the value‐add they bring to improving development outcomes through improved service delivery.

We note here a useful distinction between the demand and supply side of governance. Implementers may target stakeholders on the demand‐side of governance, such as through efforts to improve the capacity of civil society to monitor government service delivery, or the supply‐side, such as by training public officials on pro‐poor development planning. Other interventions may be geared to affecting both demand‐ and supply‐sides, such as a participatory budgeting process in which government officials are trained on the value of participatory budgeting, while community members are trained and supported to participate in the process.

The indicative theory of change presents the hypothesised causal chain for citizen engagement interventions, from changed opportunities for and capacities of citizens, followed by behavioural changes on both the supply and demand side of governance, ultimately leading to improved service delivery performance and enhanced quality of life outcomes for citizens. The indicative causal chain was developed in the systematic review protocol stage to articulate the main causal pathways through which interventions targeting public services’ and institutions’ external engagement with citizens may lead to improved development outcomes. Some interventions may contribute to all the pathways; others may only contribute to particular ones. We build on, and further refine, theories of change for specific intervention sub‐groups in the framework synthesis (Review Question 4).

Beginning with the intervention on the left‐hand side, the figure follows a primary causal chain, with immediate, intermediate and endpoint outcomes indicated in boxes, and key assumptions in bubbles. The theory of change starts with critical assumptions of the design, inception and implementation phases: first, that the intervention designed is relevant and addresses an identified local need; second, during inception that wider community acceptance for the intervention has been sought and received from key social, religious and political leaders; and finally, that community mobilisation activities are undertaken during implementation. Similar to how the quality of PITA characteristics in the public planning and service delivery spheres contributes to strengthening the corresponding development outcomes, the strength and quality of the PITA characteristics of the intervention itself are suggested to contribute to its efficacy.

The exact form of the intervention (Block 1) will vary widely, yet the majority aim to create an enabling environment for increased and mutually empowering interactions between service providers and citizens through changes to their knowledge, attitude and practices (KAPs). On the demand side, this may include efforts to improve citizens’ knowledge of the services to which they are entitled; their capacity to demand those services through key tools; and/or their sense of self‐efficacy and empowerment to do so effectively. An intervention focused on a technical skill such as participatory budgeting may start with capacity building on budgeting processes and the role that citizens can play; interventions that aim to increase inclusion of marginalised groups often start with community campaigns to raise awareness amongst the target households.

On the supply side, either in addition to demand‐side efforts or independently, interventions aim to strengthen openness from and active engagement with supply‐side stakeholders in efforts to improve service delivery. These may target the actors implementing or managing the service in question, but also other key stakeholders in the community and throughout the system. Seeking and attaining community acceptance prior to implementation is a widely‐applied best practice for ensuring that development projects do no harm and that they will have sufficient buy‐in from the community to be successful. There is some evidence that suggests that this may be particularly critical for PITA mechamisms. Securing buy‐in from stakeholders at the point of intervention, upstream and downstream along the service delivery / good governance supply chain may create an enabling environment for interventions to successfully navigate the social network and power differences within which the intervention is implemented (Mcloughlin & Batley, 2012).

Different tools implemented as elements of citizen engagement interventions may require different conditions to be effective; the framework synthesis process aimed to identify these. The context also influences what works and how; for example, if a political player can increase his or her own personal power through framing an improvement in service delivery as a personal “win,” then she or he may be more motivated to work for its improvement (e‐Pact, 2016).

The first immediate outcome (Block 2) posits that through engaging in the interventions, citizens will increase their engagement with State and public service officials. This is often an explicit aim of citizen engagement interventions, as it is a critical precursor to the higher‐level outcomes. Through the increased engagement, the next level of change (Block 3) posits that citizens develop a better understanding of processes, services, and the constraints faced by service providers, while simultaneously, service providers gain a deeper understanding of the needs of their constituents and appreciation for the engagement process.

In subtle ways, these changes reflect renegotiations of power relations between the State, civil society and citizens, mitigating the power imbalances. This happens as the citizen engagement interventions shift the dynamics of power by drawing on collective and representative voice.

- Participation interventions address power relations by building in meaningful opportunities for citizens to provide input over the direction of policies that affect them and the supply of services they rely on.
- Inclusion interventions address power relations by bringing marginalised voices to the table.
- Transparency interventions address power relations by limiting the government and public service providers’ capacities to use their positions for personal gain, and addressing the power difference caused by knowledge gaps.
- Accountability interventions address power relations by increasing the risk and severity of informal social sanctions against poorly performing bureaucrats and service providers.

Power relations are dynamic; they can change quickly, both for the better and worse, and gains are not necessarily secure. A key assumption here is that supply‐side actors are fully engaged throughout the process; otherwise, the attempts to increase soft power by citizens may be seen as confrontational rather than collaborative, which could de‐incentivise service providers from the process to avoid being seen to give up any of their power (World Bank 2004). Where PITA processes are seen as collaborative, they can be mutually empowering, creating changes in the interactions between state and society that simultaneously give citizens greater input into the provision of the services they rely on, and strengthen the standing of the service providers in the community (Fox, 2014).

Where interventions are unsuccessful at building coalitions to facilitate an enabling environment for change, they may not be successful at changing power relations, as actors may adapt to new systems (Halloran, 2015). For example, though advancements in the field of information and communications technology (ICT) offer exciting possibilities for strengthening external PITA characteristics, a change in technology that is not complemented by supporting interventions that create an enabling environment may fall flat (Hogge, 2010).

As the power relations are shifting and engagement is increasing, a core intermediate outcome of the interventions will emerge (Block 4): public service delivery will improve in efficiency, effectiveness, and equity. Once public officials and service providers are taking into account the input of community members, the selection and targeting of services will improve. This will improve the effectiveness and appropriateness of public service delivery. Inclusion interventions improve the equality of service provision, as they increase access to services and processes for the most vulnerable community members. Transparency initiatives increase the efficiency of public service delivery, as they streamline costs and processing times, and make it harder for politicians and officials to demand inflated payments for services. Finally, accountability initiatives can have direct benefits to the performance of public service delivery, as citizen feedback mechanisms such as Public Audits end with joint workshops between the service provider, citizen representatives, and other key stakeholders to come up with an actionable plan to which all parties can be held to account for how they will address the major issues identified and improve service delivery.

The key assumption here is that institutions have the capacity to respond to priorities requested and issues raised by constituents. This is a critical assumption, because in its absence, the interventions risk doing harm by having a negative consequence on perceptions of State effectiveness resulting from raised and then unmet expectations. For example, the 2017 WDR highlights the risk that investments in service provider capacities may not be enough to improve service delivery, if power relations within the institution are not addressed (World Bank, 2017). Further, depending on the structure of the intervention, improvements may be related to a one‐off change in the situation that is not sustained; many citizen engagement interventions are designed as experiments, whose study design may capture short‐term gains that revert back to the baseline conditions with time. Fung et al. suggest that transparency interventions contribute to improvements only when the information provided becomes embedded in the decision‐making process (2005).

In some cases, citizen engagement interventions, particularly those that focus on improving access to services for marginalised groups (inclusion), may not lead to the active, empowered engagement between citizens and service providers that leads to mitigated power differences and improved services. However, they could still lead to increased access to public services, particularly amongst vulnerable populations (Block 5). This comes about as a direct result of citizen engagement interventions relying on inclusion and transparency (information dissemination) mechanisms, but also through the other interventions; as communities are mobilised to engage with their local government and services, they become more invested in the services that they are attempting to improve. And thus, they are more likely to take advantage of those services, as they understand the importance of ensuring high quality service provision for themselves and their families. However, increased access for marginalised groups is not a given outcome of citizen engagement interventions; there likely needs to be concerted, targeted efforts to reach and engage these groups in order for the impacts to reach them (E‐Pact Consortium, 2016). Similarly, interventions targeting services where changes are relatively immediate and visible may be more likely to encourage buy‐in and support from supply‐side actors (ibid.).

The joint effects of changes from Blocks 4 and 5 lead to improved use of public services anduser satisfaction (Block 6). Further along the causal chain, wellbeing outcomes may also improve (Block 7). Wellbeing outcomes will vary by intervention sector (e.g. health, social protection, justice, natural resource management), and are more likely to improve in complementary enabling environments. In the majority of citizen engagement programmes, the PITA characteristics interventions are add‐ons to core interventions and outcomes in a public service sector. In the long run, all three intermediate outcomes contribute to wellbeing outcomes. A key assumption is that sustained support is provided to the institutions or service providers charged with maintaining the implementation of the intervention, such that it becomes institutionalised. As noted above, power differences are dynamic and constantly evolving. Thus, a short‐term project may well change outcomes in the short‐term, but without proper support those gains may easily be lost.

Finally, it is increasingly thought that citizen engagement interventions, through the immediate outcomes increasing engagement with government and public officials and mitigating the power differences, can have a positive impact on perceptions of state effectiveness – or state‐society relations – when services and development outcomes improve as a result (World Bank, 2017) (Block 8). As citizens engage with public processes and services, they learn more about the constraints under which these institutions operate. As they see increased responsiveness of public officials, and subsequent real improvements in the quality of services they receive, their perceptions of State effectiveness and legitimacy will increase. This is particularly critical in fragile and post‐conflict Sates, where the State may still be vying with other actors for legitimacy over governing and control. There may also be reinforcing feedback from improved state‐society relations (block 8) to use of services (block 6).

The context in which this theory of change, or elements of the same, are implemented has strong ramifications for the ways in which the interventions must be designed, implemented and supported in order to ensure success. Governance programmes are generally implemented in resource‐poor contexts, where there are entrenched problems around low levels of education and capacity, high turnover amongst public officials, and endemic corruption. Target communities are frequently difficult to access, either due to remoteness and extreme weather, or to conflict and insecurity. It is precisely because of these challenges that governance interventions are so strongly needed in such areas, but they must be taken into account during the design phase to ensure risks are appropriately mitigated. These factors breed vicious cycles of weak public service supply, which leads to weak demand, which in turn facilitates weaker public financial management, and so on. In an ideal world, the citizen engagement interventions would create a virtuous circle of active community engagement in their government and service provision.

Interventions tailored to the specific context in which they are implemented, that target both the demand and supply sides of good governance, are more likely to be successful, particularly when the interventions are supplemented by complementary ones that target the technical side of service delivery and/or service delivery supply chains. For example, in the Philippines, a project focusing on improving access and quality of maternal and child health and family planning included social accountability mechanisms in the form of Quality Assurance Partnership Committees, which Brinkerhoff and Wetterberg (2015) argue led to more effective service delivery that improved the client‐focus of providers and increased service use.

Additional factors that may influence an intervention's results include top‐down political will, which is key to ensuring that local government officials and service providers have the capacity to implement the changes they agree to with their constituents is having the support of the higher levels of government, which can ensure that funds are appropriately allocated. Political will further influences the sustainability of the results, and the possibility of a change in administration poses a risk to programmes that may be cut due to high association with the outgoing regime.

It is important at this point to also highlight two broad issues which determine the effectiveness of programmes, relating to intervention design and implementation fidelity. There are two main reasons why we might not expect to see the intended impacts of a programme implemented in the “real world” (Bamberger et al., 2010). The first is that the programme design is inappropriate – that is the underlying mechanisms that drive change are not appropriate for the context in which the programme is based, or for particular groups of participants in that context (Pawson, 2006). According to van der Knaap et al. (2007: 3), “mechanisms are the engines behind behavior, which are often not immediately recognizable… They [include] people's efforts to give way to group pressure (groupthink), people's efforts to be status‐congruent with others or to avoid or reduce cognitive dissonances, or people's desire to be an early adopter of an innovation. [T]he action of mechanisms to some extent depends on the context in which they are used… Behavioral change is achieved through this context”.

An example would be a community driven development programme that is supposed to rely on community participation to foster social cohesion, but is unable to support the appropriate level of participation, and therefore cohesion, because people are not comfortable speaking in public meetings due to elite capture (White, Menon, & Waddington, 2018). Similarly, interventions to decentralise decision making in schools are less likely to be effective in low income, low education contexts where communities have low status relative to school staff (Carr‐Hill et al., 2018). Another example would be a women's empowerment programme which is ineffective in reaching a particular group of participants (e.g. women from Muslim households) because it does not take into consideration the need to involve community leaders in design of the programme targeting strategy.

Such “failure mechanisms” will vary based on intervention design and targets; for example, in some cultures, traditional community leaders may be critical stakeholders to engage in interventions seeking to change the equity of or access to services, despite the disconnect between their de facto and de jure power – but only depending on the service targeted. Baldwin and Raffler (2016) argued that traditional leaders are often highly socially accountable for public services such as conflict resolution or natural resource management, but less so for services such as education or health care. In that case, an intervention targeting equitable access to and use of public land may fail if it does not engage traditional leaders, but a similar intervention simply targeting equitable access to and use of health services may still be successful. Failures may also come in the form of unintended consequences; for example, Chong et al. (2014) found that increasing the dissemination of corruption information to voters in Mexico decreased support not only for exposed corrupt politicians, but also for all political parties, and led to a decrease in voter turnout.

The second reason is due to implementation failures for a programme that otherwise (in theory) would be effective in the implementation context. Examples would be technical and logistical problems relating to project delivery (e.g. inadequate training and support to practitioners); weaknesses in implementer systems (e.g. human resource, financial or monitoring); or due to external factors (e.g. conflict, natural disasters).

### 3.4 Why it is important to do this review

The 2017 *World Development Report* (World Bank, 2017) posits that rather than asking which policies to implement, the global development community needs to ask what enables policies to achieve sustainable outcomes, the answer to which being better governance. This report is timely in a context in which donors are actually moving away from funding governance projects. Data from the OECD Creditor Reporting System of Official Development Assistance (ODA) shows declining funding in the government and civil society sector, from US$ 14.5 billion in 2009 to around US$ 12 billion in 2015, a decrease of almost 20 per cent. During the same time period, overall ODA increased from US$ 103 billion to US$ 118 billion (OECD, 2017). Therefore, it appears that the share of aid to governance and civil society also fell from around 14 per cent to 10 per cent, or an increasing share that was traditionally counted under governance is instead being incorporated into sector programming (health, education, agriculture, infrastructure, etc).

Governance programmes are implemented in complex socio‐political contexts, and involve many challenges in realising, demonstrating, and attributing improvements towards key outcomes. USAID (2017) notes that the lack of consistent definition of governance and poor understanding and weak documentation of evidence of governance‐related interventions contribute to a reticence to invest in such programmes. This could explain why donors are shifting their attention towards other sectors; over the same time period (2009 to 2015), funding for economic infrastructure and services increased by US$ 7.5 billion, while funding for health programmes increased by US$ 700 million (OECD, 2017).

In addition, prominent single study evidence has questioned the viability of bottom‐up, community‐based approaches, as compared to top‐down government accountability (Olken, 2007). However, it is not clear whether the findings from single studies are transferable to other contexts. This points to the need to strengthen the synthesis and dissemination of the evidence base, and to encourage decision makers to draw on systematic evidence collected from the implementation of programmes in multiple contexts.

This systematic review examines interventions that promote more effective and responsive public services and institutions, defined under Sustainable Development Goal number 16 as institutions that “deliver equitable public services and inclusive development at the central and local levels, with a particular focus on restoring core government functions in the aftermath of crisis and attention to local governance and local development” (UNDP, 2016).

The review makes two main contributions. The first is to provide systematic evidence on PITA for citizen engagement in development programming (outside of the education sector) in L&MICs. Molina et al. (2016) presented a systematic review of community monitoring studies in L&MICs. King, Samii, and Snilstveit (2010) and White et al. (2018) systematically reviewed community driven development. Hanna, Bishop, Nadel, Scheffler, and Durlacher (2011) systematically reviewed anti‐corruption interventions and Lynch et al. (2013) reviewed of the effect of interventions that improve community accountability on service delivery and corruption.^1^ Other systematic reviews have focused on education governance (Guerrero, Leon, Zapata, Sugimaru, & Cueto, 2012; Carr‐Hill et al., 2015; Snilstveit et al., 2015). Relevant non‐systematic evidence syntheses include Olken & Pande (2013), Azulai et al. (2014), Dal Bó and Finan (2016), Brinkerhoff, Jacobstein, Kanthor, Rajan., and Shephard (2017) and the Metaketa project (EGAP 2018).

The second main contribution of the review is to undertake the systematic review and meta‐analysis to Campbell Collaboration standards while also aiming to extract the mechanisms underlying programmes and reporting those systematically. We did so by including certain types of comparison groups that would enable us to extract the effect of the PITA mechanism over standard access to public services (or a different PITA mechanism). We also systematically extracted information about the contextual factors and mechanisms and through which programmes operate systematically, based on the included studies and related programme and project documents, and synthesised those using a framework synthesis approach.

As policy makers and implementers work to ensure the sustainability of their investments and interventions, institutionalising good governance practices will become increasingly important. This systematic review assesses the effectiveness of interventions that target participation, inclusion, transparency and accountability in the design and delivery of public services and institutions on development outcomes. Analysis of causal pathways and mechanisms will shed light on the contexts in which these interventions can be successful and corresponding enabling factors. The review aims to provide evidence on what is generalisable, what is context specific, in what ways, and for whom in external accountability governance programming.

## 4 OBJECTIVES

The primary objective of this review was to identify, appraise and synthesise evidence that answers the question: to what extent are programmes in low‐and middle‐income countries targeting effective and responsive public services and institutions that incorporate PITA characteristics into their design effective in achieving their objectives, as compared to otherwise similar programmes that do not? Authors compared the effectiveness of different types of programmes that incorporate PITA characteristics, both by intervention sub‐group and by which PITA mechanism(s) the intervention incorporates, using an innovative, integrated mixed‐methods approach that drew on both quantitative meta‐analysis (Review Questions 1‐3) and qualitative realist‐informed framework synthesis approaches that were then reintegrated with the meta‐analysis (Review Question 4).

The secondary objectives were to assess how effects varied by population group and location, to identify the factors relating to programme design, implementation, context, and mechanism that are associated with better or worse outcomes along the causal chain and assess the evidence on programme costs. To address these last two objectives, the review included additional programme design and implementation documents as well as cost data where possible. The review aimed to answer the following specific questions:

*Primary review questions*

1) What are the effects of interventions that aim to strengthen the PITA characteristics of public services or institutions on social and economic wellbeing for participants? (*Review Question 1*).
2) What are the effects of interventions that aim to strengthen the PITA characteristics on participatory, inclusive, transparent or accountable processes? (*Review Question 2*). *Secondary review questions*
3) To what extent do effects vary by population group and location? (*Review Question 3*).
4) What factors relating to programme design, implementation, context, and mechanism are associated with better or worse outcomes along the causal chain? (*Review Question 4*).
5) What evidence is available on programme costs and incremental cost effectiveness in included studies of effects? (*Review Question 5*).

## 5 METHODS

As described in the protocol published in the Campbell Library (Waddington et al. 2018), the review followed Campbell and Cochrane Collaborations guidance for systematic reviews (The Steering Group of the Campbell Collaboration, 2016; Higgins & Green, 2011; Kugley et al. 2017; Shadish & Myers, 2004). The approach also drew on previous approaches to incorporate theory into systematic reviews by analysing the causal chain and drawing on qualitative evidence (e.g. Snilstveit et al., 2015; Waddington et al., 2014; White et al., 2018). To address review questions 1, 2 and 3, authors used counterfactual evidence provided in quantitative causal studies (impact evaluations) and used analysis of effect size data (statistical meta‐analysis) to explore the central tendency and heterogeneity for outcomes measured along the causal chain. To address review question 4, the approach drew on realist synthesis (Pawson, 2006; Van der Knaap, Leeuw, Bogaerts, & Nijssen, 2008) and framework synthesis (Carroll et al., 2013), and incorporated multiple types of evidence, including programme and project documents to provide information about context and mechanism characteristics. The review also presents cost data from included impact studies (question 5).

### 5.1 Criteria for considering studies for this review

The criteria determining eligibility of studies in the review are summarised in Table 1.

**Table 1:** Summary of criteria for inclusion and exclusion of studies

Criteria	Inclusion definition
**Population**	Programme participants in LMICs were included. Programme participants in high‐income countries were excluded.
**Interventions**	Interventions with PITA components that targeted the means and mechanisms through which public institutions and services engage with constituents (service users) were included. Interventions that bundled PITA components alongside other programme components such as block grants (e.g. community‐driven development), or that aimed to strengthen internal or sideways PITA, or those in the education sector were excluded.
**Comparisons**	Populations that received “business as usual” service access, or an intervention with a different type or degree of PITA were included.
**Outcomes**	Intermediate and endpoint, intended or unintended outcomes at participant and project level were included. Outcomes relating to political processes (e.g. voting) were excluded. Immediate outcomes relating to citizen engagement (e.g. participation in meetings) or public service response (e.g. public spending) were eligible for the review provided that outcomes relating to access to services (e.g. facilities construction) or intermediate outcomes (e.g. service use) or final outcomes (e.g. health, nutrition, state‐society relations) were also reported.
**Study designs**	Counterfactual studies (review questions 1‐4), including relevant programme and project documents providing information on design and implementation (review question 4) and cost evidence provided in counterfactual studies (review question 5) were included.

### 5.1.1 Types of studies

To answer Review Questions 1, 2 and 3 the review included counterfactual studies that used an experimental or quasi‐experimental design and/or analysis method to measure the net change in outcomes that were attributed to an intervention or policy. The review included randomised and non‐randomised studies that were able to take into account confounding and selection bias (Reeves, Wells, & Waddington, 2017; Waddington et al., 2017). Specifically, the following study types were includable:

- Randomised controlled trials (RCTs), with assignment at individual, household, community or other cluster level, and quasi‐RCTs using prospective methods of assignment such as alternation.
- Non‐randomised studies with selection on unobservables:
  o Regression discontinuity designs, where assignment was done on a threshold measured at pre‐test, and the study used prospective or retrospective approaches of analysis to control for unobservable confounding.
  o Studies using design or methods to control for unobservable confounding, such as natural experiments with clearly defined intervention and comparison groups, which exploit natural randomness in implementation assignment by decision makers (e.g. public lottery) or random errors in implementation, and instrumental variables estimation.

- Non‐randomised studies with pre‐intervention and post‐intervention outcomes data in intervention and comparisons groups, where data were individual level panel or pseudo‐panels (repeated cross‐sections), which used the following methods to control for confounding:
  o Studies controlling for time‐invariant unobservable confounding, including difference‐in‐differences, or fixed‐ or random‐effects models with an interaction term between time and intervention for pre‐intervention and post‐intervention observations;
  o Studies assessing changes in trends in outcomes over a series of time points (interrupted time series, ITS), with or without contemporaneous comparison (controlled ITS), with sufficient observations to establish a trend and control for effects on outcomes due to factors other than the intervention (e.g. seasonality).

- Non‐randomised studies with control for observable confounding, including non‐parametric approaches (e.g. statistical matching, covariate matching, coarsened‐exact matching, propensity score matching) and parametric approaches (e.g. propensity‐weighted multiple regression analysis).

Analysis under Review Question 4 addressed programme design, implementation, context and mechanism in greater detail. Authors extracted descriptive information about each programme evaluated in included counterfactual studies, as well as from additional programme and project design and implementation documents relating to each of these. Information on underlying context and behavioural mechanisms drew on information contained anywhere in included study reports, whereas evidence on outcomes drew on effects data from relevant study arms in quantitative counterfactual estimation only.

Analysis under Review Question 5 aimed to address unit cost, cost‐efficiency, cost‐effectiveness or benefit‐cost evidence on interventions in particular contexts. This review aimed to incorporate economic evaluations of included programmes drawing on standard approaches to synthesis of economic appraisal evidence (Shemilt et al., 2011). However, authors only identified four studies that reported any cost information. They are reported descriptively in the results (Review Question 5).

Eligible comparators for review questions 1‐3 included groups that received normal service delivery (“business as usual”) without improved PITA characteristics, or groups that received an intervention testing the inclusion of different PITA design characteristics or weaker or less intensive implementation of PITA design characteristics.

### 5.1.2 Types of participants

This review included any participants from low‐and middle‐income countries (L&MICs), including participants from the general population and those from specific population sub‐groups. Authors collected data on differential effects and experiences for sub‐populations available and coded information according to the PROGRESS‐plus criteria, where progress stands for place of residence, race/ethnicity, occupation, gender, religion, education, socioeconomic status, and social capital, and “plus” represents additional categories such as age, disability, and sexual orientation (O’Neil et al. 2014).

### 5.1.3 Types of interventions

This review included interventions that aimed to increase the external engagement by public institutions and services with citizens and service users. Authors defined external engagement interventions as either stand‐alone interventions or interventions that formed part of a larger programme that inherently or by definition sought to improve the PITA‐characteristics of engagement between public services and institutions and citizens. They could be implemented either on the supply or demand side of service delivery, or target both simultaneously, for example through the introduction of public‐service audits that worked with both the community and civil servants.

To be included in the review, the intervention needed to improve the effectiveness and responsiveness of institutions’ engagement with constituents. Authors grouped eligible interventions as follows:

- *Participation:* The intervention promoted or formalised continuous citizen input in the design and implementation of public services, processes or policies. Eligible interventions were:

o **Participatory priority setting**, planning or budgeting, including participatory budgeting and healthcare committees, where a specific group of citizens participates in the health priority setting, planning and management of local health services.
o **Community‐based natural resource management (CBNRM)** committees such as forest user groups (FUGs), participatory forest management (PFM), water user associations (WUAs).

- *Transparency:* The intervention involved the disclosure and/or dissemination of information (rules, plans, processes, prices and actions) regarding the governance of public services or institutions, with the aim of changing power relations between service providers and users. Included interventions were:
  o **Rights information**, where information provided about service user rights that allows users to demand better quality or minimum quality services.
  o **Performance information**, including score cards, in which information is disseminated about the quality of services, and public audits, in which a government line department presents their budget and achievements to their constituents.

- *Accountability:* The intervention encompassed monitoring to encourage or actively hold individuals, public service providers and institutions responsible for executing their powers and mandates according to a certain standard. Included interventions were:

o **Citizen feedback mechanisms**, which allow citizens to feedback concerns or priorities around service delivery to providers, and / or to monitor the delivery of public service delivery. This category also includes social audits, whereby public forums bring together a service provider with local authorities, neighbours, and representatives, to monitor the delivery of a specific project.

- *Inclusion:* This covers the promotion of participation, transparency and accountability for marginalised and vulnerable groups such as women, ethnic minorities or lesbian, gay bisexual, transgender and intersex (LGBTI) people. Eligible interventions are:

o **Quotas** for women or minority group representation in participatory budgeting (participation) or community development committee (accountability), or information provided about service user rights of women or minority groups (transparency).

### 5.1.4 Types of outcome measures

This review included studies that reported outcomes measuring improvement in access to services, service behaviours, attitudes towards services, including user satisfaction, social and economic quality of life improvements for the proposed intervention, and “state legitimacy” (state‐society relations). The inclusion criteria for outcomes were broad in order to be able to provide a full picture of the effects of the included interventions along the causal chain, described in Table 2.

**Table 2:** Types of outcomes along the causal chain

	Secondary outcomes		Primary outcomes				
**Causal chain area**	Service user engagement	Service provider response	Service access and quality	Service use	Attitudes to services	Wellbeing outcomes	State‐society relations
**Outcomes measured in included studies**	Knowledge about services	Public spending	Facilities construction	Use of services: vaccination, antenatal/ postnatal care, family planning	User satisfaction	Heath outcomes: morbidity, mortality, fertility	Satisfaction with government
	Participation in meetings	Perceived response by users	Reliability of services	Quantity of service used (e.g. irrigation water)	Complaints reported	Nutrition	Payment of tax
	Freedom of participation	Project staff motivation	Measured quality of services availableStaff absenteeism		Perceived quality of care provided	Agricultural yields	Confidence in institutions
			Embezzlement/ leakages			Income and expenditure	
			Access to forestry or natural resources			Assets	
						Crime rates	
						Feelings of security	
						Satisfaction with life	

#### 5.1.5 Primary outcomes

intermediate outcomes were eligible that measured service access or quality (block 5 in the theory of change), use or user satisfaction (block 6) and endpoint outcomes measuring social or economic wellbeing for individuals in the relevant sector (block 7) or state legitimacy (block 8). Examples of wellbeing outcomes include: morbidity or mortality; income, wealth or poverty status; nutritional status or food security; resilience to shocks; crime rates. Studies needed to report primary outcomes relating to service delivery, wellbeing or state‐society relations to be included in the review.

#### 5.1.6 Secondary outcomes

“immediate outcomes” measuring citizen engagement with public institutions and services, such as participation in decision‐making, inclusion, transparency and accountability, and responsiveness of public services and public service delivery agents, such as public spending, leakages and corruption.

#### 5.1.7 Duration of follow‐up

The review included any follow‐up duration, coding multiple outcomes where studies report multiple follow‐ups. Several studies presented multiple follow‐ups, which are reported in the descriptive results section.

#### 5.1.8 Types of settings

Interventions could be implemented in any low‐ or middle‐income country, as defined by the World Bank at the time the intervention was implemented.

#### 5.1.9 Other

The review included both completed and ongoing studies, including protocols of ongoing studies that met all other inclusion criteria and/or studies listed in registries of ongoing impact evaluations.

The review included studies published in any language, although all included studies were in English. The review was limited to included studies published in 2000 or after, following Phillips et al. (2017) and because authors did not expect to identify any impact evaluations that met the criteria from before this date.

Table 3 gives some further examples of decisions for including and excluding similar types of studies.

**Table 3:** Reasons for inclusion and exclusion of similar interventions

Include	Exclude	Rationale
Intervention: *Tuungane* Humphreys et al. (2012)	Intervention: Mandated political representation for women (Iyer et al., 2011)	Both these interventions incorporate quotas to ensure women's participation. However, in Humphreys et al. (2012) the intervention creates quotas for women's participation *in an external citizen engagement intervention*, whereas the Iyer et al. 2011 study targets the “I” characteristics of the formal political system, which is not the governance domain of focus for this review.
Country: Democratic Republic of Congo (DRC)	Country: India	
PITA: P, I	PITA: I	
Summary: The *Tuungane* evaluation measures the impact of the social mobilisation interventions of this CDR project through an experiment in which both treatment and control communities receive a small grant, and their inclusive decision‐making capacities are evaluated (P). Further, intervention communities were randomly assigned to require gender parity in decision‐making groups or not, and thus the value‐add of quotas for women's participation can be isolated (I). In this case, the quotas ensure women citizens are able to contribute to community decision‐making, on par with male citizens.	Summary: This paper looks at the impact of introducing quotas for women's participation in local government councils (I). However, these are elected positions wherein the incumbents are formal government employees. Thus, while such a change may impact women citizen's access to public officials, it does not create specific opportunities for private citizens to engage with public officials.	
Intervention: Joint Forestry Management Persha and Meshack, (2016)	Intervention: Decentralisation of Water Supply Asthana (2012)	Though both cases focus on the management of common‐good natural resources, and both look at the impact of decentralisation, the Asthana (2012) study only devolves power from one level of government to a lower level, and thus resides within the sphere of internal systems management, as citizens are not engaged in the process. The Persha and Meshak 2016 study, in contrast, empowers communities to create their own rules for managing natural resources, which may differ from state‐level rules.
Country: Tanzania	Country: India	
PITA: P	PITA: T	
	Summary: This study evaluates	
Summary: This intervention devolves control over common resource management completely, from the government to communities (P). Thus, communities are empowered to create their own rules for natural resource use, and they share accountability with the government for the enforcement of those rules.	the impact of decentralisation from state‐level government to local government. Thus, though the intervention was designed to reduce corruption, it does not engage citizens in the process or create specific opportunities for them to engage.	
Intervention: Citizen Report Cards Björkman, Reinikka, and Svensson (2006)	Intervention: MIRA Makwanpur Manandhar et al. (2004)	Though both of these health‐sector interventions work to identify challenges and develop action plans to improve outcomes, the Björkman, Reinikka, and Svensson (2006) study enables citizens to hold public health providers accountable for delivering services, and jointly develops strategies for improvement to which the health providers are accountable. In Manandhar et al. (2004), the women's groups are empowered to take responsibility for their own healthy practices; there are no requirements on the health service providers to take responsibility for addressing challenges the women identify. The intervention aims to change health outcomes *outside* the sphere of public service delivery.
Country: Uganda	Country: Nepal	
PITA: A	PITA: P	
Summary: This study looks at the impacts of an intervention in which “report cards” of health service provision were disseminated amongst communities (T), and a series of interface meetings between service providers and citizens were organised to review the reports and identify an action plan for improvements (A).	Summary: This intervention formed community‐based, participatory women's health groups with the aim of identifying key local challenges and potential solutions (P), with the ultimate goal of improving birth outcomes.	
Intervention: Raskin subsidy identification cards Banerjee, Hanna, Kyle, Olken, and Sumarto (2018)	Intervention: Ciudad Mujer (Women's City) Bustelo, Martinez, Millard, and Silva (2016)	Both of these interventions aim to increase citizens’ knowledge of their rights to access services (or public subsidies). However, the intervention in Bustelo et al. (2016) was purely about access to services; it did not aim to change the way that women engaged with public service providers, except to encourage them to take advantage of the services. In contrast, the experiment in Banerjee et al. (2018) had the explicit aim of attempting to reduce corruption in the subsidy programme by limiting service providers’ ability to direct who received the subsidy and who didn’t. Thus, in this latter case, the change in knowledge changes the power relations between service provider and user.
Country: Indonesia	Country: El Salvador	
PITA: T	PITA: T	
Summary: This study presents the results of an experiment in which recipients of the Raskin food subsidy were sent cards confirming their right to the subsidy; an alternative intervention in which lists of eligible households in communities were publicly displayed; and a control set where there were no changes in publication of eligibility for the subsidy. The aim was to test the effect of these different transparency initiatives on reducing corruption in subsidy provision.	Summary: This intervention created “one stop shops” for a variety of public services targeted to women, under the auspices of a health facility. When women arrived, they would take part in an orientation that explained all of the different services they could access at the facility, improving their knowledge of their rights to services (T).	
Intervention: random federal government audits of transfers to sub‐national government and publication of results to citizens Timmons and Garfias (2015)	Intervention: increase in the number of government audits (Olken (2007), audit arm)	Both interventions use a “top‐down” audit to improve accountability. In the case of Olken (2007), the audit is undertaken by the government auditor (which constitutes an “internal accountability” intervention by our definitions) and is presented to communities (which constitutes a transparency intervention for “external accountability”). The probability of being audited is known to be very low in control arms, whereas it is known to be 100 per cent in treatment arms. Hence the study is not able to disentangle the effect of the internal and external accountability interventions and is therefore excluded from the review.
	Country: Indonesia	
Country: Brazil	PITA: A	
PITA: T		
		In contrast, the probability of audit in Timmons and Garfias (2015) is randomly determined; the threat is equal in all municipalities. We therefore consider that the main mechanism being evaluated is the publication of the results of the audit to citizens. The study thus evaluates the effect of providing performance information to enable citizens to hold public officials accountable, with the aim of changing power relations between public officials and citizens.

### 5.2 Search methods for identification of studies

Authors developed the systematic search strategy in consultation with an information specialist (John Eyers) to cover comprehensively the published and unpublished literature, following systematic search guidelines in Kugley et al. (2017). The review also drew upon, and expanded, the search terms used in the evidence map by Philips et al. (2017) and harvested terms from the papers included in that map that were eligible for inclusion in this review. To reduce the potential for publication bias, the search included both academic databases as well specialist organisational websites, websites of bilateral and multilateral agencies and repositories of impact evaluations in international development. The full list of databases searched is below. The substantive scope of this review is cross‐sectoral and therefore in addition to general sources of social science research, authors searched several sector specific databases, for example databases of health, governance and public management. Authors searched for studies published in 2000 or after up until 5^th^ February for the bibliographic databases and 3^rd^ March 2018 for the grey literature databases.

Search terms for the academic databases can be found in Appendix 1. Separate search strings were developed for the two academic health databases to capitalise on MeSH terms, to remove non‐health related terms and add some specific health‐related intervention terms (Medline and Global Health). The search strings combine specific intervention terms, study design terms and terms for low‐and middle‐income countries.

A simplified series of search strings was developed for searching the grey literature, wherein the search engines are not as sophisticated as the academic databases and cannot handle the same detailed strategy. Due to the broad scope of the review, and in order to ensure the grey literature search was exhaustive, a series of PITA search strings were developed. These focused on PITA terms such as participatory or participation. An intervention‐based strategy, more similar to the academic database strategy, was piloted, but discarded due to the number of individual searches per site that were required for an exhaustive search, rendering it inefficient. Population and study type terms were not included, because the advanced search options within the grey literature search engines were not sophisticated enough to allow for an “or” limiter for each L&MIC and methodology. The broad study type term “impact evaluation” was added alongside each search to improve the relevance of results. See Appendix 2.

### 5.2.1 Electronic searches

Authors searched the following academic databases in January and February 2018:

- CAB Global Health (Ovid): http://www.ovid.com/site/catalog/databases/30.jsp
- Econlit (Ovid): http://www.ovid.com/site/catalog/databases/52.jsp
- Medline (Ovid): http://www.ovid.com/site/catalog/databases/901.jsp
- Scopus: https://www.scopus.com/
- Social Sciences Citation Index (SSCI) (via Web of Science): https://webofknowledge.com/.

Authors searched the following specialist organisational databases between 5 and 20 March 2018^2^:

- CARE International: http://www.careevaluations.org/
- Catholic Relief Services: https://www.crs.org/our‐work‐overseas/research‐publications
- Centre for Public Impact: https://www.centreforpublicimpact.org/observatory/
- Chemonics International: https://www.chemonics.com/technical‐areas/democracy‐and‐governance/
- EGAP (Evidence in Governance and Politics): http://www.egap.org/
- International Growth Centre (IGC) at LSE: https://www.theigc.org/publications/
- International Rescue Committee (IRC): https://www.rescue.org/reports‐and‐resources
- Mercy Corps: https://www.mercycorps.org/research
- Oxfam International: https://policy‐practice.oxfam.org.uk/publications
- RTI International: https://www.rti.org/publications
- Samuel Hall (evaluations): http://samuelhall.org/category/publications/
- Transparency International (TI): https://www.transparency.org/
- U4 Anti‐Corruption Resource Centre: http://www.u4.no/publications/

Authors searched the following bilateral and multilateral agencies and general repositories of impact evaluations in international development from 5 to 29 March 2018:

- 3ie Repository of Impact Evaluations http://www.3ieimpact.org/en/evidence/impact‐evaluations/
- 3ie RIDIE (Registry for International Development Impact Evaluations): http://ridie.3ieimpact.org/
- African Development Bank (AfDB): https://www.afdb.org/en/documents/publications/
- Asian Development Bank (ADB): https://www.adb.org/publications
- BREAD: http://ibread.org/bread/papers
- Center for Effective Global Action (CEGA): http://cega.berkeley.edu/evidence/
- Design, Monitoring and Evaluation for Peace: www.dmeforpeace.org/learn/resources/
- DFID Research for Development (R4D): http://r4d.dfid.gov.uk/
- GEF (Global Environmental Facility) evaluation database: http://www.gefieo.org/evaluations/all?f[0]=field_ieo_grouping%3A312
- Global Facility for Disaster Reduction and Recovery: https://www.gfdrr.org/en/publication
- Innovations for Poverty Action (IPA): http://www.poverty‐action.org/projectevaluations
- Inter‐American Development Bank Publications: https://publications.iadb.org/facet‐view?locale‐attribute=en&field=type_view
- J‐Poverty Action Lab (J‐PAL): https://www.povertyactionlab.org/evaluations
- Global Facility for Disaster Reduction and Recovery: https://www.gfdrr.org/en/publications
- Locus (International Development Coalition): https://locus.ngo/resources
- Prevention Web (UNIDSR): https://www.preventionweb.net/english/professional/
- RePEc (via EBSCO Discovery): https://www.ebscohost.com/discovery
- World Bank E‐Library (via EBSCO Discovery): https://www.ebscohost.com/discovery
- United Nations Evaluation Group: http://www.uneval.org/evaluation/reports
- USAID Development Clearing House: https://dec.usaid.gov/dec/home/Default.aspx.

### 5.2.2 Other searches

The review used the evidence gap map of state‐society relations as a primary source of potential studies (Phillips et al., 2017). In addition, authors screened the bibliography of existing systematic reviews and literature reviews, including Molina et al., (2016), Lynch et al. (2013) and Hanna et al. (2011). Authors also screened the reference lists of included studies and undertook forward citation‐tracking for those studies using Google Scholar. Authors contacted the review's advisory group and the funder of the systematic review, USAID, to identify additional studies.

### 5.2.3 Targeted searches for studies to address Review Question 4

In order to answer question 4 relating to programme design, implementation, mechanisms and context, authors attempted to identify programme and project documents associated with the programmes in the impact studies identified in the first stage of the search. This was done through a targeted search for programme names and study authors using Google and Google Scholar. The reference lists of included studies were also screened for programme and project documents. Evidence on context and mechanisms were collected from any studies eligible for Review Questions 1‐4. Programme mechanisms may have been suggested by study authors or identified by the review team. Authors collected additional contextual information not provided in included studies using international data, for example the World Development Indicators (World Bank) or the “Polity IV” governance index (Marshall, Jaggers, & Gurr, 2011; as also used in Lawry et al., 2014).

### 5.2.4 Studies to address Review Question 5

The review aimed to incorporate and synthesise economic evaluations and cost data that were presented in the included studies. Only four studies presented any cost data. These are presented in the results section.

### 5.2.5 Selection of studies

All search results were imported into EPPI‐Reviewer 4 and duplicates removed. At the title and abstract stage, authors used innovative text mining technologies to reduce the initial screening workload (O’Mara‐Eves, Thomas, McNaught, Miwa, & Ananiadou, 2015). Authors used two functions in EPPI Reviewer 4 to do this: the priority‐screening function and inclusion/ exclusion classifier (Thomas, McNaught, & Ananiadou, 2011; O’Mara‐Eves et al., ibid). The priority screening function can be used at the title and abstract screening stage to prioritise the items most likely to be “includes” based on previously included documents. This involved independent double screening a random test set of citations to train the priority screening function, which learned to identify relevant records based on key‐words in the title and abstract of the included and excluded studies. All team members were involved at this stage of screening. The function continues to learn as screening progresses. Using priority screening in this way allows for the identification of includable records at an earlier stage in the review process so that work can begin earlier on full‐text screening and data extraction. This review also used the priority screening function to classify studies into groups based on their probability of inclusion in the review. Authors conducted piloting and verification of the screening function, and excluded studies with a low probability of inclusion (<20% probability of inclusion) automatically from the review. Authors screened a random 10 per cent sample of the automatically excluded studies as a check on accuracy of the function. The results of this process are presented in the search results section. The review team independently double screened all studies with 20‐99 per cent certainty of inclusion. If a title and abstract did not present enough information to definitively include or exclude a study, it was included for full‐text screening. Disagreements on inclusion or exclusion were resolved by discussion and the input of a third author if necessary.

Studies included for full‐text screening were double screened by two independent authors. Disagreements on inclusion or exclusion were resolved by discussion and the input of a third author if necessary.

Screening of studies intended to address Review Question 4 took place in a second stage of screening. Studies were assessed for relevance by one author to determine whether they covered one of the programmes included to answer Review Questions 1‐3. Each of these studies were then assessed for relevance by at least one other author.

### 5.3 Data extraction and management

Authors extracted the following descriptive, methodological, qualitative and quantitative data from each included study using a standardised data extraction form (data extraction form provided in Appendix 3):

- Descriptive data including authors, publication date and status as well as other information to characterise the study including country, type of intervention and outcome, population, context, type of intervention.
- Methodological information on study design, analysis method, type of comparison (if relevant) and external validity.
- Quantitative data for outcome measures, including outcome descriptive information, sample size in each intervention group, outcomes means and standard deviations, test statistics (e.g. t‐test, F‐test, p‐values, 95% confidence intervals), cost data, and so on.
- Information on intervention design, including how the intervention incorporates participation, inclusion, transparency and accountability characteristics, participant adherence, contextual factors and programme mechanisms.

Authors extracted quantitative data for outcomes analysis using Excel. Two authors independently calculated effect sizes for a random sample of 20 per cent of the included studies, reaching agreement in all except two cases, which the lead author resolved. Disagreements on inclusion or exclusion were resolved by discussion and the input of a third author if necessary. The rest of the quantitative data was extracted by one author only. Authors extracted descriptive, methodological and qualitative data using KoBo Toolbox. Descriptive and qualitative data were single coded by one author and checked by a second author. One author also checked the coding of intervention characteristics and mechanisms coded by others.

#### 5.3.1 Assessment of risk of bias in included studies

The critical appraisal results for each included study are reported (**Critical appraisal of included studies**).

##### Assessment of risk of bias in experimental and quasi‐experimental studies (Review Questions 1‐3)

Authors assessed the risk of bias in the included quantitative counterfactual studies (impact evaluations) drawing on the signalling questions in the 3ie risk of bias tool, which covers both internal validity and statistical conclusion validity of experimental and quasi‐experimental designs (Hombrados and Waddington, 2012) and the bias domains and extensions to Cochrane's ROBINS‐I tool and RoB2.0 (Sterne et al., 2016; Higgins et al., 2016).The risk of bias tool developed for this review can be found in Appendix 3. This review noted any potential differences in methods and risk of bias for different outcomes reported in each paper.

The review assessed risk of bias of included studies based on the following criteria, coding each paper as “Yes,” “Probably Yes,” “Probably No,” “No” and “No Information” according to sub‐questions relating to the following bias domains:

- Causal inference: Factors relating to baseline confounding and biases arising from differential selection into and out of the study (attrition);
- Deviation from intended intervention: Factors relating to biases due to performance bias (e.g. cross‐overs, contaimination and survey effects) and motivation bias (Hawthorne effects);
- Outcomes data collection: Factors relating to biases in outcomes data collection (e.g. social desirability or courtesy bias, recall bias);
- Analysis reporting: Factors relating to biases in methods of analysis and reporting.

We used the following decision rule to assign a risk of bias rating for each domain:

- “High risk of bias”: if any of the criterion within that domain were assessed as “No” or “Probably No”.
- “Some concerns”: if one or several criterion within that domain were “Unclear” and none were “No” or “Probably No”.
- “Low risk of bias”: if all of the criterion within that domain were “Yes” or “Probably Yes”.

Finally, we used the decision rule of RoB2.0 (Higgins et al., 2016) to reach an overall risk of bias judgment:

- “High risk of bias”: if any of the bias domains were assessed as being “high risk”.
- “Some concerns”: if any of the bias domains were “some concerns” and none were “high risk”.
- “Low risk of bias”: if all of the bias domains were assessed as “low risk”.

Two authors independently assessed risk of bias from a random sample of 20 per cent of the included studies. For the experimental studies, the two authors agreed on 64 per cent of the decisions for each criterion for each study. However, 33 per cent of the disagreements were cases where the two authors answered yes versus probably yes or unclear, or no versus probably no or unclear.In only three per cent of cases did the authors fully disagree on whether a study had addressed a risk of bias domain, that is, one author had answered yes and one author had answered no. For the quasi‐experimental studies, the two authors agreed on 76 per cent of the decisions for each criterion for each study. For the remaining 24 per cent, the two authors had answered either yes and unclear or no and unclear. Disagreements on bias assessments were resolved by discussion and the input of a third author if necessary. The disagreements and their resolutions are reported alongside the detailed results of the Critical Appraisal in Appendix 5. One author undertook remaining risk of bias assessments and discussed uncertain cases with a second or third author as necessary. Following the independent double assessments, one author re‐assessed all remaining studies on the criteria that had been clarified as part of the process.

##### Critical appraisal of project design and implementation (Review Question 4)

It was not necessary to critically appraise the information extracted on programme design, implementation and context from the project documents as this information was descriptive.

##### Critical appraisal of cost evidence (Review Question 5)

The review identified cost data in four studies, most of which only presented intervention cost per beneficiary, and as some authors of included studies acknowledged, unit cost estimates were “back of the envelope” calculations. Authors assessed the quality of the cost evidence, using the tool provided by Evers, Goossens, de Vet, van Tulder, and Ament (2005) as recommended in the Campbell Collaboration Economic Methods Policy Brief (Shemilt, 2008).

### 5.3.2 Measures of treatment effect

An effect size expresses the magnitude or strength of the relationship of interest (Borenstein, Hedges, Higgins, & Rothstein, 2009). To address questions 1, 2 and 3, authors extracted data from each individual study to calculate standardised effect sizes for cross‐study comparison. To ensure comparability across outcomes, authors transformed each measure so that an increase indicates an improvement (hence the sign was reversed for any variables measuring negative outcomes like mortality and absenteeism).

For continuous outcomes comparing group means in a treatment and control group, authors calculated the standardised mean difference (SMDs), measuring the mean difference in standardised units of the variance of the outcome. This review calculated SMD as Cohen's *d* along with standard error using formulae provided in Borenstein et al. (2009), which was adjusted to account for small sample bias using Hedges’ *g* method (Ellis, 2010):

$$g≅d\left(1-\frac{3}{4(n_{1}+n_{2})-9}\right)$$

Formulas for effect size calculations were used depending on data provided in included studies. For example, for studies reporting means (X) and pooled standard deviation (SD) for treatment (T) and control or comparison (C) at follow up (p+1) only:

$$d=\frac{x_{\mathrm{Tp}+1}-x_{\mathrm{Cp}+1}}{\mathrm{SD}}$$

If the study did not report the pooled standard deviation, but reported the standard deviations of outcome in each group, SD was calculated as follows:

$${\mathrm{SD}}_{p+1}=\sqrt{\frac{(n_{\mathrm{Tp}+1}-1){\mathrm{SD}}_{\mathrm{Tp}+1}^{2}+(n_{\mathrm{Cp}+1}-1){\mathrm{SD}}_{\mathrm{Cp}+1}^{2}}{n_{Tp+1}+n_{Cp+1}-2}}$$

For studies reporting means ($\underline{X})$ and standard deviations (SD) for treatment and control or comparison groups at baseline (p) and follow up (p+1):

$$d=\frac{{∆\underline{X}}_{p+1}-{∆\underline{X}}_{p}}{{\mathrm{SD}}_{p+1}}$$

For studies reporting mean differences ($∆\underline{X})$ between treatment and control and standard deviation (SD) at follow up (p+1):

$$d=\frac{\Delta {\underline{X}}_{p+1}}{SD_{p+1}}=\frac{{\underline{X}}_{Tp+1}-{\underline{X}}_{Cp+1}}{SD_{p+1}}$$

For studies reporting mean differences between treatment and control, standard error (SE) and sample size (n):

$$d=\frac{{∆\underline{X}}_{p+1}}{\mathrm{SE}\sqrt{n}}$$

For studies reporting regression results, authors intended to follow the approach suggested by Keef & Roberts (2004) and used the regression coefficient and the pooled standard deviation of the outcome. However, in most cases, the pooled standard deviation of the outcome was unavailable, and so regression coefficients and standard errors or t‐statistics were used to do the following, where sample size information was available in each group:

$$d=t\sqrt{\frac{1}{n_{T}}+\frac{1}{n_{C}}}$$

where *n* denotes the sample size of treatment group and control. The following was used where total sample size information (*N*) was available only (as suggested in Polanin, 2016):

$$d=\frac{2t}{\sqrt{N}}\;Var_{d}=\frac{4}{N}+\frac{d^{2}}{4N}$$

The t‐statistic (*t*) was calculated by dividing the regression coefficient by the standard error. If the study authors only reported confidence intervals and no standard error, the review team calculated the standard error from the confidence intervals. If the study did not report the standard error, but reported *t,* this was extracted and used as reported by the authors. In cases in which 1 per cent, 5 per cent and 10 per cent significance levels were reported rather than *t* or *se(b)*, then *t* was imputed approximately, using information about sample size, as follows:

*Prob > 0.1:t=0.5*

*0.1≥ Prob > 0.05:t = 1.58*

*0.05≥ Prob > 0.01:t = 1.96*

*0.01≥ Prob: t = 3.2.*

Where studies reported (log‐) odds ratios, we transformed them into *d* using the following (Higgins and Green, 2011):

$$d=\frac{\sqrt{3}}{\pi }ln(OR).$$

### 5.3.3 Criteria for determination of independent findings and effect sizes

In this review, data are reported according to the intervention that the evidence was based on. The review avoided double‐counting of evidence and synthesis of dependent findings from multiple studies in any single analysis by linking papers prior to analysis. Where studies reported multiple outcome sub‐groups for the same outcome construct (e.g. studies reporting simple, intermediate and complex knowledge), the review calculated “synthetic effects” (sample weighted averages) prior to synthesis. Where studies reported multiple outcomes or evidence according to sub‐groups of participants, data on relevant sub‐groups are reported separately.

Estimation of a standard meta‐analytic effect size relies on the statistical assumption of independence of each included estimation of effect (Gleser & Olkin, 2007). Dependent effect sizes arise when one study provides multiple results for the same outcome of interest, or multiple outcomes for the same outcome construct, when a study has multiple treatment arms compared to the same control group, or multiple studies use the same dataset and report on the same outcome. This review therefore used rules to ensure that only statistically independent effect sizes were included in any one meta‐analysis. In general, this review only included one effect estimate per sample in a single meta‐analysis. Where the review team identified several papers that reported on the same study, effect size data from the most recent publication was extracted. Where studies collected multiple outcomes measuring the same underlying constructs, rather than choosing a particular outcome, a more objective a priori decision rule seemed to be to calculate an average (synthetic effect). Similarly, where the authors had calculated an index comprising outcomes measuring the same construct (e.g. use of health services in Giné, Khalid, & Mansuri, 2018; knowledge of employment services in Ravallion, van de Walle, Dutta, & Murgai, 2013), the review used that estimate. Where different studies reported on the same programme, but used different samples (for example from different regions, or different treatment arms) this review included both estimates, treating them as independent samples, provided effect sizes were measured relative to separate control or comparison groups. This was the case, for example, of the two study arms mandating inclusion of women in a CDC village development council treatment group and a *jirga* (local government) treatment group in Afghanistan (Beath, Christia, & Enikolopov, 2013), where effects were measured against standard practice alternatives (respectively, a control group and a business‐as‐usual *jirga* where women's participation was not mandated).^3^

Where a study reported multiple effect size estimates using different specifications for the same outcome, the review team chose the one with the lower likelihood of bias, for example the most appropriately specified outcomes equation (e.g. covariate adjusted specifications over unadjusted specifications in non‐randomised studies). Where information was collected on the same programme for different periods of time, information on the full range of outcomes over time was extracted. However, the review team calculated an average synthetic effect size for use in any overarching analysis. There was also one case where the findings of an included study (Björkman & Jakob, 2009) were replicated by authors using the same data (Donato & Garcia Mosqueira, 2016). In this case, the review team used critical appraisal to determine which outcomes to include from which study.

### 5.3.4 Unit of analysis issues

Authors assessed studies for unit of analysis errors (The Campbell Collaboration, 2014), arising when the unit of allocation of a study or treatment unit is different to the unit of analysis of outcomes data collection. If unit of analysis errors exist, this was corrected for by calculating the effective sample size (*N*_*e*_) using the following adjustment (Higgins and Green, 2011):

$$\mathrm{Ne}=\frac{N}{1+(m-1)c}$$

where *N* is the total sample size, *m* is the average number of observations per cluster and *c* is the intra‐cluster correlation coefficient, assumed equal to 0.05. Where included studies used robust Huber‐White standard errors to correct for clustering, the review team calculated the standard error of *d* by dividing *d* by the t‐statistic on the coefficient of interest.

Authors suspected several studies to have unit of analysis errors, which were corrected in effect size calculation. These studies were Capuno & Garcia (2010), and certain outcomes within Alhassan, Nketiah‐Amponsah, Spieker, Arhinful, and de Witand Rinke (2016), Bandyopadhyay, Shyamsundar, and Xie (2010), Bradley & Igras (2005), Kasim (2016), Molina (2014), Palladium (2015), Pandey, Sehgal Ashwini, Riboud, Levine, and Goyal (2007), Persha & Meshack (2016), Rasolofoson et al. (2015), Ravaillon (2013) and Touchton & Wampler, (2014).

### 5.3.5 Dealing with missing data

In cases of missing or incomplete data, this review reported the characteristics of the study but stated that it could not be included in the analysis due to missing data. Data were missing or incomplete for some of the outcomes in one study (Palladium, 2015). Hence the review was unable to calculate effect sizes for perception related outcomes (feelings of safety, feelings about police responsiveness, feelings about collaboration of public bodies with the community), although effect sizes for reported crime were calculable for that study. The review team did not contact the authors to obtain the missing statistical information because the missing outcomes were not considered sufficiently important in the causal chain (e.g. because they did not relate to wellbeing) and, given the critical appraisal assessment, would not affect the overall conclusions of the review. In other cases, for immediate outcomes on service use, authors did not present counterfactual information hence effect size calculation was not possible (Grossman, Jonathan, Tausanovich, & Han, 2017).

### 5.3.6 Assessment of heterogeneity

This review assessed heterogeneity by calculating the Q‐statistic, I‐squared, and Tau‐squared to provide an estimate of the amount of variability in the distribution of the true effect sizes (Borenstein et al., 2009). This was complemented with assessment of heterogeneity of effect sizes graphically using forest plots. The review explored heterogeneity using moderator analysis to correlate intervention characteristics with outcomes using bivariate meta‐analysis rather than meta‐regression.

### 5.3.7 Assessment of reporting biases

This review attempted to reduce publication bias by searching for and including unpublished studies in the review. Tests for the presence of publication bias are presented through the use of contour‐enhanced funnel graphs (Peters, Sutton, Jones, Abrams, & Rushton, 2008) and statistical tests (Egger, Davey Smith, Schneider, & Minder, 1997). As these tests may be sensitive to effect size dependency, the review used study‐level synthetic effects in these analyses, to ensure only independent observations were included.

### 5.4 Data synthesis

#### 5.4.1 Methods of synthesis: review questions 1‐3

Once all included studies were identified, the review team conducted a mapping exercise, which grouped studies under intervention, main PITA mechanism, sector and outcome measure. The inclusion criteria for the review were broad, and so the mapping was used to determine appropriate categories across which to synthesise. The minimum criteria for meta‐analysis is usually to combine studies using meta‐analysis when two or more effect sizes using a similar outcome construct are identified and where the comparison group state is judged to be similar across the two, similar to the approach taken by Wilson et al. (2011).

The review conducted separate analyses by primary outcome (*Review Question 1*):

- service delivery and access (quantity and quality)
- service use
- attitudes to services
- wellbeing outcomes
- state‐society relations.

The review also analysed the intervention mechanisms by analysing secondary outcomes by intervention type (*Review Question 2*):

- service user and citizen engagement (demand‐side behaviours)
- service provider and public servant response (supply‐side behaviours).

Finally, the review explored heterogeneity in effects by intervention type, as well as global region and effects for particular sub‐groups of participants (*Review Question 3*).

As heterogeneity exists in theory due to the variety of interventions and contexts included, this review used inverse‐variance weighted, random effects meta‐analytic models (Higgins & Green, 2011). The review team used Stata's *metan* command (Sterne et al., 2008) to generate the meta‐analyses and forest plots. Sensitivity analysis was undertaken by reporting findings by study design and risk of bias assessment.

#### 5.4.2 Methods of synthesis: review question 4

In the context of “real world” programmes, project design and implementation fidelity are often the principal reasons why findings from programme evaluations differ between contexts. This is partly why advocates of mixed‐methods evaluation approaches recommend collecting implementation process data (e.g. White, 2009; Bamberger et al., 2010). This review used a realist‐informed framework synthesis approach to extract information from project design and implementation documents and included impact studies on context, implementation and mechanisms.

Framework synthesis starts with the identification or development of a framework to guide the analysis that highlights key factors that help understand or predict heterogeneity across results, which is built out through in‐depth reading of included studies to include additional relevant themes against which studies are coded and reviewed to identify patterns (Oliver et al., 2008). Framework synthesis is well‐placed to handle complexity across interventions and contexts and is amenable to the use of a wide range of potential sources of data, including evidence based on surveys and quantitative data, and more detailed evidence collected using qualitative methods, policies and implementation documents (such as proposals or monitoring reports) (Snilstveit, 2012).

Realist synthesis highlights variation in programme design in explaining differences in outcomes across contexts (Pawson, 2006). Realists argue that the effectiveness of a programme depends on the combined action of the behavioural mechanisms underlying it and the context in which it takes place. Behavioural mechanisms operate through the values, beliefs and past experiences of individuals in the social system. Thus, factors such as interpersonal networks and individual agency are important in the adoption and rejection of an intervention. The action of mechanisms depends in part on the context in which they are used. Behaviour change is achieved via the entire system of social relationships (the context) and, therefore, an intervention geared towards the achievement of behaviour change must be aligned with the context in which it is used. The approach that draws these concepts together is called context‐mechanism‐outcome (CMO) synthesis. There are different ways of conducting the synthesis including iterations of a causal model such as a theory of change diagram (e.g. Waddington et al., 2014; Carr‐Hill et al., 2016), tables presenting context mechanisms and outcomes for each intervention included in a review (e.g. Petrosino, Morgan, Fronius, Tanner‐Smith, & Boruch, 2012), and qualitative comparative analysis (QCA) (e.g. Ton et al., 2017).

Van der Knapp et al. (2006) is possibly the first example of a systematic review that explicitly incorporates context‐mechanism‐outcome synthesis. These authors undertook the CMO synthesis after the systematic review and meta‐analysis. The broad approach was to collect information on possible programme mechanisms and contextual factors from studies during the coding phase. They stated that “The focus in such a classification can be on behavioral and social “cogs and wheels” of the intervention… but could also include administrative or legal mechanisms.” (p.6).

In the present study, the review team searched included studies for information about how or why the intervention is supposed to work from descriptive information provided in the studies, author analysis (e.g. tests for “mechanisms” using statistical mediator analysis) and authors’ own hypotheses about why the intervention was effective (or not). The information collected on contextual factors was partly contained in the detailed information about the comparison condition, co‐interventions and background information about participants collected from included studies and project and programme design and implementation documents, and key contextual information collected from international datasets. As noted in more detail below, this review then identified and coded mechanisms associated with particular intervention sub‐groups and PITA elements.

CMO is largely an iterative process, and thus the full list of CMO codes for analysis was developed as part of the synthesis. Initially, the review team drew on potential codes identified in the protocol, including contextual conditions and enabling conditions, including: systemic and social levels targeted by the intervention; whether the intervention is designed to build off of and work within local systems of power relations and social norms that uphold the social contract between the State and society (as in Halloran's “accountability ecosystem” (2015); the political salience of the public service targeted (Mcloughlin and Batley, 2012); or the relative power of proponents versus opponents in the adoption phase of the policy cycle (Resnick, Babu, Haggblade, Hendriks, & Mather, 2015).

Where key enabling conditions are already in place, an intervention effectively designed may be successfully implemented in isolation; where key conditions are missing, the intervention design may need to be adjusted or expanded to include complementary interventions that seek to strengthen the enabling environment. For example, an intervention seeking to build transparency and accountability through open data interventions may need to build a coalition of support that engages people at the point in the system targeted for data release, upstream, downstream, and externally to create an environment in which data are provided, demanded, and used (Hogge, 2010). These enabling conditions may change depending on context factors such as the target level of the intervention – whether it targets service delivery at community, sub‐national, or national level (E‐Pact Consortium, 2016) or whether the external stakeholders it seeks to engage are organised civil society or interest groups, marginalised or vulnerable groups, or citizens and service users more broadly (McGee and Gaventa, 2010). The review team further conducted more detailed analysis of whether the bottleneck for good governance was likely to be properly identified as resting with citizens (e.g. lack of organization, lack of knowledge/ capacity), with the system (e.g. lack of opportunities for citizens to engage), or with individual service providers (e.g. power relations, corruption).

The combination of realist‐informed framework synthesis that moved towards “best fit” framework synthesis was selected as the most appropriate method to link the meta‐analysis with context and mechanism information given the complexity and heterogeneity of included interventions.

In the analysis, the theory of change developed during the protocol as the overarching framework was built out into a template to include the series of additional potential explanatory factors identified in the protocol regarding the enabling conditions that allow for project success, and systemic and social levels targeted by the intervention. Data from the studies was then extracted along the framework, including coding that identified the source of the data to maintain clarity between first, second and third order constructs. Each extracted data was coded as being sourced from: observations from implementers; insight reported by participants (i.e. quotes, first order constructs); survey by researcher; commentary by researcher (i.e. researcher interpretation of results, second order constructs); or commentary by authorauthor (i.e. interpretation based on insights from synthesis, third order constructs). The goal of framework synthesis is to draw conclusions that explain relationships between study findings, with a focus on explaining heterogeneity of results due to variations in context, intervention design and implementation quality (Snilstveit, 2012). The review focused on extracting data that enabled the identification of mechanisms, moderators, and other explanatory factors along the causal chain.

Following the extraction and analysis of data across the framework, interventions were organised according to broad intervention group and key PITA mechanism. Critical case comparisons were identified to evidence the role of moderators in triggering different mechanisms under different contexts. Moving towards “best fit” framework synthesis, which is more iterative and focused on building programme theories (Carroll et al., 2013), we analysed the emerging patterns of moderators and mechanisms within each set of interventions to identify those that most frequently or persuasively facilitated sense‐making of the results of each study. These insights were used to create composite frameworks for each group of interventions that refine the initial framework based on the findings from the qualitative synthesis. Thus, this review more precisely highlights intervention‐specific mechanisms and moderators influencing movement along the causal chain.

##### Moderator analysis

The following moderator variables were collected, as indicated in the protocol:

- Methodology: study design, risk of bias status, timing of evaluation (follow‐up length).
- Intervention characteristics: intervention, PITA characteristic, sector.
- Context variables: region, country income level, democracy policy index score.
- Participant characteristics: e.g. sex, socio‐economic status.

#### 5.4.3 Methods of synthesis: review question 5

This review aimed to draw on standard approaches to synthesise economic appraisal evidence (Shemilt et al., 2008; Shemilt et al., 2011). However, only four of the included studies reported cost data, and therefore this review simply reports the cost data that was identified in a table in the results section, making adjustments for estimated number of participants and real prices (2016 US$) using data provided on numbers of participants and the CCEMG‐EPPI Centre Cost Converter (v. 1.5 last update: 29 April 2016).

### 6 RESULTS OF SEARCH AND CRITICAL APPRAISAL

This section summarises the results of the search, presents descriptive information about the included studies, and discusses findings from the critical appraisal.

#### 6.1 Results of the search

**Figure 3:**
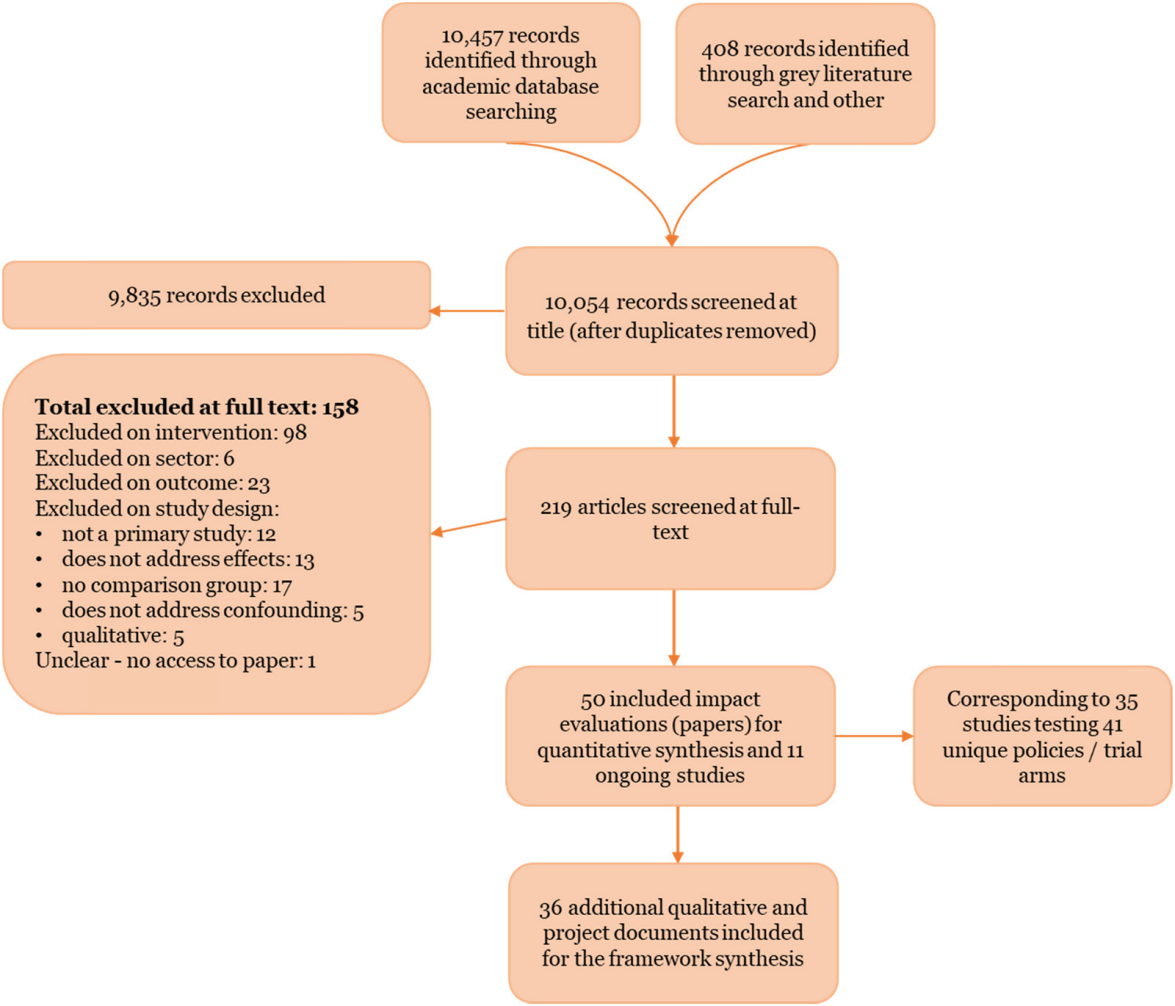
Study search flow diagram

The results of the search and screening process are shown in the study flow search diagram in Figure 3. The initial academic search resulted in 10,457 hits while the searches of grey literature resulted in 408 relevant hits (see Appendix 2 for the search record). In addition, the review took the relevant included studies from the state‐society relations evidence gap map (Phillips et al., 2017), which added an additional 348 studies, leaving a total of 10,865.

Following the removal of duplicates, 10,054 studies were left to screen at title and abstract. As described in the methods section in more detail, this review used text mining in EPPI‐Reviewer 4 to reduce the initial screening workload. Authors first independently double screened approximately 10 per cent of the search results, then used the priority screening function to develop a classifier that classified studies into groups based on their probability of inclusion in the review, using data from the 10 per cent of screened studies. The review team decided to automatically exclude studies with less than a 20 per cent probability of inclusion, corresponding to 7,241 of the search hits. Authors screened a random 10 per cent of the 0‐9 per cent and 10‐19 per cent group to check the quality of the classifier but identified no studies that had been wrongly excluded. Authors double screened all studies classified as 20‐99 per cent probability of inclusion. In total, 9,835 were excluded at title and abstract.

This left 219 papers to screen at full‐text. After independent double screening, sometimes involving a third reader, 57 impact evaluations were included in the first stage. Authors undertook forward and backward citation tracking on this initial set of studies to identify studies missed by the initial search, identifying an additional 10 papers. After detailed reading of the complete set, 17 further studies were excluded on intervention or outcome. In the end, 50 eligible papers corresponding to 35 unique studies were identified. These 35 studies assess the effect of 41 unique policies or trial arms. The systematic search also identified 11 ongoing studies, a list of which is presented in **references to ongoing studies**. Reasons for exclusion are discussed in more detail below. Overall, of the 35 unique studies included in this review, 16 had been included in the state‐society relations EGM, three of which as ongoing studies with registered trials (Phillips et al., 2017).

Following the search for impact evaluations, authors undertook a targeted search for qualitative and project documents associated with the programmes evaluated in the included impact evaluations. In total, 76 additional documents were identified, of which 36 contributed to the qualitative synthesis. These are discussed in more depth in the section on framework synthesis.

#### 6.1.1 Excluded studies

Studies were often excludable for more than one reason, but we did not search for all possible reasons for exclusion once a study met one exclusion criteria. We excluded 98 papers at full‐text for not meeting our criteria on intervention. With regards to those excluded on intervention, we excluded five as they were classified as informal sector, that is, the programme was implemented independently of government. We excluded six as they only addressed service access for marginalised populations through the delivery of a new service. We excluded 24 as they were unable to isolate the PITA element of the intervention, that is, the evaluation measured the effect of a PITA mechanism packaged with other interventions. We excluded a further 63 papers for evaluating other irrelevant interventions. One of these studies excluded on intervention was of an ineligible “recentralisation” intervention which acted to reduce the level of citizen participation (Malesky et al., 2014).

We excluded an additional six because they evaluated a study of education or a participatory planning intervention alongside a block grant (CDD), 12 because they were not a primary study, 13 because the study did not address questions of effects, five because they were qualitative, five because they did not account for confounding in design or analysis, and 17 for not using a contemporaneous comparison group (e.g. before versus after design). In addition, we were unable to access one paper.

A further seven studies were eligible for being included based on population, intervention and comparison but only examined the effects of a PITA mechanism on one or more secondary outcomes of interest, that is, citizen engagement and/or provider response, without extending the analysis to primary outcomes of interest. These studies were Casey et al. (2018), Finkel (2012), Gottlieb (2016), Grossman et al. (2014), Grossman et al. (2016), Sexton (2017), Sheely (2015) and Yanez‐Pagans and Machicado‐Salas (2014).

After the full‐text screening stage, we excluded a further two papers that appeared to be evaluations of eligible interventions, but that we discovered to be PITA mechanisms implemented alongside co‐interventions that were not reported clearly in the original evaluation (Alderwish & Dottridge, 2013; Andres et al., 2017). We discovered the presence of the additional co‐interventions in the additional documentation we identified through our targeted searches. Both papers evaluated community driven water provision. For Andres et al. (2017), we identified a 2009 World Bank Implementation Completion and Results Report associated with the project evaluated in the paper, the *Jalanidhi* project. The report described co‐interventions that would likely have impacted the outcomes covered by the evaluation, including significant technical engineering assistance, infrastructure, and capacity building. The impact evaluation does not acknowledge these co‐interventions, but rather presents the study as isolating the impact of the institutional form the water management system takes on the outcomes. Thus, due to the co‐interventions, the study did not isolate the effect of the PITA mechanism and was excluded from the review. Alderwish & Dottridge (2013) was a similar case in that a project document identified significant infrastructure interventions combined with the community water provision intervention.

#### 6.1.2 Studies awaiting classification

We identified one eligible study towards the end of the review process that we were unable to include due to time constraints, Tohari, Parsons, and Rammohan (2017). It is unlikely that the inclusion of this study would substantively change the results of our synthesis, partly as the study evaluates an intervention already included in the review (Banerjee et al., 2018). The results of that study should be included in updates of this review.

#### 6.2 Description of included studies

Here we describe the characteristics of the 35 included studies. Key characteristics of each included study are presented in Appendix 4.

#### 6.2.1 Setting

**Figure 4:**
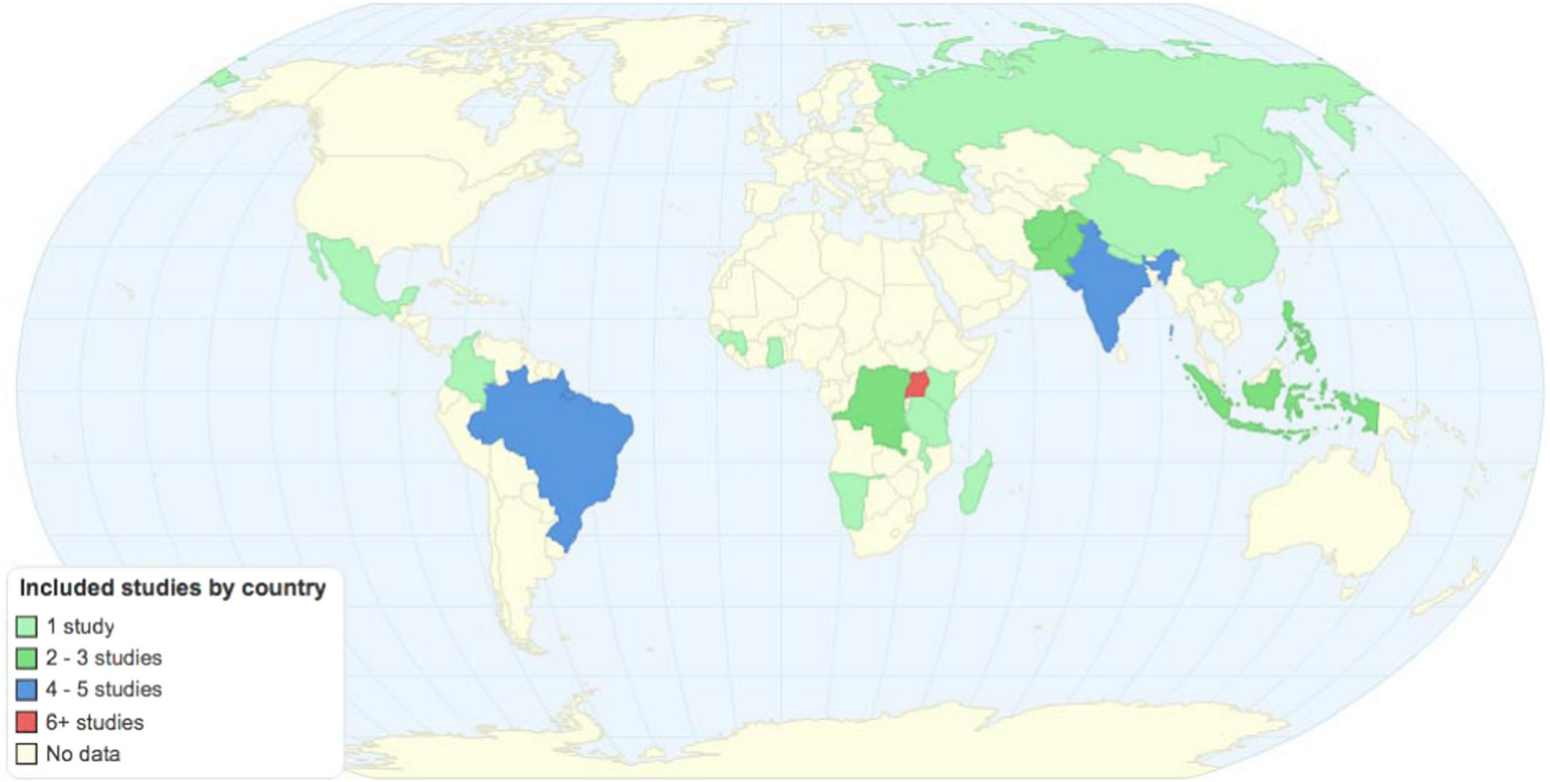
Geographical distribution of included impact evaluations

Figure 4 shows the geographical spread of the included studies. The most studied area for included interventions is Sub‐Saharan Africa (n = 13), representing almost 40 per cent of the included studies. We included five studies of interventions that took place in Uganda (Björkman & Jakob, 2009; Björkman Nyqvist, de Walque, & Svensson, 2017; incorporating Donato & Garcia Mosqueira, 2016; Fiala & Premand, 2017; Grossman & Michelitch, 2018; Grossman et al., 2017; Humphreys & Weinstein, 2012), two from the Democratic Republic of Congo (DRC) (Humphreys, Sanchez de la Sierra, & van der Windt, 2014; Palladium, 2015), one each respectively from Ghana (Alhassan, Nketiah‐Amponsah, Spieker, Arhinful, & Rinke de wit, 2016), Tanzania (Persha & Meshack, 2016), Madagascar (Rasolofoson et al., 2015), Malawi (Gullo et al., 2017), Namibia (Bandyopadhyay, Humavindu, Shyamsundar, & Wang, 2004) and a study that took place in both Kenya and Guinea (Bradley & Igras, 2005).

We identified nine studies from South Asia, four of which took place in India (Ananthpur, Malik, & Rao, 2014 in Karnataka; Banerjee et al., 2014 in Rajasthan; Pandey et al., 2007 in Uttar Pradesh; Ravallion et al., 2013 in Bihar). The remaining studies took place in Afghanistan (Beath et al., 2013; Berman et al., 2017), Pakistan (Giné et al. 2018; Kasim, 2016) and Nepal (Tachibana & Adhikari, 2009). In addition, we identified five studies from East Asia and Pacific, including two from Indonesia (Banerjee et al., 2018; Olken, 2007), two from the Philippines (Bandyopadhyay et al., 2010; Capuno & Garcia, 2010) and one from China (Huang, 2014).

We included six studies from Latin America, of which four were from Brazil (Gonclaves, 2013; Barde, 2017; Timmons & Garfias, 2015; Touchton & Wampler, 2014), and one each from Colombia (Molina, 2014) and Mexico (Diaz‐Cayeros, Magaloni, & Ruiz‐Euler, 2014).

Finally, we identified one study in Russia, a study of support for participatory budgeting (Beuermann & Amelina, 2014).

#### 6.2.2 Interventions and PITA mechanisms

We grouped the identified studies by five main intervention areas, presented in Table 4. Eleven studies provided information to citizens, either about citizen rights to access services or to participate in participatory processes (n = 5), or information about performance of politicians or public service providers, including report cards (n=7). We consider the main design mechanism for these categories to be transparency, either for increasing citizen participation or transparency to improve accountability. The majority of the studies providing performance information provided information about politicians or local governments, for example Humphreys & Weinstein's (2012) evaluation of the dissemination of a scorecard with detailed information on the performance of Ugandan Members of Parliament (MPs). We included these studies in the review as in all cases we would expect these interventions to have an impact on service delivery in politician's local areas as well as potentially having an impact on voting intentions of citizens. We also included Banerjee et al.'s (2014) RCT in this category, that placed two volunteers from the local community in police stations, as the objective of the study was for the volunteers to feed back their observations to the community rather than for them to give feedback to the police.

**Table 4:** Included interventions and associated PITA mechanisms

*Intervention type*	*Intervention definition*	*PITA Mechanism(s)*	*Included studies*
Rights information provision	Provides information about citizen rights to access services or rights to participate in participatory processes	Transparency for improved participation	• Olken (2007) – Indonesia, invitations only intervention group
			• Kassim (2016) – Pakistan
			• Ravallion et al. (2013) – Bihar, India
			• Banerjee, Hanna, Kyle, Olken, and Sumarto (2016) – Indonesia
			• Pandey et al. (2007) – India
Performance information provision	Provides citizens with information about performance of politicians or public service providers, including report cards	Transparency for improved accountability	• Humphreys & Weinstein (2012) – Uganda
			• Grossman & Michelitch (2018) – Uganda
			• Timmons & Garfias (2015) – Brazil
			• Capuno & Garcia (2010) – Philippines
			• Banerjee et al. (2014) – Rajasthan, India
			• Fiala & Premand (2017) – Uganda, scorecard only group
Citizen feedback and monitoring	Interventions to allow citizens to feedback concerns or priorities around service delivery to providers, and / or to monitor the delivery of public service delivery. This includes community scorecards and social audits.	Accountability	• Olken (2007) – Indonesia, invitations + feedback group
			• Berman et al. (2017) – Afghanistan
			• Alhassan et al. (2016) – Ghana
			• Grossman et al. (2017) – Uganda
			• Björkman et al. (2009; 2017; incorporating Donato & Garcia, 2016) – Uganda
			• Bradley & Igras (2005) – Kenya & Guinea
			• Palladium (2015) – DR Congo
			• Gullo et al. (2017) – Malawi
			• Fiala & Premand (2017) – Uganda
			• Molina (2014) – Colombia
Participatory planning	Interventions to introduce or facilitate public participation in public institutions' decision‐making processes, priority setting or budget allocation decisions, including participatory budgeting	Participation (+ inclusive planning*)	• Touchton & Wampler (2014) – Brazil
			• Gonclaves (2013) – Brazil
* inclusive planning	* Interventions that mandate the participation of the whole community or marginalised groups into planning processes		• Diaz‐Cayeros et al. (2014) – Mexico
			• Beuermann & Amelina (2014) – Russia
			• Ananthpur et al. (2014) – Karnataka, India
			• Giné et al. (2018) – Pakistan
			• Humphreys et al. (2014) – DR Congo*
			• Beath et al. (2013) – Afghanistan*
Community‐based natural resource management (CBNRM) committees	Devolution of some part of the management of a natural resource to a community group while the government retains some powers. This includes Water User Associations (WUAs) and Community‐Based Forest Management (CBFM) organisations	Participation	• Bandyopadhyay et al., (2004) – Namibia
			• Bandyopadhyay et al., (2010) – Philippines
			• Persha & Meshack. (2016) – Tanzania
			• Rasolofoson et al., (2015) – Madagascar
			• Tachibana & Adhikari (2009) – Nepal
			• Barde (2017) – Brazil
			• Huang (2014) – China

The intervention area with the greatest number of included studies is citizen feedback and monitoring mechanisms, where we identified 10 studies or treatment arms. This set includes evaluations of interventions to allow citizens to feedback concerns or priorities around service delivery to providers, and/or to introduce or facilitate monitoring of public service delivery. We consider the main design mechanism here to be accountability as it encourages or actively hold individuals, public service providers and institutions responsible for executing their powers and mandates according to a certain standard. Within this category, interventions largely fell into two groups, those with facilitated citizen feedback and those with unfacilitated citizen feedback. Facilitated citizen feedback covers interventions that solicited concerns from citizens through community meetings or focus groups in order to feed back to service providers, often using a local facilitator or civil society organisation, for example (Björkman & Jakob, 2009; Björkman Nyqvist et al. 2017) and Ananthpur et al. (2016). Unfacilitated feedback interventions gave citizens the tools or opportunities to give feedback or monitor but the collection of these concerns is not through a facilitated group meeting, for example Fiala & Premand (2017) which trains communities to monitor community CDD projects, as well as identify and make complaints about corruption and mismanagement, but does not set up forums to do so. We only identified one study that used technology to solicit feedback on service provision, namely Grossman et al.'s (2017) study of the U‐Bridge programme in Uganda that introduced a SMS‐based service for citizens and local government officials to submit, monitor and respond to requests around public service delivery.

Seven studies evaluated a participatory planning mechanism to introduce or facilitate public participation in public institutions' decision‐making processes, such as participatory budgeting. Two of these studies were different in that they introduced support for existing participatory planning mechanisms, namely Beuermann & Amelina (2014) that introduced training and technical assistance for an existing participatory budgeting system in Russia, and Ananthpur et al. (2014) which evaluated a citizenship engagement programme to encourage participation, and support, the existing ward *sabha* system in India. The other five studies compared the participatory planning mechanism to an area where the mechanism did not exist.

We identified a further two studies that evaluated mandating the participation of women into decision‐making processes around service delivery, both in the context of community driven development (CDD) programmes. These are Humphreys et al. (2014) evaluation of Tuungane in the DRC and Beath et al.'s (2013) evaluation of the NSP in Afghanistan. It should be noted that we did not include the findings from these studies that evaluate the impact of the CDD programmes themselves which was outside the scope of this review, only the comparison between those groups that mandated participation of women and those that did not. We consider these sub‐sets of the participatory planning intervention category.

Finally, we identified seven studies evaluating community management of natural resources, whereby there is some devolution of the management of a natural resource to a community group, but where the government retains some powers. These fell into two groups; those that involved management of water (Bandyopadhyay et al., 2010; Barde, 2017; Huang, 2014) and of forests or conservancies (Persha & Meshack, 2016; Rasolofoson et al., 2015;

Bandyopadhyay et al., 2004; Tachibana & Adhikari, 2009). This intervention category differs substantively from the others in that communities are equipped with considerable more power to make decisions and implement public services than the other intervention areas.

#### 6.2.3 Intervention funders

**Figure 5:**
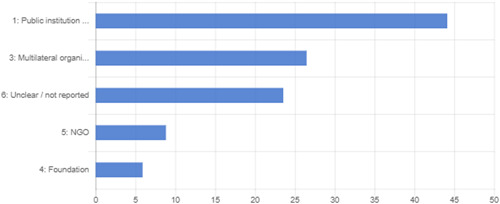
intervention funding sources

We attempted to capture information on the funders of the programmes or policies evaluated in the included impact evaluation, shown in Figure 5. Almost 45 per cent of the programmes received funding from a public institution such as a national government, university or bilateral donor such as the Department for International Development or USAID. Twenty‐five per cent received funding from a multilateral institution, all of which received funding from a department from within the World Bank, in some cases combined with funding from another multilateral institution. Three of the interventions were at least partly funded by an NGO and two by a foundation. In almost 25 per cent of the studies, the intervention funders were not reported.

#### 6.2.4 Equity

For each study, we captured information about if, and how, it addresses equity concerns, either through the design of the intervention or through the evaluation design and analysis methods. We considered an intervention to address equity if it targeted a marginalised or vulnerable group or was designed in a way to overcome local barriers to incorporate these groups into the programme. We considered an evaluation design and analysis method to incorporate equity if it undertook sub‐group analysis for the marginalised group or reported on how those groups were able to participate in the programme.

Eighteen of the included studies did not explicitly address equity concerns.^4^ Nine of the included studies evaluated an intervention that addressed equity concerns by design. Two of these studies focused exclusively on how the mandated incorporation of women into community groups affected service delivery outcomes. These were Humphreys et al.'s (2014) evaluation of how removing the gender parity component of the CDD programme, Tuungane, in the DRC affected outcomes, and Beath et al.'s (2013) evaluation of how the requiring female participation in the distribution of food aid in the context of a CDD programme in Afghanistan, the NSP, and through the traditional *jirga* system, affected delivery and corruption. Two of the citizen feedback studies, (Björkman & Jakob, 2009; Björkman Nyqvist et al. 2017) in Uganda and Gullo et al. (2017) in Malawi, divided citizens into key social groups such as women, men, youths in order to get their perspectives over issues concerning service delivery and determine their preferences for change. The Diaz‐Cayeros et al. (2014) evaluation in Oaxaca, Mexico assessed the *Usos y Costumbres* system, which formalises participation of traditional forms of governance, typically of indigenous groups, in municipality level government decision‐making. The participatory budgeting system in Brazil, evaluated in Touchton & Wampler (2014) and Gonclaves (2013), frequently adopts a “quality of life index”, which allocates greater resources on a per capita basis to poorer neighbourhoods. Banerjee et al. (2018) evaluates an information campaign on the Raskin rice for poor households programme in Indonesia, which is targeted at poor households who are entitled to the rice but do not receive it. Finally, Giné et al.'s (2018) evaluation of the initial community mobilisation stages of a CDD programme in Pakistan actively targets the inclusion of women and poor households in the mobilisation and community organisation formation process.

Only eight of the included studies addressed equity issues by evaluation design.Just six of the included studies undertook sub‐group analysis by a marginalised or vulnerable group. Palladium's (2015) evaluation of the community engagement component of the Security Sector Accountability and Police Reform (SSAPR) Programme in the DRC undertook sub‐group analysis by men and women for outcomes around crime and feelings of security in the community.Ananthpur et al. (2014) undertook a sub‐group analysis for the effect of the “People's Campaign” in Karnataka, India, on female and male agricultural wages.Ravallion et al.'s (2013) evaluation of an information campaign around NREGA (National Rural Employment Guarantee Act) assessed outcomes on service use and knowledge of rights for men and women separately. Bandyopadhyay et al. (2010) assessed the effects of the Irrigation management transfer (IMT) to Irrigation Associations in the Philippines on production of rice for both the asset rich and the asset poor. Persha & Meshack (2016) assessed how the Joint Forest Management policy in Tanzania affected women headed households. Finally, Pandey et al.'s (2007) evaluation of a rights campaign in India undertook sub‐group analysis by people belonging to lower and mid‐ to high‐level castes.

In addition to sub‐group analysis, Ananthpur et al. (2014) also included a substantial ethnographic component, which considered the participation of particularly marginalised groups in the gram sabha system in India following the information campaign and considered how women had been mobilised by the intervention. Alhassan et al.'s (2016) evaluation of a citizen feedback mechanism in Ghana considered the gender dynamics of focus groups that were part of the intervention to identify gaps in service delivery in healthcare facilities. Finally, Diaz‐Cayeros et al. (2014) considered how women's participation related to the Usos y Costumbres system in Mexico, including the share of the municipal council made up by women, and whether the current mayor is a woman.

#### 6.2.5 Types of studies

**Figure 6:**
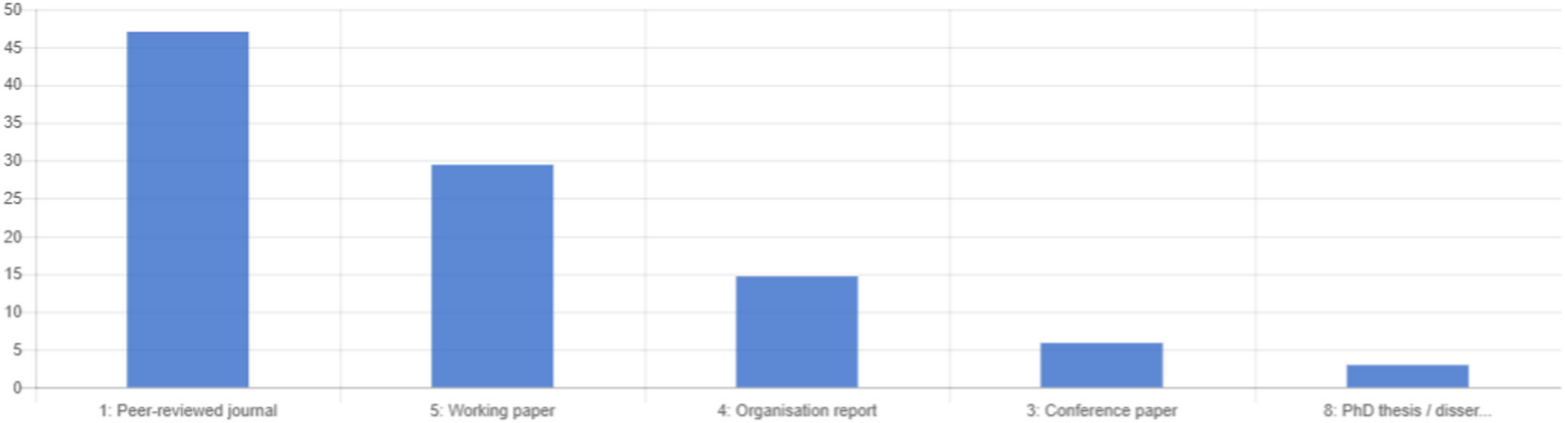
Type of publications

Figure 6 shows the types of publications we included in the review. Just under 50 per cent were peer‐reviewed journal articles. Almost 30 per cent were articles published in working paper series such as the World Bank Policy Research working paper series or Inter‐American Development Bank Paper Series. We identified five organisational reports, for example reports published in the 3ie impact evaluation series or USAID. Finally, we included two conference papers and one PhD thesis (Kasim, 2016).

Nineteen, or just over half, of the included studies were cluster RCTs, that randomised the allocation to the intervention or comparison group at the level of the public service, village, wider community or similar. Most of these studies used covariate‐adjusted regression (n = 15), including fixed effects regression, methods of analysis.Six of these studies used difference‐in‐differences (DID) analysis with baseline data from the RCT. Alhassan et al. (2016) also used Propensity Score Matching (PSM) to analyse some outcomes for their RCT, presumably as there are imbalances between the treatment and control groups.

Timmons & Garfias’ (2015) study from Brazil was the only included natural experiment. It evaluated a policy in Brazil that randomly audited sub‐national government expenditure, the results of which were then published for citizens.

The remaining 14 studies used non‐randomised, quasi‐experimental designs. Ten of the studies used a comparison group with both pre‐intervention and post‐intervention data. Three of these used pseudo‐panel with repeated measurement for groups but different individuals (Capuno & Garcia, 2010; Palladium, 2015; Persha & Meshack 2016). The remaining seven used panel data on the same individuals, households or communities (Diaz‐Cayeroset al., 2014; Touchton & Wampler, 2014; Gonclaves, 2013; Barde, 2017; Bradley & Igras, 2005; Huang, 2014; Tachibana & Adhikari, 2009, ). Six of these studies combined statistical matching with DID analysis, typically through covariate adjusted regression. The remaining studies only used covariate matching followed by a comparison of means or only covariate adjusted regression.

Finally, four of the included studies used a comparison group but only had one data point after the intervention had started. Three of these studies used statistical matching methods (Bandyopadhyay et al. 2010; Molina, 2014; Rasolofoson et al. 2015) combined with another analysis method such as covariate adjusted regression or simple comparison of means, while Bandyopadhyay et al. (2004) used instrumental variables regression only without statistical matching.

Table 5 presents the follow up period for the assessment of outcomes after the start of the citizen engagement intervention. Most of the included impact evaluations assessed outcomes between one year and five years after the start of the intervention (n= 22). In six studies, the follow period was 12 months or less. The shortest follow up period was in Kasim (2016) which looked at outcomes after six months. Two studies assessed outcomes between five and 10 years after the start of the intervention. Six of the evaluations assessed outcomes 10 years or more after the initiation of the intervention. All these evaluations assessed long‐standing national programmes: participatory budgeting in Brazil (Gonclaves, 2013; Touchton & Wampler, 2014), the *Usos y Costumbres* system in Mexico (Diaz‐Cayeros et al., 2014), rural water user associations in Brazil (Barde, 2017) and community‐based forest management in Tanzania (Persha & Meshack, 2016) and Nepal (Tachibana & Adhikari, 2009).

**Table 5:** Follow up periods of included studies

Follow up period	Study
**12 months or less**	Björkman and Svensson (2010), Olken (2007), Fiala & Premand (2017) scorecard intervention only, Kasim (2016), Pandey et al. (2007) Ravallion et al. (2013).
**1 ‐ 5 years**	Alhassan et al. (2016), Björkman Nyqvist et al. (2017), Grossman et al. (2017), Gullo et al. (2017), Palladium (2015), Berman et al. (2017 ‐ two follow ups), Fiala & Premand (2017 ‐ two follow ups), Ananthpur et al. (2014), Beuermann & Amelina (2014), Giné et al. (2018), Bandyopadhyay et al. (2010), Bradley & Igras (2005), Bandyopadhyay et al. (2004), Capuno & Garcia (2010), Humphreys & Weinstein (2012), Grossman & Michelitch (2018), Timmons & Garfias (2015), Banerjee et al. (2014), Banerjee et al. (2018), Humphreys et al. (2014), Beath et al. (2013).
**5 ‐ 10 years**	Huang (2014), Tachibana & Adhikari (2009 ‐ environmental outcomes).
**10 + years**	Diaz‐Cayeros et al. (2014 ‐ several follow up periods), Touchton & Wampler (2014), Gonclaves (2013), Barde (2017), Persha & Meshack (2016), Tachibana & Adhikari (2009 ‐ forest condition).
**Unclear**	Molina (2014), Rasolofoson et al. (2015).

#### 6.3 Critical appraisal of included studies

**Figure 7:**
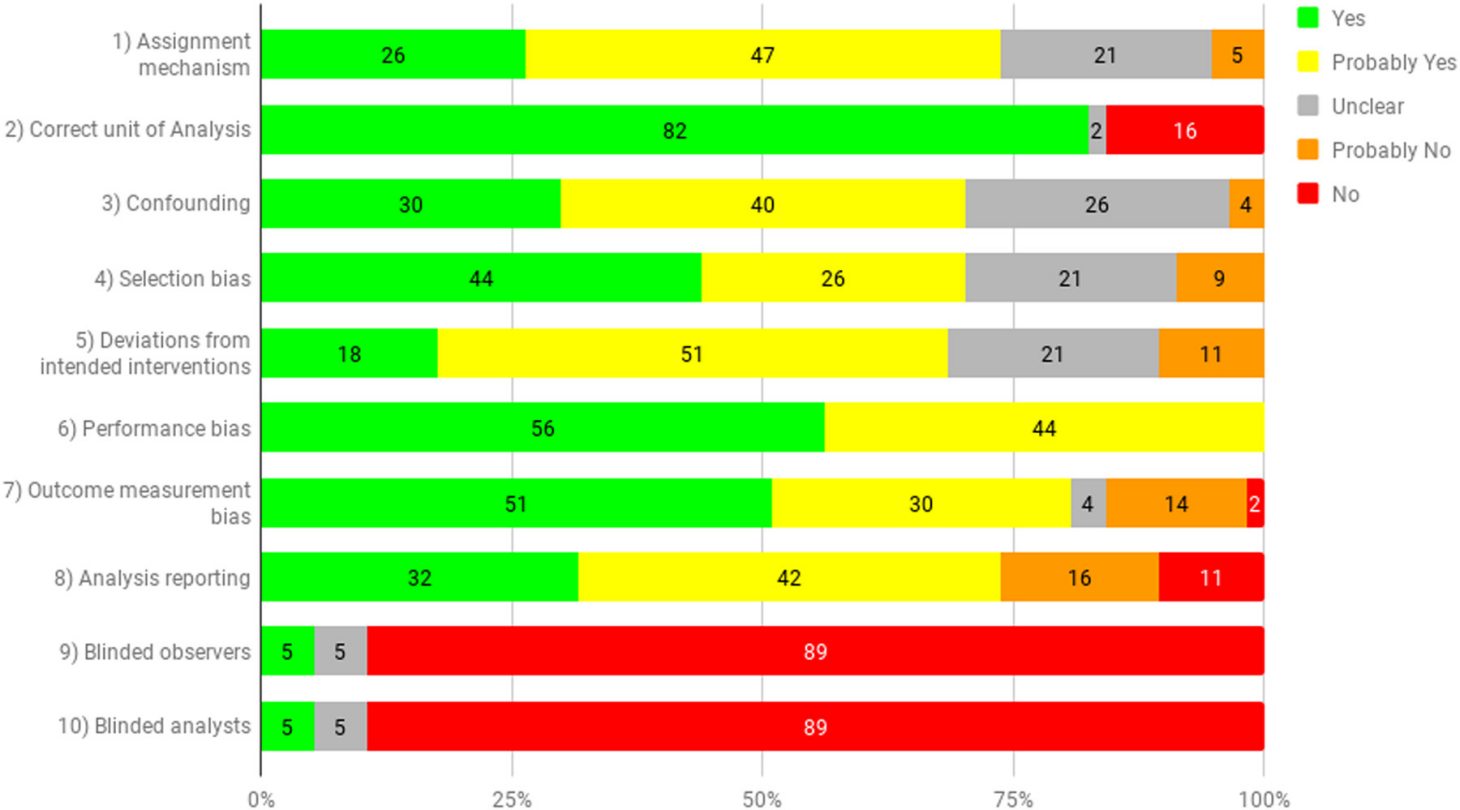
**Summary of risk of bias appraisal for randomised studies** Note: figures are rounded percentages hence may not add to 100 per cent

**Figure 8:**
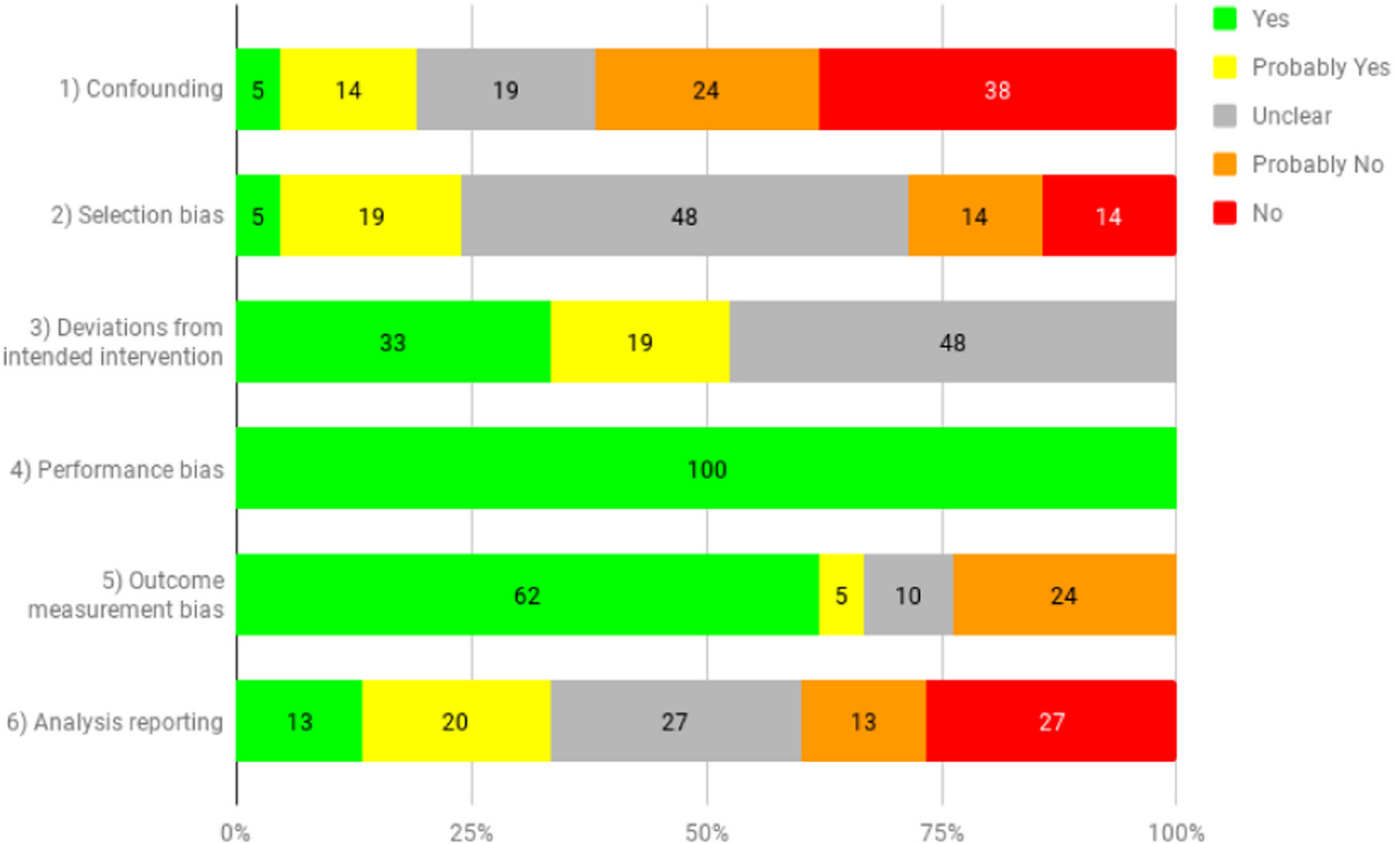
**Summary risk of bias appraisal of non‐randomised studies** Note: figures are rounded percentages hence may not add to 100 per cent

We assessed the risk of bias for all studies included in this review. Figure 7 presents the results for each criteria across all randomised studies and Figure 8 presents the results for non‐randomised studies. The criteria related to the assignment mechanism, analysis reporting and blinding are assessed at the study level whereas all the other criteria are assessed at the outcome level. While selection bias and risks of confounding are usually assessed at the study level, it can be the case that some outcomes are more exposed to bias than others, depending on the data source or the analysis method (e.g. where outcomes data are collected based on participant self‐reports rather than direct observation in non‐blinded studies)*.*

We found that out of the 166 outcomes assessed separately from non‐randomised studies, 146 had high risk of bias, 19 had some concerns, and one had low risk of bias. Out of 386 outcomes assessed separately for randomised studies, 161 had high risk of bias, 83 had some concerns and 142 had low risk of bias. A detailed and overall assessment by study and group of outcomes is presented in Appendix 5.

#### 6.3.1 Findings by risk of bias domain

##### Assignment mechanism in randomised studies

As Figure 7 illustrates, for a large majority of the studies (73%), the assignment of clusters into the different study arms was random or probably random. For only one study (Kasim, 2016), although the assignment mechanism was reported as random and the sample was relatively large, significant imbalances at baseline suggests that there might have been a problem in the random allocation. While assignment seems to have indeed been random for 73 per cent of randomised studies (and is reported as such), 47 per cent lacked detailed information about the exact randomisation method, such as whether the sequence was generated by a computer or whether a paper‐based lottery was organised. In one study, important information on the number of units of programme implementation within each cluster was missing (Berman et al., 2017).

Reporting of a baseline balance table on cluster characteristics and household characteristics is not done systematically. Five randomised studies did not report any balance table and one reported it only for balance on cluster characteristics, even though the outcomes were measured at the household level. Eight out of 14 non‐randomised studies reported a balance table at baseline and when relevant after statistical matching. In some instances, it was not possible to assess baseline balance. For instance, in Ananthpur et al. (2017), the baseline data were collected after the start of the intervention in some villages, yet the analysis method used the difference‐in‐difference technique. The extent to which this undermines the results will depend on the proportion of observations affected, but the authors did not report the information required to assess the scale of the issue.

##### Selection bias

The randomisation ensures that the risk of selection bias into the study is relatively small. A majority of outcomes measured in randomised studies were considered free or probably free from selection bias (70%). However the sampling method used to collect survey data or differential attrition at the end of the study represent threats for RCTs and non‐randomised studies. Given that tracking survey respondents over long time periods or preventing dropouts can be challenging, attrition is common across almost all studies to a certain extent. It is only a threat to validity if it represents a large proportion of the sample and is systematically larger for some study groups than others (and correlated with outcomes). This might be the case for eight per cent of outcomes and is unclear for 21 per cent of outcomes. Unfortunately, the lack of information reported on the reasons for attrition makes it hard to identify risks of selection bias out of the study. Authors do not tend to make attrition information very accessible. In three studies where attritions rates were particularly high (greater than 20 per cent of the baseline sample), authors do not report attrition rates across different treatment and control groups, or test of the relationship between covariates and treatment status, four neglect to comment on varying sample sizes between the initial sample and the results tables, and two do not provide enough information to calculate attrition.

An example of an unclear case is Giné et al. (2018), in which stunting could not be measured in one of the five districts included in the study, and no information was provided on the proportion of treatment and control communities per district. Excluding an entire geographical area because of the difficulty to collect data, could be selecting out of the study populations sharing similar characteristics, but it is not clear whether there was an equal proportion of treated and control communities which would prevent any bias from undermining the results. There could also be selection bias into the study if the sampling of survey respondents was not representative of the study sample, or too small. There is a risk that this bias exists for Kasim (2016), as the in‐person survey was conducted only in one out of eight districts where the study was implemented.

Thirteen of the non‐randomised studies are quasi‐experimental studies using various econometric techniques to control for selection bias. One of the studies (Timmons and Garfias, 2015) is a natural experiment, which evaluates the impact of a programme happening outside the researcher's control, but the selection process resembles random assignment. More than 70 per cent of non‐randomised studies did not provide enough information on the selection process into the programme to reject the risk of selection bias, or failed to overcome the selection bias that was identified. Non‐randomised studies included in this review typically evaluate programme like community‐based natural resource management reforms, because they imply a long‐term change in the management system which cannot be measured via a trial. The selection process for this type of programme is likely to be either the government's decision based on unknown criteria or through self‐selection of the communities themselves. For outcomes in four studies (24%), where the design was likely to introduce selection bias, authors conducted an in depth analysis of the selection criteria and convincingly argue that all characteristics that might affect outcomes were controlled for in the analysis. For these outcomes, the presence of unobservable characteristics that might affect the outcomes is unlikely, therefore these outcomes were rated as probably free from selection bias.

##### Deviations from intended interventions

Any spill overs from one study group to the other, contamination of the study by another program, or non‐compliance to the assigned intervention status, has been assessed under deviations from intended interventions. Only two randomised studies have outcomes that had high risks of deviations. One of the outcome in Giné et al. (2018) was assessed at the level above the unit of randomization, the Basic Health Unit, which was served by control and treatment communities. Berman et al. (2017) mentions issues in the implementation of the random assignment leading initially assigned control community to receive the treatment. For 21 per cent of outcomes in randomised and 48 per cent of outcomes in non‐randomised studies, authors did not report on the geographical distance between intervention and comparison groups, or failed to justify the absence of spill overs when there was a potential risk.

##### Performance bias

Another potential bias occurring during the data collection process is performance bias: the fact that monitoring participants influences their behaviours because they are aware of being watched (Hawthorne effect). A majority of randomised studies are protected from this bias (56%). When a process evaluation of the intervention was conducted (Fiala & Premand, 2017) it was done on a subsample of the treatment group. Banerjee et al. (2014), which was also at risk of motivation bias due to the decoy visits used as a monitoring technique, overcame this risk by adding a pure control study arm (placebo group), free from monitoring visits.

##### Outcome measurement bias

With regards to outcome measurement bias, which refers to cases where the way the outcome is being measured differs between treatment and control participants as a result of the intervention, it is worth noting that around 65 per cent of the primary outcomes in these studies are self‐reported, increasing their exposure to bias. Figure 7 and Figure 8 illustrate this with 20 per cent of the study outcomes being unclear, probably not free or not free from outcome measurement bias for randomised studies and 34 per cent for non‐randomised studies. An illustration of why this bias is greater when it is self‐reported is a situation where the participants receive information about what the expected behaviours or beliefs are, and then are asked about their own behaviours and beliefs. This issue exists with Kasim (2016), where people receive text messages about their rights with regards to certain institutions and are then asked to rate their trust toward these institutions. Measuring participants’ trust in religious institutions was used as a “placebo outcome”, which attempts to measure the effect of possible social desirability bias in survey responses by collecting self‐reported outcomes on which no information was provided as part of the intervention.

Five studies included in this review evaluate community‐based monitoring of health services (Alhassan et al. 2015 Alhassan et al. 2016, Björkman & Svensson, 2010, Björkman Nyqvist et al. 2017, Fiala & Premand 2017, Gullo et al. 2017). Because the main intervention aims to engage citizen in monitoring health worker's performance, the service users in the intervention groups in these studies are more likely to remember the services they received over the past year because they paid attention to it (recall bias). Similarly, the service providers in this setting have incentives to over‐report on their performance.

In situations where there are risks of measurement bias known from the start, four studies collect data from different sources so that they do not rely only on a biased estimate. This is what researchers have done in Berman et al. (2017) to measure road quality. Given that there is a high risk of outcome measurement bias in asking villagers who have been taught how to assess quality as part of the intervention, they have measured this outcome using both villagers report and a technical assessment.

The bias could also come from the outcome assessors, if they know the respondent's treatment status. This could still be a risk for all studies because none of them blinded outcome assessors except one (Pandey et al., 2007).

##### Analysis and reporting bias

The randomised study designs ensured comparability of groups for the analysis of almost all outcomes. As a result, 70 per cent of all outcomes in randomised studies were free or probably free from confounding. However, depending on the sample size and the randomisation procedures, some imbalances can occur by chance. The majority of authors identified these imbalances and controlled for relevant variables in the analysis method, whereas in 26 per cent of the cases, it was not clear whether imbalanced variables were controlled in adjusted analysis.

Although 12 out of the 14 non‐randomised studies used the appropriate method to control for group differences given the data available, the existing selection bias into the programme and the lack of baseline data explains why more than 60 per cent of studies did not ensure group equivalence on all relevant variables. Despite the use of combinations of matching techniques with difference‐in‐difference estimations, it was sometimes unclear whether unobservable characteristics could be accounted for (19 per cent of the outcomes).

Out of all studies, only one blinded data analysts to the treatment (Humphreys et al., 2012).

Overall, for randomised and non‐randomised studies alike, there is a lack of transparency and reporting. Non‐randomised studies do not systematically report results using different analysis methods and specifications, which is often key to assessing the robustness of their model. Three studies out of eight using statistical matching reported estimation from different matching techniques. The existence of a pre‐analysis plan, published before the start of the analysis, or a trial registration is rare across all types of studies. None of the non‐randomised studies and only three randomised studies reported having registered the trial or a list of outcomes (Banerjee et al., 2018, Pandey et al., 2007 and Fiala & Premand, 2017). Only three study reported having published a pre‐analysis plan (Beath et al., 2013, Grossman et al., 2017 and Humphreys et al., 2012). The 42 per cent of randomised studies being probably free from analysis reporting are studies which have been reported transparently but have not registered either trial, outcomes or pre‐analysis plan, therefore we cannot be certain that all relevant analyses are reported. Finally, two randomised studies failed to report analysis differentiating treatment arms (Alhassan et al., 2015; and Kasim, 2016).

More generally, as Figure 7 and Figure 8 illustrate, there is, for all criteria, a share of studies and outcomes which could not be assessed because of a lack of information (grey areas). Overall, it is sometimes the case that there is some doubt about a risk of bias, which could have been eliminated if more information on the issue was provided. These issues were particularly problematic for method of assignment (randomisation procedures), reporting of baseline data and attrition.

#### 6.3.2 Research ethics

We also captured information on whether the paper explicitly stated that the authors had ethical clearance to undertake the study. Of the 35 included studies, the majority (28) collected primary data for analysis. However, just three of the included studies reported that they had sought and received ethical clearance for their studies. The rest did not report whether ethical clearance to undertake the research was sought or granted; they may well have done, but they simply do not indicate whether this was the case in the country where the data were being collected and (if different) where the research team was based. In addition, we looked for declarations of interest in the included studies, to capture for example if any of the authors related in any way to the funding or implementing institution. We found that only two studies included conflict of interest statements. In 18 of the studies, the authors did not include a statement or did not present a statement that clearly reported on possible conflicts (known or unknown) for all authors.

#### 6.3.3 External validity

Several factors need to be taken into account when assessing the external validity of studies such as the approach used by researchers to select the study population, whether the programme implemented was a small scale pilot or a large scale established program, and the characteristics of the population and setting of the study. We captured information on the sampling strategies, as well as authors’ discussions of generalisability of their findings.

##### Selection of the study population

We identified nine studies in which random sampling was used to either select the study's geographical areas such as regions and districts, or select the clusters or units of treatment such as communities, facilities and villages. Twenty one used purposive sampling and four did not provide enough information on their method or the origin of the data set used. Table 6 shows which studies have used each of the sampling strategies, and separate the results by treatment assignment mechanism and whether survey respondents were randomly sampled.

**Table 6:** Sampling strategy used to select communities and villages

Population selection	Random sampling of survey respondents	Random allocation to treatment	Non‐random selection into treatment
Randomly sampled regions (or any geographical unit above cluster)	Yes	Beuermann & Amelina (2014), Björkman and Svensson (2010), Humphreys & Weinstein (2012)	Capuno & Garcia (2010)
	No		Timmons & Garfias (2015)
Randomly sampled clusters within purposely selected regions	Yes	Ravallion (2013), Grossman et al. (2017)	Bandyoadhyay (2010)
	No		Huang (2014)
Purposive sampling of clusters	Yes	Giné et al (2018), Ananthpur et al. (2014), Beath et al. (2013), Banerjee et al. (2018), Banerjee et al. (2014), Grossman & Michelitch (2018), Humphreys et al (2014)	Tachibana & Adhikari (2009), Palladium (2015), Persha & Meshack (2016), Rasamoelina et al. (2015)
	No	Alhassan et al. (2016), Gullo et al. (2017), Pandey et al. (2007), Olken (2007), Berman, 2017	Touchton & Wampler (2014), Molina (2014), Goncalves (2013), Bradley & Igras (2005), Barde (2017),
Unclear		Kasim (2016), Fiala & Premand (2017)	Bandyoadhyay (2004), Diaz‐cayeros et al. (2014)

Knowledge of the sampling method is not sufficient on its own and, more attention to each study is needed to be able to conclude on the representativeness of the populations selected. Of the studies which used random sampling, three did not include randomly selected regions but researchers selected the communities within the regions randomly. One decided to include a representative population of the country by randomly selecting regions or districts but then purposely selected villages.

Of the studies which used only purposive sampling, two reported specific exclusion criteria which might limit the generalisability of their results. For instance, Alhassan et al. (2016) mentions that health facilities were selected because they were less complex and easy to monitor. Gullo et al. (2017) selected areas where not many NGOs were already present to avoid contamination. Two studies had to drop communities or facilities from their sample because of constraints related to their randomization method (Berman et al., 2017; Gullo et al., 2017). Two studies selected areas specifically for their representativeness of the state or country population (Banerjee et al., 2014; Berman et al., 2017). Ananthpur et al. (2014) specifically targeted the poorest area in the state. Another selection criteria was availability of data, especially for non‐randomised retrospective studies using existing data sets. A few authors mention that villages where survey or administrative data was already available from previous studies were selected to be part of the evaluation (Banerjee et al., 2018; Pandey et al., 2007; Gonclaves, 2013). Finally, three studies evaluating the impact of an established programme were restricted to the area or communities where the NGO or the government was implementing or had had the program (Beath et al., 2013; Giné et al., 2018; Molina, 2014).

##### Author discussion of external validity

We found 11 studies where authors specifically discussed external validity. Among those studies, five acknowledged the limits to the generalisability of their findings, due to the small scale of the study or the sampling strategy. Four studies claimed generalisability of their findings, either to the level of an Indian state (Banerjee et al., 2014; Ravaillon et al., 2013), or to other areas of the country under similar conditions, such as density of population or distance to a health facility (Toutchon, 2015; Björkman Nyqvist et al. 2017). Finally, two studies claimed generalisability of their findings to other contexts, and potentially other countries (Fiala & Premand, 2017; Timmons & Garfias, 2015).

#### 6.3.4 Summary of findings from critical appraisal

The quality of evidence from randomised studies is relatively high compared to non‐randomised studies, and easier to assess due to standards of reporting for those studies. Prospective randomised study design helped ensured comparability of intervention and control groups according to observable characteristics, and protected threats from selection bias into the study in 70 per cent of the cases. For these studies, threats to internal validity are therefore more relevant at the outcome level, where concerns related to the way some outcomes are measured in the majority of studies. This is largely due to the use of self‐report measures that are more likely to be biased in open studies where blinding (of outcome data collectors or participants) is not attempted or impossible. A majority of the non‐randomised studies did not provide enough information on the selection process into the programme to reject the risk of selection bias, or failed to overcome the selection bias and confounding that was identified. Transparency in reporting is an issue for randomised and non‐randomised studies alike given the limited pre‐registrations of trial, outcomes or analysis plans. The use of methods such as placebo outcomes or groups, and blinding for outcome assessors or data analysts, is not common, though it seems relatively easy to implement and could reduce risks of biases. With regards to external validity, four studies still do not report their sampling strategies clearly, and a surprisingly small share of all studies specifically discuss the extent or limits to generalisability of their findings.

### 7 RESULTS OF META‐ANALYSIS (REVIEW QUESTIONS 1‐3)

In this section, we describe the quantitative dataset and outcome variables classification. We present the results of meta‐analysis across all included studies, by primary outcomes along the causal chain (*review question 1*). We then examine findings for secondary outcomes (*review question 2*). In both instances, we assess the extent to which findings are homogeneous for groups of interventions that aim to address different participation, inclusion, transparency and accountability mechanisms. Finally, we further examine heterogeneity according to context and implementation factors, as well as differential effects for sub‐groups of participants such as poor people (*review question 3*).

**Figure 9:**
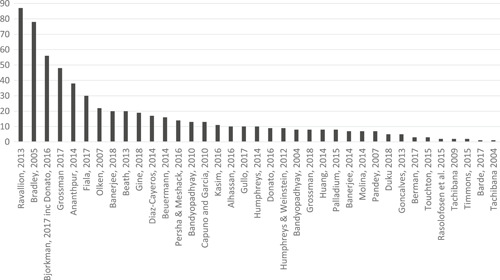
Number of effect sizes collected from included studies

As discussed, we collected all effect estimates from each included study, on any eligible outcome, population sub‐group or specification. Hence for some studies we collected large numbers of effects. Figure 9 presents the number of effect estimates collected from each study that we were able to incorporate in meta‐analysis.

In total the 35 studies yielded 618 estimates of programme impacts that we incorporated in meta‐analysis. All studies provided usable data for effect size calculations. In cases where pooled standard deviations were not available, we had to rely on t‐statistic transformations to calculate *g* and its standard error. The effect sizes are unevenly distributed between studies. The largest numbers of effect sizes were from Ravallion et al. (2013) in Rajasthan, India, with 87 effect estimates used in the analysis, followed by Bradley & Igras (2005) in Guinea and Kenya with 78 effect estimates, (Björkman & Jakob, 2009; Björkman Nyqvist et al. 2017; also incorporating Donato & Garcia Mosqueira, 2016) in Uganda with 56 effect estimates extracted, and Grossman et al. (2017) in Uganda with 48. However, the majority of studies presented far fewer effect estimates, usually less than 20.

**Figure 10:**
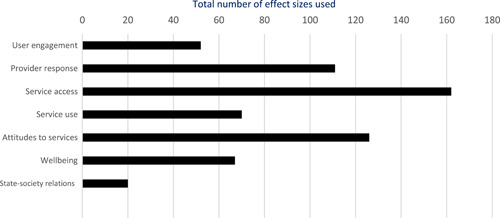
Number of effect sizes by outcome along causal chain

We assigned specific sub‐categories of outcomes (e.g. participation in meetings) to causal chain outcome groupings: intermediate outcomes (service access, service use and attitudes to services), final outcomes (wellbeing and state‐society relations) (review question 1), and immediate outcomes (user engagement and provider response) (review question 2). Figure 10 presents the number of effect sizes collected for each outcome, together with the distribution of effect size estimates, showing the mean, minimum and maximum values of g.

We drew on a recent review of community‐driven development (White et al., 2018) in informing the outcome groupings along the causal chain, as presented here. Table 7 presents the detailed description of variables included under each outcome area. As these may differ by projects, these are presented by main sector (health, social protection, justice and security, local infrastructure and economy, and natural resources). The full list of variables collected under each outcome category is presented in Appendix 6.

**Table 7:** Description of outcome variables included in meta‐analysis

	Immediate outcomes		Intermediate outcomes		Final outcomes	
	*User engagement*	*Provider response*	*Service access*	*Service use and attitudes*	*Wellbeing*	*State‐society relations*
Health	Participation in project meetings	Public spending	Access	Use of services	Illness^*^	Corruption
	Active participation (spoke at meeting)	Provider actions (prepare plan, public monitoring	(*Anganwadi*, lady health worker in village)	(health facility visit, immunisation (partial or full), antenatal / postnatal care, family	Death^*^ (IMR, U5MR)	(perceptions about corruption, payment made to official^*^)
					Births	
	Knowledge of project processes (knows about meetings)	or reporting such as staff duty roster, suggestion box)	Measured quality of service (health facility performance, wait time^*^, staff experiencing negative event^*^, lack of supplies^*^)	planning service, enrol in insurance)	Nutrition outcomes (stunting incidence^*^, wasting incidence^*^)	
	Knowledge about services available identifies services)	Project staff motivation (facility management, morale)	Absenteeism (workers not present^*^, effort index)	User satisfaction (perception of quality, complaint lodged^*^, friendliness of staff, felt able to convey concerns)	Income and expenditure	
	Participation in project targeting (number people/ women involved)	Provider actions (reported change in procedure)	Cost of service (user fees^*^)	Use of nutrition services (used food subsidy, amount purchased)	Household/personal assets	
			Access (received social security card)	Use of employment services (participate in employment opportunities, wages)	Employment (wages)	
Social protection	Knowledge about services (women have right to participate, facilities knowledge)	Responses perceived by user (better targeting)	Measured quality of service (received entitlement, employment opportunities) Leakages (embezzlement^*^)	User satisfaction (complaints reported^*^, protests reported^*^, perceived quality of staff)	Social capital (feelings of personal influence, social cohesion)	
Justice and security		Response perceived by user (change in police performance)		Perceived quality of service (perception of police awareness of crime)	Security (being a victim of crime^*^, trust, feeling safe against theft or attack)	Public confidence in institutions (fear of police, police responsiveness, trust government) Corruption perceptions^*^
Local infrastructure and economy	Participation in project meetings	Public spending Provider actions (meetings held)	Access (construction of handpumps/ tubewells) roads)	Use infrastructure (water use)	Yield/production	Public confidence (satisfaction with leaders, officials, politicians) Paid taxes
	Active participation (spoke at meeting)	Response as perceived by user (e.g. priorities match)	Measured quality of service (maintenance of facilities, visits from extension worker)	User satisfaction (satisfaction with roads, municipality cleanliness, sanitation service,		
		Politician performance (e.g. politician responds)	Leakages (quality of infrastructure^*^)	Perceived quality of service (attitudes to community assets)		
Natural resources	Active participation (prepare maintenance plan)		Access (access to natural resources)	Use of environmental resource (deforestation^*^, forest regeneration)		Public confidence (satisfaction in NRM committee, forest governance institutions)
						Paid taxes (contribution to community funds
						‐ e.g. irrigation service fees)

*Note:*

^*^ indicates negative outcome.

### 7.1 Meta‐analysis of intermediate and final outcomes (review question 1)

We present findings by primary outcome group and subgroups along the results chain (intermediate and final outcomes). In each sub‐section, we first present an overview of the different outcome metrics used in each study included in meta‐analysis (for the full list, see Appendix 6) and then present the subsequent meta‐analysis results including forest plots. When presenting the meta‐analysis, we present sensitivity analyses to disaggregate findings by study design (whether randomised or non‐randomised) and risk of bias status. Owing to the large number of outcomes collected, we present all effect sizes as standardised mean differences for ease of presentation.^5^ The total number of study participants across all studies included in the analysis is 62,500.

In general, the findings suggest that the interventions can be effective ways of boosting citizen engagement in service delivery governance and access to public services. But the evidence does not suggest that outcomes further along the results chain typically improve as a result of interventions to promote citizen engagement. In a few cases, particularly in health and infrastructure, there may be increases in service use and some wellbeing outcomes. For state‐society relations, payment of taxes may increase.

#### 7.1.1 Service access

A mix of variables was used to measure physical access including new amenities available in the community, such as water sources (Ananthpur et al., 2014; Barde et al., 2017; Diaz‐Cayeros et al., 2014; Grossman, 2017; Humphreys & Weinstein, 2012), roads, (Ananthpur et al., 2014), health units (Björkman Nyqvist et al. 2017), or new health staff posted in the community like *Anganwadi* (community creche) workers (Ananthpur et al., 2014) and lady health workers (Giné et al., 2018). Access is also measured through costs to consumers in two studies: subsidies received (Banerjee et al., 2018) and user fees paid in health (Giné et al., 2018).

Quality of service provision was assessed through measures of service provision performance such as whether there are employees in the *Anganwadi* or agricultural extension visits occur (Ananthpur et al., 2014), condition of health facilities (Alhassan et al., 2015; Björkman Nyqvist et al. 2017; Giné et al., 2018; Grossman, 2017; Gullo et al., 2017) or quality of health care received (Bradley, 2005), quality of roads (Berman et al., 2017) and irrigation provision (Bandyopadhyay et al., 2010), or environmental services like forestry cover (Persha & Meshack, 2016; Rasolofoson et al., 2015; Tachibana et al., 2004). Quality was also assessed by absenteeism in several studies in Uganda (Björkman & Jakob, 2009; Humphreys & Weinstein, 2012; Grossman, 2017; Grossman & Michelitch, 2018), which we report separately. The final measure of quality in service delivery was measured by leakages of public goods from road construction (Olken, 2007) and food aid (Beath et al., 2013).

**Figure 11:**
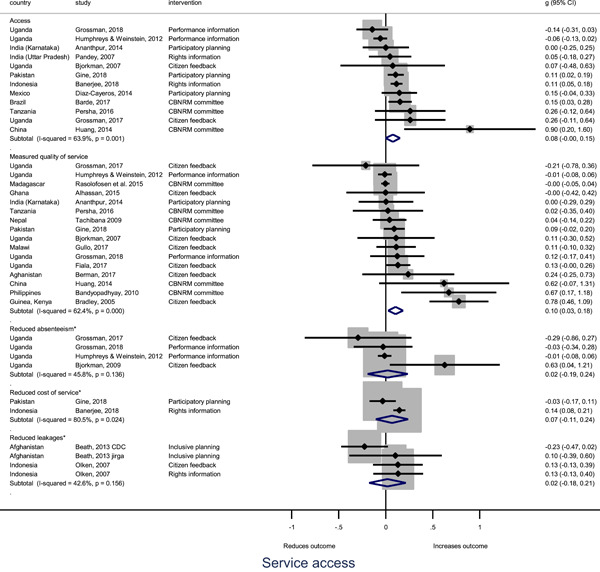
**Forest plots showing service access outcomes** Note: * effect sizes for negative outcomes are inverted for comparability

The overall findings suggest some improvement in access for some measures of service delivery (Figure 11). This is demonstrated by an increase in average effects of physical access (SMD=0.08, 95% confidence interval (CI)=0.00, 0.15; 12 studies), and service quality (SMD=0.10, 95%CI=0.03, 018; 16 studies). However, improvements in other outcomes were not apparent, including for reducing absenteeism (SMD=0.02, 95% confidence interval (CI)=‐0.19, 0.24; four studies), leakages from embezzlement (SMD=0.02, 95% confidence interval (CI)=‐0.18, 0.21; four studies) and costs paid for access (SMD=0.07, 95% confidence interval (CI)=−0.11, 0.24; 2 studies).

There was significant heterogeneity which we explored in sensitivity analysis (Table 8; forest plots presented in Appendix 6). The results indicate that the findings for non‐randomised studies tend to be bigger than those for RCTs, while results for risk of bias categories vary, although there are positive significant effects for low risk of bias studies measuring physical access (SMD=0.12, 95% confidence interval (CI)=0.06, 0.17; four studies) and quality of service (SMD=0.12, 95% confidence interval (CI)=0.01, 0.24; three studies)

**Table 8:** Service access by study design and intervention

*Outcome*	*Moderator*	*g*	*95%CI*		*I‐sq*	*Tau‐sq*	*Q*	*P‐value*	*N obs*
Physical access	Total	0.075	−0.001	0.152	63.9%	0.0084	30.49	0.001	12
	RCT	0.051	−0.027	0.128	65.4%	0.0071	23.10	0.003	9
	NRS	0.297	−0.014	0.609	52.6%	0.0396	4.22	0.121	3
	Low RoB	0.118	0.064	0.172	0.0%	0.0000	0.73	0.867	4
	Some concerns	0.057	−0.184	0.299	70.7%	0.0308	6.84	0.033	3
	High RoB	0.081	−0.069	0.230	74.7%	0.0157	15.82	0.003	5
Service quality	Total	0.105	0.026	0.184	62.4%	0.0103	39.94	0.000	16
	RCT	0.045	−0.005	0.096	0.0%	0.0000	6.66	0.672	10
	NRS	0.287	0.031	0.544	84.7%	0.0716	32.78	0.000	6
	Low RoB	0.127	0.011	0.243	0.0%	0.0000	0.01	0.996	3
	Some concerns	0.018	−0.153	0.190	0.0%	0.0000	0.69	0.405	2
	High RoB	0.127	0.023	0.232	72.5%	0.0141	36.42	0.000	11
Reduced absenteeism	Total	0.022	−0.193	0.236	45.8%	0.0216	5.54	0.136	4
	RCT	0.022	−0.193	0.236	45.8%	0.0216	5.54	0.136	4
	NRS	‐	‐	‐	‐	‐	‐	‐	0
	Low RoB	−0.028	−0.341	0.284	‐	‐	‐	‐	1
	Some concerns	−0.294	−0.863	0.274	‐	‐	‐	‐	1
	High RoB	0.240	−0.372	0.852	77.9%	0.1591	4.52	0.034	2
Reduced leakage	Total	0.019	−0.177	0.215	42.6%	0.0167	5.23	0.156	4
	RCT	0.019	−0.177	0.215	42.6%	0.0167	5.23	0.156	4
	NRS	‐	‐	‐	‐	‐	‐	‐	0
	Low RoB	0.131	−0.056	0.319	0.0%	0.0000	0.00	0.987	2
	Some concerns	−0.132	−0.424	0.159	27.4%	0.0149	1.38	0.241	2
	High RoB	‐	‐	‐	‐	‐	‐	‐	0
Reduced cost of service	Total	0.067	−0.105	0.239	80.5%	0.0127	5.13	0.024	2
	RCT	0.067	−0.105	0.239	80.5%	0.0127	5.13	0.024	2
	NRS	‐	‐	‐	‐	‐	‐	‐	0
	Low RoB	0.145	0.080	0.209	‐	‐	‐	‐	1
	Some concerns	‐	‐	‐	‐	‐	‐	‐	0
	High RoB	−0.033	−0.172	0.107	‐	‐	‐	‐	1

Note: ‐ not applicable.

#### 7.1.2 Service use and attitudes to services

Service use was measured in health and social protection sectors. Various measures of health care for children were collected such as immunisation (e.g. Donato & Garcia Mosqueira, 2016; Giné et al., 2018) and nutrition supplements (Grossman, 2017), and mothers such as use of antenatal and postnatal care (Grossman, 2017; Gullo et al., 2017). In one social protection study, the authors measured participation in employment services (Ravallion et al., 2013).

User satisfaction was measured through satisfaction surveys in health (Duku et al., 2018; Giné et al., 2018), policing (Banerjee et al., 2014), general satisfaction with local amenities provided by government including infrastructure (Beuerman et al., 2014; Molina, 2014) and employment services (Ravallion et al., 2013), and complaints reported (Banerjee et al., 2018). User satisfaction with service delivery staff was also assessed in policing (Banerjee et al., 2014), health (Bradley et al., 2005; Giné et al., 2018) and family planning (Gullo et al., 2017) and in local leadership (Fiala & Premand, 2017; Molina, 2014). One study also measured perceived user rights to employment services for women (Ravallion et al., 2013).

**Figure 12:**
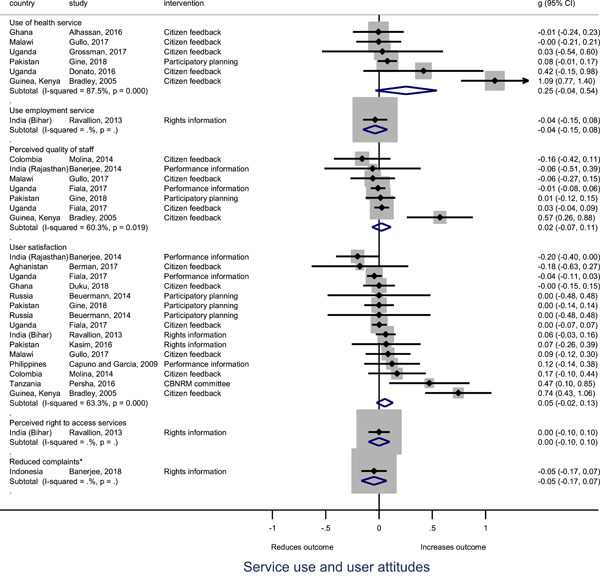
**Forest plots showing service use outcomes** Note: * effect sizes for negative outcomes are inverted for comparability

The results of the meta‐analysis (Figure 12) indicate that we do not observe any changes in use on average for health services (SMD=0.25, 95% confidence interval (CI)=−0.04, 0.54; six studies), user satisfaction (SMD=0.05, 95% confidence interval (CI)=−0.02, 0.13; 15 studies) or perceived quality of service provision (SMD=0.02, 95% confidence interval (CI)=−0.07, 0.11; seven studies). There also appeared to be significant heterogeneity in the findings although this was not related to study design or risk of bias (Table 9).

**Table 9:** Service use and satisfaction by study design and intervention

*Outcome*	*Moderator*	*g*	*95%CI*		*I‐sq*	*Tau‐sq*	*Q*	*P‐value*	*N obs*
Service use	Total	0.254	−0.035	0.544	87.5%	0.1011	40.00	0.000	6
	RCT	0.065	−0.012	0.141	0.0%	0.0000	2.35	0.672	5
	NRS	1.086	0.769	1.403	‐	‐	‐	‐	1
	Low RoB	0.417	−0.146	0.981	‐	‐	‐	‐	1
	Some concerns	0.078	−0.011	0.167	0.0%	0.0000	0.03	0.874	2
	High RoB	0.349	−0.269	0.967	94.5%	0.2809	36.43	0.000	3
User satisfaction	Total	0.035	−0.042	0.112	66.9%	0.0097	33.18	0.000	12
	RCT	−0.011	−0.052	0.030	8.1%	0.0003	8.70	0.368	9
	NRS	0.336	−0.034	0.707	80.9%	0.0867	10.49	0.005	3
	Low RoB	−0.024	−0.070	0.021	0.0%	0.0000	1.06	0.589	3
	Some concerns	−0.053	−0.309	0.204	81.3%	0.0282	5.34	0.021	2
	High RoB	0.147	−0.032	0.325	69.8%	0.0382	19.90	0.003	7
Perceived quality	Total	0.027	−0.082	0.136	66.9%	0.0091	15.11	0.010	6
	RCT	0.004	−0.043	0.051	0.0%	0.0000	0.94	0.815	4
	NRS	0.202	−0.511	0.916	91.8%	0.2434	12.23	0.000	2
	Low RoB	0.008	−0.040	0.056	0.0%	0.0000	0.50	0.480	2
	Some concerns	−0.061	−0.512	0.391	‐	‐	‐	‐	1
	High RoB	0.108	−0.292	0.507	85.9%	0.1064	14.22	0.001	3

#### 7.1.3 Wellbeing

A variety of wellbeing outcomes among study participants were measured. Health outcomes included mortality (Touchton & Wampler, 2015; Björkman Nyqvist et al. 2017; Giné et al., 2018), illness (Duku et al. 2018; Giné et al., 2018), fertility (Björkman Nyqvist et al. 2017; Donato & Garcia Mosqueira, 2016) and anthropometry (Björkman Nyqvist et al. 2017; Giné et al., 2018). Several studies reported agriculture yields (Bandyopadhyay et al., 2010; Huang et al., 2014) and livestock (Fiala & Premand, 2017). Other studies measured feelings of empowerment in and of the community (Fiala & Premand, 2017; Humphreys et al., 2014) and social cohesion via presence and membership of civil society organisations (Ananthpur et al., 2014; Capuno & Garcia, 2010; Touchton & Wampler, 2015) or trust (Kasim, 2016). Kasim (2016) also measured life satisfaction.

**Figure 13:**
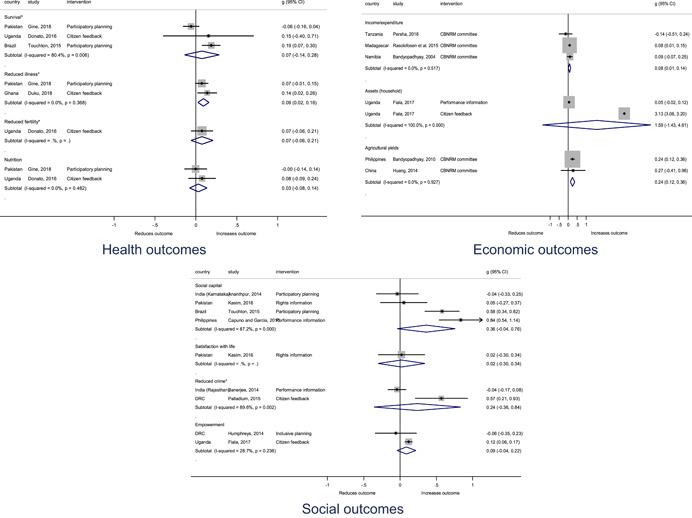
**Forest plots showing wellbeing outcomes** Note: * effect sizes for negative outcomes are inverted for comparability

Figure 13 presents forest plots for wellbeing outcomes (summarised in Table 10). These suggest outcomes may increase marginally, although usually not statistically significantly with the exceptions of reductions in illness (SMD=0.09, 95%CI=0.02, 0.16; 2 studies) in health. In the case of economic outcomes, there are improvements in yields (SMD=0.24, 95%CI=0.12, 0.36; 2 studies) and income/expenditure (SMD=0.08, 95%CI=0.01, 0.14; 3 studies). We do not see statistically significant findings for pooled effects in social wellbeing. It is also difficult to explore heterogeneity by study design in the cases of health and economic outcomes as health outcomes are mainly from RCTs while those in agriculture are all non‐randomised. For social outcomes, the heterogeneity observed appears to be due to study design, although all studies (randomised and non‐randomised) were assessed as being of high risk of bias. In general, there are too few outcomes of any type to draw conclusions.

**Table 10:** Wellbeing outcomes by study design and intervention

*Outcome*	Sub‐category	*g*	*95%CI*		*I‐sq*	*Tau‐sq*	*Q*	*P‐value*	*N obs*
Health	Survival	0.073	−0.139	0.284	80.4%	0.0233	10.21	0.006	3
	Reduced illness	0.092	0.024	0.159	0.0%	0.0000	0.81	0.368	2
	Reduced fertility	0.074	−0.061	0.210	‐	‐	‐	‐	1
	Improved nutrition	0.029	−0.078	0.136	0.0%	0.0000	0.50	0.482	2
Economic	Income/expenditure	0.076	0.011	0.141	0.0%	0.0000	1.32	0.517	3
	Agricultural yields	0.241	0.120	0.362	0.0%	0.0000	0.01	0.927	2
	Assets	1.588	−1.429	4.606	100.0%	4.7391	3668.48	0.000	2
Social	Social capital	0.361	−0.039	0.761	87.2%	0.1447	23.38	0.000	4
	Empowerment	0.089	−0.041	0.218	28.7%	0.0045	1.40	0.236	2
	Satisfaction with life	0.020	−0.303	0.343	‐	‐	‐	‐	1
	Reduced crime	0.239	−0.358	0.836	89.6%	0.1675	9.64	0.002	2

#### 7.1.4 State‐society relations

A few studies also measured the category of variable we have referred to (following Phillips et al., 2017) as state‐society relations, which are principally measures of the relationship between citizens and government. We categorised these into variables measuring taxes paid (Ananthpur et al., 2014; Beuermann & Amelina, 2014; Timmons et al., 2015) or in the case of natural resource management contribution to local service fees (Bandyopadhyay et al., 2010; Huang et al., 2014); feelings of trust in leadership and institutions (Fiala & Premand, 2017; Kasim, 2016); and, relatedly, public perception of corruption among public servants (Fiala & Premand, 2017) including the police (Banerjee et al., 2014).

**Figure 14:**
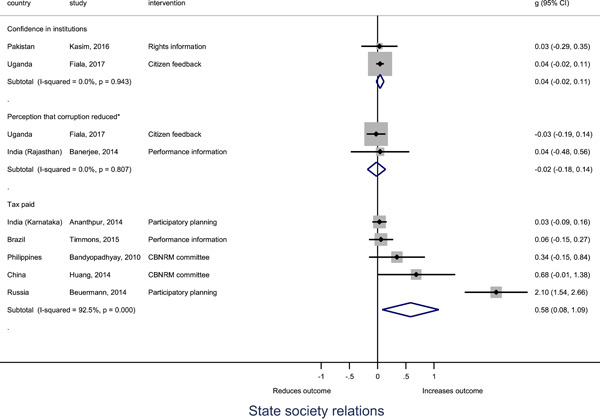
Forest plot showing state‐society relations outcomes Note: * effect sizes for negative outcomes are inverted for comparability

The results suggest that there have been improvements in taxes paid in individual studies and overall (SMD=0.58, 95%CI=0.08, 1.086; 5 studies) (Figure 14). There were no improvements for the other outcomes – corruption perceptions (SMD=−0.02, 95%CI=−0.18, 0.14; 2 studies) or confidence in institutions (SMD=0.04, 95%CI=−0.02, 0.11; 2 studies). Sensitivity analysis indicates that the estimated increase in tax paid is mainly due to the RCT of participatory budgeting training and technical assistance in Russia (Beuermann & Amelina, 2014) (Table 11).

**Table 11:** Wellbeing outcomes by study design and intervention

*Outcome*	*Moderator*	*g*	*95%CI*		*I‐sq*	*Tau‐sq*	*Q*	*P‐value*	*N obs*
Tax paid	Total	0.584	0.083	1.086	92.5%	0.2755	53.56	0.000	5
	RCT	1.048	−0.975	3.071	98.0%	2.0887	50.00	0.000	2
	NRS	0.246	−0.085	0.576	43.6%	0.0395	3.55	0.170	3
	Low RoB	2.099	1.541	2.657	‐	‐	‐	‐	1
	Some concerns	0.061	−0.152	0.273	‐	‐	‐	‐	1
	High RoB	0.238	−0.119	0.595	55.4%	0.0568	4.48	0.106	3

### 7.2 Meta‐analysis of immediate outcomes (review question 2)

We grouped immediate outcomes into user engagement and provider response, in order to break down the mechanisms through which interventions operate. In general, the findings suggest that citizen engagement interventions can be effective ways of boosting user engagement in service delivery governance, but not typically provider responsiveness. We conclude that we are able to go some way to explaining intervention mechanisms on demand and supply sides, articulating that the interventions are mainly successful in improve demand (user engagement) and not supply (provider engagement). However, heterogeneity in findings needs further explanation, which we return to below in moderator meta‐analysis and framework synthesis.

#### 7.2.1 User engagement

User engagement outcomes include knowledge about the processes of engagement with the intervention (Ananthpur et al., 2014; Björkman Nyqvist et al. 2017) or the services themselves that are available (Ananthpur et al., 2014; Ravallin et al., 2013; Banerjee et al., 2018). They also include measures of participation in the governance intervention, including meeting attendance (Ananthpur et al., 2014; Capuno and Garcia, 2010; Olken, 2007; Ravallion et al., 2013) and more active participation in processes such as public speaking (Björkman & Jakob, 2009; Björkman Nyqvist et al. 2017; Olken, 2007), and maintenance planning and expenditure (Huang et al., 2007; Bandyopadhyay et al., 2010). A few studies also measured knowledge about intervention processes (Ananthpur et al., 2014; Banerjee et al., 2018; Ravallion et al., 2013) or public services (Ananthpur et al., 2014; Björkman et al., 2018).

**Figure 15:**
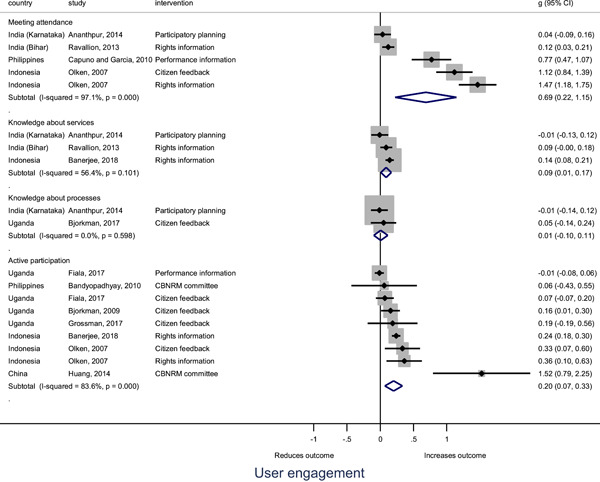
Forest plot showing service user engagement outcomes

It is worth noting that because these are secondary outcomes, which are reported in studies that also measure primary outcomes, the findings for immediate outcomes are only generalisable to the population of studies that also report immediate and final outcomes. We first present overall findings for user engagement (Figure 15). The evidence suggests that interventions appear to be particularly effective on average in getting citizens to attend meetings (SMD=0.69, 95%CI=0.22, 1.15; 5 studies), and to a lesser extent participate actively in intervention processes like speaking at meetings (SMD=0.20, 95%CI=0.07, 0.33; nine studies), and improving knowledge about services (SMD=0.09, 95%CI=0.01, 0.17; 3 studies). The two studies measuring knowledge about intervention processes did not find significant effects (SMD=0.01, 95%CI=−0.11, 0.11; 2 studies).

There was some heterogeneity in the findings which we explored in sensitivity analysis (Table 12). Most of the studies are RCTs so exploring differences by design were not especially useful. The findings suggested low risk of bias studies tended to have bigger effects than higher risk of bias studies.

**Table 12:** User engagement outcomes by study design and intervention

*Outcome*	*Moderator*	*g*	*95%CI*		*I‐sq*	*Tau‐sq*	*Q*	*P‐value*	*N obs*
Meeting attendance	Total	0.686	0.224	1.148	97.1%	0.2643	139.59	0.000	5
	RCT	0.666	0.141	1.191	97.7%	0.2752	128.09	0.000	4
	NRS	0.771	0.472	1.070	‐	‐	‐	‐	1
	Low RoB	1.289	0.945	1.632	67.1%	0.0412	3.04	0.081	2
	Some concerns	0.120	0.029	0.212	‐	‐	‐	‐	1
	High RoB	0.390	−0.330	1.111	94.9%	0.2566	19.70	0.000	2
Active participation	Total	0.203	0.072	0.334	83.6%	0.0248	48.64	0.000	9
	RCT	0.167	0.047	0.287	82.7%	0.0178	34.64	0.000	7
	NRS	0.766	−0.668	2.200	90.6%	0.9705	10.60	0.001	2
	Low RoB	0.172	0.021	0.323	88.4%	0.0229	34.46	0.000	5
	Some concerns	0.186	−0.188	0.560	‐	‐	‐	‐	1
	High RoB	0.492	−0.157	1.141	85.0%	0.2687	13.31	0.001	3
Knowledge about services	Total	0.090	0.012	0.169	‐	‐	‐	‐	1
	RCT	0.090	0.012	0.169	‐	‐	‐	‐	1
	NRS	‐	‐	‐	‐	‐	‐	‐	0
	Low RoB	0.144	0.081	0.206	‐	‐	‐	‐	1
	Some concerns	0.090	−0.001	0.180	‐	‐	‐	‐	1
	High RoB	−0.008	−0.135	0.119	‐	‐	‐	‐	1
Knowledge about processes	Total	0.008	−0.098	0.113	0.0%	0.0000	0.28	0.598	2
	RCT	0.008	−0.098	0.113	0.0%	0.0000	0.28	0.598	2
	NRS	‐	‐	‐	‐	‐	‐	‐	0
	Low RoB	0.050	−0.139	0.239	‐	‐	‐	‐	1
	Some concerns	‐	‐	‐	‐	‐	‐	‐	0
	High RoB	−0.011	−0.138	0.116	‐	‐	‐	‐	1

#### 7.2.2 Provider response

We categorised provider response variables into groups of related outcomes. A number of studies measured changes in public spending in health (Björkman Nyqvist et al. 2017; Grossman, 2017; Touchton & Wampler, 2015) or more generally (Beuermann & Amelina, 2014; Goncalves, 2013; Grossman & Michelitch, 2018). We also defined other provider actions relating to the citizen engagement intervention such as holding meetings (Pandey et al., 2007) or adopting processes like participatory budgeting (Timmons et al., 2015); or resulting from the engagement, such as activities carried out by staff (Ananthpur et al., 2014; Björkman Nyqvist et al. 2017; Diaz‐Cayeros, 2014) and projects selected (Humphreys et al., 2014). Two studies further measured variables relating to self‐motivation of staff governing the intervention (Alhassan et al., 2016; Bradley et al., 2005) or perceptions about politician performance (Diaz‐Cayeros et al., 2014; Grossman & Michelitch, 2018; Humphreys & Weinstein, 2012). Finally, a number of studies measured responsiveness of providers to the governance intervention as perceived by users (Ananthpur et al., 2014; Beath et al., 2013; Beuermann & Amelina, 2014; Capuno & Garcia, 2009; Fiala & Premand, 2017).

**Figure 16:**
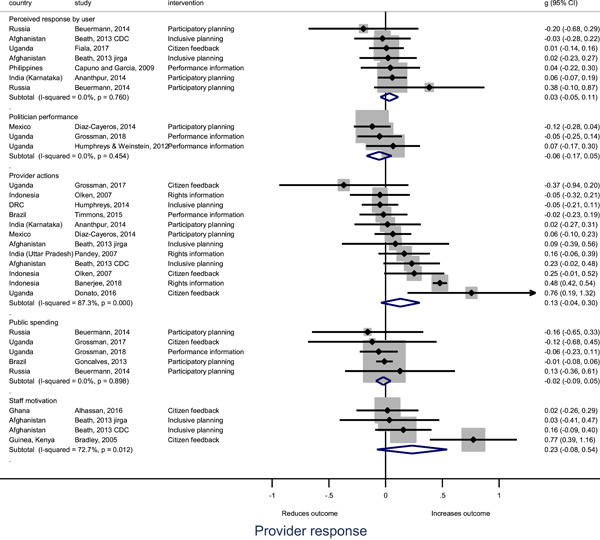
Forest plot showing provider responsive outcomes

On average, across those studies primarily concerned with intermediate and final outcomes, the findings do not suggest that the interventions improved provider response (Figure 16; Table 13). Thus, we were unable to find increases in public spending (SMD=−0.02, 95%CI=−0.08, 0.05; six studies), perceived response by users (SMD=0.03, 95%CI=−0.05, 0.11; 7 studies), staff motivation (SMD=0.23, 95%CI=−0.08, 0.54; four studies), and politician performance (SMD=−0.06, 95%CI=−0.17, 0.05; 3 studies).

**Table 13:** Provider response outcomes by study design and intervention

*Outcome*	*Moderator*	*g*	*95%CI*		*I‐sq*	*Tau‐sq*	*Q*	*P‐value*	*N obs*
Public spending	Total	−0.019	−0.084	0.046	0.0%	0.0000	1.10	0.954	6
	RCT	−0.053	−0.196	0.090	0.0%	0.0000	0.83	0.934	5
	NRS	−0.010	−0.084	0.063	‐	‐	‐	‐	1
	Low RoB	−0.004	−0.321	0.312	0.0%	0.0000	0.68	0.711	3
	Some concerns	−0.065	−0.226	0.095	0.0%	0.0000	0.04	0.851	2
	High RoB	−0.010	−0.084	0.063	‐	‐	‐	‐	1
Provider actions	Total	0.131	−0.040	0.302	87.3%	0.0696	86.37	0.000	12
	RCT	0.156	−0.040	0.352	86.5%	0.0743	66.61	0.000	10
	NRS	0.034	−0.095	0.163	0.0%	0.0000	0.37	0.544	2
	Low RoB	0.286	0.047	0.525	82.8%	0.0549	23.26	0.000	5
	Some concerns	0.027	−0.074	0.127	12.8%	0.0021	5.73	0.333	6
	High RoB	0.017	−0.275	0.308	‐	‐	‐	‐	1
Perceived response by user	Total	0.033	−0.046	0.112	0.0%	0.0000	3.38	0.760	7
	RCT	0.032	−0.051	0.115	0.0%	0.0000	3.37	0.643	6
	NRS	0.042	−0.219	0.303	‐	‐	‐	‐	1
	Low RoB	0.039	−0.195	0.274	0.0%	0.0000	2.95	0.229	3
	Some concerns	‐	‐	‐	‐	‐	‐	‐	0
	High RoB	0.038	−0.058	0.134	0.0%	0.0000	0.39	0.942	4
Staff motivation	Total	0.234	−0.077	0.544	72.7%	0.0712	11.00	0.012	4
	RCT	0.084	−0.085	0.254	0.0%	0.0000	0.62	0.733	3
	NRS	0.773	0.389	1.156	‐	‐	‐	‐	1
	Low RoB	‐	‐	‐	‐	‐	‐	‐	0
	Some concerns	0.127	−0.089	0.343	0.0%	0.0000	0.23	0.633	2
	High RoB	0.382	−0.359	1.123	89.9%	0.2575	9.95	0.002	2
Politician performance	Total	−0.058	−0.168	0.053	0.0%	0.0000	1.58	0.454	3
	RCT	−0.005	−0.156	0.147	0.0%	0.0000	0.57	0.449	2
	NRS	−0.118	−0.280	0.044	‐	‐	‐	‐	1
	Low RoB	‐	‐	‐	‐	‐	‐	‐	0
	Some concerns	−0.092	−0.217	0.033	0.0%	0.0000	0.25	0.615	2
	High RoB	0.066	−0.172	0.304	‐	‐	‐	‐	1

In the case of provider actions, there is significant heterogeneity in the effect (SMD=0.13, 95%CI=−0.04, 0.30; 12 studies). We also analysed the significant heterogeneity across studies by design and risk of bias. In general, the findings support the overall results that provider response outcomes are not significantly affected. There was some evidence that low risk of bias studies on average provided significant effects on provider actions (SMD=0.26, 95%CI=0.03, 0.48; six studies) (forest plot reported in Appendix 6).

### 7.3 Moderator analysis: analysis by intervention group and inclusion dimension

While these findings are instructive about the effects of governance interventions overall on intermediate and final outcomes, there is significant residual statistical and substantive heterogeneity. Here, we attempt to explain this by examining whether findings differ firstly by intervention group and secondly inclusion dimension. It is difficult to draw strong conclusions given the small sample sizes available at the individual intervention level. However, the findings suggest interventions focusing on rights information and community feedback appear may be effective in improving user engagement and service access. Interventions promoting participatory planning can be effective in improve service access, particularly where implementation is fully devolved through community‐based natural resource committees, where wellbeing and state‐society relations may also increase. On the other hand, interventions promoting performance information are not generally effective in improving any outcomes. Furthermore, most interventions have little if any effect on provider responsiveness and in most cases do not improve outcomes relating to use, wellbeing or state‐society relations.

### 7.3.1 Rights information

**Figure 17:**
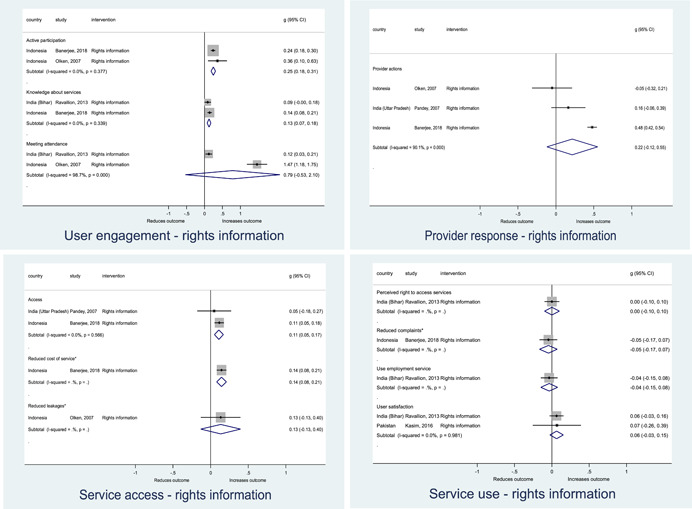
**Forest plots showing immediate and intermediate outcomes for rights information** Note: * effect sizes for negative outcomes are inverted for comparability

**Figure 18:**
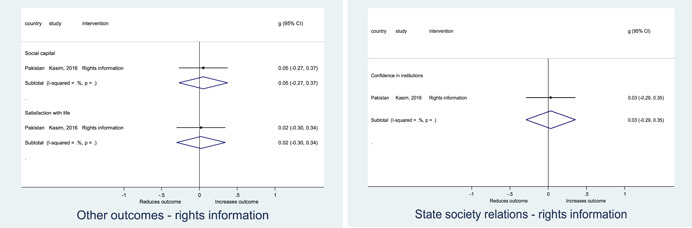
Forest plots showing final outcomes for rights information

The evidence suggests rights information interventions improve active participation (SMD=0.25, 95%CI=0.18, 0.31; 2 studies), as well as knowledge about services (SMD=0.13, 95%CI=0.07, 0.18; 2 studies) and meeting attendance (individual effect estates are positive and significant for Ravallion et al., 2013, and Olken, 2007) (Figure 17). Overall, the interventions do not necessarily improve provider responsiveness, although there is a significant improvement in the case of food subsidies in Indonesia (Banerjee et al., 2018). Service access also improves (SMD=0.11, 95%CI=0.05, 0.17; 2 studies) and costs fall (SMD=0.14, 95%CI=0.08, 0.21; one study, Banerjee et al., 2018) across the few studies available measuring those outcomes. However, the evidence does not suggest service use typically improves, with partial exception of user satisfaction that increases slightly but not significant across 2 studies (SMD=0.16, 95%CI=−0.03, 0.15; 2 studies). Only a single study (Kasim, 2016) measured any wellbeing or state‐society relations outcomes, and was not able to report any significant changes (Figure 18).

### 7.3.2 Performance information

**Figure 19:**
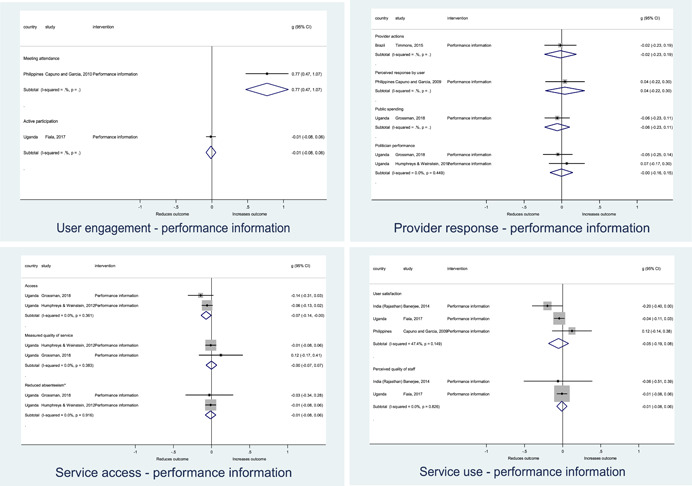
**Forest plots showing immediate and intermediate outcomes for performance information** Note: * effect sizes for negative outcomes are inverted for comparability

**Figure 20:**
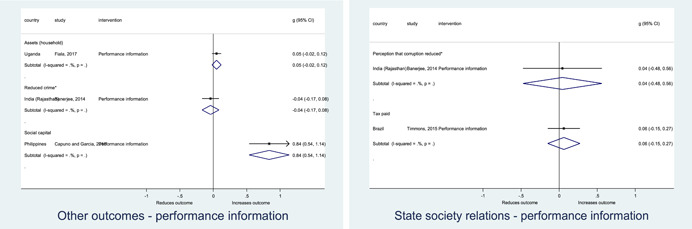
Forest plots showing final outcomes for performance information Note: * effect sizes for negative outcomes are inverted for comparability

As regards performance information, the six studies that evaluated this intervention type measured a wide range of outcomes, making it difficult to do much pooling in meta‐analysis. However, the evidence does not suggest intermediate, immediate outcomes or final outcomes in individual studies improve due to greater performance intervention (Figure 19, Figure 20). There is a partial exception in the case of one study (Capuno and Garcia, 2010).

### 7.3.3 Participatory planning

**Figure 21:**
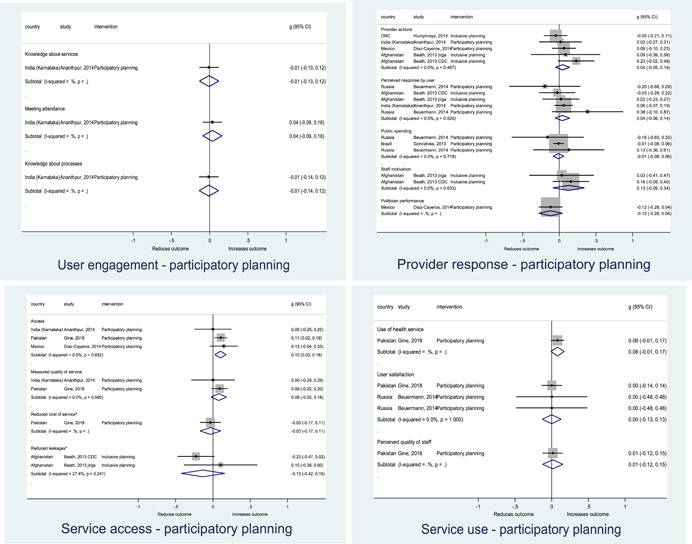
**Forest plots showing immediate and intermediate outcomes for participatory planning** Note: * effect sizes for negative outcomes are inverted for comparability

For participatory planning interventions, where seven studies measured a range of interventions, the story is mixed but largely not a positive one. Physical access to services improves on average (SMD=0.10, 95%CI=0.03, 0.18; 3 studies) (Figure 21). A few other outcomes are positive but not statistically significant, for example quality of service delivery (SMD=0.08, 95%CI=−0.02, 0.18; 2 studies) and use of health services and morbidity in Giné et al. (2018). In general, however, the evidence does not support increases in outcomes for other intermediate and final outcomes, for any low risk of bias study groups.

**Figure 22:**
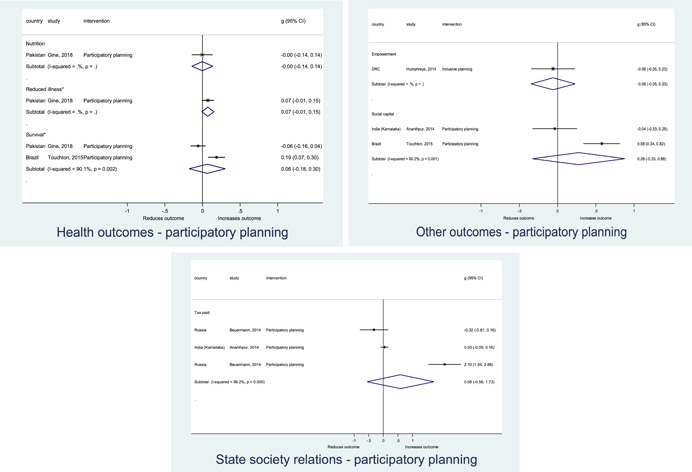
**Forest plots showing final outcomes for participatory planning** Note: * effect sizes for negative outcomes are inverted for comparability

Only one study was able to measure user engagement outcomes (Ananthpur et al., 2014). However, it is noteworthy that a relatively large number of studies that measured service access and wellbeing outcomes also measured provider response outcomes (Figure 22). The evidence does not suggest provider response improves on average or in individual studies, whether measured by provider actions (SMD=0.04, 95%CI=−0.05, 0.14; 5 studies), public spending (SMD=−0.01, 95%CI=−0.08, 0.06; 3 studies), perceived response by users (SMD=0.04, 95%CI=−0.06, 0.14; 5 studies) or staff motivation (SMD=0.13, 95%CI=−0.09, 0.13; 2 studies).

### 7.3.4 Citizen feedback mechanisms

**Figure 23:**
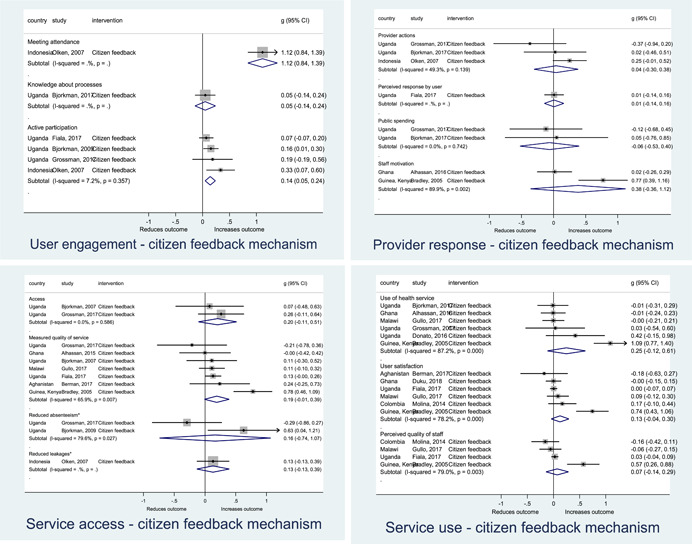
Forest plots showing immediate and intermediate outcomes for citizen feedback mechanisms Note: * effect sizes for negative outcomes are inverted for comparability

**Figure 24:**
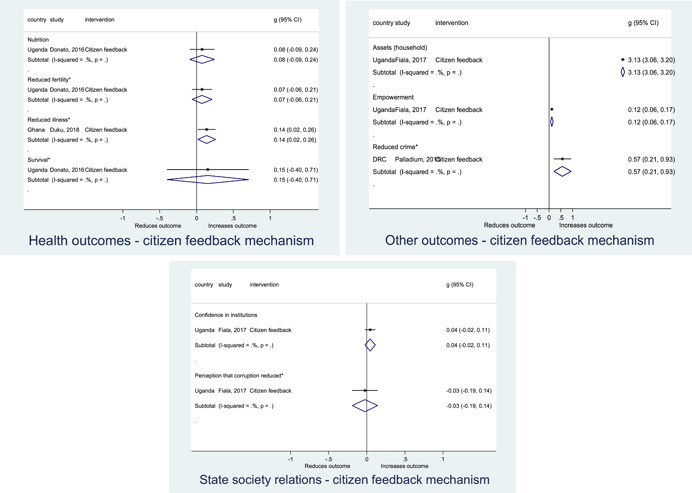
**Forest plots showing final outcomes for citizen feedback mechanisms** Note: * effect sizes for negative outcomes are inverted for comparability

The story for citizen feedback mechanisms is more positive, although there is significant heterogeneity in the findings. For evaluations that also measure primary outcomes, citizen engagement improves for active participation (SMD=0.14, 95%CI=0.05, 0.24; four studies) and in one study that measured meeting attendance (Olken, 2007). The meta‐analyses also did not suggest positive improvements in provider responsiveness on average, although some individual studies reported positive effects for provider actions (Olken, 2007) and staff motivation (Bradley et al., 2005) (Figure 23). Several service access and use outcomes were assessed as having increased on average but not statistically significantly, including service quality (SMD=0.19, 95%CI=−0.01, 0.39; 7 studies) and user satisfaction (SMD=0.13, 95%CI=−0.04, 0.30; six studies). Finally, a few single studies reported positive wellbeing outcomes for reducing illness (Duku et al., 2018) and crime (Palladium, 2015), and improving empowerment and assets (Fiala & Premand, 2017) (Figure 24). Only one study (Fiala & Premand, 2017) measured state‐society relations outcomes and was not able to detect significant changes due to citizen feedback mechanisms.

### 7.3.5 Community based natural resources management

**Figure 25:**
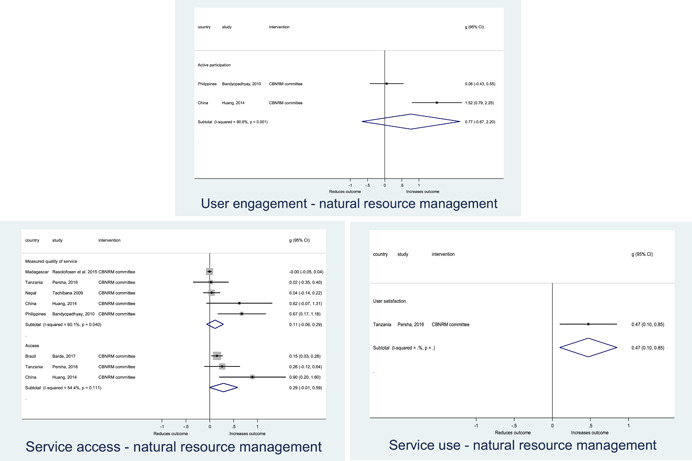
Forest plots showing immediate and intermediate outcomes for CBNRM

**Figure 26:**
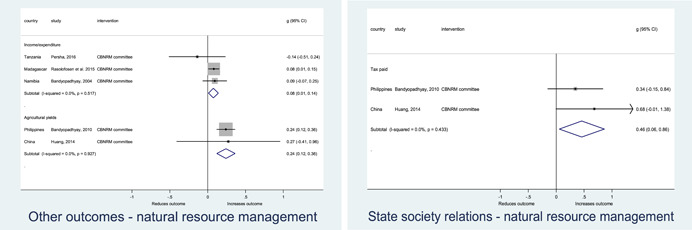
Forest plots showing final outcomes for CBNRM

To some extent the findings for CBNRM are less convincing than other interventions, because in the main the included studies were assessed as being of risk of bias largely on design grounds (the exception is for the RCT by Barde et al., 2017). The findings from meta‐analysis (Figure 25, Figure 26) suggested that final outcomes may improve for income/expenditure (SMD=0.08, 95%CI=0.01, 0.14; 3 studies), yield (SMD=0.24, 95%CI=0.12, 0.36; 2 studies) and tax payments (contribution to natural resource management) (SMD=0.46, 95%CI=0.06, 0.86; 2 studies).

### 7.3.6 Inclusion dimension

The analysis (reported in Appendix 6) suggests that where interventions had an inclusion dimension, the interventions tended to be at least as effective in improving outcomes, if not more effective (Figures A6.6 and A6.7).

### 7.4 Impacts by population group (review question 3)

This section presents results of sub‐group analysis for studies that report outcomes measured among different groups, including men, women and poor households. In addition, it presents further moderator analysis for whether interventions had an inclusiveness component by design and reporting outcomes by global region.

Three studies collected outcomes data measured separately among women and men (Ananthpur et al., 2014; Palladium, 2015; Ravallion et al., 2013) and a further five studies reported sub‐group outcomes solely for women (Beath et al., 2013; Diaz‐Cayeros et al., 2014; Fiala & Premand, 2017; Humphreys et al., 2014). The results, presented in Table 14 (see Appendix 6 for forest plots), do not suggest there are differences in outcomes by sex, where outcomes for both men and women are reported in the same studies. There are differences in magnitude in a few cases, such as the two studies of employment (Ravallion et al, 2013) and local governance (Ananthpur et al., 2014) in India. Indeed, Ananthpur et al. (2014) suggests that the positive wellbeing outcomes measured among men are not seen among women. However, there are very few observations where studies report sex disaggregated effects, and when they do the confidence intervals overlap, so any differences can be interpreted as statistically insignificant.

**Table 14:** Outcomes by sex sub‐group

*Outcome (study)*	*Sub‐group*	*g*	*95%CI*		*I‐sq*	*Tau‐sq*	*Q*	*P‐value*	*N obs*
Knowledge about services (Ravallion, 2013)	Male	0.090	−0.019	0.198	‐	‐	‐	‐	1
	Female	0.094	−0.010	0.198	‐	‐	‐	‐	1
Meeting attendance (Ravallion, 2013)	Male	0.110	0.000	0.220	‐	‐	‐	‐	1
	Female	0.128	0.030	0.227	‐	‐	‐	‐	1
Provider actions (Beath, 2013 CDC, *jirga*)	Male	‐	‐	‐	‐	‐	‐	‐	0
	Female	0.106	−0.070	0.281	0.0%	0.0000	0.04	0.836	2
Politician performance (Diaz‐Cayeros, 2014)	Male	‐	‐	‐	‐	‐	‐	‐	0
	Female	−0.198	−0.360	−0.036	‐	‐	‐	‐	1
Use employment service (Ravallion, 2013)	Male	−0.017	−0.156	0.122	‐	‐	‐	‐	1
	Female	−0.042	−0.192	0.108	‐	‐	‐	‐	1
User satisfaction (Ravallion, 2013)	Male	0.070	−0.043	0.183	‐	‐	‐	‐	1
	Female	0.039	−0.070	0.148	‐	‐	‐	‐	1
Perceived right to access service (Ravallion, 2013)	Male	−0.013	−0.133	0.107	‐	‐	‐	‐	1
	Female	0.005	−0.105	0.114	‐	‐	‐	‐	1
Assets (household) (Fiala, 2017)	Male	‐	‐	‐	‐	‐	‐	‐	0
	Female	0.009	−0.052	0.071	‐	‐	‐	‐	1
Income/expenditure (Ananthpur, 2014)	Male	0.285	−0.008	0.577	‐	‐	‐	‐	1
	Female	0.095	−0.196	0.387	‐	‐	‐	‐	1
Crime rates (Palladium, 2015)	Male	0.374	0.012	0.735	‐	‐	‐	‐	1
	Female	0.350	−0.012	0.712	‐	‐	‐	‐	1
Empowerment (Palladium, 2015)	Male	‐	‐	‐	‐	‐	‐	‐	0
	Female	−0.146	−0.434	0.142	‐	‐	‐	‐	1

Note: effect sizes for negative outcomes are inverted for comparability.

Three studies reported outcomes for poor households (Banerjee et al., 2018; Pandey et al., 2007; Persha & Meshack, 2016). In the case of Banerjee et al. (2018), the intervention targeted the poorest decile. In the case of Pandey et al. (2007) and Persha and Meshack (2017), outcomes are presented separately for lower‐caste communities and poor households. The findings suggest that outcomes for poor households are often positive and statistically significant (Table 15).

**Table 15:** Outcomes for poor sub‐group

*Outcome (study)*	*g*	*95%CI*		*I‐sq*	*Tau‐sq*	*Q*	*P‐value*	*N obs*
Physical access (Banerjee, 2018; Persha, 2017)	0.066	−0.006	0.137	0.0%	0.0000	0.18	0.672	2
Measured quality of service (Pandey, 2007)	0.221	−0.005	0.446	‐	‐	‐	‐	1
Cost of service (Banerjee, 2018)	0.084	0.009	0.159	‐	‐	‐	‐	1
User satisfaction (Persha, 2017)	0.449	0.073	0.826	‐	‐	‐	‐	1
Income/expenditure (Persha, 2017)	0.054	−0.319	0.428	‐	‐	‐	‐	1

However, there are too few observations to draw conclusions, other than that studies must more consistently present results of sub‐group analysis. Even where significant effects are not reported due to underpowered analyses, statistical synthesis (meta‐analysis) can be undertaken to detect possible effects across studies.

Finally, we conducted analysis by global region (Appendix 6). Bearing in mind that the analyses are likely to be confounded by other characteristics such as intervention type, we note simply that the analysis suggests intervention conducted in East Asia and Pacific and South Asia are more likely to have significant effects than studies conducted elsewhere.

### 7.5 Publication bias analysis

**Figure 27:**
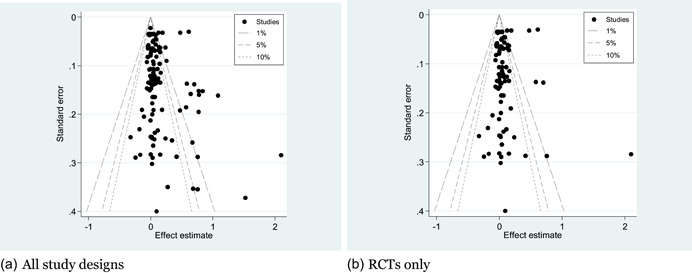
**Funnel graphs a)** All study designs b) RCTs only

This section presents results of the analysis of small study effects. Figure 27 presents contour enhanced funnel graphs for all study designs (part a) and for RCTs only (part b). There does appear to be asymmetry in the plot, which is markedly less for RCTs than all study designs. This may support Peters et al. (20o8) contention that bias may confound attribution of small study effects to publication bias. Eggers et al. (1997) test also did not find significant evidence for publication bias (Table 16).

**Table 16:** Results of Eggers tests

*Sample*	*Coeff*	*95%CI*		*p‐value*	*N obs*
All study designs	0.397	−0.417	1.212	0.336	113
RCTs	−0.644	−1.653	0.365	0.208	82

### 8 RESULTS OF FRAMEWORK SYNTHESIS (REVIEW QUESTION 4)

The following section presents the analysis of context and mechanisms that may contribute to findings along the causal chain (review question 4). We present the findings of a qualitative, realist‐informed framework synthesis that moves toward “best fit” framework synthesis, focusing on the key mechanisms and moderators along the casual chain for each broad intervention group. These findings are drawn from a mixture of first, second and third order constructs.

The analysis is broken down across the five broad intervention group: rights information provision; performance information provision; citizen feedback and monitoring; participatory planning; and community‐based natural resource management (CBNRM). For each intervention group, the analysis identifies the key moderators (barriers and enablers) that explain the mechanisms triggered by the interventions along their causal chains. Each sub‐section first presents an overview of the included primary studies and corresponding additional literature that were used for the analysis, then iterates a series of case comparisons that highlight the key explanatory factors identified through the synthesis process. Finally, the revised framework for each broad intervention group is presented as a refined theory of change articulating the primary mechanisms connecting the intervention to outcomes along the causal chain. Note that certain factors are important to all included intervention types; to avoid repetition, each factor is only discussed in‐depth through case comparisons once, though included in all refined frameworks as relevant. The extent to which certain factors are generalizable across all intervention types and those unique to specific contexts are discussed in section 5. This section concludes with a section that integrates the framework synthesis with the meta‐analysis, empirically testing the strongest moderating variables that emerged from the qualitative synthesis.

### 8.1 Rights information provision

Five studies comprised or included study arms of interventions that aimed to improve citizens’ access to information about their rights to services (Table 17).

**Table 17:** Included studies of rights information provision

First author	Year	Country	Sector and specific intervention	Additional literature included
Banerjee	2018	Indonesia	Information cards with rice subsidy rights and prices	2 (previous evaluation versions)
Kassim	2016	Pakistan	Information on government reforms	N/A
Olken	2007	Indonesia	Invitations to public construction monitoring meetings (“invitations” study arm)	1 (implementation report)
Pandey	2007	India	Health services presentation	N/A
Ravallion	2013	India	Video campaign of rights to guaranteed labour scheme	2 (qualitative and quantitative studies)

These studies look at the provision of information on rights to services that cover both merit goods (such as rice subsidies) and public goods (such as construction monitoring). Through providing citizens with information on their rights to services, including both entitlements to both quality and quantity, these interventions aim to increase their realisation of their rights. The data extracted in the qualitative synthesis for these studies were reviewed to identify patterns of movements along the causal chain. Within this intervention group, three key factors emerged that helped explain the heterogeneity of results: whether the bottleneck to service access was correctly identified as demand‐driven lack of information; whether the intervention targeted a collective or individual good; and whether the bottleneck was due to demand‐driven lack of information about existing services or supply‐driven rationing of service allocation or corruption. Case comparisons using the included studies are provided to illustrate the importance of these factors.

The first stage in the causal chain thus assumes that the underlying bottleneck to citizens’ access to services is a lack of information about their rights. However, few studies provided ex‐ante evidence that the key barrier to service access was lack of information. Olken (2007) is an exception; in explaining the design of the intervention, the researchers provided qualitative evidence that suggested that the barrier to citizen participation in construction monitoring meetings was due to the lack of written invitations. In the Indonesian cultural context, it was viewed as inappropriate to attend an event to which one had not been invited, and thus the public meetings were primarily attended by a few elite villagers. During the evaluation, this assumption was tested, and the researchers found some evidence that supported their identification of the barrier: following the intervention disseminating invitation cards, the number of non‐elite villagers present at the meetings increased by 75 per cent (Olken, 2007).

In comparison, in Ravallion et al. (2013), though the researchers conducted qualitative research during the design phase to ensure their video would be salient to the rural, poor population targeted, and identified low levels of knowledge of their rights to the labour subsidy service, the intervention ultimately had limited impacts on use of the rural guaranteed labour scheme amongst the targeted population. Subsequent research of the jobs programme suggests that the key barrier to citizens’ access to the labour programme was actually rationing of access to jobs by administrators, triggering discouragement amongst potential workers (Narayanan, Das, Liu, & Barrett, 2017).

A key theme throughout the transparency and accountability‐related studies is the difference in mechanisms triggered by interventions depending on the nature of the service they were targeting, which related to how citizens accessed the service. Broadly, the services could be split into two groups: “direct delivery” services and “indirect delivery” services. The first, “direct delivery,” refers to those services that citizens access from individual service providers, such as the healthcare one receives from a clinician or the food subsidies one collects from the distributor. In these cases, citizens engage with the service provider staff on a regular basis as part of their normal service use. The second, “indirect delivery,” refers to services that citizens access independently of the providers, such as public infrastructure that one uses without engaging with the contractors who built it. In this latter group, citizen engagement in service delivery tends to be limited to transparency/accountability interactions; in the absence of such processes, citizens may not otherwise interact with the providers at all.

Where the intervention targets a directly delivered service, such as the provision of rice subsidies, and the bottleneck is correctly identified as pertaining to lack of information on the demand‐side, then the provision of information may suffice to improve the delivery of services to citizens. In Indonesia, Banerjee et al. present evidence suggesting that disseminating cards with information on citizens’ rights to rice subsidies and standard costs was sufficient to change citizens’ bargaining power with the service provider to increase the amount of subsidised rice they received (2018). The authors highlight facilitating factors that triggered a significant change in response to a relatively small intervention, including:

- The *salience of the information* provided: rice is a staple of the Indonesian government, and the subsidised rice is significantly cheaper than the market rate yet doesn’t cover their full monthly consumption; thus, citizens are highly motivated to attempt to access as much as possible;
- The *creation of perceptions of common knowledge* of eligibility to and costs of the subsidised rice, through the public campaigns in a sub‐set of the treatment;
- The *appropriateness of the strength of the social sanctions* risk: The provision of information regarding rights to services is a relatively weak instrument for changing the balance of power between service providers and service users. However, Banerjee et al. argue that it was effective in the case of the rice subsidies because it created a small shift in citizens’ bargaining power without eradicating the service providers’ control completely over allocation of resources. In their context, this was important because the central government relied on the cooperation of the local village officials for the dissemination of the service; without their cooperation, it would be difficult to implement the project in their villages, and the authors present qualitative evidence suggesting that government officials were cautious of sanctioning incomplete compliance too forcefully (Banerjee et al., 2018).

Conversely, in the case of indirectly delivered services, the ability of citizens to influence service providers appears much weaker. In Olken (2007), though the bottleneck was likely correctly identified as described above, and an increase in participation suggested that communities were motivated to monitor the projects and did not suffer from the free rider problem, the analysis found statistically insignificant results of the intervention on decreasing corruption within the community construction projects. However, he provides evidence that supports the identification of the direct versus indirect delivery mechanism: in the treatment villages, the invitations to participate in monitoring did have an effect on lowering corruption as regards labour costs in construction projects, but not for materials costs. As materials costs comprise the majority of construction budgets, the overall results were insignificant. Yet the community construction projects required voluntary, unpaid labour contributions by community members in addition to providing paid labour opportunities; individual villagers were thus interacting with the contractors to access labour and wages, in addition to participating in the accountability meetings. They were thus highly aware of the real wages and amount of paid labour provided. Conversely, materials were sourced directly amongst contractors with community engagement only through accountability processes, and Olken notes that villagers likely had incomplete information about real costs.

A common element of rights‐information provision interventions is a focus on engaging primarily or solely with demand‐side actors. This thus triggers demand‐driven responses, and may explain the lack of evidence regarding service provider response that led to breaks in the causal chain. However, in cases where service use has a direct effect on wellbeing outcomes, the provision of rights‐information may be able to achieve results further along the causal chain directly through inspiring changes in citizen use of services, despite failing to influence the quality of service provision. For example, in India, Pandey et al. (2007) find that an information campaign on access to health services was successful in increasing citizens’ knowledge of existing services that they could choose to access; unlike the video campaign for the guaranteed labour scheme, service allocation rationing was not an issue. However, though the campaign informed citizens on their rights and how to complain when service delivery didn’t meet quality standards, the authors present qualitative evidence that suggested that the lack of engagement with the supply‐side actors throughout the intervention may have triggered a break in the causal chain for service provider response and service quality improvements (Pandey et al., 2007).

**Figure 28:**
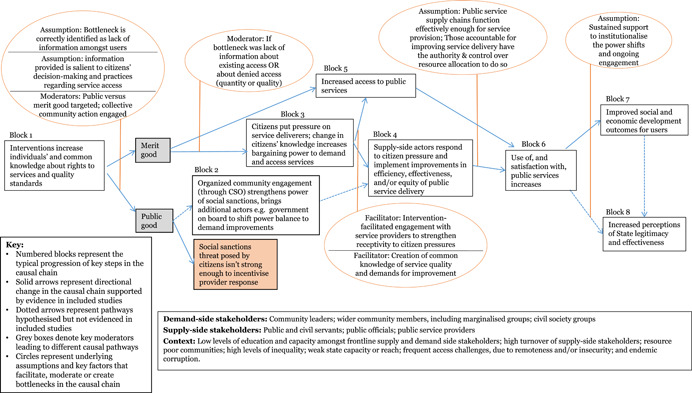
Theory of change for interventions providing information on rights to public service quantity and quality

Following the synthesis process, the original framework was adapted to create a “best fit” framework that highlights the abovementioned key mechanisms and moderating factors (Figure 28). Though the included studies within this intervention group did not include any instances in which an intervention targeting an indirectly delivered service was able to have an effect on service quality through the dissemination of information, the synthesis across the entire sample of included studies in this review identified the strong facilitating capabilities of building social capital and capacity for collective action amongst citizens, such as through working with organised community groups (e.g. local civil society organisations (CSOs) or interest groups) in addressing this bottleneck.Indeed, a subsequent intervention document related to the Olken (2007) experiment noted that a key project lesson had been the success of shifting from implementer‐facilitated monitoring to forming and training groups of community monitors for the construction projects (World Bank, 2011). In the refined theory of change for this intervention group, we thus include the potential of CSO engagement to overcome the indirect delivery bottleneck.

In the diagram, the dotted lines denote the lack of evidence in the included studies. Grey boxes denote key moderators that trigger different mechanisms, leading to slightly different causal change pathways.

### 8.2 Performance information provision

Six studies comprised or included study arms of interventions that improved citizens’ access to information about the performance of public service providers (Table 18).

**Table 18:** Included studies of performance information provision

First author	Year	Country	Sector and specific intervention	Additional literature included
Banerjee	2014	India	Police community observers	N/A
Capuno	2010	Philippines	Dissemination of municipal scorecards	N/A
Fiala	2017	Uganda	Dissemination of scorecards of CDD projects (“scorecard” study arm)	2 (implementation reports)
Grossman	2018	Uganda	Intensive dissemination of district councillor scorecards	2 (implementation reports)
Humphreys	2012	Uganda	Intensive dissemination of scorecards of Members of Parliament (MP) performance	1 (previous evaluation version)
Timmons	2015	Brazil	Publication of municipal audit reports	N/A

These studies include interventions that provided performance information about both individual service providers in the form of elected politicians (Capuno & Garcia, 2010; Grossman & Michelitch, 2018; Humphreys and Weinstein, 2012) and service provider institutions (Timmons & Garfias, 2015; Banerjee et al., 2014; Fiala and Premand, 2017). Through providing citizens with performance information, these interventions aim to trigger mechanisms in which service providers respond to a change in motivated citizens’ efforts to hold them accountable to performance improvements.

The data extracted in the qualitative synthesis for these studies were reviewed to identify patterns of movements along the causal chain. Within this intervention group, four key factors emerged that helped explain the heterogeneity of results: for interventions targeting elected politicians, the relative competitiveness of their constituency and the timing of the intervention in relation to the next election; for all types, the extent to which targeted supply‐side actors accept the intervention (buy‐in); and whether the information provided changes citizens’ priors. Case comparisons using the included studies are provided to illustrate the influences of these factors.

As noted above, this sub‐sample of studies includes interventions that disseminate performance information about individual elected politicians and about service provider institutions. Within the first group, Humphreys & Weinstein (2012) and Grossman & Michelitch (2018) measure the impact of intensive dissemination of performance information against a comparatively weak dissemination, whereas Capuno & Garcia (2010) measure the impact of providing performance information versus its absence. In the second group, Timmons & Garfias (2015) measures the impact of providing performance information in the form of audits related to municipal governments, and thus is still impacted by the electoral factors. Fiala and Premand (2017) include a study arm that provides scorecards to communities with the overall and relative performance of their local community‐driven development (CDD) council regarding the community's chosen project. Banerjee et al. (2014) is the only included study which attempts to evaluate performance information regarding non‐elected service provider performance, specifically the police.

The studies that evaluate the provision of performance information of elected politicians are included in this review because they attempt to make an explicit link between politician performance and service delivery and report the results on service delivery quality accordingly. As noted in the background section, the different spheres of governance interact, and in the case of these studies, the underlying theory is that changes to politician performance can be realised via informal processes of vertical accountability through a “shortened long route” of direct citizen pressures on politicians outside of the electoral cycle, which relies on the threat of immediate social sanctions and future sanctions at the ballot box. While there are many reasons for desiring strong politician performance and accountability to constituents, in this review we focus on the effects of these interventions on service delivery.

The first key moderator identified through the synthesis along the causal chain for politician performance interventions is the influence of competition within an electoral constituency on politicians’ behaviours. This mechanism is specifically tested in Grossman & Michelitch (2018), wherein they find that the intense dissemination of scorecards for politician performance only triggered an improvement in politician performance in electorally competitive constituencies. Grossman & Michelitch (2018) provide contextual information suggesting that in Uganda, while the national‐level politics are dominated by a single party, locally there is variation in relative competition for elected seats, which enabled them to test this mechanism. The findings in Humphreys & Weinstein (2012) support this theory; they find that while voters were strongly receptive to the disseminated performance information, it did not trigger improved performance amongst national‐level MPs, who face minimal electoral competition.

This leads to the next key assumption: that the information provided is salient to citizens’ decision‐making. As noted, Humphreys & Weinstein (2012) found that while the information was salient to citizens’ interests, it did not translate into changes in politicians’ chances for re‐election, thus suggesting that citizens’ electoral decisions were based on factors other than politician performance. Grossman and Michelitch (2018) suggest that the salience of performance information to voters’ decision‐making depends on the political culture; in a context where voting is primarily along party, ethnic or religious lines, politician performance is unlikely to have a large impact on voters’ actions. Given the Ugandan context of limited national‐level electoral competition, this factor could also help explain the null results.

In determining whether the performance information provided is likely to be salient to constituents, the extent to which it changes their priors appears to be influential. This mechanism is tested by Timmons & Garfias (2015), who find that the publication of the results of a municipal government audit influenced the willingness to pay taxes for those constituents whose priors were changed by the audit results. This mechanism may also help explain the dissipation in results over time that Capuno & Garcia observe; in their intervention, performance information was regularly disseminated to constituents over two years, and while the intervention started off by often triggering strong results, by the end of the project the results had weakened or even disappeared in some cases (2010). Drawing on the insights from Timmons & Garfias (2015), this dissipation could potentially be explained as the result of a decrease in the strength of the “shock” provided by the transparency initiative, as citizens and government developed expectations of the results.

Another potential explanatory factor between these two studies is the relative power difference between targeted supply‐side actors (i.e. the politicians) and demand‐side (constituents). It is reasonable to expect that there is a larger power difference between national‐level MPs and their primarily rural constituents, compared to rural constituents and district‐level councillors. Thus, in the absence of the potential for electoral sanctions, politicians who enjoy a greater level of power difference compared to their constituents are more able to ignore increased transparency without fear of credible social sanctions.

Timmons & Garfias (2015) present some evidence that suggests that while elections are not the only mechanism at play in determining whether performance information dissemination triggers improvements in performance, the timing of information dissemination relative to elections does have some effect. The authors of all included studies evaluating elected politician performance note that the reactions to the dissemination of performance information for elected politicians are likely to be affected by whether they are up for re‐election and the time until the next election. Grossman & Michelitch (2018) argue that performance information should be disseminated at such a time that the politicians have the scope to improve their performance before the next election, yet not so close to the election that a negative response (e.g. vote buying or intimidation) is potentially triggered.

Even where the information provided is salient to constituents’ decision‐making, the politicians may still manage to subvert the efforts to hold them accountable, either through preventing the dissemination of information or discrediting the messenger and/or the message. Across the included studies, whether this disruption occurred tended to depend on the extent to which the targeted supply‐side actors were engaged in the intervention design; their support or “buy in” for the intervention; and the relative local credibility of the messenger of the performance information compared to the targeted actor or institution.

In Banerjee et al. (2014), the only included study which looked at non‐elected service providers, the break in the causal chain occurred extremely early on, as the actors charged with implementing the intervention were the very ones whose performance was being measured, and they were able to successfully prevent effective implementation. The purpose of the community observer intervention was to increase citizens’ understanding of the police performance and improve their perceptions, and it had been designed at national level, with the engagement of the national police leadership, yet it sought to change behaviours amongst local police chiefs. Without their buy‐in, the implementation of the intervention was extremely poor, as they falsified records or simply ignored the directives (Banerjee et al., 2014). Humphreys & Weinstein (2012) noted cases in which the MPs forcefully blocked the dissemination of performance information within their constituencies. These cases evidence the importance of ensuring buy‐in amongst the supply‐side actors whose behaviours are targeted by the intervention.

In contrast, the intervention evaluated by Capuno & Garcia (2010) actively engaged the local government units (LGUs) in the implementation process, including at times selecting the LGU as the presenter of the performance information to the communities. Similarly, Grossman and Michelitch (2018) present qualitative evidence suggesting that many district councillors supported the scorecard initiative, as it increased competition.

The importance of the local credibility of the messenger can be understood by comparing the results of Capuno & Garcia (2010) with Humphreys & Weinstein (2012). In the latter, the information was developed and disseminated by a national‐level NGO that did not necessarily have strong ties across all of the treatment constituencies. The authors present qualitative evidence from town hall meetings where the MP was effectively able to discredit the information presented by the NGO staff and undermine the message to such an extent that participants in the meetings had a *worse* estimation of their MP's performance compared to comparison groups (Humphreys and Weinstein, 2012). Conversely, in Capuno & Garcia (2010), the information was disseminated through local partners in each municipality, who were engaged in the process of gathering and analysing the performance data as well. In some cases, the researchers actually worked through the LGUs to present the data, yet even in those where the local partner presented the results, the local partners’ strong ties to the community reduced the politicians’ ability to “shoot the messenger” (Capuno & Garcia, 2010).

**Figure 29:**
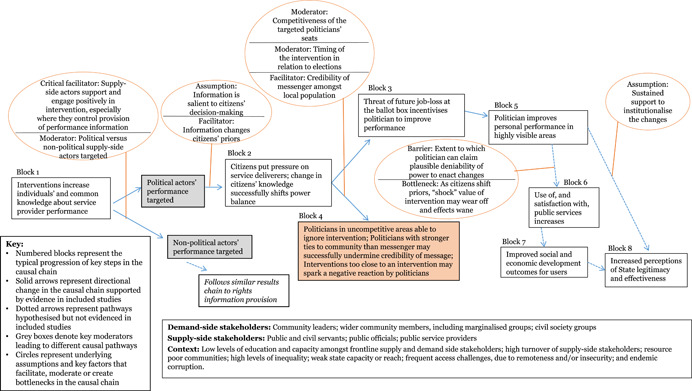
Theory of change for interventions providing information on individual and institutional service provider performance

Incorporating these insights into the framework, the following refined theory of change presents an improved “fit” framework for performance information interventions (Figure 29). While the only included study to investigate performance information dissemination on service delivery through non‐elected actors failed at the first stage of the causal chain, as described above, we nonetheless suspect that should the support of targeted service providers be secured for an intervention, the causal chain for these interventions would likely mimic that of rights information provision. Note that the results chain from interventions targeting elected politicians through to service delivery is quite long. The final barrier to move from changes in politician performance to improvements in service delivery was not reached in any of the included studies. Grossman and Michelitch (2018) suggest that this may be because improvements in service delivery cannot be the result of changes to a single actor (the politician); rather, they rely on multiple actors who may have limited to no direct accountability to the targeted politician (2018). This suggests the relative weakness of interventions that aim to affect service delivery through changes to politician performance.

### 8.3 Citizen feedback and monitoring

Ten studies covered interventions that created or strengthened citizens’ access to feedback and monitoring processes for public services (Table 19).

**Table 19:** Studies included in analysis of citizen feedback and monitoring

First author	Year	Country	Sector and specific intervention	Additional literature included
Alhassan	2016	Ghana	Collaborative community‐based monitoring (CBM) + community assessment of health service performance	2 (qualitative studies)
Berman	2017	Afghanistan	Technical training for community monitors + facilitated accountability meetings for infrastructure projects (roads)	1 (qualitative case studies)
Björkman	2017	Uganda	Collaborative CBM of health services (two arms:(1) CBM only; (2) CBM + externally‐generated performance information)	N/A
Bradley	2005	Kenya and Guinea	Healthcare services feedback loops	1 (working paper)
Fiala	2017	Uganda	Technical training for community monitors (two arms: (1) CBM only; (2) CBM + externally‐generated performance information)	2 (implementation and completion reports)
Grossman	2017	Uganda	SMS‐based anonymous feedback on public services	N/A
Gullo	2017	Malawi	Collaborative CBM of health services + participatory performance measurement	2 (implementation report + synthesis document)
Molina	2014	Colombia	Public construction monitoring meetings (“citizen audits”)	N/A
Olken	2007	Indonesia	Anonymous feedback and invitations to public construction monitoring meetings (invites + feedback group)	1 (implementation report)
Palladium	2015	DRC	Community forums, scorecards and other engagement with security services	1 (implementation report)

This intervention group included the largest sample of included studies, though there were key differences in the intervention components that influenced the causal chains, particularly related to the nature of the public service that was targeted. Of the included interventions, four targeted healthcare, a directly delivered service, three targeted infrastructure, an indirectly delivered service, one targeted the security services, and two targeted a mixture of services. Regarding the nature of the intervention approach, two studies offered only community feedback opportunities: Grossman et al. (2017) and Bradley and Igras (2005).

The rest comprised a version of community‐based monitoring, yet differed as to whether the monitoring comprised a collaborative process engaging both citizens and service providers or provided support only to citizens; whether the accountability or “interface” meetings between providers and citizens were facilitated; whether performance information was provided, and if so, if it was generated by the community or provided by external researchers; and whether technical training on monitoring for the particular service was provided to communities. The ramifications of these differences are discussed in depth below.

The framework synthesis identified two key moderating factors that influenced the causal chain and five common facilitators. Moderating factors included: 1) the type of service targeted, as above, and whether for indirectly delivered services, some additional support was provided to shift the power difference between service providers and citizens, either through well‐respected civil society or government engagement; and 2) collaborative versus confrontational approaches. The common facilitators included the provision of technical monitoring skills; access to contracts and other key information; the inclusion of provider performance information; the incorporation of a dedicated community monitoring group; and the creation of common knowledge of provider performance.

As with the other accountability and transparency‐for‐accountability interventions, the nature of the service being targeted appeared to be a key moderating factor within the causal chains. Alongside the indirectly delivered services, the intervention evaluated by Grossman et al. (2017) followed a similar causal chain, as the SMS‐based anonymous feedback intervention aimed to encourage citizens to complain to government officials regarding public services, and thus, the indirect accountability relationship between citizen and frontline service provider was mirrored. Conversely, the other study comprising a mixture of service types, Fiala and Premand (2017), was implemented in the context of a national CDD programme; in each community, only a single project prioritised and implemented through the CDD programme was targeted, such that while the nature of the services varied across communities, it was constant within each community.

The studies of indirectly delivered infrastructure projects demonstrate the key role external support to the community can play in overcoming the comparatively weaker social sanctions that are posed by communities monitoring indirectly delivered services. Similarly to Olken (2007), the interventions evaluated in Molina (2014) and Grossman (2017), both of which rely on engagement with unorganised citizens, were unable to realise significant improvements in public service delivery, despite achievements in triggering citizen engagement with the respective platforms. Conversely, even in a challenging context such as the DRC, in Palladium (2015), the implementer's work with local civil society led to greater‐than‐expected project success in organising and hosting well‐attended community fora to encourage citizen engagement with the security sector. The evaluation presented qualitative evidence that suggested that participation in these fora had positively impacted people's perceptions of security and the security sector (Palladium, 2015). The role of civil society support to communities may be critical not only for encouraging engagement in monitoring and accountability processes, but also for shifting the balance of power between citizens and public service providers of indirectly delivered services. In Berman et al. (2017), the authors present evidence from qualitative research to test the underlying mechanisms, which found that the active engagement of the large, well‐respected national‐level NGO Integrity Watch Afghanistan (IWA) in the construction monitoring intervention was critical to the project's success. The social capital provided by IWA enabled the community monitors to access the critical information they needed to monitor the road construction, such as contracts; brought key stakeholders to the table to discuss issues in Provincial Monitoring Board meetings, including local leaders, government officials, contractors and community monitors; and thus increased the bargaining power of community monitors, enabling them to often enforce improvements before escalating the situation by complaining to the government. This theory is supported by the quantitative evidence, which showed the dissipation of the positive effects of the project after IWA ended its direct engagement in the intervention.

The creation of common knowledge amongst the community of the monitoring results further emerged as a strong facilitating factor. Two interventions incorporated the provision of anonymous feedback: Olken (2007), which consisted of invitations to monitoring meetings + anonymous feedback cards; and Grossman et al. (2017), the uBridge SMS programme to increase engagement between constituents and local government. In the former, consolidated anonymous feedback forms were read out at the open meetings, which the author argues created a common knowledge amongst participants as to the common nature of people's complaints, which had a small positive impact on their ability to trigger sanction measures (Olken, 2007). Conversely, in Grossman et al., 2017, though many messages were sent by constituents commenting on the quality of service delivery, common knowledge was not created, as the content of the messages was not public. This prevented the citizens from using the intervention to identify like‐minded compatriots, build social capital and undertake collective action that might have increased the relative strength of their pressure on service delivery. This suggests a potential explanation for the break in the causal chain for this intervention.

Similarly, in analysing citizen audits of construction projects in Colombia, Molina presents evidence that suggests that low participation in monitoring opportunities prevented the creation of common knowledge about the projects, which in turn discouraged politicians and service providers from adhering to quality standards, which he refers to as the “self‐fulfilling prophecy” phenomenon (2014). However, in Fiala and Premand (2017), the authors report no significant change in numbers of community members engaged in monitoring following the intervention, despite seeing positive results; what changed was the capacity of the group monitoring the projects to carry out their mandate, and the creation of common knowledge of the monitoring results through intervention‐led activities such as the scorecard presentation. Berman et al. (2017) present similar findings, including qualitative evidence of “social shaming” initiatives undertaken by the monitors, such as partnering with the local mullah to announce the monitoring findings (good and bad) during sermons. The qualitative evidence further stresses the importance of the technical training to enable the monitors to effectively identify whether the construction was of sufficient quality or not (Berman et al., 2017); such technical training was absent from the intervention studied in Molina (2014). Thus, it may be that a dedicated monitoring group, with a mandate from the community and technical training in monitoring the service targeted, could have a greater impact than an open‐forum type of intervention as in Molina (2014) and Olken (2007), and as noted above, the intervention studied in Olken (2007) ultimately adopted the approach of establishing and training a dedicated group of community monitors (World Bank, 2011).

Amongst the sample of community feedback and monitoring interventions, a unique feature of those targeting healthcare services was a focus on a collaborative process that engaged both supply and demand‐side actors, i.e. both community members and frontline health centre staff. This set the group apart from the other interventions, which focused on training and/or creating opportunities for citizens to hold providers accountable through dedicated accountability meetings. This included both public Town‐Hall style meetings, as in Molina (2014), Olken (2007) and Palladium (2015), and higher‐level fora such as the Provincial Monitoring Board meetings in Berman et al. (2017).These meetings are often more confrontational than in the phased, collaborative approach, wherein the implementers guide communities and service providers through a series of three types of meetings: citizen meetings, to build capacity for monitoring and ensure understanding of rights; service provider meetings, to present the emerging findings of the citizen meeting and begin planning for ways to address the highlighted issues; and an interface meeting, during which the community's priorities and ideas for improvements are incorporated into the relevant service delivery plan, with a focus on assigning responsibilities amongst both community members and service provider staff to address areas in need of improvement. These interventions can be adjusted to include an explicit inclusivity component to improve the engagement of vulnerable groups along the causal chain. In the evaluation of CARE's Community Scorecards by Gullo et al. (2017) and in Björkman Nyqvist et al. (2017), a series of community meetings were held with different interest groups, including women, youth, the disabled, and the elderly. This ensured that views from across the community are fully captured. However, the approach relies in significant extent on the capacity of implementer staff and their facilitation skills.

To attempt to explain the black box of intervention and outcome, Alhassan et al. tested the underlying mechanisms for service provider motivation, and found that the service providers working in rural health clinics were highly intrinsically motivated, and through the collaborative engagement with the community, increased their intrinsic motivation (2016). This suggests that monitoring interventions that rely on the “soft” power of social sanctions may be more effective when they focus on identifying mutually empowering “win‐win” opportunities and ways for citizens and service providers to work together. This theory is also supported by qualitative evidence presented in Bradley and Igras (2005), wherein healthcare staff reported that the empowering process of local problem identification and solving had a strong impact on their attitudes, and led to changes in the way they engaged with each other and with community members. The increased sense of self‐efficacy built through this type of approach may extend to the community members, who see the responses to their efforts enacted by service providers, as suggested by Gullo et al. (2017).

The relationship between service providers and users may also be strengthened through the facilitated, collaborative approach because while learning about their service entitlements and identifying opportunities for improvement, citizens also learn more of the intricacies and challenges in service delivery, which may enable them to mitigate their expectations and be more understanding of the frontline staff. Gullo et al. (2017) suggest that the more realistic expectations held by households in treatment communities may account for their increased satisfaction with the health services, despite the context in which there were serious issues in health service supply chains due to a national‐level scandal, which led to decreasing satisfaction with health services in control communities.

**Figure 30:**
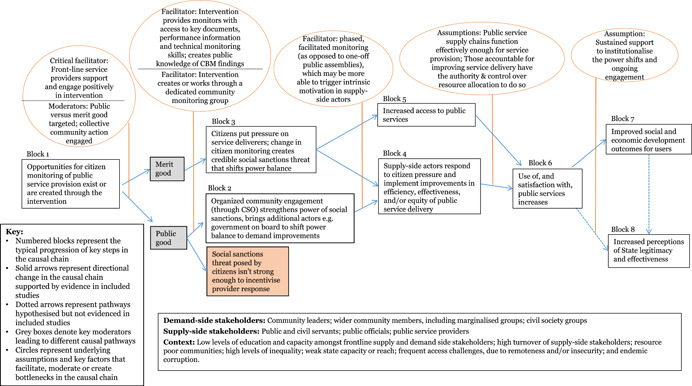
Theory of change for citizen monitoring and feedback interventions

A final key facilitator in community monitoring interventions is the benefits wrought by including performance measurement information into the intervention. In Björkman Nyqvist et al. (2017) this was done by external researchers and research assistants, who gathered the data and presented it to communities in a digestible and locally appropriate way. This was a very thorough approach, but it has made replication challenging, an issue the authors identify (Björkman Nyqvist et al. 2017). In Alhassan et al. (2016), the implementers worked with the community groups to support them to undertake the performance assessment, which they then used to identify the key opportunities for improvement. CARE's Community Scorecard methodology takes this further, working with communities to create a localised scorecard in which communities develop their own list of priorities and indicators (Gullo et al., 2017). In comparing their two treatment arms, wherein the difference was access to performance information, Björkman Nyqvist et al. (2017) present evidence suggesting that having information on performance and benchmarks was critical for enabling communities to identify realistic opportunities for service improvements. Conversely, in Palladium (2015), which didn’t include any performance information, though perceptions of security rose amongst participants, the study did not find evidence of improved service delivery outcomes, and conclude that changes in perceptions may occur more quickly than changes in service delivery (2015). Fiala and Premand, in a study arm comprising only interventions in livestock provision, also find that the inclusion of both community monitoring support and performance information is critical to achieving positive impacts on household assets (2017). Through the framework synthesis, the key mechanisms, barriers and facilitators were collected and used to refine the theory of change for this group of interventions (Figure 30).

### 8.4 Participatory planning

Eight studies evaluated seven interventions or policies that created or strengthened citizens’ access to participatory planning processes (Table 20) – note that while Touchton & Wampler, 2014 and Gonçalves, 2013 are separate studies, they are of the same countrywide policy.

**Table 20:** Studies included in analysis of citizen engagement in planning

First author	Year	Country	Sector and specific intervention (* if inclusive planning)	Additional literature included
Ananthpur	2014	India	Support to engage in participatory development planning	N/A
Beath	2013	Afghanistan	Mandated women's inclusion in participatory development planning*	2 (qualitative studies)
Beuermann	2014	Russia	Increased facilitation of participatory budgeting	N/A
Diaz‐Cayeros	2014	Mexico	Municipal indigenous participatory governance*	1 (qualitative study)
Giné	2018	Pakistan	Community mobilisation for participatory development planning*	N/A
Gonçalves	2013	Brazil	Municipal participatory budgeting*	5 (qualitative and quantitative studies)
Touchton	2014			
Humphreys	2014	DRC	Mandated women's inclusion in participatory development planning*	4 (implementation reports and qualitative study)

Within this sample, three studies measure the effect of participatory processes against the status quo (Touchton & Wampler, 2014; Gonçalves, 2013; and Diaz‐Cayeros et al., 2014); two studies measure the effect of external support to participatory planning processes (Ananthpur et al., 2014; Beuermann & Amelina, 2014); two studies measure the effect of mandating women's inclusion in participatory planning (Beath et al., 2013; Humphreys et al., 2014); and one study measures the effect of participatory planning training on citizens’ empowerment to demand services (Giné et al., 2018). Grouped differently, five of the studies look at interventions wherein citizens engage in government planning processes (Touchton & Wampler, 2014; Gonçalves, 2013; Ananthpur et al., 2014; Beuermann & Amelina, 2014; and Diaz‐Cayeros et al., 2014), and three of them pertain to interventions wherein citizens are engaged in community‐driven development (CDD) types of deliberations (Beath et al., 2013; Humphreys et al., 2014; and Giné et al., 2018). Through engaging citizens in the identification of priorities and allocation of resources, these interventions aim to improve the responsiveness of service delivery to citizens’ prioritised needs, particularly for vulnerable groups.

The data extracted in the qualitative synthesis for these studies were reviewed to identify patterns of movements along the causal chain. Within this intervention group, four key factors emerged that helped explain the heterogeneity of results: the extent to which the intervention correctly identified and adequately addressed barriers to participation for vulnerable groups; the extent to which the intervention process was designed to encouraged the growth of local social capital and capacity for collective action; the extent to which the local government or decision‐making body supported the process and had the capacity to implement it; and the incorporation of explicit measures to facilitate the inclusion of vulnerable groups. Case comparisons using the included studies are provided to illustrate the importance of these factors.

As noted above, a key goal of participatory planning processes is frequently to ensure the priorities of vulnerable and marginalised members of society are incorporated into decision‐making. As described above in the equity discussion, however, only a minority of the included interventions were described as incorporating specific components to improve the inclusion of vulnerable groups in the activities. The majority of these were incorporated into participatory planning interventions; of the seven interventions in this set, five incorporated specific inclusion components. Barriers and facilitators to inclusive planning is thus a key focus of the framework synthesis. Again, as noted previously, however, studies that incorporated inclusion components generally only focused either on inclusion for the poorest or women's inclusion. Thus, the dataset is limited in its capacity to provide insights into the barriers for vulnerable groups in general, and particularly weak to the barriers and facilitators of including other types of vulnerable groups, such as people with disabilities, refugees or internally displaced persons.

In order to support vulnerable groups to participate, the barriers they face at baseline must be adequately assessed (bottleneck identification) and the intervention designed to address the specific barriers in a culturally appropriate and locally relevant way. The different mechanisms triggered in four of our included studies can help illustrate the trickiness of doing so.

Two of the included studies, Beath et al. (2013) and Humphreys et al. (2014), demonstrate these challenges regarding including women in decision‐making. Humphreys et al. (2012) evaluated the effects of mandating women's participation in the village development councils in DRC, and found no effects; where women's participation was not mandatory, they nonetheless participated in roughly equal numbers. This suggests that the barrier to women's voices being heard in the local context was not the result of them being denied a seat at the table, an example of bottleneck misidentification. Meanwhile, Beath et al. (2013) studied the effect of mandating women's inclusion in food distribution planning. While in the Afghan context, women are frequently denied a seat at the table, the intervention design comprised an externally‐imposed participation requirement that was not adapted to the local context, leading to unintended effects. The authors found that mandating women's participation alongside traditional *jirga* leaders led to an increase in leakages of food aid (Beath et al., 2013). Qualitative evidence in the evaluation suggested that the *jirga* elders were retaining some of the food aid for themselves as compensation for their services in the distribution, and that when women were required to participate, they were generally the wives or relatives of the *jirga* members (*ibid.*). This suggests the possibility that mandating women's participation may have triggered elite families to “double dip” into the food aid as compensation for the services of both their male and female representatives. This demonstrates how nuanced an understanding of local practices is required in selecting an appropriate intervention to address an identified barrier.

Understanding and adequately addressing the power gap between “status quo” participants in decision‐making processes and those excluded may be key to addressing participation barriers. In Giné et al. (2018), communities that received community‐driven development training were evaluated to ascertain the effects of this sector‐non‐specific training on citizens’ capacity to demand public service provision. The training covered elements of participatory development planning, and communities were organised and mobilised to prepare for project implementation. They found that the intervention had a significant effect on the provision of health services by the local “Lady Health Workers” (LHWs), which they attribute to the growth in collective action capabilities amongst women participants, who had indicated at baseline that healthcare was a priority concern (Giné et al., 2018). However, LHWs are local women from the village in a conservative area wherein women are frequently disempowered; the relative difference in power, therefore, between the LHWs and the other villagers is extremely small. Thus, in this context, an intervention that was designed to be empowering but did not specifically address people's capacity to demand health services nonetheless had an effect, given that the women had indicated that health was their priority area of focus and the relative power difference between village women and LHWs was minimal. It is telling that the study found limited to no effects on health services at the health centre level (*ibid.*). This is in stark contrast to the experiences documented through qualitative research in Ananthpur et al. (2014), in which the members of the local elite at times actively attempted to dissuade or prevent villagers from participating in the decision‐making processes. The ethnographic component of the study identified remnants of a feudal relationship between villagers and local elites; elites thus capitalised on this larger and entrenched power difference to stifle participation.

Incorporating into intervention design flexibility to enable communities to adapt the activities to their local context may be key to avoiding such shortcomings. In Brazil, participatory budgeting was designed as a pro‐poor intervention at the national level, yet the specifics for how municipalities went about ensuring participation was left to them to decide. Thus, the extent to which measures were put in place to actively include vulnerable populations varied by municipality. While all incorporated pro‐poor measures, in at least one case, specific mechanisms were created to facilitate the participation of historically marginalised groups such as LGBT citizens (Hernandez‐Medina, 2010). Though the included impact evaluations of this policy do not present outcomes data disaggregated by vulnerable groups, evidence from participant interviews in a qualitative study of the policy suggested that the explicit measures adopted by some municipalities were critical to opening up the process to diverse disadvantaged groups (*ibid*.).

In Diaz‐Cayeros et al. (2014), the authors note mixed effects of the intervention on women's participation in local governance. On the one hand, quantitative evidence suggested that the switch from political‐party based to traditional governance systems led to a decrease in the number of women in senior municipal government positions, yet the authors also found qualitative evidence that women's participation in traditional governance processes was slowly increasing (Diaz‐Cayeros et al., 2014). In this last case, the intervention (the shift to traditional governance) was not imposed by an external party but rather chosen by the community. While the externally‐imposed processes evaluated in Humphreys et al. (2014) and Beath et al. (2013) misidentified the local barrier and appropriate response, respectively, to ensuring women's inclusion, and thus do not enable a comparison of the value of incorporating explicit measures to address inclusion barriers, the qualitative evidence noted above from Brazil suggests that explicit measures may be required to support the engagement of vulnerable groups in processes in which they have been historically excluded. Though the intervention in Mexico increased participation across the community as a whole (by making it mandatory), the lack of complementary measures to support women's and other groups’ empowered participation may have led to the initial declines in women's leadership evidenced in the evaluation.

The framework synthesis of the data suggests that the capacity of these interventions to empower communities to participate in local planning processes (i.e. to reach the first block of the causal chain) could be strongly facilitated through designs that encouraged the growth of local social capital and capacity for collective action. This theme emerges as a key mechanism for changing the balance of power between targeted actors on the supply and demand sides. In Brazil, the design of the participatory planning policy explicitly sought to incentivise collective action through engagement with the planning process, by encouraging citizens to create coalitions in support of their favoured priorities, which stimulated the growth of local civil society (Touchton & Wampler, 2014). This success is also due to the Brazilian context, characterised by lower initial barriers to participation for marginalised citizens and historically strong civil society, and the long timeframe of the intervention and evaluation follow‐up, uniquely long amongst this group of interventions.

In comparison, the experience in India studied by Ananthpur et al. (2014) was very different: in this intervention, pairs of facilitators were trained and dispatched to the intervention areas to attempt to support the implementation of the community meetings and engagement with the Gram Panchayat (local village council). By relying on the individual capacity of two consultants in each area, this intervention failed to generate social capital and capacity for collective action amongst the targeted communities, and the balance of power between villagers and local elites was not challenged.

Ensuring buy‐in from the government or community decision‐making body for implementing participatory planning processes may be critical to their success, and opt‐in style policies may strongly facilitate such buy‐in. In Brazil, municipal governments choose whether or not to adopt participatory budgeting, ensuring strong government support for the process. Conversely, in Russia, the reforms were passed at national level and implemented across the country, without the flexibility for “settlements” to choose whether or not to adopt the policy (Beuermann & Amelina, 2014). Similarly, in India, Ananthpur et al. find qualitative evidence that suggests that local elites worked hard to inhibit the participatory nature of the intervention, as it jeopardised their control over development resources (2014).

The final critical barrier to successful implementation of participatory planning processes identified through the synthesis was the importance of ensuring that the government or community decision‐making body had the capacity to implement the participatory planning process. In the case of Mexico, Diaz‐Cayeros et al. documented the return to “traditional” governance for indigenous communities in an impoverished state (2014). Thus, the intervention was implemented in a context in which there were strong local capacities and traditions of engaging in such processes. In contrast, Beuermann & Amelina found that newly established “settlement”‐level governments tasked with implementing participatory budgeting were saturated with attempting to establish and learn how to run their governments in general (2014); alongside everything else they were trying to learn, participatory budgeting fell by the wayside. This bottleneck further highlights the importance of timing in an intervention.

**Figure 31:**
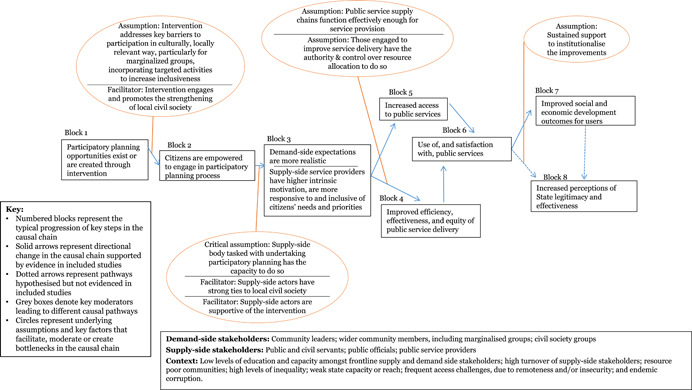
Theory of change for participatory planning and priority setting interventions

Incorporating these insights into the framework, the following refined theory of change presents an improved “fit” framework for performance information interventions (Figure 31)**.**

### 8.5 Community‐based natural resource management

Seven studies covered interventions that created or strengthened citizens’ capacity to manage full or close‐to‐full decentralisation of service delivery (Table 21).

**Table 21:** Studies examining CBNRM

First author	Year	Country	Sector and specific intervention	Additional literature included
Bandyopadhyay	2004	Namibia	Community wildlife conservancies	3 (qualitative studies and policy paper)
Bandyopadhyay	2010	Philippines	Irrigation management transfer to Irrigation Associations	1 (qualitative study)
Barde	2017	Brazil	Water User Associations	N/A
Huang	2014	China	Water User Associations	2 (qualitative studies)
Persha	2016	Tanzania	Joint forestry management	2 (policy document and implementation report)
Rasolofoson	2015	Madagascar	Community‐based forestry management	2 (previous evaluation versions)
Tachibana	2009	Nepal	Community‐based forestry management	5 (qualitative and quantitative studies)

The included studies in this intervention are quite different from those in the previous groups, as the service provision has been decentralised to such an extent that communities themselves are both the user and the provider. This fundamentally shifts the power dynamics at play, complicating the delineation between supply‐side and demand‐side actors. Community‐based natural resource management (CBNRM) interventions aim to improve communities’ sustainable access to resources through increasing their control over resource management and maintenance. The complexities and tensions involved in marrying the dual goals of resource use and preservation are evident throughout the interventions, which cover wildlife conservancy (Bandyopadhyay et al. 2004); participatory forestry management (Persha & Meshack 2016; Rasolofoson et al. 2015; and Tachibana & Adhikari 2009); and irrigation or water use (Bandyopadhyay et al. 2010; Barde 2017; and Huang 2014). Each of these studies evaluates the implementation of a national‐level policy, which tend to have smaller results than pilots or experiments wherein the quality and uniformity of implementation is more easily managed.

A key moderator identified early in the causal chain for these interventions is the extent to which the policy constitutes a relinquishment of government control over the productive resource. For example, in Nepal, the community forestry project studied by Tachibana & Adhikari primarily represents a formalisation and standardisation of existing practices; the government remained only lightly involved in forestry management (2009). Conversely, in Tanzania, the Joint Forestry Management (JFM) intervention requires a more engaged and active partnership between government forestry officials and communities, which has proven more challenging to implement (Persha & Meshack, 2016).

Building on this moderator, where the government is required to give up some of its control over the benefits from the productive resource through the CBNRM intervention, there is often a barrier wherein local officials choose not to fully implement the requirements of the policy or seek to undermine its promise of transferring resource benefits to communities. This tends to happen after the devolution of resource management responsibilities to the community, but before communities’ rights to benefits are formalised, as in Persha & Meshack's study, wherein they note that only seven per cent of targeted communities had signed joint forestry agreements with the government, and present evidence to suggest that this barrier led to a break in the causal chain that inhibited communities’ ability to realise the economic benefits of JFM (2016). In a second example, evaluating the implementation of irrigation management transfer (IMT) in the Philippines, Bandyopadhyay et al. present qualitative evidence that suggested the government water agency was withholding fees from the community associations; this risk was further evidenced in a qualitative study whose findings suggested that the government water agency only agreed to IMT in order to reduce its operating costs (Bedore 2011). This is a serious risk of CBNRM projects, as it may leave communities shouldering more of the burden of resource management without enjoying the benefits; in contexts where most communities are resource‐ and time‐poor, this cost can be substantial.

The likelihood of incomplete implementation for national‐level policies is compounded when the policies are not clearly specified, aligned with other key laws and regulations, or especially when contradictory to them. This was found to be the case in Rasolofoson et al. (2015), wherein the researchers conducted in‐depth analysis of the myriad national policies, laws and regulations pertaining to natural resource management, and identified a number of inconsistencies and contradictions that helped explain the lack of impact on outcomes found in the statistical analysis. These inconsistencies and contradictions are vulnerable to exploitation by supply‐side actors intent on retaining access to their benefits; Rasolofoson et al. present qualitative data suggesting that local officials selected a mixture of the policies that best suited their interests, rather than the interests of communities (2015). This sensitivity to capture by government officials substantially decreases the potential benefits communities may realise through CBNRM.

The success of CBNRM further depends on the type of resource use in which communities engage, and their capacity to enforce the rules. In a qualitative study of the community conservancies evaluated by Bandyopadhyay et al. (2004), participants highlighted the challenge of preventing poaching in areas frequented by migrants (Jones, 1999). Such high‐stakes monitoring may be beyond the capacity of communities to enforce, particularly without resorting to violence. Conversely, Tachibana & Adhikari suggest that the primarily light resource use in the Middle Hills of Nepal (collecting leaves and sticks for kindling) was more conducive to CBNRM; the authors interpret their findings to suggest that forests where logging is common may be more of a challenge for the CBNRM model (2015).

The provision of alternative livelihoods support is vital in areas where communities’ traditional access to the resource is restricted as a result of the implementation of the conservation component of CBNRM. While this speaks to the tension between human quality of life outcomes versus environmental outcomes, various studies identified the potential to overcome this barrier through support for alternative livelihood means and practices. Further, analysis by Barde of community‐based water management in Brazil suggested that CBNRM groups were effective at improving outcomes for communities because they had a higher level of downwards accountability to their communities (2017).

The synthesis of included studies and additional texts suggests that key factors for success in CBNRM interventions may rest on full legalisation of the communities’ ownership of resource benefits (Persha & Meshack, 2016); the injection of donor funds to catalyse the change in resource use (Barnes, MacGregor, & Weaver, 2002); sustained external support to enable the community groups to institutionalise slowly over years (Jones, 1999); and the presence of tourism opportunities for communities to undertake alternative livelihoods (Barnes et al., 2002; Persha & Meshack, 2016).

**Figure 32:**
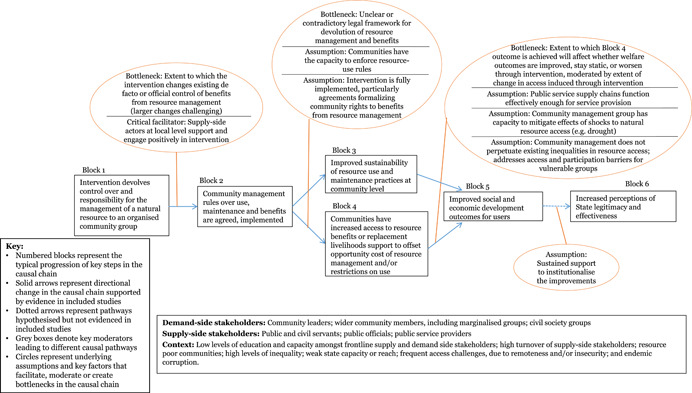
Theory of change for community‐based natural resource management interventions

As a result of the synthesis process, the theory of change was refined for CBNRM interventions. While the causal chain appears relatively linear, the large number of moderators, assumptions and identified barriers and bottlenecks, combined with the often weak results from the evaluations, suggests that these interventions are extremely tricky to carry out at national scale (Figure 32).

### 8.6 Common cross‐cutting factors and integrated synthesis

Many interventions experienced challenges stemming from a lack of positive engagement with supply‐side actors at the intervention target level, whose relative power the interventions often sought to diminish. Interventions implemented within the good governance domain of external engagement generally operate within a context of an imbalance of power in favour of the service provider, who controls the quality of and access to resources and services. Interventions that seek to change this balance of power without engagement with and buy‐in from these actors may trigger response mechanisms in which the service providers attempt to block, discredit or co‐opt the intervention to maintain their relative power. For example, Humphreys and Weinstein report evidence that some politicians whose performance scorecards were due to be disseminated successfully blocked implementation of the intervention in their constituencies, threatening violence (2012). Banerjee et al. (2014) identify this triggering of a negative response by the service providers at the targeted level (police station chiefs, in this case, who successfully prevented the implementation of community observers in most areas) as the key mechanism leading to a break in the causal chain. Similarly, Persha and Meshack (2016) and Rasolofoson et al. (2015) present evidence that government forestry staff members are able to exploit lack of clarity in national‐level policies or top‐down enforcement of complete implementation such that the officials are able to maintain their control over the resource benefits despite having devolved the responsibilities of management to the communities. Conversely, interventions that were designed and implemented with the support of key power brokers at the level targeted by the intervention, as in the case of municipal governments that chose to implement participatory budgeting in Brazil (Touchton and Wampler, 2014; Gonçalves, 2013) or structured community engagement in the health sector that aimed to strengthen service providers’ intrinsic motivation (Alhassan et al. 2016), were able to realise positive impacts across the causal chain.

It is important to note that while in the majority of included broad intervention groups, a break in the causal chain at this stage may at best prevent outcomes tied to service provider response or lead to null effects, in the case of community‐based natural resource management there is a risk of causing negative effects on well‐being outcomes. As noted in the Persha and Meshack (2016) and Rasolofoson et al. (2015) cases, this may happen where a lack of full intervention implementation leads to a context in which resource‐ and time‐poor communities increase their burden of natural resource management, have less access to the resource due to sustainability restrictions, and are not afforded adequate compensation in the form of resource benefits ownership or alternative livelihoods support. The risk that an intervention may do harm to a community should be seriously considered during project design, and locally appropriate mitigation measures should be developed to lessen the likelihood of negative impacts.

Building on the above, the findings of this review lend some support to the theory that citizens’ attempts to increase their relative power through means seen as confrontational by service providers often disincentivise the service provider from participating (World Bank 2004). The findings of this review suggest that approaches to citizen‐service provider engagement in the realm of accountability, including transparency for accountability, appear to work more effectively when implemented through phased, facilitated processes that are framed as collaborative, as opposed to one‐off accountability meetings that tend to be interpreted as confrontational. Interventions that promote transparency with the aim of triggering mechanisms that motivate citizens to demand greater accountability often fall closer to the confrontational spectrum, and their limited success on realizing outcomes along the causal chain is evident throughout the included studies. Those that promote an explicitly collaborative process may be more effective, particularly when they incorporate measures to improve citizens’ understanding of performance benchmarks, such as in Björkman and Svensson (2010) and Alhassan et al. (2016). In these two programs, though citizens were provided or supported to gather information on service provider performance quality, respectively, the process of applying that knowledge to service improvements was done in a collaborative way that was mutually empowering, in line with the theory suggested by Fox (2014).

We note, however, a difference between interventions targeting individuals versus service provider institutions, and caution that it may be more difficult to engage in collaborative approaches to performance improvements with individuals, such as politicians, who are understandably more likely to feel personally targeted. In these situations, the synthesis suggests that ensuring the engagement of a locally credible messenger to disseminate performance information reduces the ability of the targeted individual to undermine, co‐opt or discredit the information.

One potential limitation of interventions relying on accountability and transparency‐for‐accountability through community engagement, however, is that while such interventions often met with some success in realising improvements at a local level regarding service delivery quality, there are many service delivery bottlenecks that cannot be dealt with through community engagement. This was a barrier highlighted in Bradley and Igras (2005) and Gullo et al. (2017): in both these evaluations, the authors identified improvements only among indicators that could be addressed without changes in resources or support. This provides some support to an assumption identified in the initial theory of change, which identified a risk that improvements would be limited to those that were within the purview of the service providers targeted for support. Bottlenecks such as issues in service supply chains or those requiring the approval and engagement of more senior management, particularly at provincial level and above, are unlikely to be successfully addressed through community engagement efforts. This reinforces the need for proper bottleneck identification during project design, to ensure the proper tools are applied.

The findings of the framework synthesis suggest a key facilitator for interventions across the external engagement sphere of good governance was the incorporation of active engagement with local organised community groups, such as CSOs or interest groups, or the inclusion of measures that explicitly sought to build local social capital and capacity for collective action. This facilitator was present in each intervention that succeeded in addressing the bottleneck caused by a lack of service provider response in indirectly delivered service provision. For example, in their replication of Björkman and Svensson (2010), Donato and Mosqueira demonstrate the significant contribution of a strong presence of the local CSO partner in the targeted community on achieving positive results (2016). Similarly, in their in‐depth ethnography of a “failed” intervention, Ananthpur et al. present qualitative evidence that suggests that positive results were achieved where the facilitators tasked with supporting the implementation of participatory planning processes were able to build relationships with local citizen groups, particularly women's groups, and work with them to address key issues (2014).

Following the completion of the initial framework synthesis, we added codes to the meta‐analysis data to test the strength of some of the mechanisms identified. We first tested the strength of the influence from the different types of service delivery. Initially, the distinction was theorised to be between pure public goods ‐ services provided by the state which are non‐rival and non‐excludable, e.g. public roads ‐ and merit goods ‐ public services which are rival and excludable, usually because they are provided by front‐line public servants, e.g. health services, or are subject to rationing, e.g. food subsidies. We expected to see stronger results around citizen engagement in merit goods provision, in which accountability to service users is more direct, leading to differential effects on access and possibly use and wellbeing further along the causal chain. Note that this distinction relates only to the three accountability and transparency interventions (rights information, performance information, and community feedback and monitoring); it did not emerge as a strong explanatory factor in participation interventions (participatory planning and CBNRM).

**Figure 33:**
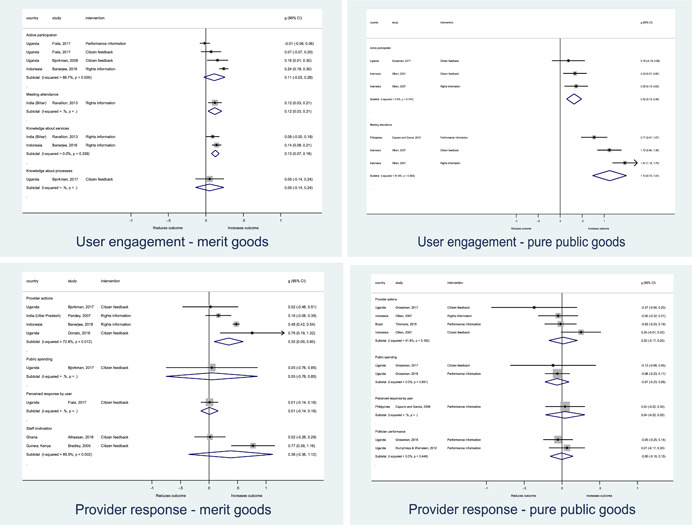
Immediate outcomes for pure public and merit goods

The results of meta‐analysis showing immediate, intermediate and final outcomes are presented below. As Figure 32 demonstrates, the expected difference in citizen engagement for merit versus pure public goods was not identified. This suggests that these interventions do not necessarily suffer from a free‐rider or collective action bottleneck; the interventions were successful in stimulating citizen engagement in feedback and monitoring opportunities whether they are for pure public goods or merit goods. However, the distinction between the two groups of services becomes starker when looking at provider response (Figure 33), where the only outcome that suggests a significance increase is for provider actions (SMD=0.35, 95%CI=0.09, 0.60) and subsequently on changes in service access.

**Figure 34:**
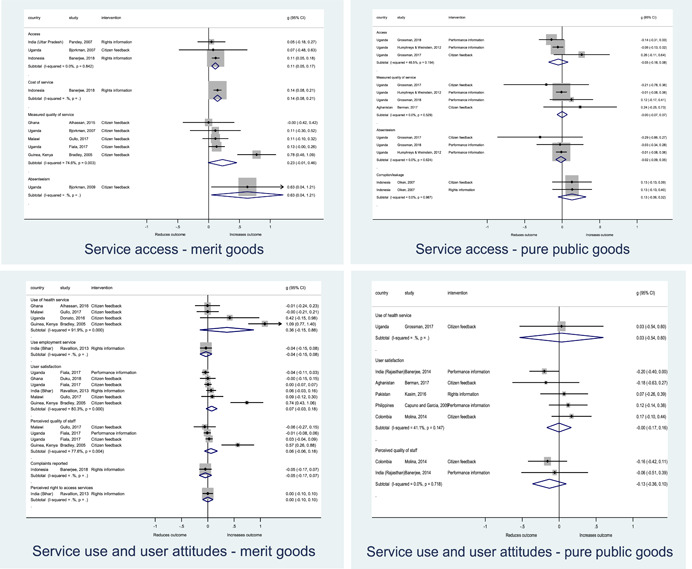
Intermediate outcomes for pure public and merit goods

Figure 34 shows findings for intermediate outcomes, indicating consistent differences between merit goods and pure public goods for service access outcome categories. The findings show positive effects for all outcome sub‐categories (physical access, service cost, quality of service, absenteeism) for merit goods, but insignificant findings for pure public goods outcomes. There are no significant effects for service use variables; pooled effects for merit goods were positive in several cases, including health service use (SMD=0.36, 95%CI=−0.15, 0.88), user satisfaction (SMD=0.07, 95%CI=−0.03, 0.18) and perceived quality of staff (SMD=0.06, 95%CI=−0.06, 0.18), where they were null or negative for health service use, user satisfaction and quality of staff.

**Figure 35:**
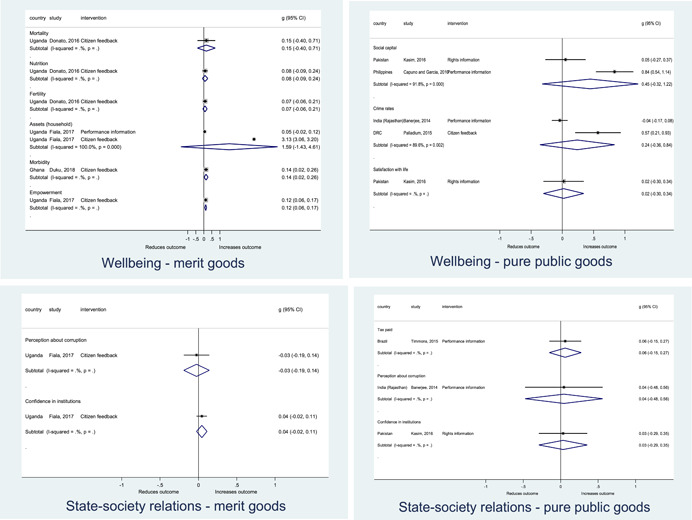
Final outcomes for pure public and merit goods

Figure 35 presents findings for wellbeing and state‐society relations outcomes. No more than a single study measured most outcomes, and the results no not suggest any differences between wellbeing and state‐society relations for merit versus pure public goods.

Based on the results of the integration with the meta‐analysis, we revised the theory, including the theory of change best‐fit frameworks, to hypothesise that the break in the causal chain at provider response for services such as infrastructure or municipal government is more likely to be due to the nature of the interaction between citizens and those they are attempting to hold accountable. In what we initially conceptualised as merit good services, such as food subsidies, citizens collect the subsidies directly from the service provider staff member; thus, the citizens and providers interact in the provision of services, and thereby have a relationship that extends beyond the accountability measures. This is in contrast to a service such as a road, which is built by service providers but accessed by citizens independently of the providers; once the road is built, the providers are no longer engaged in its day to day management and use. As a result, the relationship between the citizens and service providers is constrained to the accountability initiatives. Upon revisiting the framework synthesis, we extracted further evidence in support of this theory, which is described above.

In addition to the moderating variable regarding the nature of service provision, we further attempted to test the strength of the facilitator identified around service provider engagement by coding interventions according to whether they engaged with service providers in the design and/or implementation of the intervention a) at the point targeted by the intervention; b) with different public service officials whose behaviour wasn’t targeted; or c) no engagement with the supply side. However, the results were inconclusive, which was likely due to the small sample of studies within each group and additional key factors that made it difficult to statistically isolate the potential impact of service provider engagement.

### 9 COST EVIDENCE (REVIEW QUESTION 5)

Cost effectiveness is a key question for decision makers, and one that is rarely incorporated into systematic reviews.^6^ Unfortunately, few included studies included cost information and no studies included cost information systematically. We present the data here drawn directly from the study reports. Table 22 presents the types of programme costs analysed and key findings.

**Table 22:** Studies reporting implementation costs

*Study (Evers 2005 score)*	*Programme costs reported*	*Total cost data*	*Unit cost*	*Cost‐effectiveness*
Ghana: Alhassan et al. (2015) (5/12 = 42 per cent)	Community engagement implementation costs per cycle per year as part of the intervention design	US$ 12,160 total intervention cost^*^	US$ 380 per year per facility	‐
Karnataka: Ananthpur et al. (2014) (3/12 = 25 per cent)	Intervention costs	US$ 200,000 total intervention cost across 100 villages over four years	US$ 250 per village per year^*^	‐
Uganda: Björkman Nyqvist et al. (2017) (6/19 = 32 per cent)	Costs for collecting data for the report cards excluding the costs of collecting data for the evaluation or researcher time	USD$130,000 over 13 treatment facilities over a four‐year period^*^)	US$ 2,500 per facility/ community per year^*^	US$ 278 per death averted of a child aged under five
Uttar Pradesh: Pandey et al. (2007) (5/12 = 42 per cent)	Total costs of intervention (information campaign)	US$ 4,000 total costs of information campaign	US$ 72 per village per year^*^; US$ 0.22 per household in a village cluster	‐

*Notes: ‐ not reported*. ^*^ as estimated by authors based on reported data.

Two studies (Björkman Nyqvist et al. 2017; Pandey et al. 2007) presented some description of cost per outcome.^7^ The measures used to define costs and expenditures varied across these studies. None of the studies presented tables with detailed breakdown of costs by any kind of category or intervention. This limited the potential for any kind of comparisons across programme settings and intervention designs.

Programme costs were reported in four studies (Alhassan et al. 2015; Ananthpur et al. 2014; Björkman Nyqvist et al. 2017; Pandey et al. 2007). Only total costs were presented across these studies, and the costing methodology used in arriving at these cost values were described as “back of the envelope” and were not detailed. No studies included cost information systematically. One study was assessed as a full economic evaluation (cost‐effectiveness) (Björkman Nyqvist et al. 2017) and the remaining three were assessed as partial economic evaluations. The methodological quality of all the economic studies were found to be low (Table 22). Full critical appraisals using Evers et al.'s (2005) checklist are in Appendix 7.

Björkman Nyqvist et al. (2017) presented approximate total intervention costs (cost for collecting data for the report cards which were the main cost item) over a four‐year period for 13 treatment facilities at USD10,000 per facility.

Ananthpur et al. (2014) reported implementation costs of citizenship training and facilitation programme in rural India from 100 treatment villages. However, the attrition rate of household respondents was relatively high (attrition rate=39.91%) as 3,545 households were visited on both rounds of the panel compared to 4,000 households as per sample size calculations at the start of the study. The total cost reported at US$200,000 (2009 reference year) might have resulted in censoring^8^ of cost data calculations as missing not at random (Glick et al., 2015). Since the implementation involved citizen training and facilitation programme, the implementation costs are correlated to the cost of participants who were censored might differ from the cost of those without censored data.

Pandey et al. (2007) presented intervention costs of US$4,000 across 55 village clusters receiving the information campaign. There is very low attrition (1.91%), hence limited censoring of cost data. The authors also report costs of US$0.22 per household, presumably based on numbers of households with women reached by the intervention (data not reported). We also used information reported in the papers to standardise cost estimates wherever possible. For example, Alhassan (2015) reported costs across 32 health facilities (private: 21 intervention, 16 controls; public: 11 intervention, 15 controls) which represent about 10 per cent of the total number of accredited clinics/health centres in each of the two study regions. The costs reported at US$380 per intervention design would mean the overall cost of intervention to be US$ 12,160 across the 32 intervention health facilities.

We adjusted the cost estimates across the studies to specific target currency (US$) and the latest price year at which exchange rate conversion data are available (2016). The revised costs across the interventions identified for the calculations are reported in Table 23.

**Table 23:** Converted cost calculations to target currency (US$) and price year (2016)

Study	Assessment Period	Total cost data	Unit cost per year	Cost‐effectiveness
Ghana: Alhassan et al. (2015)	Baseline: June 2013	US$ 12,417	US$ 388 per facility	‐
	Follow‐up: March 2014			
Karnataka: Ananthpur et al. (2014)	Baseline: Oct‐Nov 2007	US$ 221,700	US$ 277 per village	‐
	Follow‐up: Oct‐Dec 2009			
Uganda: Björkman Nyqvist et al. (2017)	Long‐term evaluation: 2005‐2009	USD$144,105	US$ 2,771 per facility/ community	US$ 308 per death averted of a child aged under five
	Short‐run evaluation:			
	2005, 2007‐09			
Uttar Pradesh: Pandey et al. (2007)	Baseline: 2004	US$ 4,820	US$ 87 per village per year (US$ 0.27 per household in a village cluster)	‐
	Follow‐up: 2005 (after 12 months)			

* reference price year reported in the study used as base year for the cost calculation.

Three studies reported factors influencing implementation costs. Alhassan et al. (2015) suggested that factors such as champions being community members and resources being mobilised from within the community, influenced in keeping the implementation costs low. Björkman et al. (2017) highlighted the cost of data collection for the report card to be the main cost item influencing implementation costs. Pandey et al. (2007) suggested that if the government or local organisations could disseminate the information campaign, such as radio or newspapers, it could result in even lower intervention costs.

## 10 DISCUSSION

### 10.1 Summary of main results

This systematic review synthesises both quantitative and qualitative evidence from Thirty five studies of 41 unique policies or trial arms in 20 low‐ and middle‐income countries spanning five regions on programs that incorporate the principles of participation, accountability, transparency and inclusion (PITA) to increase citizen engagement in public service delivery. This covered programmes promoting participation (participatory priority setting); inclusion of marginalised groups; transparency (information on rights and public service performance), and/or citizen efforts to ensure public service accountability (citizen feedback and monitoring). The primary goal was to determine the programs’ impact on the quality of and access to public services, including health care, social protection, justice and physical infrastructure, and social and economic wellbeing of citizens (review question 1). We also considered the impact on intermediate outcomes in the causal chain, including citizen engagement and provider response (review question 2), and how results vary by participants and location (review question 3). In addition, we aimed to understand the mechanisms and processes through which change happens, by identifying programme design, implementation, context, and mechanism factors associated with programme effectiveness along the causal chain (review question 4). Due to insufficient cost data, we were unable to address review question 5 on the cost‐effectiveness of interventions incorporating PITA characteristics.

We used quantitative meta‐analysis to combine the results of the impact evaluations, including sub‐group analysis to explore heterogeneity by intervention, study location and other moderators. We conducted a detailed critical appraisal of the included impact evaluations to assess the credibility of the results. From the included programmes, we identified 36 associated qualitative and programmatic documents that we used to address review question 4. We used framework synthesis to synthesise the data.

We reported our quantitative results in this section along the causal chain to address review questions 1‐3, supported by the results from the qualitative framework synthesis to address review question 4. We start by presenting the results of the overall synthesis, followed by the individual results for the five intervention areas.

#### 10.1.1 Effectiveness of citizen engagement interventions

The meta‐analysis found that citizen engagement interventions are usually *effective in increasing the engagement of service users*, for example improving meeting attendance, contributing to community funds, and general knowledge about services. The average pooled effect on user engagement was an increase of 0.23 standard deviations (95%CI=0.12, 0.34) in the typical outcome measure across all interventions. Yet, the *effects of interventions promoting citizen engagement on provider actions were very limited*: the pooled effect on provider responsiveness was not significant across all PITA mechanisms and interventions.

##### Heterogeneity of impacts across populations

We considered diversity and equity of impacts across different population groups in three ways. Overall, few of the studies reported disaggregated intervention approaches and/or analysis of results for different population groups. We identified five studies that incorporated specific measures within the intervention to extend the engagement to vulnerable groups, which comprised three participatory planning interventions and one each of rights information provision and citizen feedback or monitoring. These programmes tended to have smaller effects on citizen engagement and access to services than other programmes, but it is unclear whether this was due to many of the programmes being implemented in challenging contexts (e.g. Afghanistan, Pakistan and DRC) rather than problems inherent in targeting vulnerable community members. Further, we identified nine studies that conducted sub‐group analysis to differentiate impacts for different population groups, most commonly by socio‐economic status and by gender, yet these were spread widely across intervention type and geography. Finally, we looked for studies that conducted equity‐oriented causal chain analysis, and identified only one study that conducted a detailed qualitative assessment that incorporated consideration of differentiated impacts for women. We also examined overall differences by global region, but were not able to find consistent differences by intervention or outcomes along the results chain. Ultimately, due to the small sample of studies across a wide range of interventions and outcomes, it is difficult to conclude anything systematically for different population or geographic groups.

#### 10.1.2 Performance information provision

We identified six evaluations of public official or service provider performance information interventions, such as the dissemination of municipal government performance scorecards in Afghanistan, Brazil, the Philippines and Uganda, and monitoring information provided in police stations in India.

The framework synthesis identified that amongst performance information interventions, a key facilitating factor was the extent to which implementers secured the support of and buy‐in from the actors whose performance was being analysed and disseminated. Without such support, the findings suggest that the targeted actors may be able to avoid accountability by either preventing full implementation of the intervention, or by successfully undermining the credibility of the performance information disseminated. Most of these interventions targeted political actors’ performance (as opposed to specific public services), in attempt to “shorten the long route” of citizen‐state accountability by increasing citizen engagement with politicians outside of elections. While interventions were at times successful in eliciting some improvements in politician performance, the findings suggest that, ultimately, this route remains too long to identify short‐term effects on service delivery. Politicians may claim plausible deniability of their individual capacity to influence service delivery change, and such interventions do not engage many key actors involved along the public service delivery supply chain.

#### 10.1.3 Citizen feedback mechanisms

We identified 10 evaluations of accountability interventions, which specifically comprised citizen feedback or monitoring mechanism interventions, that is, those that solicited feedback regarding and/or actively engaged citizens in the monitoring of service delivery, to hold public service providers and institutions responsible for executing their powers and mandates according to appropriate standards. These include community report cards in infrastructure (Afghanistan, Indonesia and Colombia), health (Ghana, Malawi and Uganda), agriculture (Uganda) and the security sector (DRC), and individual citizen “feedback loops” in Guinea, Kenya and Uganda.

The framework synthesis suggested that citizen feedback and monitoring interventions were more successful at achieving results where some or all of the following factors were present:

- Interventions targeted a service that citizens accessed through interactions with front‐line providers;
- A phased, facilitated approach jointly engaged citizens and service providers in monitoring
- Performance benchmarks;
- Creation of common knowledge of feedback or monitoring results; and
- Working through local community organizations to strengthen community members’ voices.

#### 10.1.4 Rights information provision

We identified five evaluations of *rights information* interventions, which enable users to demand minimum standards for access to services, such as for social protection services in Indonesia (food subsidies) and India (public works), maternal and child health care in India and freedom of information in Pakistan.

The results from the framework synthesis suggested that interventions informing citizens of their rights were more likely to succeed where they targeted the provision of a service citizens access directly from front‐line providers; created a sense of common knowledge about people's rights to the service among citizens and providers; and built an appropriate level of social sanction risk for providers.

#### 10.1.5 Participatory planning interventions

We identified nine participatory priority setting, planning or budgeting interventions, wherein citizens participated in setting the priorities for and/or planning of local services. These include support for participatory budgeting in municipal governments in Brazil, Mexico and Russia, and support for participatory planning in India, Pakistan, Guinea and Kenya. It also included requirements for inclusive participation in two fragile contexts, Afghanistan and DRC.

The framework synthesis suggested three factors improved the likelihood of achieving results along the causal chain:

- Strong local buy‐in from front‐line service providers for the intervention;
- Incorporating specific, culturally appropriate measures that address local barriers to the participation of vulnerable groups; and
- Interventions designed to spur the growth of local civil society and capacity for collective action.

##### Inclusive participation interventions

Five studies incorporated specific measures to strengthen the **inclusion** of marginalised and vulnerable groups such as women, ethnic minorities or lesbian, gay bisexual, transgender and intersex (LGBTI) people in citizen engagement interventions. These interventions are first grouped based on their primary aim (participation, accountability or transparency), but subsequently coded to identify the value‐add of specific measures to include vulnerable groups. These include programmes which tested the effectiveness of mandating women's participation in food distribution (Afghanistan and DRC), holding separate meetings for vulnerable groups in health programmes (India, Pakistan and Uganda).

### 10.1.6 Community‐based natural resource management

We identified seven community‐based natural resource management (CBNRM) interventions, wherein citizens form local collectives and take over the management of a shared resource, for forest management in Nepal, Madagascar and Tanzania, and water user associations in Brazil, China and the Philippines, and Namibia.

We identified four key contextual factors that mediated results chains amongst community‐based natural resource management (CBNRM) interventions. Where interventions required large shifts in control over the resource, representing a relinquishment of power from local officials to community groups, we identified a lack of engagement and buy‐in from local officials as a frequent barrier to the full implementation of the CBNRM policy. Critically, this barrier often resulted in situations in which community groups took on additional responsibilities for resource management, but did not gain access to the corresponding promised benefits. A related factor is the clarity of the national CBNRM policy context; where there were multiple vague and overlapping policies governing natural resource use, officials were more able to adjust or block full implementation of CBNRM in a way that preserved their power and control over resource benefit access. We identified external support to change resource use as a key facilitating factor: even in the absence of full policy implementation, access to alternative livelihoods such as tourism may still enable communities to realise the joint socio‐economic and environmental objectives of CBNRM. Finally, we identified the type and intensity of local resource use as a key moderating factor influencing the effectiveness of CBNRM; community management may not be appropriate in contexts prone to illegal logging or poaching, where attempts to enforce regulations may endanger community members.

### 10.2 Overall completeness and applicability of evidence

We identified 50 papers associated with 35 studies in low‐and middle‐income countries. While this is a growing evidence base, with 60 per cent of the included papers published within the last five years and 11 ongoing studies identified, this still represents a limited evidence base from which to make conclusions. The largest number of studies or trial arms testing a particular mechanism was 16, for studies testing policies to encourage or mandate participation, and these studies reported on a diverse range of outcomes. Geographically, the evidence base is skewed towards Sub‐Saharan Africa and India, representing half of the evidence base. We identified no studies from North Africa or the Middle East and limited evidence from Latin America and the Caribbean and East Asia and the Pacific region.

While we identified seven studies of community‐based natural resource management committees, these were all rated as having a high risk of bias or having some concerns, with the exception of Barde's (2017) evaluation of water user associations in Brazil.

We also undertook a formal assessment of the external validity of the included studies. A number of studies still do not report their sampling strategy clearly, and a surprisingly small share of studies specifically discuss the generalisability of their findings to other contexts. Only 11 studies explicitly discussed external validity. Among those studies, five acknowledged the limits to the generalisability of their findings, due to the small scale of the study or the sampling strategy. Four studies claimed generalisability of their findings, either to the level of an Indian state (Banerjee et al. 2014; Ravaillon et al., 2013), or to other areas of the country under similar conditions, such as density of population or distance to a health facility (Toutchon 2015; Björkman Nyqvist et al. 2017). Finally, two studies claimed generalizability of their findings to other contexts, and potentially other countries (Fiala & Premand, 2017; Timmons & Garfias, 2015).

### 10.3 Quality of the evidence

Overall, the quality of evidence from randomised studies is relatively high, with studies for the most part ensuring comparability of intervention and control groups and protecting them from selection bias. The risk of bias assessment is therefore more relevant at the outcome level. We identified concerns related to the way some outcomes are measured in the majority of studies. This is due to the use of self‐report measures that are often biased by the intervention itself. A majority of the non‐randomised studies are natural experiments, which in most cases did not provide enough information on the selection process into the programme to reject the risk of selection bias, or failed to overcome the selection bias and confounding that was identified. Transparency in reporting is an issue for randomised and non‐randomised studies alike given the few pre‐registrations of trial, outcomes or analysis plan. The use of methods such as placebo outcomes or groups, and blinding for outcome assessors or data analysts, is not common, though it seems relatively easy to implement and could reduce risks of biases.

### 10.4 Limitations and potential biases in the review process

There are several limitations of this review related to both the existing evidence base in this area and the synthesis approach.

### 10.4.1 Limitations of the existing evidence base

1. Statistical power for the meta‐analyses and heterogeneity analysis: Our ability to make strong conclusions on the effectiveness of the PITA mechanisms and interventions were limited by the number of studies looking at each intervention and outcome area. This was despite using a fairly high level of aggregation for mechanisms, intervention areas and outcomes.In addition, we were unable to undertake the full moderator analyses that we specified in the protocol to explore heterogeneity quantitatively that due to a limited number of included studies in each mechanism and intervention category.
2. Reporting in primary studies: We were limited in our ability to test key mechanisms quantitatively that we identified through the framework synthesis due to limited reporting of design and contextual characteristics in the impact evaluations. For example, our framework synthesis and previous reviews have suggested that the extent to which interventions engaged with or were strongly supported by national or local governments would be an important determining factor for effectiveness. However, primary studies rarely reported on this in detail.
3. Cost‐effectiveness analysis: We aimed to undertake an analysis of the cost‐effectiveness of the included set of interventions (review question 5), however we were limited by the available cost data.

### 10.4.2 Limitations of the review scope and synthesis process

1. The focus of our review questions were on the valued added of incorporating PITA characteristics into existing service delivery, and therefore we did not include studies that studied the impact of combining PITA‐based interventions with co‐interventions to improve resources or capacity for service delivery. One of the hypotheses emerging from our review is that citizen engagement interventions that do not incorporate complementary interventions along the service provider supply chain may be insufficient to improve key wellbeing outcomes for target communities. However, we are unable to say this conclusively without comparing to the results of interventions that do combine PITA mechanisms and supply side interventions. We believe that this would be a valuable subject for future synthesis.
2. We did not include studies of education related to PITA mechanisms in our review due to overlap with existing systematic reviews and time and resource limitations. However, the inclusion of this evidence base may have increased the power of our quantitative analysis and the generalisability of our results to this sector.
3. Due to time and resource limitations, we did not undertake independent double coding of effect size information or the qualitative data extraction. In addition, we only undertook double coding for the risk of bias assessments for a sample of 20 per cent of studies rather than the full set. However, the results of the independent double coding of risk of bias demonstrated a high level of agreement between the two authoauthors.

### 10.5 Deviations from protocol

This review largely followed the approach described in the associated protocol published in the Campbell Library (Waddington et al., 2018). However, we note several deviations.

1. Upon identifying the included studies, we mapped the characteristics of each intervention and produced a framework of five sub‐interventions that shared similar design characteristics. These categories were not pre‐specified in the protocol as we defined our intervention inclusion criteria using PITA design characteristics and were unsure what the final set of included interventions would look like. We used these categories to undertake sub‐group analysis by intervention area.
2. As noted in the previous section, we did not undertake full independent double coding of effect size information or the qualitative data extraction although categorisation of all effect sizes into outcome groups for every study was done by two authorauthors.
3. We discussed exploring the possibility of applying alternate methods to link the meta‐analysis with context and mechanism information, such as QCA (Befani, 2016).QCA articulates the associations between empirical effects and context and mechanism conditions drawing on “truth‐tables” which articulate all possible instances of conditions and show which cases share the same combination of conditions. We noted that the application of QCA is limited by the number of included studies, their comparability and the completeness of reporting within them, hence the application of QCA was not feasible in this review. We were unable to apply QCA to our review due to number of included studies, their comparability and the completeness of reporting within them. Instead we used realist‐informed framework synthesis that moved towards “best fit” framework synthesis to explore context and mechanism information.
4. In addition, we identified potential programme mechanisms and a moderator variable (merit versus pure public goods) in the qualitative framework synthesis that we subsequently tested in the meta‐analysis through sub‐group analysis. This moderator analysis was not described in the protocol.

### 10.6 Agreements and disagreements with other studies or reviews

This systematic review is the first that we are aware of to consider the effects of a range of interventions with PITA characteristics across a range of sectors. The findings from the review are broadly consistent with reviews that have examined governance interventions and/or have examined demand and supply in service delivery. For example, the recent review of community driven development programmes by White et al. (2018) found that effects tended to diminish further along the causal chain, such as programmes were often ineffective in improving wellbeing outcomes, apart from in the special case of water and sanitation.

Several high quality systematic reviews exist focusing specifically on the impact of community‐based monitoring and information interventions (Molina et al. 2016; Snilstveit et al. 2015). In 2016, Molina et al. published a review of the effects of 15 community monitoring studies in the health and education sectors. Snilstveit et al.'s (2015) mixed method systematic review examines the effects of education interventions including community‐based monitoring of schools and education systems.

Hanna et al.'s (2011) systematic review of anti‐corruption interventions found that monitoring interventions have been effective in cases where they were implemented and monitored by a party desiring to lower corruption, and where they have been combined with either nonfinancial or financial incentives. They also suggested community‐level monitoring works but only “*when the community can punish corruption*” (Hanna et al. 2011: 49).

USAID's (2015) Practitioner's Guide for Anticorruption Programming Guide aggregates lessons from more than 300 USAID programs between 2007 and 2013 which included anticorruption design elements.They suggest that public awareness campaigns or citizen monitoring groups have little impact without willing coordination with governments.

## 11 AUTHORS’ CONCLUSIONS

### 11.1 Implications for policy and programming

This section presents the main conclusions for policy and programmes from the synthesis of impact evidence on interventions promoting external participation and accountability in low‐ and middle‐income countries. As might be expected for a review of broad interventions and even broader scope of outcomes, there is significant heterogeneity in findings. In order to manage the anticipated heterogeneity, we developed a framework which enabled sensible grouping of interventions and outcomes. The results from analysis according to this grouping suggested significant heterogeneity in findings across intervention groupings and outcomes.

The first conclusion is that, regardless of intervention type, interventions are usually effective in improving engagement of citizens in service delivery and improving access to services and quality of service provision. However, external participation and accountability interventions are not often able to elicit strong responses from public services.

Secondly, evidence suggests some interventions may be more effective in improving service delivery outcomes, including those with stronger accountability components, and those providing rights information. The findings about relative effectiveness across interventions are tentative in light of the heterogeneity in evidence included in the review. More promising evidence, however, was found in the effectiveness of accountability interventions, including transparency for accountability, that targeted the provision of merit good‐type public services, as opposed to those that targeted pure public good‐type services. For merit good services, such as health care, citizens typically already came into contact with service delivery agents in order to access the service, and simply built on those relationships to advocate for improvements in service provision management; this multidimensional and ongoing personal engagement between providers and users, comprising both everyday service delivery and accountability engagement, was better able to elicit improvements in service provider actions, leading to greater impacts in quality of and access to services. In contrast, for pure public good‐type services, such as roads, citizens typically accessed or used the service independently of front‐line providers, and thus their relationship with service providers through citizen engagement efforts was more one‐dimensional, focused solely on the accountability efforts. The social sanctions threat of local civic engagement was not strong enough to overcome the power difference between providers and users, and thus interventions often failed to elicit responses in service provider actions, leading to a break in the causal chain. However, there is some evidence from Afghanistan that suggests that where interventions targeting pure public goods incorporate the engagement of strong, locally well‐respected civil society groups, the additional social capital provided by the CSO enables citizens to overcome this bottleneck and realise improvements in service delivery quality through citizen engagement – yet there is a caveat that effects may only hold so long as the active CSO engagement continues (Berman et al., 2018).

The third main conclusion is that outcomes tend to get smaller along the causal chain, to the extent that we do not expect participation and accountability interventions of themselves to improve wellbeing. This finding should not be surprising, partly because the deteriorating causal chain is a common occurrence, called elsewhere the “funnel of attrition” (see White, 2014). The other reason is that the systematic review inclusion criteria were limited to studies examining the marginal effect of a participation or accountability intervention on top of standard public service delivery. Hence, any study (or trial arm) that incorporated any co‐interventions, including increased resource delivery, was excluded. It is highly possible that participation and accountability interventions when provided alongside other services that can relieve important bottlenecks, can act to improve behavioural responses and wellbeing.

The results suggest particular attention should be paid to the following areas when designing and implementing interventions:

**Ensuring positive engagement with supply‐side actors at the intervention target level**

Many interventions experienced challenges stemming from a lack of positive engagement with supply‐side actors at the intervention target level, whose relative power the interventions often sought to diminish. Interventions seeking to change this balance of power with engagement and buy‐in from these actors are likely to be more effective in improving service delivery outcomes and state‐society relations. Interventions implemented with the strong support of the targeted supply‐side actors, such as the case of municipal governments that chose to implement participatory budgeting in Brazil or structured community engagement in the health sector have been able to realise positive impacts across the causal chain. In contrast, in Rajasthan, India, only national police leadership were involved in the design of the intervention; local police chiefs, whose behaviour was targeted, were not engaged, and were subsequently able to undermine or effectively block implementation of the intervention (Banerjee et al., 2014).

**Particular consideration for natural resource management committees**

In the majority of included intervention sub‐groups, a limited response on behalf of the service provider may at worst prevent outcomes tied to service provider response or lead to null effects. In the case of CBNRM, however, there is a risk of causing negative effects on well‐being outcomes, where a lack of full intervention implementation leads to a context in which resource‐ and time‐poor communities increase their burden of natural resource management, have less access to the resource due to sustainability restrictions, and are not afforded adequate compensation in the form of resource benefits ownership or alternative livelihoods support. For example, in Madagascar, Rasolofoson et al. (2015) reviewed the set of policies, laws and regulations for natural resource management in the country, and identified numerous inconsistencies and contraditions. They presented qualitative evidence suggesting that this complicated and contradictory policy and legal framework was exploited by front‐line forestry staff, who were able to manipulate implementation of the CBNRM forestry policy to suit their purposes and retain power and effective control over the resources, thus causing a break in the causal chain as implementation of the policy was neither complete nor consistent.

**Collaborative versus confrontational approaches to service provider engagement**

The findings of this review lend some support to the theory that citizens’ attempts to increase their relative power through means seen as confrontational by service providers often disincentivise service provider participation (World Bank 2004). The findings of this review suggest that approaches to citizen‐service provider engagement in the realm of accountability, including transparency for accountability, appear to work more effectively when implemented through phased, facilitated processes that are framed as collaborative, as opposed to one‐off accountability meetings that tend to be interpreted as confrontational. Interventions that promote transparency with the aim of triggering mechanisms that motivate citizens to demand greater accountability often fall closer to the confrontational spectrum, and their limited success on realizing outcomes along the causal chain is evident throughout the included studies. Those that promote an explicitly collaborative process may be more effective, particularly when they incorporate measures to improve citizens’ understanding of performance benchmarks. This was the case in Uganda, where Björkman Nyqvist et al. (2017) found that communities were better able to identify locally‐solvable problems within healthcare service provision and advocate for their improvements when they had access to performance benchmarks and training on healthcare monitoring; when local performance information was not provided, service proiders were better able to skirt accountability by identifying for community monitors key constraints over which they had no control. We note, however, a difference between interventions targeting individuals versus service provider institutions, and caution that it may be more difficult to engage in collaborative approaches to performance improvements with individuals, who are understandably more likely to feel personally targeted. In these situations, the synthesis suggests that ensuring the engagement of a locally credible messenger to disseminate performance information reduces the ability of the targeted individual to undermine, co‐opt or discredit the information. This was the case in the Philippines, where Capuno and Garcia (2010) evaluated the impact of a municipal scorecard intervention wherein the municipal governments themselves or locally respected CSOs presented the performance information, which prevented politicians under scrutiny from “shooting the messenger.” In contrast, Humphreys and Weinstein (2012) reported incidences of politicians either completely blocking dissemination of scorecards in their constituencies, or undermining and co‐opting the presentations of the findings by a nationally‐respected but locally less‐well‐known CSO, such that participants came away with an improved perception of the politician's effectiveness despite poor performance based on the scorecard.

**Facilitating engagement by building local social capital and capacity for collective action**

Across included interventions, a key facilitator identified in the framework synthesis was the value‐add of incorporating into intervention design active engagement with local organized community groups, such as CSOs or interest groups, or the inclusion of measures that explicitly sought to build local social capital and capacity for collective action. The role of civil society support to communities may be critical not only for encouraging engagement in monitoring and accountability processes, but also for shifting the balance of power between citizens and public service providers of indirectly delivered services. There is some evidence that CSO engagement is particularly critical for interventions targeting indirectly‐delivered, pure public goods. Engaging CSOs in the intervention may strengthen the social capital of individual citizens: the stronger voice may increase citizens’ ability to access the information needed to hold service providers accountable; help bring key stakeholders together in interface meetings; and increase citizens’ bargaining power with service providers, thus strengthening their capacity to realize improvements in service delivery quality. As above, this was found to be the case in Afghanistan, where Berman et al. (2018) presented qualitative evidence suggesting that the strength of the name of the highly respected CSO in the intervention enabled community monitors to access key documents such as contracts that had previously been denied. The CSO was also able to engage key actors from government in the monitoring meetings, strengthening the risk for service providers – yet when the CSO disengaged, the effects petered out. This suggests the importance both of long‐term engagement and of long‐term follow‐up, as outcomes are frequently not static.

### 11.2 Implications for research

The results suggested significant heterogeneity according to study design and implementation characteristics. Thus, RCTs tended to have smaller effects than non‐randomised studies. Although this finding is consistent across different literatures, and is indicate of the types of effect estimand that RCTs produce, it is important to note that well‐conducted RCTs are considered to provide the most reliable estimates of outcome changes, and as a study design is highly amenable to the types of interventions contained in this review. The result of the risk of bias analysis has shown that the overall quality of evidence from the randomised studies is relatively high: risks of confounding and selection bias are low, however researchers should rely less on self‐reported outcome measures, which are more susceptible to biases. A majority of non‐randomised studies were at high risk of selection bias and confounding, due to the unclear or self‐selection of communities into the programme and the lack of baseline data. When baseline data are available and the appropriate analysis method is used, authors may overcome these biases. There are concerns related to reporting; in particular, there is a lack of transparency with regards to how analyses were conducted, how authors responded to implementation problems (e.g. attrition), and approaches to selecting groups for inclusion in the study (external validity).

More evaluations are needed comparing citizen‐engagement interventions efforts against (or combined with) interventions focused on other aspects of governance such as by increasing access and quality of public services through the compact between state and service provider (e.g. Dal Bó et al., 2019; Callen, Gulzar, Hasanain, Khan, & Rezaee, 2018). Efforts to evaluate comparative effects of governance and other approaches could also draw on successful approaches from other areas (McIntosh & Zeitlin, 2018). The evidence provided usually relates to between 12 months and five years of follow‐up after initiation of intervention. There may therefore be greater opportunities to measure the impact of interventions over the longer‐term, by following‐up existing studies. We also anticipate that there are more opportunities to conduct rigorous natural experiments evaluating “real world” national policy or reform over the longer‐term than have been taken so far, including through use of regression discontinuity design (as indicated by the study awaiting classification in this review – Tohari et al., 2017). Such studies may be done particularly cost‐effectively where existing survey or administrative data can be used.

Researchers should consider the following when undertaking impact evaluations in this area:

1. **Reporting of intervention and comparison group conditions:** in many cases, we had difficulties in identifying precisely what the impact evaluation was evaluating; either due to limited reporting of the intervention characteristics or because the status of the citizens in the comparison group was unclear. As noted in the search results section, we decided to exclude two studies after identifying additional documents that alerted us to the presence of significant co‐interventions not reported on in the impact evaluations. This limits the amount of learning that can take place from the studies, for implementers who may wish to take the intervention to a new setting or for synthesis work. Authors should consider drawing on tools such as the TIDieR intervention reporting guidelines for health (Hoffman et al. 2014).
2. **Consideration of equity**: there is a lack of research on how citizen engagement interventions affect women, ethnic groups or other vulnerable groups. For example, few impact evaluations undertook sub‐group analysis for these groups or undertook parallel qualitative research to understand how these groups are able to participate in this type of programme or their perspectives. For example, we only identified two studies that assessed how mandating the participation of women into PITA processes affects services and wellbeing. Given that the majority of the interventions covered by our review rely extensively on participation of the community and frequently do not, at least explicitly, make efforts to incorporate vulnerable groups, it is important to understand how vulnerable groups are able to participate.
3. **Prioritisation of mixed‐methods impact evaluations:** few studies incorporated qualitative research that would allow them to uncover the mechanisms that lead to the success or failure of the intervention. Ananthpur et al. (2014) was one notable exception that included a four‐year ethnography of the intervention to understand the mechanisms that led to the lack of impact in the programme.
4. **Greater standardisation of outcomes collected in studies of PITA mechanisms:** in many sectors, there are common wellbeing outcome indicators which facilitates cross‐study learning (e.g. child diarrhoeal morbidity in studies of water, sanitation and hygiene interventions). There does seem to have been some standardisation already done for some governance interventions, for example, reporting of quality of participation in community‐driven development programmes. However, there is far greater scope for standardisation of outcomes for commonly used constructs for citizen engagement interventions, as shown in the great diversity of outcomes collected.

We have attempted in this review to demonstrate that it is possible to undertake higher‐level synthesis work to articulate broader mechanisms at play which aimed to inform centralised strategic planning. However, we note that systematic reviews are usually most effective – especially in communicating findings to programmers – when they examine a particular intervention, such as “community‐driven development”. Hence our attempt in this study to provide both broader‐level analysis of empirical results across studies and within‐study findings for particular interventions. In addition, our study identified several potential areas for future synthesis work:

1. We focused in this review on interventions that isolated the PITA component, and therefore **did not incorporate co‐interventions to target the resource base or capacity of the public service providers**. It would be useful for a future systematic review to compare the findings of interventions that introduce only PITA mechanisms alongside interventions that combine PITA mechanisms with co‐interventions. Future research could also explore the comparative effectiveness of interventions instigating PITA mechanisms within the external engagement domain of governance versus those aiming to strengthen PITA mechanisms within the internal institutional systems of public service provision. Any synthesis work would likely need to focus on particular aspects of participation and accountability, or intervention groups, in order to be both manageable and policy‐relevant.
2. We excluded studies of interventions from the education sector, as they have been synthesised by several previous reviews. However, we note that a similar mechanisms synthesis could be undertaken of studies in the education sector, which constitute a substantial body of research in this area.
3. Fully mixed‐methods systematic reviews examining the effectiveness of particular intervention types (e.g. participatory budgeting, water user associations) would also be valuable.

## 12 ROLES AND RESPONSIBILITIES

The study protocol was developed by Hugh Waddington (HW), Ada Sonnenfeld (AS) and Jennifer Stevenson (JS). The search strategy was designed with John Eyers, and carried out by AS, JS and HW. Juliette Finetti and JS did the critical appraisal with inputs from HW. JS and HW collected the effect size data with inputs from AS, and HW did the meta‐analysis. AS collected the qualitative data and did the framework synthesis with inputs from HW. HW, AS and JS wrote the report. Denny John did the cost analysis with inputs from HW.

## 13 DECLARATIONS OF INTEREST

The authors have no vested interest in the outcomes of this review, nor any incentive to represent findings in a biased manner.

## INFORMATION ABOUT THIS REVIEW

### Review authors

#### Lead review author

The lead author is the person who develops and co‐ordinates the review team, discusses and assigns roles for individual members of the review team, liaises with the editorial base and takes responsibility for the on‐going updates of the review.

**Table:** 

Name: Hugh Waddington
Title: Senior Evaluation Specialist
Affiliation: International Initiative for Impact Evaluation
Address: 20 Bloomsbury Square
City, State, Province or County: London
Post code: WC1A 2NS
Country: U.K.
Phone: +44 207 958 8351
Email: hwaddington (at) 3ieimpact (dot) org

**Co‐author(s):**

**Table:** 

Name: Ada Sonnenfeld
Title: Research Consultant
Affiliation: International Initiative for Impact Evaluation
Country: U.K.
Email: asonnenfeld (at) 3ieimpact (dot) org

Name: Jennifer Stevenson		
Title: Evaluation Manager		
Affiliation: Education Endowment Foundation		
Country: U.K.		
Email: jennifer.stevenson (at) eefoundation (dot) org (dot) uk		

Name: Juliette Finetti
Title: Research Associate, 3ie
Country: U.K.
Email: jfinetti (at) 3ieimpact (dot) org

Name: Marie Gaarder
Title: Head of Evaluation, 3ie
Affiliation: International Initiative for Impact Evaluation
Country: Norway
Email: mgaarder (at) 3ieimpact (dot) org

Name: Denny John
Title: Evidence Synthesis Specialist
Affiliation: Campbell Collaboration
Country: New Delhi
Email: djohn (at) campbellcollaboration (dot) org

## 14 ROLES AND RESPONSIBILITIES

- **Content:** The substantive component of the analysis was undertaken by Ada Sonnenfeld (AS), Jennifer Stevenson (JS) and Hugh Waddington (HW), who provided additional inputs on intervention design and outcomes. The team were supported by an advisory group of academics and policy makers with specific expertise in governance.
- **Information retrieval:** AS and JS designed the electronic search terms, which were developed in platform search protocols by John Eyers. AS and JS undertook the screening of titles and abstracts and online sources. AS, JS and HW screened studies at full text.
- **Data collection:** AS, JS and HW collected descriptive data from included studies. JS and HW extracted effect size data. Juliette Finetti and JS did the risk of bias assessment. Denny John from the Campbell Collaboration extracted information on unit costs.
- **Statistical analysis and framework synthesis**: HW did the meta‐analysis. AS did the framework synthesis. All authors contributed to the discussion and implications.

### 14.1 Sources of support

We are grateful for financial support from the United States Agency for International Development (USAID) via NORC project number 7554.070.01.

Raj Popat provided research assistance on searching.

We thank Daniel Phillips (NatCen) for coordinating the peer review. Helpful inputs were given at various stages of the review process by the Campbell Collaboration Methods Group, three anonymous referees, USAID and the following stakeholder advisory group members:

- Andrew Greer, USAID
- Annette Brown, FHI360
- Courtney Tolmey, Results for Development
- Erik Wibbels, Duke University
- Guy Grossman, University of Pennsylvania
- Joanne Trotter, Aga Khan Foundation
- Laura Adams, USAID
- Morgan Holmes, USAID.

Finally, we are also grateful to Rohini Pande for helpful advice at the scoping stage of the review.

## 15 DECLARATIONS OF INTEREST

None of the team members have any financial interests in the review or have worked on primary research covering the interventions covered by the review.

## 16 PLANS FOR UPDATING THE REVIEW

The authors will undertake, or contribute to, updates once resources are identified and further high quality studies become available.

## Supporting information

Supplementary information

## Footnotes

1 Killias et al. (2016) are registering a review on the effectiveness of anti‐corruption measures. The study protocol is available from the Campbell library: https://www.campbellcollaboration.org/media/k2/attachments/Killias_Corruption_Protocol.pdf.

2 See appendix 2 for the full details of the grey literature search.

3 Several of the RCTs that we included had multiple trial arms using the same comparison group, but tested interventions that fell into different categories in our intervention framework (e.g. Olken, 2007). In these cases, the trial arms were included in different meta‐analyses by intervention type.

4 We state they did not explicitly address equity concerns, as it is possible the intervention design considered marginalised and vulnerable groups, but it was not reported in the intervention description in the impact evaluation or additional documents.

5 As requested by the methods reviewer, we also present odds ratios for dichotomous outcomes in **Appendix 6 Figure A6.13**.

6 For a good example, see Doocy and Tappis (2017).

7 Björkman et al. (2017) only reported results of the combined package of facilitated meetings to enhance participation with the dissemination of report cards on the facility's performance. Hence the comparison of costs with the facilitated meetings without report cards was not possible based on published data.

8 Cost data may be considered as incomplete due to loss of follow‐up. MNAR or non‐ignorably missing censoring occurs when the mechanism that generates the censored observations is correlated with the mechanism that generates cost (Glick, Doshi, Sonnad, & Polsky, 2015).
