# Supplementary material for: Citizen engagement in public services in low‐ and middle‐income countries: A mixed‐methods systematic review of participation, inclusion, transparency and accountability (PITA) initiatives
Source: Campbell Syst Rev. 2019 Aug 2;15(1-2):e1025. doi: 10.1002/cl2.1025 (PMC8356537; doi:10.1002/cl2.1025)
Supplement: Supplementary file 1 — Supplementary information [file CL2-15-e1025-s001.docx]

Appendix 1: Detailed search strategy

1. Social Sciences Citation Index (Web of Science) – Searched 18th January 2018

Indexes=SSCI Timespan=2000-2018

# 29 708 – (Economic limit)

#26 AND #11 AND #5

# 28 2,428 (with Study design limit)

#27 AND #26 AND #5

# 27 656,852

TS=("random* control* trial*" or "random* trial*" or RCT or "propensity score matching" or PSM or "regression discontinuity design" or RDD or "difference in difference*" or DID or difference-in-difference or evaluat* or matching or "interrupted time series" or (random* NEAR/3 allocat*) or "instrumental variable*" or IV or ((quantitative or "comparison group" or counterfactual or "counter factual" or counter-factual or experiment* or quasi-experimental or "quasi experimental" ) NEAR/3 (design or study or analysis)) or QED or "field experiment" or "field trial")

# 26 56,831

#25 OR #24 OR #23 OR #22 OR #21 OR #20 OR #19 OR #18 OR #17 OR #16 OR #15 OR #14 OR #13 OR #12

# 25 5,752

TS=(((sms OR "short message*" OR "text message*" OR bulk-messag* OR "bulk messag*" OR mass-messag* OR "mass messag*" OR "public awareness" OR engagement OR information) NEAR/3 (campaign* OR strategy OR strategies)) OR "information dissemination")

# 24 155

TS=("standard service*" OR standard-service* OR standardized-service* OR standardised-service* OR "standardized service*" OR "standardised service*")

# 23 2,079

TS=((service* OR one-stop OR "one stop") NEAR/1 (centre* OR center* OR shop*)) OR TS=((communit* OR community-based) NEAR/3 monitor*)

# 22 177

TS=(("social* accountab*") NEAR/2 (mechanism* OR system* OR arrange* OR organi* OR regulat*)) OR TS=((social OR public) NEAR/1 audit)

# 21 2,533

TS=("report card*" OR reportcard* OR report-card* OR "score card*" OR scorecard* OR score-card*) OR TS=("political reserv*" OR "reserved place*" OR "reserved position*" OR "reserved seat*")

# 20 5,495

TS=("e governance" OR e-governance OR egovernance OR "electronic governance") OR TS=((politic* NEAR/3 (inclus* OR participat* OR quota OR quotas)) OR (quota* NEAR/3 participat*))

# 19 13

TS=((((disaster* OR disaster-risk*) NEAR/6 (respond* OR response* OR management OR reduc* OR preparedness)) OR DRR) NEAR/3 (committee* OR council* OR association* OR shura))

# 18 73

TS=((communit* OR district* OR cluster OR cluster-level) NEAR/6 development NEAR/3 (committee* OR council* OR association* OR shura))

# 17 2,383

TS=(("natural resource*" OR natural-resource* OR NRM OR "common property" OR common-property OR "common resource*" OR common-resource* OR water-use* OR "water use*" OR "water management" OR water-management OR land-use* OR "land use*" OR land-management OR "land management" OR irrigat*) NEAR/6 (participat* OR transparen* OR inclus* OR represent* OR consult* OR community* OR committee* OR council* OR association* OR group* OR shura))

# 16 9,551

TS=((health OR healthcare OR hospital*) NEAR/3 (committee* OR "action group*" OR council* OR association* OR shura))

# 15 24,498

TS=((inclus* OR particip* ) NEAR/6 (strateg* OR action* OR budget* OR development OR plan*))

# 14 2,853

TS=("community engagement" OR "community consultation*" OR (civic NEAR/3 education))

# 13 2,480

TS=((communit* OR inclus* OR particip*) NEAR/6 (((climate-change OR "climate change") NEAR/2 (adapt* OR mitigat* OR vulnerab*)) or resilien*))

# 12 1,000

TS=(((disaster* NEAR/2 reduc* NEAR/2 risk*) OR (disaster* NEAR/2 (respond* OR response* OR manag*)) OR ((hazard* OR risk* OR vulnerab*) NEAR/2 (map* OR assess*)) OR HVA OR HVRA OR DRR) NEAR/6 (participat* OR inclus* OR consult* OR communit*))

# 11 202,194

#10 OR #9 OR #8 OR #7 OR #6

# 10 7,159

TS=(economic NEAR/2 model*)

# 9 18,324

TS=("cost minimi*" OR "cost-utilit*" OR "health utilit*" OR "economic evaluation*" OR "economic review*" OR "cost outcome" OR "cost analys*" OR "economic analys*" OR "budget* impact analys*")

# 8 188,627

TS=(cost-effective* OR cost-benefit OR costs)

# 7 5,346

TS=("life year" OR "life years" OR qaly* OR daly*)

# 6 189,478

TS=((cost OR economic*) AND (costs OR cost-effectiveness OR markov))

# 5 443,462

#4 OR #3 OR #2 OR #1

# 4 2,714

TS=((lmic or lmics or "third world" or "lami countr*")) OR TS=("transitional countr*")

# 3 8,531

TS=(((developing or "less* developed" or "under developed" or underdeveloped or "middle income" or "low* income") NEAR/1 (economy or economies))) OR TS=((low* NEAR/1 (gdp or gnp or "gross domestic" or "gross national"))) OR TS=((low NEAR/3 middle NEAR/3 countr*))

# 2 151,215

TS=("Developing Countries") OR TS=(Africa or Asia or Caribbean or "West Indies" or "South America" or "Latin America" or "Central America") OR TS=(((developing or "less* developed" or "under developed" or underdeveloped or "middle income" or "low* income" or underserved or "under served" or deprived or poor*) NEAR/1 (countr* or nation* or population* or world)))

# 1 374,017

TS=((Afghanistan or Albania or Algeria or Angola or Argentina or Armenia or Armenian or Aruba or Azerbaijan or Bangladesh or Benin or Byelarus or Byelorussian or Belarus or Belorussian or Belorussia or Belize or Bhutan or Bolivia or Bosnia or Herzegovina or Hercegovina or Botswana or Brasil or Brazil or Bulgaria or "Burkina Faso" or "Burkina Fasso" or "Upper Volta" or Burundi or Urundi or Cambodia or "Khmer Republic" or Kampuchea or Cameroon or Cameroons or Cameron or Camerons or "Cape Verde" or "Central African Republic" or Chad or China or Colombia or Comoros or "Comoro Islands" or Comores or Mayotte or Congo or Zaire or "Costa Rica*" or "Cote d'Ivoire" or "Ivory Coast" or Cuba or Djibouti or "French Somaliland" or Dominica or "Dominican Republic" or "East Timor" or "East Timur" or "Timor Leste" or Ecuador or Egypt or "United Arab Republic" or "El Salvador" or Eritrea or Ethiopia or Fiji or Gabon or "Gabonese Republic" or Gambia or Gaza or "Georgia Republic" or "Georgian Republic" or Ghana or Grenada or Guatemala or Guinea or Guiana or Guyana or Haiti or Hungary or Honduras or India or Maldives or Indonesia or Iran or Iraq or Jamaica or Jordan or Kazakhstan or Kazakh or Kenya or Kiribati or Korea or Kosovo or Kyrgyzstan or Kirghizia or "Kyrgyz Republic" or Kirghiz or Kirgizstan or "Lao PDR" or Laos or Lebanon or Lesotho or Basutoland or Liberia or Libya or Macedonia or Madagascar or "Malagasy Republic" or Malaysia or Malaya or Malay or Sabah or Sarawak or Malawi or Mali or "Marshall Islands" or Mauritania or Mauritius or "Agalega Islands" or Mexico or Micronesia or "Middle East" or Moldova or Moldovia or Moldovian or Mongolia or Montenegro or Morocco or Ifni or Mozambique or Myanmar or Myanma or Burma or Namibia or Nepal or "Netherlands Antilles" or "New Caledonia" or Nicaragua or Niger or Nigeria or Pakistan or Palau or Palestine or Panama or Paraguay or Peru or Philippines or Philipines or Phillipines or Phillippines or "Puerto Ric*" or Romania or Rumania or Roumania or Rwanda or Ruanda or "Saint Lucia" or "St Lucia" or "Saint Vincent" or "St Vincent" or Grenadines or Samoa or "Samoan Islands" or "Navigator Island" or "Navigator Islands" or "Sao Tome" or Senegal or Serbia or Montenegro or Seychelles or "Sierra Leone" or "Sri Lanka" or "Solomon Islands" or Somalia or "South Africa" or Sudan or Suriname or Surinam or Swaziland or Syria or Tajikistan or Tadzhikistan or Tadjikistan or Tadzhik or Tanzania or Thailand or Togo or Togolese Republic or Tonga or Tunisia or Turkey or Turkmenistan or Turkmen or Uganda or Ukraine or Uzbekistan or Uzbek or Vanuatu or "New Hebrides" or Venezuela or Vietnam or "Viet Nam" or "West Bank" or Yemen or Yugoslavia or Zambia or Zimbabwe) NOT ("African-American*" OR "African-American*" OR "Mexican American*" OR "American Indian*" OR "Asian American*" OR "native american*"))

2. Econlit (Ovid) <1886 to December 2017> Searched 18th January 2018

1 ((Afghanistan or Albania or Algeria or Angola or Argentina or Armenia or Armenian or Aruba or Azerbaijan or Bangladesh or Benin or Byelarus or Byelorussian or Belarus or Belorussian or Belorussia or Belize or Bhutan or Bolivia or Bosnia or Herzegovina or Hercegovina or Botswana or Brasil or Brazil or Bulgaria or "Burkina Faso" or "Burkina Fasso" or "Upper Volta" or Burundi or Urundi or Cambodia or "Khmer Republic" or Kampuchea or Cameroon or Cameroons or Cameron or Camerons or "Cape Verde" or "Central African Republic" or Chad or China or Colombia or Comoros or "Comoro Islands" or Comores or Mayotte or Congo or Zaire or "Costa Rica*" or "Cote d'Ivoire" or "Ivory Coast" or Cuba or Djibouti or "French Somaliland" or Dominica or "Dominican Republic" or "East Timor" or "East Timur" or "Timor Leste" or Ecuador or Egypt or "United Arab Republic" or "El Salvador" or Eritrea or Ethiopia or Fiji or Gabon or "Gabonese Republic" or Gambia or Gaza or "Georgia Republic" or "Georgian Republic" or Ghana or Grenada or Guatemala or Guinea or Guiana or Guyana or Haiti or Hungary or Honduras or India or Maldives or Indonesia or Iran or Iraq or Jamaica or Jordan or Kazakhstan or Kazakh or Kenya or Kiribati or Korea or Kosovo or Kyrgyzstan or Kirghizia or "Kyrgyz Republic" or Kirghiz or Kirgizstan or "Lao PDR" or Laos or Lebanon or Lesotho or Basutoland or Liberia or Libya or Macedonia or Madagascar or "Malagasy Republic" or Malaysia or Malaya or Malay or Sabah or Sarawak or Malawi or Mali or "Marshall Islands" or Mauritania or Mauritius or "Agalega Islands" or Mexico or Micronesia or "Middle East" or Moldova or Moldovia or Moldovian or Mongolia or Montenegro or Morocco or Ifni or Mozambique or Myanmar or Myanma or Burma or Namibia or Nepal or "Netherlands Antilles" or "New Caledonia" or Nicaragua or Niger or Nigeria or Pakistan or Palau or Palestine or Panama or Paraguay or Peru or Philippines or Philipines or Phillipines or Phillippines or "Puerto Ric*" or Romania or Rumania or Roumania or Rwanda or Ruanda or "Saint Lucia" or "St Lucia" or "Saint Vincent" or "St Vincent" or Grenadines or Samoa or "Samoan Islands" or "Navigator Island" or "Navigator Islands" or "Sao Tome" or Senegal or Serbia or Montenegro or Seychelles or "Sierra Leone" or "Sri Lanka" or "Solomon Islands" or Somalia or "South Africa" or Sudan or Suriname or Surinam or Swaziland or Syria or Tajikistan or Tadzhikistan or Tadjikistan or Tadzhik or Tanzania or Thailand or Togo or Togolese Republic or Tonga or Tunisia or Turkey or Turkmenistan or Turkmen or Uganda or Ukraine or Uzbekistan or Uzbek or Vanuatu or "New Hebrides" or Venezuela or Vietnam or "Viet Nam" or "West Bank" or Yemen or Yugoslavia or Zambia or Zimbabwe) not ("African-American*" or "African-American*" or "Mexican American*" or "American Indian*" or "Asian American*" or "native american*")).ti,ab,hw. (168475)

2 ("Developing Countries" or Africa or Asia or Caribbean or "West Indies" or "South America" or "Latin America" or "Central America" or ((developing or "less* developed" or "under developed" or underdeveloped or "middle income" or "low* income" or underserved or "under served" or deprived or poor*) adj1 (countr* or nation* or population* or world))).ti,ab,hw. (92566)

3 (((developing or "less* developed" or "under developed" or underdeveloped or "middle income" or "low* income") adj1 (economy or economies)) or (low* adj1 (gdp or gnp or "gross domestic" or "gross national")) or (low adj3 middle adj3 countr*)).ti,ab,hw. (5319)

4 (lmic or lmics or "third world" or "lami countr*" or "transitional countr*").ti,ab,hw. (1994)

5 or/1-4 (226610)

6 ((cost or economic*) and (costs or cost-effectiveness or markov)).ti,ab,hw. (60622)

7 ("life year" or "life years" or qaly* or daly*).ti,ab,hw. (847)

8 (cost-effective* or cost-benefit or costs).ti,ab,hw. (104771)

9 ("cost minimi*" or "cost-utilit*" or "health utilit*" or "economic evaluation*" or "economic review*" or "cost outcome" or "cost analys*" or "economic analys*" or "budget* impact analys*").ti,ab,hw. (11892)

10 (economic adj2 model*).ti,ab,hw. (13405)

11 or/6-10 (128373)

12 ("random* control* trial*" or "random* trial*" or RCT or "propensity score matching" or PSM or "regression discontinuity design" or RDD or "difference in difference*" or DID or difference-in-difference or evaluat* or matching or "interrupted time series" or (random* adj3 allocat*) or "instrumental variable*" or IV or ((quantitative or "comparison group" or counterfactual or "counter factual" or counter-factual or experiment* or quasi-experimental or "quasi experimental") adj3 (design or study or analysis)) or QED or "field experiment" or "field trial").ti,ab,hw. (121663)

13 (((disaster* adj2 reduc* adj2 risk*) or (disaster* adj2 (respond* or response* or manag*)) or ((hazard* or risk* or vulnerab*) adj2 (map* or assess*)) or HVA or HVRA or DRR) adj6 (participat* or inclus* or consult* or communit*)).ti,ab,hw. (58)

14 ((communit* or inclus* or particip*) adj6 (((climate-change or "climate change") adj2 (adapt* or mitigat* or vulnerab*)) or resilien*)).ti,ab,hw. (196)

15 ("community engagement" or "community consultation*" or (civic adj3 education)).ti,ab,hw. (225)

16 ((inclus* or particip*) adj6 (strateg* or action* or budget* or development or plan*)).ti,ab,hw. (3832)

17 ((health or healthcare or hospital*) adj3 (committee* or "action group*" or council* or association* or shura)).ti,ab,hw. (283)

18 (("natural resource*" or natural-resource* or NRM or "common property" or common-property or "common resource*" or common-resource* or water-use* or "water use*" or "water management" or water-management or land-use* or "land use*" or land-management or "land management" or irrigat*) adj6 (participat* or transparen* or inclus* or represent* or consult* or community* or committee* or council* or association* or group* or shura)).ti,ab,hw. (734)

19 ((communit* or district* or cluster or cluster-level) adj6 development adj3 (committee* or council* or association* or shura)).ti,ab,hw. (33)

20 ((((disaster* or disaster-risk*) adj6 (respond* or response* or management or reduc* or preparedness)) or DRR) adj3 (committee* or council* or association* or shura)).ti,ab,hw. (0)

21 ("e governance" or e-governance or egovernance or "electronic governance" or (politic* adj3 (inclus* or participat* or quota or quotas)) or (quota* adj3 participat*)).ti,ab,hw. (1125)

22 ("report card*" or reportcard* or report-card* or "score card*" or scorecard* or score-card* or "political reserv*" or "reserved place*" or "reserved position*" or "reserved seat*").ti,ab,hw. (618)

23 (("social* accountab*" adj2 (mechanism* or system* or arrange* or organi* or regulat*)) or ((social or public) adj1 audit)).ti,ab,hw. (58)

24 (((service* or one-stop or "one stop") adj1 (centre* or center* or shop*)) or ((communit* or community-based) adj3 monitor*)).ti,ab,hw. (273)

25 ("standard service*" or standard-service* or standardized-service* or standardised-service* or "standardized service*" or "standardised service*").ti,ab,hw. (19)

26 (((sms or "short message*" or "text message*" or bulk-messag* or "bulk messag*" or mass-messag* or "mass messag*" or "public awareness" or engagement or information) adj3 (campaign* or strategy or strategies)) or "information dissemination").ti,ab,hw. (941)

27 or/13-26 (8171)

28 5 and 12 and 27 (365)

29 limit 28 to yr="2000 -Current" (331) – Study Design Filter

30 5 and 11 and 27 (195)

31 limit 30 to yr="2000 -Current" (166) – Economic Filter

3. Ebsco Discovery Database – Repec & World Bank e-Library – Searched 18th January 2018

S29 S5 AND S11 AND S26 . 5,500 – Economic Filter (limited to Repec – 348; WB e-lib 48)

Date of Publication: 20000101-20181231 Database - Discovery Service for 3ie, Inc.

S28 S5 AND S26 AND S27 17,792 – Study Design Filter (limited to Repec – 844; WB e-lib 123)

S27 TI ( ("random* control* trial*" or "random* trial*" or RCT or "propensity score matching" or PSM or "regression discontinuity design" or RDD or "difference in difference*" or DID or difference-in-difference or evaluat* or matching or "interrupted time series" or (random* N3 allocat*) or "instrumental variable*" or IV or ((quantitative or "comparison group" or counterfactual or "counter factual" or counter-factual or experiment* or quasi-experimental or "quasi experimental" ) N3 (design or study or analysis)) or QED or "field experiment" or "field trial") ) OR AB ( ("random* control* trial*" or "random* trial*" or RCT or "propensity score matching" or PSM or "regression discontinuity design" or RDD or "difference in difference*" or DID or difference-in-difference or evaluat* or matching or "interrupted time series" or (random* N3 allocat*) or "instrumental variable*" or IV or ((quantitative or "comparison group" or counterfactual or "counter factual" or counter-factual or experiment* or quasi-experimental or "quasi experimental" ) N3 (design or study or analysis)) or QED or "field experiment" or "field trial") ) OR SU ( ("random* control* trial*" or "random* trial*" or RCT or "propensity score matching" or PSM or "regression discontinuity design" or RDD or "difference in difference*" or DID or difference-in-difference or evaluat* or matching or "interrupted time series" or (random* N3 allocat*) or "instrumental variable*" or IV or ((quantitative or "comparison group" or counterfactual or "counter factual" or counter-factual or experiment* or quasi-experimental or "quasi experimental" ) N3 (design or study or analysis)) or QED or "field experiment" or "field trial") ) 12,556,913

S26 S12 OR S13 OR S14 OR S15 OR S16 OR S17 OR S18 OR S19 OR S20 OR S21 OR S22 OR S23 OR S24 OR S25 589,952

S25 TI ( (((sms OR "short message*" OR "text message*" OR bulk-messag* OR "bulk messag*" OR mass-messag* OR "mass messag*" OR "public awareness" OR engagement OR information) N3 (campaign* OR strategy OR strategies)) OR "information dissemination") ) OR AB ( (((sms OR "short message*" OR "text message*" OR bulk-messag* OR "bulk messag*" OR mass-messag* OR "mass messag*" OR "public awareness" OR engagement OR information) N3 (campaign* OR strategy OR strategies)) OR "information dissemination") ) OR SU ( (((sms OR "short message*" OR "text message*" OR bulk-messag* OR "bulk messag*" OR mass-messag* OR "mass messag*" OR "public awareness" OR engagement OR information) N3 (campaign* OR strategy OR strategies)) OR "information dissemination") ) 65,870

S24 TI ( ("standard service*" OR standard-service* OR standardized-service* OR standardised-service* OR "standardized service*" OR "standardised service*") ) OR AB ( ("standard service*" OR standard-service* OR standardized-service* OR standardised-service* OR "standardized service*" OR "standardised service*") ) OR SU ( ("standard service*" OR standard-service* OR standardized-service* OR standardised-service* OR "standardized service*" OR "standardised service*") )

. 1,408

S23 TI ( (((service* OR one-stop OR "one stop") N1 (centre* OR center* OR shop*)) OR ((communit* OR community-based) N3 monitor*)) ) OR AB ( (((service* OR one-stop OR "one stop") N1 (centre* OR center* OR shop*)) OR ((communit* OR community-based) N3 monitor*)) ) OR SU ( (((service* OR one-stop OR "one stop") N1 (centre* OR center* OR shop*)) OR ((communit* OR community-based) N3 monitor*)) ) 45,757

S22 TI ( ((("social* accountab*") N2 (mechanism* OR system* OR arrange* OR organi* OR regulat*)) OR (social OR public) N1 audit)) ) OR AB ( ((("social* accountab*") N2 (mechanism* OR system* OR arrange* OR organi* OR regulat*)) OR (social OR public) N1 audit)) ) OR SU ( ((("social* accountab*") N2 (mechanism* OR system* OR arrange* OR organi* OR regulat*)) OR (social OR public) N1 audit)) ) 2,111

S21 TI ( ("report card*" OR reportcard* OR report-card* OR "score card*" OR scorecard* OR score-card* OR "political reserv*" OR "reserved place*" OR "reserved position*" OR "reserved seat*") ) OR AB ( ("report card*" OR reportcard* OR report-card* OR "score card*" OR scorecard* OR score-card* OR "political reserv*" OR "reserved place*" OR "reserved position*" OR "reserved seat*") ) OR SU ( ("report card*" OR reportcard* OR report-card* OR "score card*" OR scorecard* OR score-card* OR "political reserv*" OR "reserved place*" OR "reserved position*" OR "reserved seat*") ) 38,968

S20 TI ( ("e governance" OR e-governance OR egovernance OR "electronic governance" OR (politic* N3 (inclus* OR participat* OR quota OR quotas)) OR (quota* N3 participat*)) ) OR AB ( ("e governance" OR e-governance OR egovernance OR "electronic governance" OR (politic* N3 (inclus* OR participat* OR quota OR quotas)) OR (quota* N3 participat*)) ) OR SU ( ("e governance" OR e-governance OR egovernance OR "electronic governance" OR (politic* N3 (inclus* OR participat* OR quota OR quotas)) OR (quota* N3 participat*)) ) 75,990

S19 TI ( ((((disaster* OR disaster-risk*) N6 (respond* OR response* OR management OR reduc* OR preparedness)) OR DRR) N3 (committee* OR council* OR association* OR shura)) ) OR AB ( ((((disaster* OR disaster-risk*) N6 (respond* OR response* OR management OR reduc* OR preparedness)) OR DRR) N3 (committee* OR council* OR association* OR shura)) ) OR SU ( ((((disaster* OR disaster-risk*) N6 (respond* OR response* OR management OR reduc* OR preparedness)) OR DRR) N3 (committee* OR council* OR association* OR shura)) )

230

S18 TI ( ((communit* OR district* OR cluster OR cluster-level) N6 development N3 (committee* OR council* OR association* OR shura)) ) OR AB ( ((communit* OR district* OR cluster OR cluster-level) N6 development N3 (committee* OR council* OR association* OR shura)) ) OR SU ( ((communit* OR district* OR cluster OR cluster-level) N6 development N3 (committee* OR council* OR association* OR shura)) ) 1,425

S17 TI ( (("natural resource*" OR natural-resource* OR NRM OR "common property" OR common-property OR "common resource*" OR common-resource* OR water-use* OR "water use*" OR "water management" OR water-management OR land-use* OR "land use*" OR land-management OR "land management" OR irrigat*) N6 (participat* OR transparen* OR inclus* OR represent* OR consult* OR community* OR committee* OR council* OR association* OR group* OR shura)) ) OR AB ( (("natural resource*" OR natural-resource* OR NRM OR "common property" OR common-property OR "common resource*" OR common-resource* OR water-use* OR "water use*" OR "water management" OR water-management OR land-use* OR "land use*" OR land-management OR "land management" OR irrigat*) N6 (participat* OR transparen* OR inclus* OR represent* OR consult* OR community* OR committee* OR council* OR association* OR group* OR shura)) ) OR SU ( (("natural resource*" OR natural-resource* OR NRM OR "common property" OR common-property OR "common resource*" OR common-resource* OR water-use* OR "water use*" OR "water management" OR water-management OR land-use* OR "land use*" OR land-management OR "land management" OR irrigat*) N6 (participat* OR transparen* OR inclus* OR represent* OR consult* OR community* OR committee* OR council* OR association* OR group* OR shura)) ) 36,294

S16 TI ( ((health OR healthcare OR hospital*) N3 (committee* OR "action group*" OR council* OR association* OR shura)) ) OR AB ( ((health OR healthcare OR hospital*) N3 (committee* OR "action group*" OR council* OR association* OR shura)) ) OR SU ( ((health OR healthcare OR hospital*) N3 (committee* OR "action group*" OR council* OR association* OR shura)) ) 93,586

S15 TI ( ((inclus* OR particip* ) N6 (strateg* OR action* OR budget* OR development OR plan*)) ) OR AB ( ((inclus* OR particip* ) N6 (strateg* OR action* OR budget* OR development OR plan*)) ) OR SU ( ((inclus* OR particip* ) N6 (strateg* OR action* OR budget* OR development OR plan*)) ) 195,635

S14 TI ( ("community engagement" OR "community consultation*" OR (civic N3 education)) ) OR AB ( ("community engagement" OR "community consultation*" OR (civic N3 education)) ) OR SU ( ("community engagement" OR "community consultation*" OR (civic N3 education)) ) 26,249

S13 TI ( ((communit* OR inclus* OR particip*) N6 (((climate-change OR "climate change") N2 (adapt* OR mitigat* OR vulnerab*)) or resilien*)) ) OR AB ( ((communit* OR inclus* OR particip*) N6 (((climate-change OR "climate change") N2 (adapt* OR mitigat* OR vulnerab*)) or resilien*)) ) OR SU ( ((communit* OR inclus* OR particip*) N6 (((climate-change OR "climate change") N2 (adapt* OR mitigat* OR vulnerab*)) or resilien*)) ) 14,756

S12 TI ( (((disaster* N2 reduc* N2 risk*) OR (disaster* N2 (respond* OR response* OR manag*)) OR ((hazard* OR risk* OR vulnerab*) N2 (map* OR assess*)) OR HVA OR HVRA OR DRR) N6 (participat* OR inclus* OR consult* OR communit*)) ) OR AB ( (((disaster* N2 reduc* N2 risk*) OR (disaster* N2 (respond* OR response* OR manag*)) OR ((hazard* OR risk* OR vulnerab*) N2 (map* OR assess*)) OR HVA OR HVRA OR DRR) N6 (participat* OR inclus* OR consult* OR communit*)) ) OR SU ( (((disaster* N2 reduc* N2 risk*) OR (disaster* N2 (respond* OR response* OR manag*)) OR ((hazard* OR risk* OR vulnerab*) N2 (map* OR assess*)) OR HVA OR HVRA OR DRR) N6 (participat* OR inclus* OR consult* OR communit*)) ) 6,824

S11 S6 OR S7 OR S8 OR S9 OR S10 2,744,933

S10 TI (economic N2 model*) OR AB (economic N2 model*) OR SU (economic N2 model*) 91,429

S9 TI ( ("cost minimi*" OR "cost-utilit*" OR "health utilit*" OR "economic evaluation*" OR "economic review*" OR "cost outcome" OR "cost analys*" OR "economic analys*" OR "budget* impact analys*") ) OR AB ( ("cost minimi*" OR "cost-utilit*" OR "health utilit*" OR "economic evaluation*" OR "economic review*" OR "cost outcome" OR "cost analys*" OR "economic analys*" OR "budget* impact analys*") ) OR SU ( ("cost minimi*" OR "cost-utilit*" OR "health utilit*" OR "economic evaluation*" OR "economic review*" OR "cost outcome" OR "cost analys*" OR "economic analys*" OR "budget* impact analys*") ) 164,977

S8 TI ( (cost-effective* OR cost-benefit OR costs) ) OR AB ( (cost-effective* OR cost-benefit OR costs) ) OR SU ( (cost-effective* OR cost-benefit OR costs) )

2,562,618

S7 TI ( ("life year" OR "life years" OR qaly* OR daly*) ) OR AB ( ("life year" OR "life years" OR qaly* OR daly*) ) OR SU ( ("life year" OR "life years" OR qaly* OR daly*) ) 54,262

S6 TI ( ((cost OR economic*) AND (costs OR cost-effectiveness OR markov)) ) OR AB ( ((cost OR economic*) AND (costs OR cost-effectiveness OR markov)) ) OR SU ( ((cost OR economic*) AND (costs OR cost-effectiveness OR markov)) )

2,300,671

S5 S1 OR S2 OR S3 OR S4 11,769,084

S4 TI ( (lmic or lmics or "third world" or "lami countr*" OR "transitional countr*") ) OR AB ( (lmic or lmics or "third world" or "lami countr*" OR "transitional countr*") ) OR SU ( (lmic or lmics or "third world" or "lami countr*" OR "transitional countr*") ) 27,294

S3 TI ( (((developing or "less* developed" or "under developed" or underdeveloped or "middle income" or "low* income") N1 (economy or economies))) OR ((low* N1 (gdp or gnp or "gross domestic" or "gross national"))) OR ((low N3 middle N3 countr*)) ) OR AB ( (((developing or "less* developed" or "under developed" or underdeveloped or "middle income" or "low* income") N1 (economy or economies))) OR ((low* N1 (gdp or gnp or "gross domestic" or "gross national"))) OR ((low N3 middle N3 countr*)) ) OR SU ( (((developing or "less* developed" or "under developed" or underdeveloped or "middle income" or "low* income") N1 (economy or economies))) OR ((low* N1 (gdp or gnp or "gross domestic" or "gross national"))) OR ((low N3 middle N3 countr*)) ) 60,445

S2 TI ( ("Developing Countries") OR (Africa or Asia or Caribbean or "West Indies" or "South America" or "Latin America" or "Central America") OR (((developing or "less* developed" or "under developed" or underdeveloped or "middle income" or "low* income" or underserved or "under served" or deprived or poor*) N1 (countr* or nation* or population* or world))) ) OR AB ( ("Developing Countries") OR (Africa or Asia or Caribbean or "West Indies" or "South America" or "Latin America" or "Central America") OR (((developing or "less* developed" or "under developed" or underdeveloped or "middle income" or "low* income" or underserved or "under served" or deprived or poor*) N1 (countr* or nation* or population* or world))) ) OR SU ( ("Developing Countries") OR (Africa or Asia or Caribbean or "West Indies" or "South America" or "Latin America" or "Central America") OR (((developing or "less* developed" or "under developed" or underdeveloped or "middle income" or "low* income" or underserved or "under served" or deprived or poor*) N1 (countr* or nation* or population* or world))) ) 3,274,425

S1 TI ( ((Afghanistan or Albania or Algeria or Angola or Argentina or Armenia or Armenian or Aruba or Azerbaijan or Bangladesh or Benin or Byelarus or Byelorussian or Belarus or Belorussian or Belorussia or Belize or Bhutan or Bolivia or Bosnia or Herzegovina or Hercegovina or Botswana or Brasil or Brazil or Bulgaria or "Burkina Faso" or "Burkina Fasso" or "Upper Volta" or Burundi or Urundi or Cambodia or "Khmer Republic" or Kampuchea or Cameroon or Cameroons or Cameron or Camerons or "Cape Verde" or "Central African Republic" or Chad or China or Colombia or Comoros or "Comoro Islands" or Comores or Mayotte or Congo or Zaire or "Costa Rica*" or "Cote d'Ivoire" or "Ivory Coast" or Cuba or Djibouti or "French Somaliland" or Dominica or "Dominican Republic" or "East Timor" or "East Timur" or "Timor Leste" or Ecuador or Egypt or "United Arab Republic" or "El Salvador" or Eritrea or Ethiopia or Fiji or Gabon or "Gabonese Republic" or Gambia or Gaza or "Georgia Republic" or "Georgian Republic" or Ghana or Grenada or Guatemala or Guinea or Guiana or Guyana or Haiti or Hungary or Honduras or India or Maldives or Indonesia or Iran or Iraq or Jamaica or Jordan or Kazakhstan or Kazakh or Kenya or Kiribati or Korea or Kosovo or Kyrgyzstan or Kirghizia or "Kyrgyz Republic" or Kirghiz or Kirgizstan or "Lao PDR" or Laos or Lebanon or Lesotho or Basutoland or Liberia or Libya or Macedonia or Madagascar or "Malagasy Republic" or Malaysia or Malaya or Malay or Sabah or Sarawak or Malawi or Mali or "Marshall Islands" or Mauritania or Mauritius or "Agalega Islands" or Mexico or Micronesia or "Middle East" or Moldova or Moldovia or Moldovian or Mongolia or Montenegro or Morocco or Ifni or Mozambique or Myanmar or Myanma or Burma or Namibia or Nepal or "Netherlands Antilles" or "New Caledonia" or Nicaragua or Niger or Nigeria or Pakistan or Palau or Palestine or Panama or Paraguay or Peru or Philippines or Philipines or Phillipines or Phillippines or "Puerto Ric*" or Romania or Rumania or Roumania or Rwanda or Ruanda or "Saint Lucia" or "St Lucia" or "Saint Vincent" or "St Vincent" or Grenadines or Samoa or "Samoan Islands" or "Navigator Island" or "Navigator Islands" or "Sao Tome" or Senegal or Serbia or Montenegro or Seychelles or "Sierra Leone" or "Sri Lanka" or "Solomon Islands" or Somalia or "South Africa" or Sudan or Suriname or Surinam or Swaziland or Syria or Tajikistan or Tadzhikistan or Tadjikistan or Tadzhik or Tanzania or Thailand or Togo or Togolese Republic or Tonga or Tunisia or Turkey or Turkmenistan or Turkmen or Uganda or Ukraine or Uzbekistan or Uzbek or Vanuatu or "New Hebrides" or Venezuela or Vietnam or "Viet Nam" or "West Bank" or Yemen or Yugoslavia or Zambia or Zimbabwe) NOT ("African-American*" OR "African-American*" OR "Mexican American*" OR "American Indian*" OR "Asian American*" OR "native american*")) ) OR AB ( ((Afghanistan or Albania or Algeria or Angola or Argentina or Armenia or Armenian or Aruba or Azerbaijan or Bangladesh or Benin or Byelarus or Byelorussian or Belarus or Belorussian or Belorussia or Belize or Bhutan or Bolivia or Bosnia or Herzegovina or Hercegovina or Botswana or Brasil or Brazil or Bulgaria or "Burkina Faso" or "Burkina Fasso" or "Upper Volta" or Burundi or Urundi or Cambodia or "Khmer Republic" or Kampuchea or Cameroon or Cameroons or Cameron or Camerons or "Cape Verde" or "Central African Republic" or Chad or China or Colombia or Comoros or "Comoro Islands" or Comores or Mayotte or Congo or Zaire or "Costa Rica*" or "Cote d'Ivoire" or "Ivory Coast" or Cuba or Djibouti or "French Somaliland" or Dominica or "Dominican Republic" or "East Timor" or "East Timur" or "Timor Leste" or Ecuador or Egypt or "United Arab Republic" or "El Salvador" or Eritrea or Ethiopia or Fiji or Gabon or "Gabonese Republic" or Gambia or Gaza or "Georgia Republic" or "Georgian Republic" or Ghana or Grenada or Guatemala or Guinea or Guiana or Guyana or Haiti or Hungary or Honduras or India or Maldives or Indonesia or Iran or Iraq or Jamaica or Jordan or Kazakhstan or Kazakh or Kenya or Kiribati or Korea or Kosovo or Kyrgyzstan or Kirghizia or "Kyrgyz Republic" or Kirghiz or Kirgizstan or "Lao PDR" or Laos or Lebanon or Lesotho or Basutoland or Liberia or Libya or Macedonia or Madagascar or "Malagasy Republic" or Malaysia or Malaya or Malay or Sabah or Sarawak or Malawi or Mali or "Marshall Islands" or Mauritania or Mauritius or "Agalega Islands" or Mexico or Micronesia or "Middle East" or Moldova or Moldovia or Moldovian or Mongolia or Montenegro or Morocco or Ifni or Mozambique or Myanmar or Myanma or Burma or Namibia or Nepal or "Netherlands Antilles" or "New Caledonia" or Nicaragua or Niger or Nigeria or Pakistan or Palau or Palestine or Panama or Paraguay or Peru or Philippines or Philipines or Phillipines or Phillippines or "Puerto Ric*" or Romania or Rumania or Roumania or Rwanda or Ruanda or "Saint Lucia" or "St Lucia" or "Saint Vincent" or "St Vincent" or Grenadines or Samoa or "Samoan Islands" or "Navigator Island" or "Navigator Islands" or "Sao Tome" or Senegal or Serbia or Montenegro or Seychelles or "Sierra Leone" or "Sri Lanka" or "Solomon Islands" or Somalia or "South Africa" or Sudan or Suriname or Surinam or Swaziland or Syria or Tajikistan or Tadzhikistan or Tadjikistan or Tadzhik or Tanzania or Thailand or Togo or Togolese Republic or Tonga or Tunisia or Turkey or Turkmenistan or Turkmen or Uganda or Ukraine or Uzbekistan or Uzbek or Vanuatu or "New Hebrides" or Venezuela or Vietnam or "Viet Nam" or "West Bank" or Yemen or Yugoslavia or Zambia or Zimbabwe) NOT ("African-American*" OR "African-American*" OR "Mexican American*" OR "American Indian*" OR "Asian American*" OR "native american*")) ) OR SU ( ((Afghanistan or Albania or Algeria or Angola or Argentina or Armenia or Armenian or Aruba or Azerbaijan or Bangladesh or Benin or Byelarus or Byelorussian or Belarus or Belorussian or Belorussia or Belize or Bhutan or Bolivia or Bosnia or Herzegovina or Hercegovina or Botswana or Brasil or Brazil or Bulgaria or "Burkina Faso" or "Burkina Fasso" or "Upper Volta" or Burundi or Urundi or Cambodia or "Khmer Republic" or Kampuchea or Cameroon or Cameroons or Cameron or Camerons or "Cape Verde" or "Central African Republic" or Chad or China or Colombia or Comoros or "Comoro Islands" or Comores or Mayotte or Congo or Zaire or "Costa Rica*" or "Cote d'Ivoire" or "Ivory Coast" or Cuba or Djibouti or "French Somaliland" or Dominica or "Dominican Republic" or "East Timor" or "East Timur" or "Timor Leste" or Ecuador or Egypt or "United Arab Republic" or "El Salvador" or Eritrea or Ethiopia or Fiji or Gabon or "Gabonese Republic" or Gambia or Gaza or "Georgia Republic" or "Georgian Republic" or Ghana or Grenada or Guatemala or Guinea or Guiana or Guyana or Haiti or Hungary or Honduras or India or Maldives or Indonesia or Iran or Iraq or Jamaica or Jordan or Kazakhstan or Kazakh or Kenya or Kiribati or Korea or Kosovo or Kyrgyzstan or Kirghizia or "Kyrgyz Republic" or Kirghiz or Kirgizstan or "Lao PDR" or Laos or Lebanon or Lesotho or Basutoland or Liberia or Libya or Macedonia or Madagascar or "Malagasy Republic" or Malaysia or Malaya or Malay or Sabah or Sarawak or Malawi or Mali or "Marshall Islands" or Mauritania or Mauritius or "Agalega Islands" or Mexico or Micronesia or "Middle East" or Moldova or Moldovia or Moldovian or Mongolia or Montenegro or Morocco or Ifni or Mozambique or Myanmar or Myanma or Burma or Namibia or Nepal or "Netherlands Antilles" or "New Caledonia" or Nicaragua or Niger or Nigeria or Pakistan or Palau or Palestine or Panama or Paraguay or Peru or Philippines or Philipines or Phillipines or Phillippines or "Puerto Ric*" or Romania or Rumania or Roumania or Rwanda or Ruanda or "Saint Lucia" or "St Lucia" or "Saint Vincent" or "St Vincent" or Grenadines or Samoa or "Samoan Islands" or "Navigator Island" or "Navigator Islands" or "Sao Tome" or Senegal or Serbia or Montenegro or Seychelles or "Sierra Leone" or "Sri Lanka" or "Solomon Islands" or Somalia or "South Africa" or Sudan or Suriname or Surinam or Swaziland or Syria or Tajikistan or Tadzhikistan or Tadjikistan or Tadzhik or Tanzania or Thailand or Togo or Togolese Republic or Tonga or Tunisia or Turkey or Turkmenistan or Turkmen or Uganda or Ukraine or Uzbekistan or Uzbek or Vanuatu or "New Hebrides" or Venezuela or Vietnam or "Viet Nam" or "West Bank" or Yemen or Yugoslavia or Zambia or Zimbabwe) NOT ("African-American*" OR "African-American*" OR "Mexican American*" OR "American Indian*" OR "Asian American*" OR "native american*")) ) Limiters - Date of Publication: 20000101-20181231

Database - Discovery Service for 3ie, Inc. 10,413,433

4. Scopus – Searched 8th February 2018

( ( TITLE-ABS-KEY ( ( afghanistan OR albania OR algeria OR angola OR argentina OR armenia OR armenian OR aruba OR azerbaijan OR bangladesh OR benin OR byelarus OR byelorussian OR belarus OR belorussian OR belorussia OR belize OR bhutan OR bolivia OR bosnia OR herzegovina OR hercegovina OR botswana OR brasil OR brazil OR bulgaria OR "Burkina Faso" OR "Burkina Fasso" OR "Upper Volta" OR burundi OR urundi OR cambodia OR "Khmer Republic" OR kampuchea OR cameroon OR cameroons OR cameron OR camerons OR "Cape Verde" OR "Central African Republic" OR chad OR china OR colombia OR comoros OR "Comoro Islands" OR comores OR mayotte OR congo OR zaire OR "Costa Rica*" OR "Cote d'Ivoire" OR "Ivory Coast" OR cuba OR djibouti OR "French Somaliland" OR dominica OR "Dominican Republic" OR "East Timor" OR "East Timur" OR "Timor Leste" OR ecuador OR egypt OR "United Arab Republic" OR "El Salvador" OR eritrea OR ethiopia OR fiji OR gabon OR "Gabonese Republic" OR gambia OR gaza OR "Georgia Republic" OR "Georgian Republic" OR ghana OR grenada OR guatemala OR guinea OR guiana OR guyana OR haiti OR hungary OR honduras OR india OR maldives OR indonesia OR iran OR iraq OR jamaica OR jordan OR kazakhstan OR kazakh OR kenya OR kiribati OR korea OR kosovo OR kyrgyzstan OR kirghizia OR "Kyrgyz Republic" OR kirghiz OR kirgizstan OR "Lao PDR" OR laos OR lebanon OR lesotho OR basutoland OR liberia OR libya OR macedonia OR madagascar OR "Malagasy Republic" OR malaysia OR malaya OR malay OR sabah OR sarawak OR malawi OR mali OR "Marshall Islands" OR mauritania OR mauritius OR "Agalega Islands" OR mexico OR micronesia OR "Middle East" OR moldova OR moldovia OR moldovian OR mongolia OR montenegro OR morocco OR ifni OR mozambique OR myanmar OR myanma OR burma OR namibia OR nepal OR "Netherlands Antilles" OR "New Caledonia" OR nicaragua OR niger OR nigeria OR pakistan OR palau OR palestine OR panama OR paraguay OR peru OR philippines OR philipines OR phillipines OR phillippines OR "Puerto Ric*" OR romania OR rumania OR roumania OR rwanda OR ruanda OR "Saint Lucia" OR "St Lucia" OR "Saint Vincent" OR "St Vincent" OR grenadines OR samoa OR "Samoan Islands" OR "Navigator Island" OR "Navigator Islands" OR "Sao Tome" OR senegal OR serbia OR montenegro OR seychelles OR "Sierra Leone" OR "Sri Lanka" OR "Solomon Islands" OR somalia OR "South Africa" OR sudan OR suriname OR surinam OR swaziland OR syria OR tajikistan OR tadzhikistan OR tadjikistan OR tadzhik OR tanzania OR thailand OR togo OR togolese AND republic OR tonga OR tunisia OR turkey OR turkmenistan OR turkmen OR uganda OR ukraine OR uzbekistan OR uzbek OR vanuatu OR "New Hebrides" OR venezuela OR vietnam OR "Viet Nam" OR "West Bank" OR yemen OR yugoslavia OR zambia OR zimbabwe ) ) ) OR ( TITLE-ABS-KEY ( "Developing Countries" OR africa OR asia OR caribbean OR "West Indies" OR "South America" OR "Latin America" OR "Central America" OR ( ( developing OR "less* developed" OR "under developed" OR underdeveloped OR "middle income" OR "low* income" OR underserved OR "under served" OR deprived OR poor* ) W/1 ( countr* OR nation* OR population* OR world ) ) ) ) OR ( TITLE-ABS-KEY ( ( ( developing OR "less* developed" OR "under developed" OR underdeveloped OR "middle income" OR "low* income" ) W/1 ( economy OR economies ) ) OR ( low* W/1 ( gdp OR gnp OR "gross domestic" OR "gross national" ) ) OR ( low W/3 middle W/3 countr* ) ) ) OR ( TITLE-ABS-KEY ( ( ( lmic OR lmics OR "third world" OR "lami countr*" ) ) OR "transitional countr*" ) ) ) AND ( ( ( ( TITLE-ABS-KEY ( ( ( ( disaster* W/2 reduc* W/2 risk* ) OR ( disaster* W/2 ( respond* OR response* OR manag* ) ) OR ( ( hazard* OR risk* OR vulnerab* ) W/2 ( map* OR assess* ) ) OR hva OR hvra OR drr ) W/6 ( participat* OR inclus* OR consult* OR communit* ) ) ) ) OR ( TITLE-ABS-KEY ( ( ( communit* OR inclus* OR particip* ) W/6 ( ( ( climate-change OR "climate change" ) W/2 ( adapt* OR mitigat* OR vulnerab* ) ) OR resilien* ) ) ) ) OR ( TITLE-ABS-KEY ( "community engagement" OR "community consultation*" OR ( civic W/3 education ) ) ) OR ( TITLE-ABS-KEY ( ( ( inclus* OR particip* ) W/6 ( strateg* OR action* OR budget* OR development OR plan* ) ) ) ) OR ( TITLE-ABS-KEY ( ( ( health OR healthcare OR hospital* ) W/3 ( committee* OR "action group*" OR council* OR association* OR shura ) ) ) ) OR ( TITLE-ABS-KEY ( ( ( "natural resource*" OR natural-resource* OR nrm OR "common property" OR common-property OR "common resource*" OR common-resource* OR water-use* OR "water use*" OR "water management" OR water-management OR land-use* OR "land use*" OR land-management OR "land management" OR irrigat* ) W/6 ( participat* OR transparen* OR inclus* OR represent* OR consult* OR community* OR committee* OR council* OR association* OR group* OR shura ) ) ) ) OR ( TITLE-ABS-KEY ( ( ( communit* OR district* OR cluster OR cluster-level ) W/6 development W/3 ( committee* OR council* OR association* OR shura ) ) ) ) ) OR ( ( TITLE-ABS-KEY ( ( ( ( ( disaster* OR disaster-risk* ) W/6 ( respond* OR response* OR management OR reduc* OR preparedness ) ) OR drr ) W/3 ( committee* OR council* OR association* OR shura ) ) ) ) OR ( TITLE-ABS-KEY ( ( "report card*" OR reportcard* OR report-card* OR "score card*" OR scorecard* OR score-card* OR "political reserv*" OR "reserved place*" OR "reserved position*" OR "reserved seat*" ) ) ) OR ( TITLE-ABS-KEY ( ( ( "social* accountab*" W/2 ( mechanism* OR system* OR arrange* OR organi* OR regulat* ) ) OR ( ( social OR public ) W/1 audit ) ) ) ) OR ( TITLE-ABS-KEY ( ( ( ( service* OR one-stop OR "one stop" ) W/1 ( centre* OR center* OR shop* ) ) OR ( ( communit* OR community-based ) W/3 monitor* ) ) ) ) OR ( TITLE-ABS-KEY ( ( "standard service*" OR standard-service* OR standardized-service* OR standardised-service* OR "standardized service*" OR "standardised service*" ) ) ) OR ( TITLE-ABS-KEY ( ( ( ( sms OR "short message*" OR "text message*" OR bulk-messag* OR "bulk messag*" OR mass-messag* OR "mass messag*" OR "public awareness" OR engagement OR information ) W/3 ( campaign* OR strategy OR strategies ) ) OR "information dissemination" ) ) ) ) ) AND ( TITLE-ABS-KEY ( "random* control* trial*" OR "random* trial*" OR rct OR "propensity score matching" OR psm OR "regression discontinuity design" OR rdd OR "difference in difference*" OR did OR difference-in-difference OR evaluat* OR matching OR "interrupted time series" OR ( random* W/3 allocat* ) OR "instrumental variable*" OR iv OR ( ( quantitative OR "comparison group" OR counterfactual OR "counter factual" OR counter-factual OR experiment* OR quasi-experimental OR "quasi experimental" ) W/3 ( design OR study OR analysis ) ) OR qed OR "field experiment" OR "field trial" ) ) ) AND ( LIMIT-TO ( PUBYEAR , 2018 ) OR LIMIT-TO ( PUBYEAR , 2017 ) OR LIMIT-TO ( PUBYEAR , 2016 ) OR LIMIT-TO ( PUBYEAR , 2015 ) OR LIMIT-TO ( PUBYEAR , 2014 ) OR LIMIT-TO ( PUBYEAR , 2013 ) OR LIMIT-TO ( PUBYEAR , 2012 ) OR LIMIT-TO ( PUBYEAR , 2011 ) OR LIMIT-TO ( PUBYEAR , 2010 ) OR LIMIT-TO ( PUBYEAR , 2009 ) OR LIMIT-TO ( PUBYEAR , 2008 ) OR LIMIT-TO ( PUBYEAR , 2007 ) OR LIMIT-TO ( PUBYEAR , 2006 ) OR LIMIT-TO ( PUBYEAR , 2005 ) OR LIMIT-TO ( PUBYEAR , 2004 ) OR LIMIT-TO ( PUBYEAR , 2003 ) OR LIMIT-TO ( PUBYEAR , 2002 ) OR LIMIT-TO ( PUBYEAR , 2001 ) OR LIMIT-TO ( PUBYEAR , 2000 ) ) – 2760 hits

Searches on Health Databases

1. Ovid MEDLINE(R) In-Process & Other Non-Indexed Citations, Ovid MEDLINE(R) Daily and Ovid MEDLINE(R) <1946 to Present> - Searched 5th February 2018

1 ((Afghanistan or Albania or Algeria or Angola or Argentina or Armenia or Armenian or Aruba or Azerbaijan or Bangladesh or Benin or Byelarus or Byelorussian or Belarus or Belorussian or Belorussia or Belize or Bhutan or Bolivia or Bosnia or Herzegovina or Hercegovina or Botswana or Brasil or Brazil or Bulgaria or "Burkina Faso" or "Burkina Fasso" or "Upper Volta" or Burundi or Urundi or Cambodia or "Khmer Republic" or Kampuchea or Cameroon or Cameroons or Cameron or Camerons or "Cape Verde" or "Central African Republic" or Chad or China or Colombia or Comoros or "Comoro Islands" or Comores or Mayotte or Congo or Zaire or "Costa Rica*" or "Cote d'Ivoire" or "Ivory Coast" or Cuba or Djibouti or "French Somaliland" or Dominica or "Dominican Republic" or "East Timor" or "East Timur" or "Timor Leste" or Ecuador or Egypt or "United Arab Republic" or "El Salvador" or Eritrea or Ethiopia or Fiji or Gabon or "Gabonese Republic" or Gambia or Gaza or "Georgia Republic" or "Georgian Republic" or Ghana or Grenada or Guatemala or Guinea or Guiana or Guyana or Haiti or Hungary or Honduras or India or Maldives or Indonesia or Iran or Iraq or Jamaica or Jordan or Kazakhstan or Kazakh or Kenya or Kiribati or Korea or Kosovo or Kyrgyzstan or Kirghizia or "Kyrgyz Republic" or Kirghiz or Kirgizstan or "Lao PDR" or Laos or Lebanon or Lesotho or Basutoland or Liberia or Libya or Macedonia or Madagascar or "Malagasy Republic" or Malaysia or Malaya or Malay or Sabah or Sarawak or Malawi or Mali or "Marshall Islands" or Mauritania or Mauritius or "Agalega Islands" or Mexico or Micronesia or "Middle East" or Moldova or Moldovia or Moldovian or Mongolia or Montenegro or Morocco or Ifni or Mozambique or Myanmar or Myanma or Burma or Namibia or Nepal or "Netherlands Antilles" or "New Caledonia" or Nicaragua or Niger or Nigeria or Pakistan or Palau or Palestine or Panama or Paraguay or Peru or Philippines or Philipines or Phillipines or Phillippines or "Puerto Ric*" or Romania or Rumania or Roumania or Rwanda or Ruanda or "Saint Lucia" or "St Lucia" or "Saint Vincent" or "St Vincent" or Grenadines or Samoa or "Samoan Islands" or "Navigator Island" or "Navigator Islands" or "Sao Tome" or Senegal or Serbia or Montenegro or Seychelles or "Sierra Leone" or "Sri Lanka" or "Solomon Islands" or Somalia or "South Africa" or Sudan or Suriname or Surinam or Swaziland or Syria or Tajikistan or Tadzhikistan or Tadjikistan or Tadzhik or Tanzania or Thailand or Togo or Togolese Republic or Tonga or Tunisia or Turkey or Turkmenistan or Turkmen or Uganda or Ukraine or Uzbekistan or Uzbek or Vanuatu or "New Hebrides" or Venezuela or Vietnam or "Viet Nam" or "West Bank" or Yemen or Yugoslavia or Zambia or Zimbabwe) not ("African-American*" or "African-American*" or "Mexican American*" or "American Indian*" or "Asian American*" or "native american*")).ti,ab,hw. (1284842)

2 ("Developing Countries" or Africa or Asia or Caribbean or "West Indies" or "South America" or "Latin America" or "Central America" or ((developing or "less* developed" or "under developed" or underdeveloped or "middle income" or "low* income" or underserved or "under served" or deprived or poor*) adj1 (countr* or nation* or population* or world))).ti,ab,hw,kw. (324042)

3 (((developing or "less* developed" or "under developed" or underdeveloped or "middle income" or "low* income") adj1 (economy or economies)) or (low* adj1 (gdp or gnp or "gross domestic" or "gross national")) or (low adj3 middle adj3 countr*)).ti,ab,hw,kw. (9725)

4 (lmic or lmics or "third world" or "lami countr*" or "transitional countr*").ti,ab,hw,kw. (5281)

5 or/1-4 (1436981)

6 ("random* control* trial*" or "random* trial*" or RCT or "propensity score matching" or PSM or "regression discontinuity design" or RDD or "difference in difference*" or DID or difference-in-difference or evaluat* or matching or "interrupted time series" or (random* adj3 allocat*) or "instrumental variable*" or IV or ((quantitative or "comparison group" or counterfactual or "counter factual" or counter-factual or experiment* or quasi-experimental or "quasi experimental") adj3 (design or study or analysis)) or QED or "field experiment" or "field trial").ti,ab,hw,kw. (5339673)

7 exp Randomized Controlled Trial/ or Randomized Controlled Trials as Topic/ or random allocation/ or Propensity Score/ or Quasi-Experimental Studies/ or Controlled Before-After Studies/ or Interrupted Time Series Analysis/ (641694)

8 6 or 7 (5341090)

9 (((communit* or village* or stakeholder*) adj3 (engag* or consult* or meeting* or outreach* or represent* or participat* or network)) or (civic adj3 education) or audit or "social responsibility" or (moral* adj2 obligat*)).ti,ab,hw,kw. (101640)

10 Community Participation/ or Community Networks/ or Stakeholder Participation/ or Social responsibility/ or Moral Obligations/ or Management Audit/ (45973)

11 ((inclus* or participat*) adj6 (strateg* or action* or budget* or plan*)).ti,ab,hw,kw. (11800)

12 ((health or healthcare or hospital* or women* or communit*) adj3 (committee* or "action group*" or council* or association* or shura)).ti,ab,hw,kw. (30165)

13 ((((disaster* or disaster-risk*) adj6 (respond* or response* or management or reduc* or preparedness)) or DRR) adj3 (committee* or council* or association* or shura)).ti,ab,hw,kw. (26)

14 ("e governance" or e-governance or egovernance or "electronic governance").ti,ab,hw,kw. (10)

15 ("report card*" or reportcard* or report-card* or "score card*" or scorecard* or score-card*).ti,ab,hw,kw. (2444)

16 ((accountab* adj2 (mechanism* or system* or arrange* or organi* or regulat*)) or ((social or public) adj1 audit) or ((communit* or community-based) adj3 monitor*)).ti,ab,hw,kw. (3468)

17 ((service* or one-stop or "one stop") adj1 (centre* or center* or shop*)).ti,ab,hw,kw. (2194)

18 ("standard service*" or standard-service* or standardized-service* or standardised-service* or "standardized service*" or "standardised service*").ti,ab,hw,kw. (171)

19 Mass Media/ or Electronic Mail/ or Internet/ or Text Messaging/ or Communication/ or Health promotion/ or Consumer Health Information/ or Information Dissemination/ (219065)

20 ((((sms or "short message*" or "text message*" or bulk-messag* or "bulk messag*" or mass-messag* or "mass messag*" or "public awareness" or engagement or information or "mass media" or email or e-mail or "electronic mail" or internet or communicat*) adj3 (campaign* or strategy or strategies)) or "health promot*" or "consumer health information" or (information* adj2 disseminat*)) and (entitle* or rights or (health adj1 (service* or provider*) adj3 performance*) or "service provision")).ti,ab,hw,kw. (1652)

21 or/9-20 (361338)

22 5 and 8 and 21 (10890)

23 limit 22 to yr="2000 -Current" (9117)

24 exp health facilities/ or Delivery of Health Care/ or Regional Health Planning/ (781442)

25 (hospital or hospitals or infirmary or infirmaries or clinic or clinics or ((health or medical) adj (centre* or center* or facilit*)) or (deliver* adj2 ("health care" or healthcare or "health service*")) or (plan* adj2 region* adj2 health)).ti,ab,hw,kw. (1585431)

26 24 or 25 (1775217)

27 23 and 26 (2759)

2. Global Health (Ovid) <1910 to 2018 Week 04> Searched 8th February 2018

1 exp africa/ (228694)

2 exp Central America/ or exp Latin America/ or exp South America/ (169107)

3 mexico/ (20917)

4 exp central asia/ (11758)

5 east asia/ or china/ or korea democratic people's republic/ or korea republic/ or mongolia/ (221537)

6 exp south asia/ or himalaya/ (141050)

7 exp south east asia/ or pacific rim/ (91596)

8 exp caribbean/ (21088)

9 exp pacific islands/ (13988)

10 exp developing countries/ (841574)

11 (Africa or Asia or Caribbean or West Indies or South America or Latin America or Central America).tw. (949502)

12 (Afghanistan or Albania or Algeria or Angola or Argentina or Armenia or Armenian or Azerbaijan or Bangladesh or Benin or Byelarus or Byelorussian or Belarus or Belorussian or Belorussia or Belize or Bhutan or Bolivia or Bosnia or Herzegovina or Hercegovina or Botswana or Brazil or Bulgaria or Burkina Faso or Burkina Fasso or Upper Volta or Burundi or Urundi or Cambodia or Khmer Republic or Kampuchea or Cameroon or Cameroons or Cameron or Camerons or Cape Verde or Central African Republic or Chad or China or Colombia or Comoros or Comoro Islands or Comores or Mayotte or Congo or Zaire or Costa Rica or Cote d'Ivoire or Ivory Coast or Cuba or Djibouti or French Somaliland or Dominica or Dominican Republic or East Timor or East Timur or Timor Leste or Ecuador or Egypt or United Arab Republic or El Salvador or Eritrea or Ethiopia or Fiji or Gabon or Gabonese Republic or Gambia or Gaza or Georgia Republic or Georgian Republic or Ghana or Grenada or Guatemala or Guinea or Guiana or Guyana or Haiti or Honduras or India or Maldives or Indonesia or Iran or Iraq or Jamaica or Jordan or Kazakhstan or Kazakh or Kenya or Kiribati or Korea or Kosovo or Kyrgyzstan or Kirghizia or Kyrgyz Republic or Kirghiz or Kirgizstan or Lao PDR or Laos or Lebanon or Lesotho or Basutoland or Liberia or Libya or Macedonia or Madagascar or Malagasy Republic or Malaysia or Malaya or Malay or Sabah or Sarawak or Malawi or Mali or Marshall Islands or Mauritania or Mauritius or Agalega Islands or Mexico or Micronesia or Middle East or Moldova or Moldovia or Moldovian or Mongolia or Montenegro or Morocco or Ifni or Mozambique or Myanmar or Myanma or Burma or Namibia or Nepal or Netherlands Antilles or Nicaragua or Niger or Nigeria or Muscat or Pakistan or Palau or Palestine or Panama or Paraguay or Peru or Philippines or Philipines or Phillipines or Phillippines or Papua New Guinea or Romania or Rumania or Roumania or Rwanda or Ruanda or Saint Lucia or St Lucia or Saint Vincent or St Vincent or Grenadines or Samoa or Samoan Islands or Navigator Island or Navigator Islands or Sao Tome or Senegal or Serbia or Montenegro or Seychelles or Sierra Leone or Sri Lanka or Solomon Islands or Somalia or Sudan or Suriname or Surinam or Swaziland or South Africa or Syria or Tajikistan or Tadzhikistan or Tadjikistan or Tadzhik or Tanzania or Thailand or Togo or Togolese Republic or Tonga or Tunisia or Turkey or Turkmenistan or Turkmen or Uganda or Ukraine or Uzbekistan or Uzbek or Vanuatu or New Hebrides or Venezuela or Vietnam or Viet Nam or West Bank or Yemen or Zambia or Zimbabwe).tw. (927670)

13 ((developing or less* developed or under developed or underdeveloped or middle income or low* income or underserved or under served or deprived or poor*) adj (countr* or nation? or population? or world or state*)).ti,ab. (46335)

14 ((developing or less* developed or under developed or underdeveloped or middle income or low* income) adj (economy or economies)).ti,ab. (235)

15 (low* adj (gdp or gnp or gross domestic or gross national)).tw. (64)

16 (low adj3 middle adj3 countr*).tw. (5149)

17 (lmic or lmics or third world or lami countr*).tw. (20053)

18 transitional countr*.tw. (90)

19 or/1-18 (1070258)

20 ("random* control* trial*" or "random* trial*" or RCT or "propensity score matching" or PSM or "regression discontinuity design" or RDD or "difference in difference*" or DID or difference-in-difference or evaluat* or matching or "interrupted time series" or (random* adj3 allocat*) or "instrumental variable*" or IV or ((quantitative or "comparison group" or counterfactual or "counter factual" or counter-factual or experiment* or quasi-experimental or "quasi experimental") adj3 (design or study or analysis)) or QED or "field experiment" or "field trial").ti,ab,hw. (862995)

21 randomized controlled trials/ or meta-analysis/ or systematic reviews/ or regression analysis/ or time series/ or quantitative analysis/ or experimental design/ or "controls (experimental)"/ or field experimentation/ (71778)

22 or/20-21 (880676)

23 (((communit* or village* or stakeholder*) adj3 (engag* or consult* or meeting* or outreach* or represent* or participat* or network)) or (civic adj3 education) or audit or "social responsibility" or (moral* adj2 obligat*)).ti,ab,hw. (13885)

24 social participation/ or public participation/ or personal support networks/ or stakeholders/ or moral values/ or auditing/ or monitoring/ (33527)

25 ((inclus* or participat*) adj6 (strateg* or action* or budget* or plan*)).ti,ab,hw. (2748)

26 ((health or healthcare or hospital* or women* or communit*) adj3 (committee* or "action group*" or council* or association* or shura)).ti,ab,hw. (8871)

27 ((((disaster* or disaster-risk*) adj6 (respond* or response* or management or reduc* or preparedness)) or DRR) adj3 (committee* or council* or association* or shura)).ti,ab,hw. (8)

28 ("e governance" or e-governance or egovernance or "electronic governance").ti,ab,hw. (6)

29 ("report card*" or reportcard* or report-card* or "score card*" or scorecard* or score-card*).ti,ab,hw. (421)

30 ((accountab* adj2 (mechanism* or system* or arrange* or organi* or regulat*)) or ((social or public) adj1 audit) or ((communit* or community-based) adj3 monitor*)).ti,ab,hw. (802)

31 ((service* or one-stop or "one stop") adj1 (centre* or center* or shop*)).ti,ab,hw. (576)

32 ("standard service*" or standard-service* or standardized-service* or standardised-service* or "standardized service*" or "standardised service*").ti,ab,hw. (44)

33 ((((sms or "short message*" or "text message*" or bulk-messag* or "bulk messag*" or mass-messag* or "mass messag*" or "public awareness" or engagement or information or "mass media" or email or e-mail or "electronic mail" or internet or communicat*) adj3 (campaign* or strategy or strategies)) or "health promot*" or "consumer health information" or (information* adj2 disseminat*)) and (entitle* or rights or (health adj1 (service* or provider*) adj3 performance*) or "service provision")).ti,ab,hw. (474)

34 mass media/ or internet/ or web sites/ or communication/ or health promotion/ or consumer information/ or "diffusion of information"/ or campaigns/ or government campaigns/ or civil rights/ or human rights/ (38138)

35 or/23-34 (93201)

36 hospitals/ or hospices/ or health centres/ or health clinics/ or health services/ or community health services/ (96386)

37 (hospital or hospitals or infirmary or infirmaries or clinic or clinics or ((health or medical) adj (centre* or center* or facilit*)) or (deliver* adj2 ("health care" or healthcare or "health service*")) or (plan* adj2 region* adj2 health)).ti,ab,hw. (305852)

38 or/36-37 (344538)

39 19 and 22 and 35 and 38 (3006)

40 limit 39 to yr="2000 -Current" (2709)

Appendix 2: Search record for online repositories

| ID | Site name | URL | Search start | Search end | Num. hits | Num. studies included in in-depth assessment |
| --- | --- | --- | --- | --- | --- | --- |
| 1 | EGAP (Experiments in Governance and Politics) | http://egap.org/biblio | 05/03/18 | 05/03/18 | 233 | 3 |
| 2 | World Bank Open Knowledge Repository | https://openknowledge.worldbank.org/ | 06/03/18 | 08/03/18 | 2106 | 11 |
| 3 | 3ie Repository of Impact Evaluations | http://www.3ieimpact.org/en/evidence/impact-evaluations/ | 08/03/18 | 12/03/18 | 566 | 17 |
| 4 | Innovations for Poverty Action (IPA) | http://www.poverty-action.org/search-studies | 13/03/18 | 13/03/18 | 47 | 6 |
| 5 | J-Poverty Action Lab (J-PAL) | https://www.povertyactionlab.org/evaluations | 13/03/18 | 13/03/18 | 147 | 11 |
| 6 | Design, Monitoring and Evaluation for Peace | www.dmeforpeace.org/learn/resources/ | 13/03/18 | 13/03/18 | 35 | 0 |
| 7 | United Nations Evaluation Group | http://www.uneval.org/evaluation/reports | 13/03/18 | 13/03/18 | 0 | 0 |
| 8 | Oxfam International | https://policy-practice.oxfam.org.uk/publications | 19/03/18 | 19/03/18 | 19 | 2 |
| 9 | CARE International | http://www.careevaluations.org/ | 19/03/18 | 19/03/18 | 7 | 0 |
| 10 | Mercy Corps | https://www.mercycorps.org/research | 19/03/18 | 19/03/18 | 5 | 0 |
| 11 | Catholic Relief Services | https://www.crs.org/our-work-overseas/research-publications | 20/03/18 | 20/03/18 | 1 | 0 |
| 12 | DFID Research for Development (R4D) | http://r4d.dfid.gov.uk/ | 20/03/18 | 20/03/18 | 79 | 1 |
| 13 | IDEAS / REPEC | https://ideas.repec.org/ | 20/03/18 | 21/03/18 | 476 | 2 |
| 14 | BREAD | http://ibread.org/bread/papers | 21/03/18 | 21/03/18 | 16 | 0 |
| 15 | Locus (International Development Coalition) | https://locus.ngo/resources | 21/03/18 | 21/03/18 | 1 | 0 |
| 16 | GEF (Global Environmental Facility) evaluation database | http://www.gefieo.org/evaluations/all?f[0]=field_ieo_grouping%3A312 | 27/03/18 | 27/03/18 | 34 | 0 |
| 17 | Global Facility for Disaster Reduction and Recovery | https://www.gfdrr.org/en/publications | 28/03/18 | 28/03/18 | 0 | 0 |
| 18 | Samuel Hall (evaluations) | http://samuelhall.org/category/publications/ | 28/03/18 | 28/03/18 | 0 | 0 |
| 19 | IFPRI | http://www.ifpri.org/publications | 28/03/18 | 28/03/18 | 6 | 0 |
| 20 | LSE ICG | https://www.theigc.org/search/?select-post_type%5B%5D=publication | 28/03/18 | 28/03/18 | 0 | 0 |
| 21 | 3ie RIDIE (Registry for International Development Impact Evaluations) | http://ridie.3ieimpact.org/ | 28/03/18 | 28/03/18 | 8 | 1 |
| 22 | Open Governance Partnership | https://www.opengovpartnership.org/resources/all-resources | 28/03/18 | 28/03/18 | 0 | 0 |
| 23 | CGIAR: Consultative Group on International Agricultural Research | https://cgspace.cgiar.org/handle/10568/83389 | 28/03/18 | 28/03/18 | 299 | 0 |
| 24 | Asian Development Bank (ADB) | https://www.adb.org/publications | 28/03/18 | 28/03/18 | 31 | 0 |
| 25 | Center for Effective Global Action (CEGA) | http://cega.berkeley.edu/evidence/ | 28/03/18 | 28/03/18 | 34 | 1 |
| 26 | ICNL Research Centre | http://www.icnl.org/research/library/ol/ | 28/03/18 | 28/03/18 | 0 | 0 |
| 27 | RTI International | https://www.rti.org/publications | 28/03/18 | 28/03/18 | 15 | 0 |
| 28 | Chemonics International | https://www.chemonics.com/technical-areas/democracy-and-governance/ | 28/03/18 | 28/03/18 | 3 | 0 |
| 29 | USAID Development Clearing House | https://dec.usaid.gov/dec/home/Default.aspx | 28/03/18 | 28/03/18 | 36 | 0 |
| 30 | Inter-American Development Bank Publications | https://publications.iadb.org/facet-view?locale-attribute=en&field=type_view | 29/03/18 | 29/03/18 | 63 | 1 |
| 31 | African Development Bank (AfDB) | https://www.afdb.org/en/documents/publications/ | 29/03/18 | 29/03/18 | 23 | 0 |
| 32 | AgEcon | https://ageconsearch.umn.edu/?ln=en | 29/03/18 | 29/03/18 | 79 | 0 |
| 33 | Prevention Web (UNIDSR) | https://www.preventionweb.net/english/professional/ | 29/03/18 | 29/03/18 | 111 | 1 |
| 34 | AGRIS | http://agris.fao.org/agris-search/index.do | 29/03/18 | 29/03/18 | 220 | 1 |
| 35 | Transparency International (TI) | https://www.transparency.org/ | 29/03/18 | 29/03/18 | 0 | 0 |
| 36 | U4 Anti-Corruption Resource Centre | http://www.u4.no/publications/ | 29/03/18 | 29/03/18 | 0 | 0 |
| 37 | Centre for Public Impact | https://www.centreforpublicimpact.org/observatory/ | 29/03/18 | 29/03/18 | 11 | 0 |
| 38 | World Vision | http://www.wvi.org/resources | 29/03/18 | 29/03/18 | 123 | 0 |
| 39 | IRC | https://www.rescue.org/reports-and-resources | 29/03/18 | 29/03/18 | 0 | 0 |
| 40 | Independent Development Evaluation, AfDB | http://idev.afdb.org/en/page/evaluations | 29/03/18 | 29/03/18 | 2 | 1 |

Appendix 3: Data extraction forms

Study characteristics coding tool

The study descriptive characteristics were extracted using the survey tool KoBo Toolbox. The full survey tool will be available in the online Annexes. The question headings are presented here:

1: Report Identification

Unique study ID

First author and short title

Other papers used for coding

Study publication date

Publication type

Funding agency type(s)

Funding agency name(s)

Independence of the evaluation

Independent data collection

Conflict of interest

Comments on conflict of interest

Ethical clearance

Name of Ethics Board reviewing and clearance number

Language of publication

Other methods

Any other relevant information you'd like to add for this section?

2: Context

Country

Detailed location

World Bank region

WB income category

Country performance - governance indicators

Any other relevant information you'd like to add for this section?

3: Intervention Descriptives

Programme or project name

Intervention type

PITA type(s)

Intervention sector

Intervention description

Objectives of the intervention

Intervention scale

Intervention development

Intervention implementing agency

Intervention funding agency type

Intervention funding agency name

Intervention target group

Targeting methods

Intervention start date

Intervention end date

Any other relevant information you'd like to add for this section?

4: Equity

Consideration of equity

Equity methods

Equity dimensions

Any other relevant information you'd like to add for this section?

5: Process and Implementation

Information about programme take-up (among participants)

Methods of assessing take-up

Results of the assessment of take-up

Information about programme adherence (among participants)

Methods of assessing adherence

Results of the assessment of adherence

Information about implementation fidelity / intervention delivery quality

Methods of assessing implementation fidelity

Results of the assessment of intervention fidelity

Other description of process / implementation factors

6: Contextual Barriers /Facilitators

Causal mechanisms / barriers and facilitators

Methods of identifying causal mechanisms / barriers and facilitators

Results of identifying causal mechanisms / barriers and facilitators

Any other relevant information you'd like to add for this section?

7: Cost

Are any unit cost data / cost-effectiveness estimates provided?

If yes, please list the page numbers where this is reported

Any other relevant information you'd like to add for this section?

8: External Validity

Study length

Efficacy or effectiveness trial

Personnel implementing the programme

Sampling frame for the study

Author discussion of external validity

If yes, please summarize the external validity discussion

Programme theory discussed?

Report any description/statement of programme theory as stated by author(s).

Is the study using theory to inform the evaluation design and/or analysis?

Any other relevant information you'd like to add for this section?

9: Study Design

Does the study include multiple study arms?

If so, do the study arms involve different study designs?

Please create a unique identifier for each study arm

Primary study design

Corresponding study arm(s)

Additional study design

Corresponding study arm(s)

Further study designs

Methods used for analysis

Methods used in each study arm

Design and analysis method description

Unit of analysis (UoA)

Method used to address differences between UoA and unit of data collection

Type of comparison group

Comparison group description

Any other relevant information

10: Blinding

Blinded participants

Blinded observers

Blinded analysts

Method used to blind

Any other relevant information

11: Outcomes

For this section, answer all questions (as relevant) for each outcome reported

Outcome

Corresponding study arm(s)

Definition of outcome

Follow up period

Sub-group analysis

Sub-group analysis decription

Location of effect size data

Mechanisms and thematic information

To synthesise the mechanisms, moderators, and other explanatory factors, data including first, second and third order constructs were extracted from included studies and corresponding additional documents using the following prompts:

Study ID, identifier // Region // Country // Country income level // Democracy policy index score

Intervention designer // Study frame // Level of society at which change targeted // Sector

Demand-side type of participants targeted // Supply-side type of participants targeted

Demand-side participants actively engaged // Supply-side participants actively engaged

Underlying bottleneck or barrier project aims to address

Bottleneck identification - how was the bottleneck/problem identified?

What evidence is presented for the existence of the bottleneck locally?

What evidence is presented that the problem identified is the key problem for addressing the overarching issue?

Actors targeted by intervention // Initial power difference // Onus for change // Type of change

Do targeted supply-side actors have the authority or capacity to influence outcomes desired?

Intervention Type // Intervention Description // PITA characteristic(s) // Intensity of PITA change

Strategic (complex) or tactical (tool based) - degree of flexibility/adaptability of intervention

Implemented in isolation or as integrated programme // Institutionalized or one-off intervention

Support provided by implementer to actors whose behaviours are targeted for change

Information on take-up (did targeted people actually participate?) // Implementation fidelity

Evidence on buy-in from above, at, and below targeted actors // Implementation quality

Barriers/Facilitators assessed by the authors

Moderators (barriers/facilitators) and mechanisms identified through synthesis, to outcomes of:

Citizen engagement

Provider response

Provision and quality of services

Use of services

Attitudes about services

Sustainability

Income / poverty status

Health

Nutritional status / food security

Human resilience

Environmental

Social / psychological

State - society relations

Risk of bias coding tool for randomized studies

| Question | Coding | Criteria and decision-rules | |
| --- | --- | --- | --- |
| Unique study identification # | For example, PITA001 |  |  |
| Paper :  Surname, year of first author of paper for effect size data extraction | Open answer |  |  |
| Outcome | Open answer |  |  |
| Outcome description | Open answer | Write more information on the outcome and how it was measured. | |
| Design type :  What type of study design is used? | 1= Randomised controlled trial (RCT) (random assignment to households/individuals) or quasi-RCT 2= Cluster-RCT (quasi-RCT) |  |  |
| Methods used for analysis :  Which methods are used to control for selection bias and confounding? | 1= Statistical matching (PSM, CEM, covariate matching) 2= Difference in differences (DID) estimation methods 3= IV-regression (2-stage least squares or bivariate probit) 4=Heckman selection model 5= Fixed effects regression 6= Covariate adjusted estimation 7= Propensity weighted regression 8= Comparison of means 9 = Other |  |  |
| Design and analysis method description | Open answer | Briefly describe the study design and analysis method undertaken by the authors | |
| Unit of analysis :  Is unit of analysis in cluster allocation addressed in standard error calculation (RCT and NRS)? | 1=Yes 2=No 3=Not reported/unclear 4=Not applicable | -Score "Yes" if UoA = UoR OR if UoA != UoR and standard erros are clustered at the UoR level OR data are collapsed to the UoR level  -Score "Not reported/unclear" if not enough information is provided on the way the standard errors were calculated or what the unit of analysis is.  -Score "Not applicable" if it is not a cluster RCT.  -Score "No" otherwise. | |
| Method used to address differences between UoA and unit of data collection | Open answer | Briefly describe methods used to adjust standard errors to account for correlation of observations within clusters (e.g. cluster-robust standard errors reported). Unit of analysis (UoA) is the unit of observation and unit of randomization (UoR) is the unit of assignment to control or treatment groups. | |
| Type of comparison group | 1=No intervention (service delivery as usual) 2=Other intervention comprising PITA mechanism(s) 3=Pipeline (wait-list) control (still service delivery as usual) 8=Other | Indicate type of comparison group | |
| Type of comparison group (if other) | Open answer |  |  |
| Assignment mechanism :  Was the allocation or identification mechanism random or as good as random? | 1= Yes, 2 = Probably Yes, 3 = Probably No, 4 = No, 8 = Unclear | a) The authors describe a random component in sequence generation/ randomization method (e.g. lottery, coin toss, random number generator) and assignment is performed for all units at the start of the study centrally or using a method concealed from participants and intervention delivery.  b) If public lottery is used for the sequence generation, authors provide detail on the exact settings and participants attending the lottery. c) If a special randomization procedure is used to ensure balance, it is well described and justified given the study setting (stratification, pairwise matching, unique random draw, multiple random draws etc.).  d) A balance table is reported suggesting that allocation was random between all groups including subgroup receiving different treatment within control or treatment groups (if the comparison is relevant for this assessment). | -Score “Yes” if all criterion a), b), c) and d) are satisfied.  -Score "Probably Yes" if only criterion a) and b) are not satisfied OR if only criteria c) is not satisfied.  -Score “Unclear” if d) is not satisfied because no balance table is reported.  -Score "Probably No" if d) is not satisfied because there is no balance table reported and there is evidence suggesting a problem in the randomization, such as baseline coefficients in a diff-in-diff regression table are very different or sample size is too small for the procedure used (using stratification when there are less than two units for each intervention and control group in each strata can lead to imbalance).  -Score “No” if d) is not satisfied because there are large imbalances concerning a large number of variables, providing evidence that the assignment was not random. If this is scored as no, use the NRS tool. |
| Assignment justification | Open answer | Justification for coding decision (Include a brief summary of justification for rating, mentioning your response to all sub questions, cite relevant pages). | |
| Confounding :  Was the method of analysis executed adequately to ensure comparability of groups throughout the study and prevent confounding? | 1= Yes, 2 = Probably Yes, 3 = Probably No, 4 = No, 8 = Unclear | a) Baseline characteristics are similar in magnitude;  b) Unbalanced covariates at the individual and cluster level are controlled in adjusted analysis;  c) Adjustments to the randomization were taken into account in the analysis (stratum fixed effects, pairwise matching variables)? (Bruhn and McKenzie 2009) | -Score “Yes” if criterion a) and b) are satisfied; -Score "Probably yes" if a) is not satisfied but b) is satisfied and imbalances are small in magnitude OR if only a) is satisfied.  -Score “Unclear” if no balance table is provided or if imbalances are controlled for but they are very large in magnitude and assignment mechanism is not coded as "Yes" or "Probably yes".  -Score "Probably no" if a) and b) are not satisfied and the magnitude of imbalances are small.  -Score “No” if a) and b) are not satisfied and the magnitude of imbalances are large and covariates are clear determinant of the outcomes. |
| Confounding justification | Open answer | Justification for coding decision (Include a brief summary of justification for rating, mentioning your response to all sub questions, cite relevant pages). | |
| Selection bias :  Was any differential selection into or out of the study (attrition bias) adequately resolved? | 1= Yes, 2 = Probably Yes, 3 = Probably No, 4 = No, 8 = Unclear | -Score "Yes" if there is no attrition or it is less than 10 per cent and the study establishes that attrition is randomly distributed (e.g. by presenting balance by key characteristics across groups) AND if survey respondents were randomly sampled.  -Score "Probably yes" if there is less than 20 per cent attrition and the study establishes that attrition is randomly distributed (e.g. by presenting balance by key characteristics across groups) AND if survey respondents were randomly sampled.  -Score "Unclear" if there is an attrition problem but no information provided on the relationship between attrition and treatment status, OR if there is not enough information on how the population surveyed was sampled.  -Score "Probably no" if there is attrition which is likely to be related to the intervention OR there is some indication that the survey respondents were purposely sampled in a way that might have led the sampling to be different between treatment and control groups.  -Score "No" if there is evidence of differential attrition affecting more than 20 per cent of the data. | |
| Selection bias justification | Open answer | Justification for coding decision (Include a brief summary of justification for rating, mentioning your response to all sub questions, cite relevant pages). | |
| Deviations from intended interventions :  Was the study adequately protected against spill-overs, cross-overs and contamination? | 1= Yes, 2 = Probably Yes, 3 = Probably No, 4 = No, 8 = Unclear | a) There was no implementation issues that might have led the control participants to receive the treatment (implementer's mistake).  b) The intervention is unlikely to spill-over to comparisons (e.g. participants and non-participants are geographically and/or socially separated from one another and general equilibrium effects are not likely) or the potential effects of spill-overs were measured (e.g. variation in the % of unit within a cluster receiving the treatment).  c) There is no risk of contamination by external programs: the treatment and comparisons are isolated from other interventions which might explain changes in outcomes.  d) There is nothing in the surveys that might have given the control participants an idea of what the other group might receive OR they did but there is no risk that this has changed their behaviours; AND the survey process did not reveal information to the control group that they did not have before (e.g. the study aims to measure increase in take up of a service or product that participants might not know about) Authors might put something in place in the design of the study that allows to control for that survey effect (e.g. a pure control with no monitoring except baseline end-line) | -Score “Yes” if criterion a), b), c) and d) are satisfied;  -Score "Probably yes" if there is no obvious problem but there is no information reported on potential risks related to spillovers, contamination, or survey effects in the control group OR if htere were issus with spill-overs but they were contolled for or measured.  -Score “Unclear” if spill-overs, cross-overs, survey effects and/or contamination are not addressed clearly.  -Score "Probably no" if any of the criterion a), b), c) or d) are not satisfied but the scale of the issue is not clear.  -Score “No” if any of the criterion a), b), c) or d) are not satisfied and happened at a large scale in the study. |
| Deviations justification | Open answer | Justification for coding decision (Include a brief summary of justification for rating, mentioning your response to all sub questions, cite relevant pages). | |
| Performance bias :  Was the process of monitoring individuals unlikely to introduce motivation bias among participants? | 1= Yes, 2 = Probably Yes, 3 = Probably No, 4 = No, 8 = Unclear | a) The authors state explicitly that the process of monitoring the intervention and outcome measurement is blinded and conducted in the same frequency for treatment and control groups, or argue convincingly why it is not likely that being monitored could affect the performance of participants in treatment and comparison groups in different ways (such as resulting in Hawthorne or John Henry effects).  b) The outcome is based on data collected in the context of a survey, and not associated with a particular intervention trial, or data are collected from administrative records or in the context of a retrospective (ex post) evaluation. | -Score “Yes” if either criterion a) or b) are satisfied;  Score "Probably yes" if the study is based on data collected during a trial and there is no obvious issue with the monitoring processes but authors do not mention potential risks.  -Score “Unclear” if it is not clear whether the authors use an appropriate method to prevent Hawthorne and John Henry Effects (e.g. blinding of outcomes and, or enumerators, other methods to ensure consistent monitoring across groups). Hawthorne effects may result where participants know that they are being observed and John Henry Effects may result from participant knowledge of being compared.  -Score "Probably no" if there was imbalance in the frequency of monitoring in intervention groups, which might have influenced participants' behaviours.  -Score "No" if neither criterion a) or b) are satisfied. |
| Performance justification | Open answer | Justification for coding decision  (Include a brief summary of justification for rating, mentioning your response to all sub questions, cite relevant pages). | |
| Outcome measurement bias :  Was the study free from biases in outcome measurement? | 1= Yes, 2 = Probably Yes, 3 = Probably No, 4 = No, 8 = Unclear | a) Outcome assessors are blinded or the outcome measures are not likely to be biased by their judgement.  b) For self-reported outcomes: respondents in the intervention group are not more likely to have accurate answers due to recall bias;  c) For self-reported outcomes: respondents do not have incentives to over/under report something related to their performance or actions, OR researchers put in place mechanisms to reduce the risk of reporting bias (researchers not strongly involved in the implementation of the programme and it is clear that their answers to the survey will not affect what they receive in the future) OR authors have measured the risks of bias through falsification tests or measuring the effect on placebo outcomes in cases where there was a risk of repoting bias.  d) Timing issue: the data collection period did not differ between intervention and comparison group, the baseline data are not likely to be affected by the beginning of the intervention or affects a small percentage of the study participants. | -Score “Yes” if criterion a), b), c) and d) are satisfied:  -Score "Probably yes" if there is a small risk related to any of a), b), c) or d) and there is no more information provided to to justify the absence of bias OR if there was a high risk of bias but authors have either controled it in their design or measured it with a placebo outcomes.  -Score “Unclear” if it there is a high risk related to any of a), b), c) or d) and there is no more information provided to to justify the absence of bias.  -Score "Probably no" if there are high risk related to a), b), c) or d) and it is clear that authors were not able to control for this bias.  -Score “No” if there is evidence of bias. |
| Outcome justification | Open answer | Justification for coding decision  (Include a brief summary of justification for rating, mentioning your response to all sub questions, cite relevant pages). | |
| Analysis reporting :  Was the study free from selective analysis reporting? | 1= Yes, 2 = Probably Yes, 3 = Probably No, 4 = No, 8 = Unclear | a) A pre-analysis plan or trial protocol is published and referred to or the trial was pre-registered or the outcomes were pre-registered;  b) Authors report results corresponding to the outcomes announced in the method section (there is no outcome reporting bias);  c) Authors report results of unadjusted analysis and intention to treat (ITT) estimation, alongside any adjusted and treatment-on-the-treated/complier-average-causal-effects analysis.)  d) Authors use the appropriate analysis method (use baseline data when available) and different treatment arms are differentiated in the analysis e) Authors have reported all the analysis which could help understand the results and no other bias is assessed as unclear due to the lack of an important analysis (e.g. a balance table or a subgroup analysis) | -Score "Yes" if all the criterion a), b), c), d), and e) are satisfied;  -Score "Probably yes" if all the conditions are met except a), or if all the conditions are met but there is some element missing that could have helped understand the results better (e);  -Score "Unclear" if there is not enough information to determine that there is an analysis missing;  -Score "Probably no" if any of the criterion b), c) or d) are not satisfied; -Score "No" if any of the criterion b), c) or d) are not satisfied and there is evidence that the analysis results would be different because large imbalances were not controled for, compliance was very low and ITT estimation was not reported or different treatment arms were pooled |
| Analysis reporting | Open answer | Justification for coding decision (Include a brief summary of justification for rating, mentioning your response to all sub questions, cite relevant pages). | |
| Other bias :  Is the study free from other sources of bias? | 1= Yes, 2 = Probably Yes, 3 = Probably No, 4 = No, 8 = Unclear |  |  |
| Other bias | Open answer | Justification for coding decision  (Include a brief summary of justification for rating, mentioning your response to all sub questions, cite relevant pages). | |
| Blinded observers  Were outcome assessors blinded to the intervention and control group? | 1=Yes 2=No 8=unclear 9= N/A | If there is no information, code NO. If there is information but it is ambiguous, code UNCLEAR. | |
| Blinded analysts :  Were data analysts blinded to the intervention and control observations? | 1=Yes 2=No 8=unclear 9= N/A | If there is no information, code NO. If there is information but it is ambiguous, code UNCLEAR. | |
| Method used to blind | Open answer, 9= N/A | Describe method(s) used to blind including method for placebo control. | |

| Question | Coding | Criteria and decision-rules | Decision-rules |
| --- | --- | --- | --- |
| Unique study identification | For example, PITA001 |  |  |
| Study first author | Open answer |  |  |
| Outcome | Open answer |  |  |
| Study design :  What type of study design is used? | 1= Natural experiment: randomised or as-if randomised 2= Natural experiment: regression discontinuity (RD) 3= CBA (non-randomised assignment with treatment and contemporaneous comparison group, baseline and endline data collection) – individual repeated measurement 4= CBA pseudo panel (repeated measurement for groups but different individuals) 5= Interrupted time series (with or without contemporaneous control group) 6= Panel data, but no baseline (pre-test) 7 = Comparison group with endline data only |  |  |
| 1: Mechanism of assignment :  Was the allocation or identification mechanism able to control for selection bias? | 1= Yes, 2 = Probably Yes, 3 = Probably No, 4 = No, 8 = Unclear |  |  |
| Experimental approaches (random allocation of the treatment): | Open answer | a) A random component in the sequence generation process is described (for example, referring to a random number table)**; b) and if the unit of allocation was at group level (geographical/ social/ institutional unit) and allocation was performed on all units at the start of the study; c) or if the unit of allocation was by beneficiary or group and there was some form of centralised allocation mechanism such as an on-site computer system; d) and if the unit of allocation is based on a sufficiently large sample size to equate groups on average.  **If a quasi-randomised assignment approach is used (for example, alphabetical order), you must be sure that the process truly generates groupings equivalent to random assignment, to score “Yes” on this criteria. In order to assess the validity of the quasi-randomisation process, the most important aspect is whether the assignment process might generate a correlation between participation status and other factors (for example, gender, socio-economic status) determining outcomes; you may consider covariate balance in determining this (see question 2). . | -Score “Yes” if criteria a), b), c), d) are all satisfied  -Score "Probably Yes" if all criteria are satisfied but there is no balance table to justify the random allocation -Score “Unclear” if the paper does not provide details on the randomisation process, or uses a quasi-randomisation process for which it is not clear has generated allocations equivalent to true randomisation. -Score "Probably No" if there are concerns with any of the criteria and no balance is provided. -Score “No” if the sample size is not sufficient or any failure in the allocation mechanism could affect the randomisation process. If the research has serious concerns with the validity of the randomisation process or the group equivalence completely fails, we recommend to assess the risk of bias of the study using the relevant questions for the appropriate methods of analysis (cross-sectional regressions, difference-in-difference, etc.) rather than the RCTs questions. |
| Regression discontinuity design : | Open answer | a) Allocation is made based on a pre-determined discontinuity on a continuous variable (regression discontinuity design) and blinded to participants or;  b) if not blinded, individuals reasonably cannot affect the assignment variable in response to knowledge of the participation decision rule; c) and the sample size immediately at both sides of the cut-off point is sufficiently large to equate groups on average. | -Score “Yes” if criteria a), b), c) are all satisfied -Score "Probably Yes" if there are minor differences in between both sides of the cut-off point but authors convincingly argue that the differences are unlikely to affect the outcome, OR individuals are not blinded and there are low risk of them affecting the assignment but the authors do not mention it.  -Score “Unclear” if it is unclear whether participants can affect it in response to knowledge of the allocation mechanism.  -Score "Probably No" if there are differences between individuals on both sides of the cutt-off point, and there are doubts that the differences are due to individuals altering the assignment OR the participants are blinded but there is evidence that the decisions that determined the discontinuity is basd on differences between the two groups or differences in time.  -Score “No” if the sample size is not sufficient OR there is evidence that participants altered the assignment variable prior to assignment. If the research has serious concerns with the validity of the assignment process or the group equivalence completely fails, we recommend assessing risk of bias of the study using the relevant questions for the appropriate methods of analysis (cross-sectional regressions, difference-in-difference, etc.) rather than the RDDs questions. |
| For assignment based non-randomised programme placement and self-selection (studies using a matching strategy or regression analysis, excluding IV) : | Open answer | a) Participants and non-participants are either matched based on all relevant characteristics explaining participation and outcomes, or; b) all relevant characteristics are accounted for.** c) and the data set used contains relevant variable that are measured in a relevant way (i.e. they were not collected for a different purpose initially and therefore are good proxy for some characteristics).  **Accounting for and matching on all relevant characteristics is usually only feasible when the programme allocation rule is known and there are no errors of targeting. It is unlikely that studies not based on randomisation or regression discontinuity can score “YES” on this criterion. There are different ways in which covariates can be taken into account. Differences across groups in observable characteristics can be taken into account as covariates in the framework of a regression analysis or can be assessed by testing equality of means between groups. Differences in unobservable characteristics can be taken into account through the use of instrumental variables (see also question 1.d) or proxy variables in the framework of a regression analysis, or using a fixed effects or difference-in-differences model if the only characteristics which are unobserved are time-invariant | -Score “Yes” if a) or b) and c) are satisfied -Score "Probably yes" if a) or b) are addressed for but there is some doubt related to c), OR authors combind statstical matching and difference-in-difference to cope with unobservable differences, OR they only did statistical matching and there was clear rules for selectino into the programme (no self-selection). -Score “Unclear” if · it is not clear whether all relevant characteristics (only relevant time varying characteristics in the case of panel data regressions) are controlled.  -Score "Probably no" if only a statistical matching was done and there was self-selection into the program. -Score “No” if relevant characteristics are omitted from the analysis. |
| For identification based on an instrumental variable (IV estimation) : | Open answer | -Score “Yes” if an appropriate instrumental variable is used which is exogenously generated: for example, due to a ‘natural’ experiment or random allocation.  -Score "Probably yes" if there is less evidence (no balance table showing differences between the intervention and comparison group). -Score “Unclear” if the exogeneity of the instrument is unclear (both externally as well as why the variable should not enter by itself in the outcome equation). -Score "Probably no" if there is evidence that enrollment in the programme is correlated with a variable that might also have an effect on outcome and on the instrumental variable. -Score “No” if it is clear that the instrument is not exogenous and affect the outcome through other channels than the program. | |
| 2: Group equivalence : was the method of analysis executed adequately to ensure comparability of groups throughout the study and prevent confounding? | 1= Yes, 2 = Probably Yes, 3 = Probably No, 4 = No, 8 = Unclear |  |  |
| Experimental approaches (random allocation of the treatment) : | Open answer | a) Baseline characteristics of the study and control/comparisons are reported and overall similar based on t-test or ANOVA for equality of means across groups; OR individual covariate differences, and for cluster-assignment external cluster-level factors, are controlled using multivariate analysis,** b) and the attrition rates (losses to follow up) are sufficiently low and similar in treatment and control, or the study assesses that loss to follow up units are random draws from the sample (for example, by examining correlation with determinants of outcomes, in both treatment and comparison groups);  c) and problems with cross-overs and drop outs are dealt with using intention-to-treat analysis or in the case of drop outs, by assessing whether the drop outs are random draws from the population;   **Even in the context of RCTs, when randomisation is successful and carried out over sufficiently large assignment units, it is possible that small differences between groups remain for some covariates. In these cases, study authors should use appropriate multivariate methods to correcting for these differences. | -Score "Yes, if a, b, c and d are addressed. -Score "Probably yes" ifthere are imbalances due to any of the criterion a), b), c) or d) but they are controled for in the analysis. -Score “Unclear” if insufficient details are provided on covariate differences or methods of adjustment or insufficient details are provided on cluster controls.  -Score "Probably no" if there are signs that one of the criterion a), b), c) or d) is not addressed (differential attrition, small samle leading to high likelihood of imbalances due to chance etc), and there is no balance table to confirm equivqlence of groups. -Score “No” if there is evidence of inequivalence that is not controled for. |
| Regression discontinuity design : | Open answer | a) The interval for selection of treatment and control group is reasonably small OR authors have weighted the matches on their distance to the cut-off point;  b) and the mean of the covariates of the individuals immediately at both sides of the cut-off point (selected sample of participants and non-participants) are overall not statistically different based on t-test or ANOVA for equality of means; c) Significant differences in covariates of the individuals have been controlled in multivariate analysis; and for cluster-assignment, authors control for external cluster-level factors that might confound the impact of the programme. | -Score "Yes, if criterion a), b), c) and d) are addressed. -Score "Probably yes" if b) is not addressed but c) is addressed and differences in means are not large. -Score “Unclear” if insufficient details are provided on controls; or if insufficient details are provided on cluster controls. -Score "Probably no" if b) is not addressed (absence of a difference test or balance table) and there are doubt regarding the continuity on both sides of the cut-off point (a). -Score “No” otherwise. |
| For non-randomised trials using difference-in-differences methods of analysis : | Open answer | a) The authors use a difference-in-differences (or fixed effects) multivariate estimation method; b) the authors control for a comprehensive set of individual time-varying characteristics, and for cluster-assignment, authors control for external cluster-level factors that might confound the impact of the programme**; c) and the attrition rate is sufficiently low and similar in treatment and control, or the study assesses that drop-outs are random draws from the sample (for example, by examining correlation with determinants of outcomes, in both treatment and comparison groups);   **Knowing allocation rules for the programme – or even whether the non-participants were individuals that refused to participate in the programme, as opposed to individuals that were not given the opportunity to participate in the programme – can help in the assessment of whether the covariates accounted for in the regression capture all the relevant characteristics that explain differences between treatment and comparison | -Score "Yes, if a, b, c, d (if relevant) are addressed and baseline imbalances between groups were relatively low OR the method was combined by a stastistical matching. -Score "Probably yes" if all possible variables are controlled for and the selection into the programme was done according to clear rules, but baseline imbalances between groups were very large. -Score “Unclear” if insufficient details are provided; or if insufficient details are provided on cluster controls.  -Score "Probably no" if some time-varying characteristics are not controled for and the programme was self-selected by the intervention groups. -Score “No” if any of the criterion is not addressed. |
| For statistical matching studies including propensity scores (PSM) and covariate matching** : Matching strategies are sometimes complemented with difference-in-difference regression estimation methods. This combination approach is superior since it only uses in the estimation the common support region of the sample size, reducing the likelihood of existence of time-variant unobservables differences across groups affecting outcome of interest and removing biases arising from time-invariant unobservable characteristics. | Open answer | a) Matching is either on baseline characteristics or time-invariant characteristics which cannot be affected by participation in the programme; and the variables used to match are relevant (for example, demographic and socio-economic factors) to explain both participation and the outcome (so that there can be no evident differences across groups in variables that might explain outcomes); and, for cluster-assignment, authors control for external cluster-level factors that might confound the impact of the programme b) in addition, for PSM Rosenbaum’s test suggests the results are not sensitive to the existence of hidden bias;  c) and, with the exception of Kernel matching, the means of the individual covariates are equated for treatment and comparison groups after matching;  d) different matching methods including varying sample sizes yields the same results and authors take into account the use of control observations multiple times against the same treatment in their standard error calculation. | -Score "Yes, if a, b, c, and d (if relevant) are addressed. -Score "Probably yes" if the selection into the programme was done according to clear rules, which are used for the matching but there are slight imbalances remaining after matching. -Score “Unclear” if relevant variables are not included in the matching equation, or if matching is based on characteristics collected at endline; or if insufficient details are provided on cluster controls. -Score "Probably no" if the programme was self-selected by the intervention groups or participants OR if the selection into the programme was done according to clear rules but there is no baseline data available to match the participants or groups on. -Score “No” if matching was done based on variables that are likely to be affected by the programme or any other scenario that affect a), b) c) or d). |
| For regression-based studies using cross sectional data (excluding IV) : | Open answer | a) The study controls for relevant confounders that may be correlated with both participation and explain outcomes (for example, demographic and socio-economic factors at individual and community level) using multivariate methods with appropriate proxies for unobservable covariates, and, for cluster-assignment, authors control particularly for external cluster-level factors that might confound the impact of the programme; b) and a Hausman test with an appropriate instrument suggests there is no evidence of endogeneity**; c) and none of the covariate controls can be affected by participation; d) and either, only those observations in the region of common support for participants and non-participants in terms of covariates are used, or the distributions of covariates are balanced for the entire sample population across groups;  **The Hausman test explores endogeneity in the framework of regression by comparing whether the OLS and the IV approaches yield significantly different estimations. However, it plays a different role in the different methods of analysis. While in the OLS regression framework the Hausman test mainly explores endogeneity and therefore is related with the validity of the method, in IV approaches it explores whether the author has chosen the best available strategy for addressing causal attribution (since in the absence of endogeneity OLS yields more precise estimators) and therefore is more related with analysis reporting bias. | -Score "Yes, if a, b, c and d are addressed. -Score "Probably yes" if all criterion are addressed but authors did not report the Hausman test (b). -Score “Unclear” if relevant confounders are controlled but appropriate proxy variables or statistical tests are not reported; or if insufficient details are provided on cluster controls.  -Score "Probably no" if any of the criterion other than b) is not addressed. -Score “No" if none of the criterion are addressed. |
| For instrumental variables approaches : | Open answer | a) The instrumenting equation is significant at the level of F≥10 (or if an F test is not reported, the authors report and assess whether the R-squared (goodness of fit) of the participation equation is sufficient for appropriate identification);  b) the identifying instruments are individually significant (p≤0.01); for Heckman models, the identifiers are reported and significant (p≤0.05); c) where at least two instruments are used, the authors report on an over-identifying test (p≤0.05 is required to reject the null hypothesis); and none of the covariate controls can be affected by participation and the study convincingly assesses qualitatively why the instrument only affects the outcome via participation. If the instrument is the random assignment of the treatment, the author should also assess the quality and success of the randomisation procedure in part a). d) and, for cluster-assignment, authors particularly control for external cluster-level factors that might confound the impact of the programme (for example, weather, infrastructure, community fixed effects, and so forth) through multivariate analysis. | -Score "Yes, if a, b, c, d (if relevant) are addressed. -Score "Probably yes" if one of the test required for criterion a) or b) is not reported but the other is, and the rest of the criterion are addressd and the instrument is convincing. -Score “UNCLEAR” if relevant confounders are controlled for but appropriate statistical tests are not reported; or if insufficient details are provided on cluster controls -Score "Probably no" if exogeneity of the instrument is not convincing and appropriate tests are not reported. -Score “No” otherwise if any of the tests required for criterion a), b) or c) are reported and not satisfied. |
| Performance bias : was the process of being observed free from motivation bias? | 1= Yes, 2 = Probably Yes, 3 = Probably No, 4 = No, 8 = Unclear | a) For data collected in the context of a particular intervention trial (randomised or non-randomised assignment), the authors state explicitly that the process of monitoring the intervention and outcome measurement is blinded, or argue convincingly why it is not likely that being monitored could affect the performance of participants in treatment and comparison groups in different ways (such as resulting in Hawthorne or John Henry effects). b) The study is based on data collected in the context of a survey, and not associated with a particular intervention trial, or data are collected from administrative records or in the context of a retrospective (ex post) evaluation. | -Score “Yes” if either criterion a) or b) are satisfied; -Score "Probably yes" if the study is based on survey data collected during a trial and there is no obvious issue with the monitoring processes bu authors do not mention potential risks. -Score “Unclear” if it is not clear whether the authors use an appropriate method to prevent Hawthorne and John Henry Effects (e.g. blinding of outcomes and, or enumerators, other methods to ensure consistent monitoring across groups). Hawthorne effects may result where participants know that they are being observed and John Henry Effects may result from participant knowledge of being compared. -Score "Probably no" if there was imbalance in the frequency of monitoring in intervention groups, which might have influenced participants' behaviours. - Score "No" if neither criterion a) or b) are satisfied. |
| Performance bias - Justification | Open answer | Justification for coding decision  (Include a brief summary of justification for rating, mentioning your response to all sub questions, cite relevant pages). | |
| Spill-overs, cross-overs and contamination : was the study adequately protected against spill-overs, cross-overs and contamination? | 1= Yes, 2 = Probably Yes, 3 = Probably No, 4 = No, 8 = Unclear | a) There was no implementation issues that might have led the control participants to receive the treatment (implementer's mistake). b) The intervention is unlikely to spill-over to comparisons (e.g. participants and non-participants are geographically and/or socially separated from one another and general equilibrium effects are not likely) or the potential effects of spillovers were measured (e.g. variation in the % of unit within a cluster receiving the treatment). c) There is no risk of contamination by external programs: the treatment and comparisons are isolated from other interventions which might explain changes in outcomes.  d) There is nothing in the surveys that might have given the control participants an idea of what the other group might receive OR they did but there is no risk that this has changed their behaviours; AND the survey process did not reveal information to the control group that they did not have before (e.g. the study aims to measure increase in take up of a service or product that participants might not know about) Authors might put something in place in the design of the study that allows to control for that suvey effect (e.g. a pure control with no monitoring except baseline endline) | -Score “Yes” if criterion a), b), c) and d) are satisfied; -Score "Probably yes" if there is no obvious problem but there is no information reported on potential risks related to spillovers, contamination, or survey effects in the control group OR if htere were issus with spill-overs but they were contolled for or measured. -Score “Unclear” if spill-overs, cross-overs, survey effects and/or contamination are not addressed clearly. -Score "Probably no" if any of the criterion a), b), c) or d) are not satisfied but the scale of the issue is not clear. -Score “No” if any of the criterion a), b), c) or d) are not satisfied and happened at a large scale in the study. |
| Spill-overs, cross-overs and contaminatio - Justification | Open answer | Justification for coding decision (Include a brief summary of justification for rating, mentioning your response to all sub questions, cite relevant pages). | |
| Outcome measurement bias | 1= Yes, 2 = Probably Yes, 3 = Probably No, 4 = No, 8 = Unclear | a) Outcome assessors are blinded or the outcome measures are not likely to be biased by their judgement.  b) For self-reported outcomes: respondents in the intervention group are not more likely to have accurate answers due to recall bias; c) For self-reported outcomes: respondents do not have incentives to over/under report something related to their performance or actions, OR researchers put in place mechanisms to reduce the risk of reporting bias (researchers not strongly involved in the implementation of the programme and it is clear that their answers to the survey will not affect what they receive in the future) OR authors have measured the risks of bias through falsification tests or measuring the effect on placebo outcomes in cases where there was a risk of repoting bias. d) Timing issue: the data collection period did not differ between intervention and comparison group, the baseline data are not likely to be affected by the beginning of the intervention or affects a small percentage of the study participants. | -Score “Yes” if criterion a), b), c) and d) are satisfied: -Score "Probably yes" if there is a small risk related to any of a), b), c) or d) and there is no more information provided to to justify the absence of bias OR if there was a high risk of bias but authors have either controled it in their design or measured it with a placebo outcomes. -Score “Unclear” if it there is a high risk related to any of a), b), c) or d) and there is no more information provided to to justify the absence of bias.  -Score "Probably no" if there are high risk related to a), b), c) or d) and it is clear that authors were not able to control for this bias. -Score “No” if there is evidence of bias. |
| Outcome measurement bias - Justification | Open answer | Justification for coding decision (Include a brief summary of justification for rating, mentioning your response to all sub questions, cite relevant pages). | |
| Selective analysis reporting : was the study free from selective analysis reporting? | 1= Yes, 2 = Probably Yes, 3 = Probably No, 4 = No, 8 = Unclear | a) a pre-analysis plan is published, especially for prospective NRS but it should also be for retrospective studies b) authors use ‘common’ methods of estimation (i.e. credible analysis method to deal with attribution given the data available) ; c) There is no evidence that outcomes were selectively reported (e.g. results for all relevant outcomes in the methods section are reported in the results section) ; d) Requirements for specific methods of analysis: - For PSM and covariate matching: (a) Where over 10 per cent of participants fail to be matched, sensitivity analysis is used to re-estimate results using different matching methods (Kernel Matching techniques); (b) For matching with replacement, no single observation in the control group is matched with a large number of observations in the treatment group. - For IV (including Heckman) models, (a) The authors test and report the results of a Hausman test for exogeneity (p≤0.05 is required to reject the null hypothesis of exogeneity); (b) the coefficient of the selectivity correction term (Rho) is significantly different from zero (P<0.05) (Heckman approach).  - For studies using multivariate regression analysis, authors conduct appropriate specification tests (e.g. testing robustness of results to the inclusion of additional variables, or (very rare) reporting results of multicollinearity test etc.). | -Score “Yes” if a), b), c) and d) are satisfied OR if a) is not met and it is a retrospective NRS. -Score "Probably Yes" if authors combined methods and reported relevant tests (d) only for one method OR if all the criteria are met except for a) and it is a prospective NRS -Score "Unclear" if intended outcomes not specified in the paper OR if any of the requirements for d) are not reported.  -Score "Probably No" if b) is addressed, but authors did not present results for all outcomes announced in the method section OR did not meet requirement d) although reported.  -Score “No” if authors use uncommon or less rigorous estimation methods such as failure to conduct multivariate analysis for outcomes equations OR if some important outcomes are subsequently omitted from the results or the significance and magnitude of important outcomes was not assessed. |
| Analysis reporting bias - Justification | Open answer | Justification for coding decision  (Include a brief summary of justification for rating, mentioning your response to all sub questions, cite relevant pages). | |
| Other risks of bias : Is the study free from other sources of bias? | 1= Yes, 4 = No | Score “Yes” if the reported results do not suggest any other sources of bias. Score “No” if other potential threats to validity are present, and note these here (e.g. coherence of results, survey instruments used are not reported) | |
| Other risks of bias - Justification | Open answer | Justification for coding decision  (Include a brief summary of justification for rating, mentioning your response to all sub questions, cite relevant pages). | |

Appendix 4: Characteristics of included studies

Table A4.1: Characteristics of included studies: Citizen feedback and monitoring interventions

| Papers and location | PITA mechanism | Sector | Intervention description | Intervention development | Equity considered? | Study length -months | Primary study design and analysis method | Comparison group | Outcomes |
| --- | --- | --- | --- | --- | --- | --- | --- | --- | --- |
| Alhassan et al. 2016 (Duku et al., 2018; Alhassan et al. 2015)  Ghana - Greater Accra and Western (predominantly rural) regions of Ghana | Accountability | Health | WOTRO-COHEiSION Ghana project: This intervention used existing community groups or associations to identify gaps in service delivery in healthcare facilities, using a tool known as MyCare. Focus groups were held by a facilitator identify these gaps. The issues were then communicated to all intervention health facilities by the facilitator and a community liaison person and the facilities were encouraged to initiate changes through the development of an improvement plan. A small token prize was offered to the best performing health facilities. | No explanation of how the intervention was developed. Field trial of community engagement activities, designed to improve a national policy but not implemented by the Ghanaian government. | Evaluation design: consider gender dynamics of focus groups | 24 - 48 | Cluster RCT  Statistical matching (PSM), Different-in-difference (DID) estimation methods | No PITA mechanism (service delivery as usual) | Attitudes to services  Service access / quality  Service use  Well-being  Provider Response |
| Berman et al. 2017  Afghanistan | Accountability | Local infra-structure | Integrity Watch Afghanistan (IWA)'s infrastructure monitoring program: Works with community volunteers to train them in a combination of engineering and accounting skills in order to monitor local road construction quality, as well as how to monitor financials of providers. The implementer, IWA, provides ongoing support for monitoring. In addition to training, they also establish semi-formal accountability mechanisms called Provincial Monitoring Boards, which include representatives from the Ministry of Rural Rehabilitation and Development (MRRD), Provincial Councils, IWA-trained community monitors, construction contractors, and sometimes aid agencies. At these meetings, they discuss construction quality, contractor performance, and potential misappropriation of funds. Finally, IWA encourages informal accountability through monitor-led community mobilization. | Appears locally developed through the implementing NGO, IWA. This study tests their existing programme through a field experiment. | Not explicit | Two follow ups: 24, 48 | Cluster RCT  Fixed effects regression | No PITA mechanism (service delivery as usual) | Service access / quality |
| Björkman et al. 2017 (Björkman et al. 2014; Björkman & Svensson 2009; Björkman & Svensson 2010; Björkman & Svensson 2007; Björkman et al. 2006; Donato & Garcia, 2016)  Uganda - districts from Eastern and Central, Western and Northern regions | Accountability | Health | Community Scorecard and Monitoring: Two citizen feedback interventions tested in this RCT:  - Researchers created a scorecard of health facilities based on two surveys of health facility data and user data, and presented the results through a series of meetings with (a) community members (through PRA-informed techniques), (b) service providers, and (c) a mixed interface meeting with both groups (five days). This was followed by a one-day midterm review at six months, data collection after at one year, repeat engagement at two years mark, one day review at three years mark, and final follow-up and data collection at four years mark. - In the second intervention, a similar process was followed without the initial scorecard development, presentation and dissemination. | Designed by staff from Stockholm University and the World Bank, implemented in cooperation with a number of Ugandan practitioners and community organizations. | Intervention design: Participants divided into key social groups such as women, men, youths, disabled, elderly to get perspectives over service delivery and determine preferences for change. | Two follow ups: 12, 48 | Cluster RCT  DID estimation methods, Fixed effects regression, covariate-adjusted estimation | No PITA mechanism (service delivery as usual) | Wellbeing  Service use  Provider response  Citizen engagement  Service access / quality  Other |
| Fiala & Premand, 2017  Afghanistan | Accountability | Social protection; Local infrastructure | Community monitoring training for NUSAF2: Communities selected to receive a local development project through a community-based development programme called the Second Northern Uganda Social Action Fund (NUSAF2). Three treatment arms, including the two below (the other is under performance information provision): - A randomly selected sub-set of the NUSAF2 communities received intensive, six-day training on how to monitor community projects, as well as identify and make complaints about corruption and mismanagement to implementing partners, local, sub-national or national leaders. The trainings were implemented in partnership with local civil society organizations.  - Within these communities, a randomly selected sub-set were presented researcher generated scorecards - a community facilitator, trained by the research team went to communities to present these scores. The information presented included a ranking of communities relative to other NUSAF2 communities. | Not clear - NUSAF2 was a large-scale CDD programme implemented by the Office of the Prime Minister, in coordination with local district and sub-county with funding from the World Bank and DFID. This particular RCT worked with the Inspectorate of Government, the main oversight arm of the government of Uganda. | Not explicit | Two follow ups: 19, 24 | Cluster RCT  Covariate-adjusted estimation | Other PITA mechanism | Wellbeing  Provider response  Citizen engagement  Other  State-society relations  Service access / quality |
| Grossman et al. (2017)  Uganda - Arua | Accountability | Health | U-Bridge: U-Bridge is an SMS-based service system that allows citizens and local government officials to submit, monitor and respond to requests around public service delivery. It is an open-source software that runs on mobile devices, including tablets and smartphones. Citizens can choose to register to participate in the sending and receiving of messages. The implementing team registered mobile phone numbers at community meetings, and the team undertaking the research also did door-to-door registration. | U-Bridge was designed by UNICEF Uganda and RTI International. It is not stated to what extent, if any, local stakeholders were engaged in the design process. | Not explicit | 14 [intervention] | Cluster RCT  Covariate-adjusted estimation | No PITA mechanism (service delivery as usual) | Provider response  Service access / quality  Service use  Citizen engagement |
| Gullo et al. 2017  Malawi -Ntcheu district | Accountability | Health | CARE Malawi Community Score Card (CSC) around health (6-month cycles, after which all phases repeated): - 1st phase: relevant stakeholders identify the sectoral and geographic scope of the initiative, and facilitators trained. 2nd phase: CSC is conducted with the community via focus group discussions to identify and prioritize issues they are facing in accessing services. Groups are separated by men, women, youth, etc. A measurable indicator is developed for each theme. The indicators are then verified and scored by the community, generating a Score Card. They also give suggestions for improvement. 3rd phase: The same process is conducted with service providers. 4th phase: interface meeting between community members and service providers, as well as local government officials and other power holders to share and discuss their respective Score Cards, issues and priorities. Community-wide action plan for service improvement is agreed. 5th phase: action plan implementation, monitoring. | The Community Score Card was developed by CARE Malawi in 2002. | Intervention design: Participants were divided into key social groups such as women, men, youths in order to get their perspectives over issues concerning service delivery and determine their preferences for change. | 24 | Cluster RCT  DID estimation methods | No PITA mechanism (service delivery as usual) | Attitudes to services  Service access / quality  Service use |
| Molina, 2014  Colombia - Nationwide projects | Accountability | Local infrastructure | Citizen Visible Audit (CVA) program: for funds allocated to infrastructure projects to facilitate provision of public goods for people who live in the nearby community. Selection of projects to be audited was determined by the size of royalties received, the number of previous irregularities in the management of royalties, and the expected social impact of the project. Initially, information disseminated about the programme in the relevant community via radio, newspapers, invitations and television. During the first public forum the infrastructure project was introduced to the community, citizens were told about their rights and entitlements. Periodic public forums held, bringing together local authorities, neighbors, and representatives from the implementing firm, where the project progress was explained in detail. Commitments monitored by the community, facilitators from the central government (DNP) and the project supervisor. If a commitment was not honored, facilitators and supervisors intervene to let the local government know about this. If the problem persists, administrative complaints submitted to the Supreme Audit Body. Before making the final payment to the executing firm, the finalized project is presented to the community. | N/A | Not explicit | Unclear | Comparison group with endline data only (NRS)  Statistical matching, Covariate-adjusted estimation, Comparison of means | No PITA mechanism (service delivery as usual) | Attitudes to services  Provider response  Service access / quality |
| Olken, 2007  Indonesia - Java (East Java and Central Java) | Accountability | Local infrastructure | Kecamatan Development Project (KDP) - invitation to accountability meetings + scorecards treatment arm: In the context of a CDD project in which communities apply for block grants to fund development projects, one treatment arm included invitations plus anonymous comments forms asking for villagers' opinions of project. The distribution was the same as the invitations treatment. Comments forms contained three closed-response questions (good, satisfactory, poor) about aspects of the projects and two free response questions asking about job performance of implementers and project-related issues. These were to be returned to sealed drop box before the meeting, placed either at a village school or at a store in the village. Comments forms were collected from drop boxes two days before the meeting and summarized by an enumerator, who read the summary at the meeting. | The programme that this intervention is part of is a World Bank-funded and government implemented CDD program. The intervention PITA mechanism itself seems to be created by donors or the researchers. | Not explicit | 7 | Cluster RCT  Covariate adjusted regression |  | Service access / quality  Citizen engagement  Provider response |
| Palladium, 2015  DRC - Bukavu, Kananga, and Mutavi cities | Accountability | Justice and security | Security Sector Accountability and Police Reform (SSAPR) Program: Various citizen engagement components to encourage policy accountability:  - Journalists trained with the aim of bringing security issues into public debates and better informing citizens, raising awareness on security issues and reporting on the implementation of police reform.  - Individuals trained from more than 150 CSOs to increase their knowledge of local security issues and specific tools designed to assist local community members to hold the police to account. These include scorecards, suggestion boxes and various forms of action research. - A number of community forums designed to support collaboration between police, the community and administrative officials.  - Worked with local government assemblies to link them more closely to the police reform process. Parliament members in all pilot cities participated in security control activities as well as security monitoring missions. | The design of the theory of change involved extensive consultative process between the DFID Stabilization Unit and the SSAPR design and implementation team. It was based on SSAPR’s implementation experience and the personal knowledge of the programme team. Beneficiaries not included in this process | Evaluation design: They do sub-group analysis by women and men. | 48 [intervention] | CBA - pseudo-panel (repeated measurement for groups but different individuals) (NRS)  Statistical matching, Different-in-difference (DID) estimation methods | No PITA mechanism (service delivery as usual) | Provider response  State-society relations  Attitudes to services  Well-being |

Table A4.2: Characteristics of included studies: Participatory planning interventions

| Papers and location | PITA mechanism | Sector | Intervention description | Intervention development | Equity considered? | Study length -months | Primary study design and analysis method | Comparison group | Outcomes |
| --- | --- | --- | --- | --- | --- | --- | --- | --- | --- |
| Ananthpur et al. 2014  India - Karnataka state - Gulbarga, Raichur, Bidar, Davengere and Chitradurga districts | Participation | Local government / Sub-national government - no specific sector | “People’s Campaign” Karnataka - RCT: An information campaign intervention that had three stages:  - citizenship engagement programme which lasted one week where facilitators visited each village neighborhood to encourage them to attend neighborhood meetings (ward sabhas). Citizens were informed about training that would instruct them in processes of participatory planning, and disseminated information about the budgets. They explained the purpose and value of the gram sabha, rights of citizens, and information on how to examine panchayat records such as budgets, and minutes of meetings. At the end of the week, a special meeting was held where priorities were finalized and listed in a Village Action Plan. Straight after the Village Action Plan, a meeting was held with local bureaucrats to reach an agreement on the plan implementation, bureaucrats committed to provide funding and technical support for projects over the course of the year. Over the next 2 years, the progress of the implementation was monitored roughly every month, citizen initiatives were tracked. | Intervention format itself was developed in another state Kerala - locally driven campaign by the communist (CPI-M) led government. Bureaucrats and activists from the Karnataka State Institute Development (KSIRD) designed the intervention for Karnataka - contextualized to the low literacy, high inequality, and semi-arid context of north Karnataka. | Evaluation design: in their qualitative analysis, they consider the participation of particularly marginalized groups and how women have been mobilized by the intervention. Also gender sub-group analysis | 24 | Cluster RCT   DID estimation methods; Fixed effects regression | No PITA mechanism (service delivery as usual) | Citizen engagement  Provider response  Service access / quality  State-society relations  Wellbeing |
| Beath et al. 2013  Afghanistan | Participation - Inclusion | Social protection | The programme considers the provision of wheat / food aid through the National Solidarity Programme (NSP) CDD programme in Afghanistan. The intervention that we include aims to test whether mandating (women's) participation in elected councils (traditional unelected councils) overseeing food aid distribution leads to better targeting and less leakage. We do not include the results that focused only on the impacts of the NSP programme itself. | World Bank program, including the mandated interventions | Intervention design: considers the mandating of women into elected councils | 48 | Cluster RCT | Other PITA mechanism | Provider response  Citizen engagement  Service access / quality |
| Bradley & Igras, 2005  Guinea / Kenya | Participation | Health | COPE (Client-Oriented, Provider-Efficient services): intervention implemented COPE in health facilities: COPE is a tool to help health providers identify service delivery issues, and develop plans to deal with them. Includes client exit interview too, which encourages staff to talk with and listen to their clients about the quality of the services offered. At the beginning, external facilitators oriented district supervisors and intervention site managers to the method in a one-day workshop. After the orientation, COPE was introduced in four sites in each country. District supervisors were encouraged to attend (and mostly they did attend), the first and subsequent COPE exercises, to help site staff address some of the more difficult issues. As per the usual COPE process, external resources were used to conduct short, on-site training in all intervention sites on topics identified as priorities by facility staff. | Seems not to be locally driven: COPE was pioneered by Engender Health in the early 1990s and has been adapted by a variety of agencies since. | Not explicit | 15 | CBA (non-randomized study (NRS) with comparison group with pre-test and post-test) - individual repeated measurement  Statistical matching; Comparison of means | No PITA mechanism (service delivery as usual) | Service access / quality  Service use  Attitudes to services  Provider response |
| Beuermann & Amelina, 2014  Russia - Adygea, Penza, and Perm. | Participation | Local government / Sub-national government - no specific sector | Participatory Budgeting Training Russia - RCT: In 2006, all rural settlements in Russia were required by law to hold public hearings before approving newly legislated formal settlement budgets - participatory budgeting. This trial supported this process, tested through 2 treatment groups: - The first group provided six training sessions covering the whole cycle of participatory budgeting (training treatment).  - The second provided the same training sessions plus two full-time consultants for 1 year to each settlement. The consultants were local residents trained in the fundamentals of fiscal planning, participatory budgeting and in the creation of the necessary local legal documents. They ensured the realization of six community meetings for budgetary priorities. | Not clear - this is an RCT that introduces training to implement a government law - so the RCT interventions probably not driven at the local settlement level but funded by World Bank + government of Russia so potentially nationally driven. | Not explicit | 22 | Cluster RCT  Fixed effects regression | Weaker PITA mechanism (same PITA type) | Provider response  Citizen engagement  State-society relations  Attitudes to services |
| Diaz-Cayeros et al., 2014  Mexico - Oaxaca | Participation - inclusion | Local government / Sub-national government - no specific sector | Usos y costumbres, Oaxaca: A 1995 constitutional government amendment in Oaxaca gave municipalities the choice to change to a traditional system of local governance (indigenous areas). It is a varying set of rules and practices to select leaders and solve collective-choice dilemmas at a local level - varies by municipality. Based on community assemblies where discussion over public priorities shapes the decision about budget spending. Engaging most of the community in the process of deliberation. Once collective decisions are taken according to these informal rules, they are taken to the formal municipal government institutions. Other differences between the usos and other system include the way in which political leaders are chosen, how collective decisions are made, the way in which tax rates and bases are decided upon and employment in public services and how monitoring and sanctions are decided for public employees. | Constitutional change was a formalization of informal, traditional means of local government that already existed in most indigenous areas. The authors hint at one of the drivers of the law change - however, there is no information presented on why the change in constitutional government amendment came about. | Intervention design: formalizes participation of traditional forms of governance, specifically indigenous groups in municipality level government decision-making.  Evaluation design: measures women participation | 120 / 240 | CBA (non-randomized study (NRS) with comparison group with pre-test and post-test) - individual repeated measurement  Statistical matching; DID estimation methods | Weaker PITA mechanism (same PITA type) | Service access / quality |
| Giné et al. 2018  Pakistan - Rural districts of Nowshera, Mianwali, Bahawalpur, Hyderabad and Tando Muhammed Khan | Participation - inclusion | Health | Social Mobilization for Empowerment (MORE) program: The MORE programme is a CDD program, however, in the first 3 years of the program, treatment villages were only provided support for social mobilization. They assess the impact of this intervention before the resource inputs came. Representatives from the NGO helped organize villagers into grass-roots organizations of 15 to 20 members called Community Organizations (COs), which aimed to provide a platform for collective efforts and allow members to pool resources for common development goals. A social mobilization team (SMT) approached a few people in the village to help organize a meeting of the community with the social organizer (SO). COs hold regular meetings where members can discuss local issues, prioritize community needs, and resolve any conflicts at the local level. | CDD programme with local NGO designed community mobilization intervention | Intervention design: the inclusion of women and poor households in the mobilization and CO formation process was actively encouraged | 36 | Cluster RCT   Fixed effects regression | Pipeline (wait-list) control (still service delivery as usual) | Service access / quality  Wellbeing  Attitudes to services  Service use |
| Goncalves, 2013  Brazil - Nationwide | Participation - inclusion | Infra-structure - roads electricity telecom water and sanitation | Participatory Budgeting Brazil - city council is in charge of organization and promotion of meetings, also providing technical information to participants. Participatory process formally begins with a set of parallel neighborhood assemblies, open to all residents. An update of the previous years’ approved works is given, local needs are discussed, desired investments are listed, and neighborhood representatives are elected by the attendants. Elected delegates take part in municipality-wide coordinating meetings, to draw up a final draft for the different area investment priorities, which is then passed to the executive and the participatory council. Budget is defined by them using the popular priority ranking together with a set of weights (such as the share of population affected by the project, the index of local poverty and measure of need/shortage of the good demanded for example) which are designed to promote equity in the distribution of resources as well as to take account of the project technical and financial feasibility. | Locally developed - PB emerged from direct negotiations between government officials and civil society leaders, as they sought to produce practical solutions to pressing needs. Community participation promoted by the elected mayors of the Worker's Party of Brazil. | Intervention design: some participatory budgeting areas adopt a “quality of life index,” which allocates greater resources on a per capita basis to poorer neighborhoods. | 168 | CBA (non-randomized study (NRS) with comparison group with pre-test and post-test) - individual repeated measurement  Statistical matching; Fixed effects regression | No PITA mechanism (service delivery as usual) | Provider response  Wellbeing |
| Touchton & Wampler, 2014  Brazil - Nationwide (municipalities with 100000+ people) | Participation - inclusion | Local government / Sub-national government - no specific sector | Participatory Budgeting Brazil - city council is in charge of organization and promotion of meetings, also providing technical information to participants. Participatory process formally begins with a set of parallel neighborhood assemblies, open to all residents. An update of the previous years’ approved works is given, local needs are discussed, desired investments are listed, and neighborhood representatives are elected by the attendants. Elected delegates take part in municipality-wide coordinating meetings, to draw up a final draft for the different area investment priorities, which is then passed to the executive and the participatory council. Budget is defined by them using the popular priority ranking together with a set of weights (such as the share of population affected by the project, the index of local poverty and measure of need/shortage of the good demanded for example) which are designed to promote equity in the distribution of resources as well as to take account of the project technical and financial feasibility. | Locally developed - PB emerged from direct negotiations between government officials and civil society leaders, as they sought to produce practical solutions to pressing needs. Community participation promoted by the elected mayors of the Worker's Party of Brazil. | Intervention design: some participatory budgeting areas adopt a “quality of life index,” which allocates greater resources on a per capita basis to poorer neighborhoods. | 240 | CBA (non-randomized study (NRS) with comparison group with pre-test and post-test) - individual repeated measurement  Statistical matching | No PITA mechanism (service delivery as usual) | Provider response  State-society relations  Wellbeing |

Table A4.3 Characteristics of included studies: Community-based natural resource management committees

| Papers and location | PITA mechanism | Sector | Intervention description | Intervention development | Equity considered? | Study length -months | Primary study design and analysis method | Comparison group | Outcomes |
| --- | --- | --- | --- | --- | --- | --- | --- | --- | --- |
| Bandyopadhyay et al. 2004  Namibia - Kunene and Caprivi regions | Participation | Natural resource management | Policy on Wildlife Management, Utilization and Tourism in Communal Lands: In 1995, the post-independent government laid out a set of access rules for communal lands. It created communal conservancies or areas where communities could economically exploit and gain from wildlife resource management. They give communities rights over wildlife resources if they are able to identify conservancy boundaries, have a well-defined membership, choose a representative committee to implement programs and develop an acceptable constitution. The local villagers benefit by being able to negotiate contracts with tourism agencies, manage guards and game-hunting activities, and make decisions about revenue sources and uses. | The intervention is a consequence of a policy enacted by the post-independent government in 1995, the "Policy on Wildlife Management, Utilization and Tourism in Communal Lands" | Not explicit | 12 - 48 [intervention] | Comparison group with endline data only (NRS)  IV-regression; Covariate-adjusted estimation; Comparison of means | Weaker PITA mechanism (same PITA type) | Wellbeing |
| Bandyopadhyay et al. 2010  Philippines - Magat River Integrated Irrigation System (MRIIS) in Region-2, Luzon | Participation | Natural resource management | Irrigation Management Transfer (IMT) Philippines: The intervention involves Irrigation Management Transfer (IMT) contracts for Irrigation Associations (IAs) - transfer of more management responsibility to local farmer organizations. An IMT contract requires prior infrastructural improvements such as canal lining, modified pipes, improved gates and so on. A greater role for IAs is expected to increase the responsiveness of water management to suit real-time needs. IMT IAs also collect irrigation service fees (ISF) from their members and remit these to NIA (the national irrigation organization), which is expected to return 50% of the fees.  Compared to IAs without the IMT contracts (less power devolved to them than the IMT IAs) | Combination of local driver and donor funding - interest from international organizations, local forces toward decentralization and a natural evolution within the irrigation sector. Launched under a World Bank funded program. | Evaluation design: sub-group analysis by asset rich or asset poor | 48 | Comparison group with endline data only (NRS)  Statistical matching; IV-regression | Weaker PITA mechanism (same PITA type) | Provider response  Service access / quality  Citizen engagement  Wellbeing |
| Barde, 2017  Brazil | Participation | Natural resource management | Water User Associations Brazil - small-scale water supply systems implemented and operated by water user associations |  | Not explicit | 120 | CBA (non-randomized study (NRS) with comparison group with pre-test and post-test   Statistical matching; DID estimation methods | No PITA mechanism (service delivery as usual) | Service access / quality |
| Huang, 2014  China - Ningxia and Henan provinces from the Yellow River Basin (YRB) and Hebei province in the Hai River Basin | Participation | Natural resource management | Water User Associations (WUAs) China: WUA is a farmer-based, participatory organization in which farmers organize themselves to elect a board as their representative to manage the village’s irrigation system. WUAs should be set up in a context-specific manner and contingent on the local history of water management and specific problems needed to be resolved - so some heterogeneity in how they operate. | Unclear whether locally driven or donor created - the first WUA was established in south China in 1995 with the assistance of the World Bank - not clear who drove the policy forward. Policy documents starting from 2002 made clear the government’s intention to extend WUAs nationwide. | Not explicit | 72 | CBA (non-randomized study (NRS) with comparison group with pre-test and post-test) - individual repeated measurement  IV-regression; Fixed effects regression | Other PITA mechanism / no PITA mechanism (service delivery as usual) | Provider response  Service access / quality  Citizen engagement  Wellbeing  Service use |
| Persha & Meshack, 2016  Tanzania -Kilimanjaro, Tanga, Pwani, Lindi, Morogoro, Iringa, and Mbeya regions | Participation | Natural resource management | Joint Forestry Management: Under JFM, the government and a village institution jointly manage a government forest reserve via a formal co-management agreement and decentralized process. JFM creates forest management institutions in villages, with a formal decision-making role for villagers around the conservation and management of government forest reserves. It specifies a set of management activities that communities should engage in as part of JFM, implements a legal agreement on the allocation of rights and responsibilities around forest use and management on the part of the government and community signatories, and provides for revenue-sharing from forest management activities between government and communities." (p.3) | PFM was first introduced in 1998 and strengthened through the government’s 2001 National Forest Program, but the authors do not state to what extent the policies were developed locally or with influence of donors. The programme is implemented from the top down. | Evaluation design: assess the impact of the evaluation on women headed households | 144 for forest outcomes | CBA - pseudo-panel (repeated measurement for groups but different individuals) (NRS)  Statistical matching; DID estimation methods | No PITA mechanism (service delivery as usual) | State-society relations  Wellbeing  Service access / quality |
| Rasamoelina et al. 2015  Madagascar | Participation | Natural resource management | Gestion Contractualisée des Forêts (GCF), CFM component: A law to pass responisibility over natural resources over to community groups. A local natural resources management group (in Malagasy Vondron’Olona Ifotony (VOI), or Grassroots Community) is created. The VOI operates according to a set of rules. Once created, the VOI can request the transfer of management of a given resource from its legal owner, be it the State or the local authority. The contract is signed by three parties: (i) the VOI; (ii) the owner of the resources, be it the State or the Municipality (in the case of forests, typically the forest administration); and (iii) the Municipality (Commune), which is the most decentralized institution with elected leaders. The typical forest contract is often established with support from NGOs and requires the expertise of an environmental mediator. | The policy is presented as having been developed by the Madagascar government, but it is not clear the extent to which the World Bank may or may not have exerted influence over the policy development. | Not explicit | N/A | Comparison group with endline data only (NRS)  Statistical matching | No PITA mechanism (service delivery as usual) | State society relations  Wellbeing |
| Tachibana & Adhikari, 2009 (Tachibana & Adhikari, 2005)  Nepal - Middle Hills region | Participation | Natural resource management | Community Forestry Management Nepal: Co-management indicates management by user groups which are officially approved and registered at local forest offices. By complying with the management criteria set by the forest offices, the registered user groups receive various support from the government. 02). Upon satisfying several prerequisites, the district forest offices (DFOs) have provided legal status to well-functioning user groups by registering them. The DFOs must provide various supports, notably technical advice, to the registered user. Groups need to:  - establish an election system for the committee responsible for forest management.  - prepare forest management plans | Mix - traditional approaches to CFM emerged spontaneously out of government failures to forest management. Also supported by international donors - several specific projects mentioned including Australian donors. Government began to support at the end of the 1980s. | Not explicit | 216 months for forest condition, approximately 84 months for other outcomes | CBA (non-randomized study (NRS) with comparison group with pre-test and post-test) - individual repeated measurement  IV-regression; Covariate-adjusted estimation | No PITA mechanism (service delivery as usual) | State-society relations |

Table A4.4 Characteristics of included studies: performance information

| Papers and location | PITA mechanism | Sector | Intervention description | Intervention development | Equity considered? | Study length -months | Primary study design and analysis method | Comparison group | Outcomes |
| --- | --- | --- | --- | --- | --- | --- | --- | --- | --- |
| Banerjee et al., 2014 (Benerjee et al. 2012)  India - Rajasthan | Transparency | Justice and security | RCT - Community monitoring in police stations: Two volunteers from the local community were assigned to spend around three hours in the police station during peak operating hours. Their role was to watch the activities within the police station and become familiar with the duties, procedures and challenges faced by the police. The goals were to give a group of citizens firsthand experience with the police in a positive setting and encourage them to share their experience with others, and to provide community oversight in the police station. In addition, within each police station, staff members were randomly selected for a training intervention. They were trained in either / or soft skills such as communication, mediation, stress management, motivation, team building, leadership, attitudinal, change or professional/Investigation skills. | Developed collaboratively by researchers and the implementing agency, the Rajasthan police. The community monitoring intervention was developed in response to the Police Reform Commission report recommendations on community policing. | Not explicit | 18 | Cluster RCT (quasi-RCT)  Covariate-adjusted estimation | No PITA mechanism (service delivery as usual) | Attitudes to services  Wellbeing  State-society relations |
| Capuno & Garcia, 2010 (Capuno & Garcia, 2009)  Philippines - 12 local government units (LGUs) in Bulacan and Davao del Norte Provinces | Transparency | Local government / Sub-national government - no specific sector | Good Governance and Local Development (GGLD) project: The project developed and disseminated a set of indicators about good local governance on the responsiveness of local officials as assessed by their constituents and on citizens' civic participation and trust in local officials. In treatment areas, the intervention activities included the generation of governance index (GI) scores and their public dissemination. The GI assesses Local Government Units from zero to 100 (highest) from household surveys and official audited financial reports and meeting minutes of local consultative bodies, along three performance domains. In each province, local partners in each site were two Local Government Units (local planning and development office) and two CSOs. GI materials were distributed in transport terminals, municipal halls and marketplaces. Some also sent materials to households. | The Philippine Center for Policy Studies, an NGO, developed the intervention. In 2000, the Governance for Local Development Index (Gofordev Index or GI) was formulated, which was subsequently piloted for two years (2001-2003) in this impact evaluation. | Not explicit | 22 | CBA - pseudo-panel (repeated measurement for groups but different individuals) (NRS)  DID estimation methods; Statistical matching | Weaker PITA mechanism (same PITA type) | Attitudes to services  Provider response  State-society relations  Citizen engagement |
| Fiala & Premand, 2017  Afghanistan | Transparency | Other | Community monitoring training for NUSAF2: Communities selected to receive a local development project through a community-based development programme called the Second Northern Uganda Social Action Fund (NUSAF2). Three treatment arms, the one below is performance information provision:  - A randomly selected sub-set of the NUSAF2 communities were presented researcher generated scorecards - a community facilitator, trained by the research team went to communities to present these scores. The information presented included a ranking of communities relative to other NUSAF2 communities in their district. | Not clear - NUSAF2 was implemented by the Office of the Prime Minister, in coordination with local district and sub-county with funding from the World Bank and DFID. Community monitoring element worked with the Inspectorate of Government, the main oversight arm of the government of Uganda. | Not explicit | Scorecard only int.: 4 | Cluster RCT  Covariate-adjusted estimation | Other PITA mechanism | Provider response  Wellbeing  Citizen engagement  Service access / quality  State-society relations  Other |
| Grossman & Michelitch, 2018  Uganda - nationwide | Transparency | Local government / Sub-national government - no specific sector | ACODE Scorecard: outlined politician legally defined job duties and included scorecard dissemination to citizens. Politicians were informed and invited to meetings | It appears that the intervention was designed jointly by the research team and the local NGO | Not explicit | 24 | Cluster RCT (quasi-RCT)  Fixed effects regression | Weaker PITA mechanism (same PITA type) | Provider response  Service access / quality  Other |
| Humphreys & Weinstein, 2012  Uganda - nationwide | Transparency | Local government / Sub-national government - no specific sector | Policing Politicians: Using a scorecard, previously developed, containing "detailed information on the performance of Ugandan Members of Parliament (MPs), informed a randomly selected sample of MPs that the information would be disseminated in their constituencies, and provided voters with information about their MP’s performance through a variety of dissemination channels. While scorecards were produced and released publicly for all 319 elected MPs, the authors informed the MPs that a random sample of constituencies had been selected for robust dissemination campaigns prior to the 2011 elections. In the month before the 2011 election, a sample of constituencies also received dissemination campaigns to distribute ﬂiers with updated scorecard information. As part of the baseline survey and the endline survey, the researchers provided a random sample of voters from across all constituencies with their MP’s scorecard. | Developed by the researchers in partnership with the implementing agency | Not explicit | 48 | Prospective randomized assignment (RCT) or quasi-randomized assignment (e.g. alternation) (quasi-RCT)  Covariate-adjusted estimation | Weaker PITA mechanism (same PITA type) | Provider response  Services access / quality  Citizen engagement |
| Timmons & Garfias, 2015  Brazil - Nationwide | Transparency | Local government / Sub-national government - no specific sector | Random Public Audit - Results Publication: The Brazilian federal government undertakes random audits of sub-national expenditures (coming from federal transfers). Audits contain detailed information about the manner in which funds were spent. They identify corruption, theft, and other improper expenditure; they also identify violations in the procedural rules governing expenditure and record-keeping. The results of the audits are then posted on the internet and distributed to journalists. | No mention of donor support - seems to be locally driven. | Not explicit | 48 | Natural experiment: randomized or 'as-if' randomized assignment  Fixed effects regression | No PITA mechanism (service delivery as usual) | State-society relations  Other |

Table A4.5 Table of characteristics of included studies: rights information

| Papers and location | PITA mechanism | Sector | Intervention description | Intervention development | Equity considered? | Study length -months | Primary study design and analysis method | Comparison group | Outcomes |
| --- | --- | --- | --- | --- | --- | --- | --- | --- | --- |
| Banerjee et al., 2018 (Banerjee et al. 2016; Banerjee et al. 2015a; Banerjee et al. 2015b)  Indonesia - Six districts in provinces of Lampung, South Sumatra and Central Java | Transparency | Social protection | Raskin programme (Rice for the Poor): Food subsidy giving poor households 15kg of rice (half a typical monthly rice consumption) at co-pay price one-fifth of market price. The evaluated intervention is information is given in three treatments: 1) Entitlements information (amount). The government prints the quantity of the entitlement per household (15Kg): 1a) In half of the villages, all eligible households received cards. 1b) In the other half, only those in the lowest decile of predicted per capita household consumption received it.  2) Entitlements information (amount and price). The government printed the copay price on the card. 3) Entitlements information + public information with beneficiary lists. A community facilitator hung up posters announcing cards and beneficiary lists and also played prerecorded announcement about the cards in the local language over the village mosque loudspeaker. | Mixture of government and researchers | Intervention design: Raskin is targeted at poor households | 18 | Cluster RCT  Fixed effects regression | Weaker PITA mechanism (same PITA type) | Citizen engagement  Service access / quality  Service use  Attitudes to services |
| Kasim, 2016  Pakistan - Province of Khyber Pakhtunkhwa (KPK) | Transparency | Local government / Sub-national government - no specific sector | Information Campaign on RTI Law - Khyber Pakhtunkhwa (KPK) Pakistan: Multiple (7) treatment groups testing variations of an intervention that delivered messaging campaign on three recent reforms implemented by the provincial government: (i) the Right to Information (RTI) Act; (ii) the Right to Services (RTS) Act; and (iii) an e-Grievance Redressal System established as part of the Peshawar High Court. Messages on each act were disseminated via robot-calls to randomly selected cellular phone users in the province, which was followed by an SMS message. The messaging campaign for the e-Grievance System followed a procedure, but at the end of the call, the recipient was asked about violation of their rights and interest in using the new service. | It seems researcher driven, although messages were designed in association with the support of the provincial government in Khyber Pakhtunkhwa (KPK). | Not explicit | 6 | Cluster RCT  DID estimation methods; Covariate-adjusted estimation | Weaker PITA mechanism (same PITA type) | Wellbeing  State-society relations  Attitudes to services |
| Olken, 2007   Indonesia - Java (East Java and Central Java) | Transparency | Infrastructure - roads, electricity, telecom | Kecamatan Development Project (KDP) - RCT of invitations to participate in accountability meetings. In the context of a CDD project in which communities apply for block grants to fund development projects (mainly road surfacing to ensure year-round use), the specific treatment arm for this intervention area is the following: invitations to participate in village accountability meetings. Either 300 or 500 invitations were distributed throughout the village several days prior to each of the three accountability meetings. Invitations were distributed either by sending them home with school children or asking heads of hamlets and neighborhood associations to distribute throughout their areas of the village. | The programme that this intervention is part of is a World Bank-funded and government implemented CDD program. The PITA intervention itself seems to be created by donors or the researchers. |  | 7 | Cluster RCT  Fixed effects regression | Weaker PITA mechanism (same PITA type) | Citizen engagement  Provider response  Service access / quality |
| Pandey et al. 2007  India - Uttar Pradesh, intervention focused on 21 central, central-eastern and southern districts (out of 70 districts in total in the state). | Transparency | Health | Rights information campaign: The information campaign was conducted in two rounds in each village cluster, separated by a period of 2 weeks. Each round consisted of 2-3 meetings (4-6 meetings in total) as well as distribution of posters and leaflets. Residents were informed in advance about the dates and locations of meetings, and separate meetings were held in low and mid to high caste neighborhoods. Each meeting lasted an hour and consisted of a 15-minute audiotaped presentation that was played twice, opportunities to ask questions, and distribution of leaflets. Research assistants read a scripted introduction and were allowed to answer questions only to which the answers were already written on the leaflets. Health services information included information about the services available and where to complain about quality of quantity of health services. | Local NGO Sahbagi Shikshan Kendra and researcher team | Evaluation design: sub-group analysis by lower and mid-high caste. | 12 | Cluster RCT (quasi-RCT)  DID estimation methods | No PITA mechanism (service delivery as usual) | Service access / quality  Provider response |
| Ravallion et al. 2013  India - Bihar | Transparency | Social protection | NREGA (National Rural Employment Guarantee Act) information campaign: NREGA is a public works scheme promising 100 days’ work per year to rural households. The information campaign consists of a video providing information on NREGA services and entitlements. The 25-minute video involved professional actors performing in an entertaining and emotionally engaging story-based plot. The main story line centered on a temporary migrant worker returning to his village from the city to see his wife and daughter, learning there is NREGA work even in the lean season so he can stay with family. The film was shown in common areas such as open ground, school building or community hall. The showings were followed by question and answer sessions and distribution of one-page flyers that pictorially illustrated the main entitlements and processes under the scheme. Efforts were made by facilitators to announce and advertise the upcoming screenings in advance. Local officials including community leaders were invited to attend. | Developed following an in-depth needs assessment, which suggested that knowledge was low on NREGA entitlements and that an information campaign would need to engage viewers emotionally, should influence public knowledge not just that of participants, and should be relatively easy to scale up if proved effective in a trial. | Evaluation design: sub-group analysis for men and women. | 12 | Cluster RCT  DID estimation methods; Comparison of means | No PITA mechanism (service delivery as usual) | Citizen engagement  Attitudes to services  Service use  Service access / quality  Other  Provider response  Wellbeing |

Appendix 5: Results of critical appraisal

Risk of bias assessment for RCTs: is the study free from these sources of bias

| Study and Outcome | Random assignment mechanism | Confounding | Selection bias | Deviations from intended interventions | Performance bias | Outcome measure-ment bias | Analysis reporting bias |
| --- | --- | --- | --- | --- | --- | --- | --- |
| Alhassan et al. (2016)  1-Staff experiences with clients | Yes | Probably No | Probably No | Probably Yes | Yes | Probably Yes | No |
| Alhassan et al. (2016)  2-Staff motivation levels | Yes | Probably No | Probably No | Yes | Yes | Probably No | No |
| Alhassan et al. (2015)  3-Patient safety & risk status | Yes | Probably Yes | Yes | Probably Yes | Yes | Probably Yes | No |
| Ananthpur et al. (2014)  1-Information availability & participation | Probably Yes | Probably No | Probably Yes | Probably Yes | Yes | Probably No | Probably No |
| Ananthpur et al. (2014)  2-Public goods | Probably Yes | Probably Yes | Probably Yes | Probably Yes | Yes | Probably Yes | Probably No |
| Banerjee et al. (2014)  1-Police behavior (Decoy survey outcomes) | Yes | Probably Yes | Yes | Probably Yes | Yes | Yes | Probably Yes |
| Banerjee et al. (2014)  2-Crime victim satisfaction (Victimization survey) | Yes | Probably Yes | Unclear | Probably Yes | Yes | Yes | Probably Yes |
| Banerjee et al. (2014)  3-Public perception of police (household survey) | Yes | Probably Yes | Unclear | Yes | Probably Yes | Yes | Probably Yes |
| Banerjee et al. (2018)  1-Card receipt, belief about their eligibility & card use | Yes | Yes | Yes | Probably Yes | Probably Yes | Yes | Yes |
| Banerjee et al. (2018)  2-Quantity purchased & price paid | Yes | Yes | Yes | Probably Yes | Yes | Yes | Yes |
| Beath et al. (2013)  1-Targeting | Probably Yes | Unclear | Yes | Probably Yes | Yes | Probably No | Yes |
| Beath et al. (2013)  2-Corruption & nepotism | Probably Yes | Unclear | Yes | Probably Yes | Yes | Yes | Yes |
| Beath et al. (2013)  3-Participation | Probably Yes | Unclear | Yes | Probably Yes | Yes | Yes | Yes |
| Berman et al. (2017)  Road quality (technical assessment) | Probably Yes | Yes | Unclear | Probably No | Yes | Probably No | Probably Yes |
| Beuerman & Maria (2014)  1-Participation, alignment of priorities & satisfaction (mostly household data) | Probably Yes | Yes | Probably Yes | Yes | Yes | Yes | Probably Yes |
| Beuerman & Maria (2014)  2-Tax collection & budget allocation (administrative data) | Probably Yes | Yes | Yes | Yes | Yes | Yes | Probably Yes |
| Björkman & Svensson (2009)  1-First stage outcome: community involvement in the monitoring | Yes | Yes | Probably Yes | Yes | Yes | Yes | Probably Yes |
| Björkman & Svensson (2009)  2-Impact on practices & management, Utilization & coverage | Yes | Probably Yes | Probably Yes | Yes | Yes | Probably No | Probably Yes |
| Björkman & Svensson (2009)  3- Immunization & other health outcomes | Yes | Probably No | Probably Yes | Yes | Yes | Probably Yes | Probably Yes |
| Björkman et al. (2017)  1-Health outcomes | Yes | Probably Yes | Probably Yes | Probably Yes | Yes | Yes | Probably Yes |
| Björkman et al. (2017)  2-Utilization & coverage | Yes | Yes | Yes | Probably Yes | Yes | Probably No | Probably Yes |
| Björkman et al. (2017)  3-processes&health treatment practices | Yes | Yes | Yes | Probably Yes | Yes | Yes | Probably Yes |
| Björkman et al. (2017)  4-Health treatment practices from household survey | Yes | Probably Yes | Probably Yes | Probably Yes | Yes | Probably Yes | Probably Yes |
| Björkman et al. (2017)  5-Alternative mechanisms | Yes | Yes | Yes | Probably Yes | Yes | Yes | Probably Yes |
| Fiala & Premand (2017)  All outcomes | Yes | Probably Yes | Probably Yes | Yes | Probably Yes | Probably Yes | Yes |
| Giné et al. (2018)  1- Illness incidence, immunization, diarrhea & nutrition WASH outcomes | Probably Yes | Probably Yes | Unclear | Probably Yes | Yes | Yes | Probably Yes |
| Giné et al. (2018)  2- Utilization of basic health unit (BHU) | Probably Yes | Probably Yes | Probably Yes | No | Yes | No | Probably Yes |
| Giné et al. (2018)  3- Pregnancy & Lady Health Worker performance & satisfaction | Probably Yes | Probably Yes | Yes | Probably Yes | Yes | Probably No | Probably Yes |
| Gullo et al. (2017)  1-Maternal health service utilization | Yes | Probably Yes | Probably No | Unclear | Yes | Yes | Probably Ye |
| Gullo et al. (2017)  2-Perceived quality of services when last received | Yes | Probably Yes | Probably No | Unclear | Yes | Yes | Probably Yes |
| Gullo et al. (2017)  3- Supportive care | Yes | Probably Yes | Probably No | Unclear | Yes | Probably Yes | Probably Yes |
| Kasim (2016)  2-Phone survey outcomes | Unclear | Probably Yes | Unclear | Yes | Yes | Probably Yes | No |
| Kasim (2016)  1-In person survey outcomes | Unclear | Probably No | Unclear | Yes | Yes | Probably No | No |
| Olken (2007)  1-Corruption | Yes | Probably Yes | Probably Yes | Yes | Yes | Probably Yes | Probably Yes |
| Olken (2007)  2-Nepotism | Yes | Probably Yes | Yes | Yes | Yes | Yes | Probably Yes |
| Olken (2007)  3-Participation | Yes | Yes | Yes | Yes | Yes | Yes | Probably Yes |
| Olken (2007)  4-Impact on meetings | Yes | Yes | Yes | Yes | Probably Yes | Yes | Probably Yes |
| Pandey et al. (2007)  1- Outcomes which could be compared before & after | Yes | Probably Yes | Yes | Yes | Yes | Probably Yes | Yes |
| Pandey et al. (2007)  2- Outcomes which could not be compared before & after | Yes | Probably No | Yes | Yes | Yes | Probably Yes | Yes |

Risk of bias assessment for non-randomised studies: is the study free from these sources of bias

| Study and outcome | Group equivalence (confounding) | Selection bias | Deviations from intended interventions | Performance bias | Outcome measure-ment bias | Analysis reporting bias | Other bias |
| --- | --- | --- | --- | --- | --- | --- | --- |
| Bandyopadhyay, 2004  Houshold income | No | Probably no | Yes | Yes | Unclear | Probably no | Yes |
| Bandyopadhyay, 2004  Household expenditude | No | Probably no | Yes | Yes | Probably yes | Probably no | Yes |
| Bandyopadhyay, 2010  Irrigation level indicators | No | Unclear | Unclear | Yes | Unclear | Probably yes | Yes |
| Bandyopadhyay, 2010  Farmers yields | No | Unclear | Unclear | Yes | Yes | Probably yes | Yes |
| Barde, 2017  Access to piped water | Probably yes | Yes | Probably Yes | Yes | Yes | Probably Yes | Yes |
| Bradley, 2005  All outcomes | Probably No | Unclear | Unclear | Yes | Probably no | No | Yes |
| Capuno, 2010  Membership in local organization and participation in local projects | No | No | Yes | Yes | Probably no | Yes | Yes |
| Capuno, 2010  Desired change in service delivery, responsiveness of leaders | No | Unclear | Unclear | Yes | Yes |  | Yes |
| Diaz-Cayeros, 2014  All outcomes | Probably yes | Probably yes | Unclear | Yes | Yes | Unclear | Yes |
| Goncalves, 2013  Expenditude share in different sectors | Probably No | Probably yes | Probably yes | Yes | Yes | Unclear | Yes |
| Huang, 2014  All outcomes | Probably No | Unclear | Probably Yes | Yes | Yes | Unclear | Yes |
| Molina, 2014  All outcomes | No | No | Unclear | Yes | Yes | No | No |
| Palladium, 2015  All outcomes | Unclear | unclear | Yes | Yes | Unclear | No | Yes |
| Persha, 2016  Forest conditions | Yes | Probably yes | Unclear | Yes | Unclear | Probably no | Yes |
| Persha, 2016  Governance and livelihood | Probably yes | Probably yes | Probably yes | Yes | Unclear |  | Yes |
| Rasamoelina, 2015  Deforestation | Unclear | Unclear | Unclear | Yes | Yes | No | Yes |
| Rasamoelina, 2015  Household consumption expenditures | No | No | Probably yes | Yes | Yes | No | Yes |
| Tachibana, 2009  All outcomes | Unclear | Unclear | Unclear | Yes | Yes | Yes | Yes |
| Timmons, 2015  Log tax collection | Unclear | Unclear | Unclear | Yes | Yes | Probably Yes | Yes |
| Touchton, 2015  Health care and sanitation spending, infant mortality | Probably No | Unclear | Probably yes | Yes | Yes | Unclear | Yes |
| Touchton, 2015  Number of CSO per municipality | Probably No | Probably No | Probably yes | Yes | Yes | Unclear | Yes |

Coder Disaggreement Resolution

Legend:

Codes reported for disagreement are in the following order: Second author / Main author.

Codes in bold are the codes on which the two authors agreed after discussion.

Process:

After discussing disagreement, if the difference in coding were related to a difference in understanding of the decision criteria, changes were made for all studies to harmonize the coding. For example, the second author disagreed on an outcome being free from deviations from the intended intervention if there is no mention of the issue or geographical distance between study units, even when there does not seem to be an obvious risk of spillovers or contamination. The discussion led us to code the outcome as “Probably Yes” in these cases and adjusted all studies to make it match this reasoning.

| Study and outcome | 1)  Assign-  ment mecha-  nism | 2)  Correct unit of Analysis | 3) Confoun-  ding | 4) Selec-  tion bias | 5) Deviations from intended interven-  tions | 6)  Perfor-  mance bias | 7) Outcome measure-ment bias | 8) Analysis reporting | 9) Blinded obs-  ervers | 10) Blinded analysts | Total | Agreed | Dis-  agreed |
| --- | --- | --- | --- | --- | --- | --- | --- | --- | --- | --- | --- | --- | --- |
| Alhassan et al., (2016)  1-Staff experiences with clients | Agreed | Agreed | Agreed | Agreed | Probably Yes | Probably Yes/ Yes | Some concern/ Probably Yes | Agreed | Probably Yes/ Some concern | Probably Yes/ Some concern | 10 | 6 | 4 |
| Alhassan et al., (2016)  2-Staff motivation levels | Agreed | Agreed | Some concern/ Probably No | Agreed | Probably Yes/ Yes | Probably Yes/ Yes | Some concern/ Probably No | Agreed | Probably Yes/ Some concern | Probably Yes/ Some concern | 10 | 4 | 6 |
| Alhassan et al., (2015)  3-Patient safety & risk status | Agreed | Agreed | Agreed | Agreed | Probably No/ Probably Yes | Probably Yes/ Yes | Agreed | Agreed | Probably Yes/ Some concern | Probably Yes/ Some concern | 10 | 6 | 4 |
| Duku et al., 2018  4. Health care utilization and perceived quality, and health insurance enrolment | Agreed | Agreed | Agreed | Agreed | Some concern/ Probably Yes | Yes/ Probably Yes | Yes/ Probably Yes | Agreed | Probably Yes/ Some concern | Probably Yes/ Some concern | 10 | 5 | 5 |
| Ananthpur et al. (2014)  1-Information availability & participation | Agreed | Agreed | Probably Yes/ Probably No | Agreed | Some concern/ Probably Yes | Probably Yes/ Yes | Agreed | Agreed | Agreed | Agreed | 10 | 7 | 3 |
| Ananthpur et al. (2014)  2-Public goods | Agreed | Agreed | Agreed | Agreed | Some concern/ Probably Yes | Probably Yes/ Yes | Agreed | Agreed | Agreed | Agreed | 10 | 8 | 2 |
| Kasim (2016)  1-In person survey outcomes | Probably No/ Some concern | Agreed | Some concern/ Probably No | Agreed | Probably Yes/ Yes | Agreed | Agreed | Probably No/ No | Agreed | Agreed | 10 | 6 | 4 |
| Kasim (2016)  2-Phone survey outcomes | Probably No/ Some concern | Agreed | Some concern/ Probably Yes | Agreed | Probably Yes/ Yes | Agreed | Agreed | Probably No/ No | Agreed | Agreed | 10 | 6 | 4 |
| Banerjee et al. (2018)  1-Card receipt, belief about their eligibility & card use | Probably Yes/ Yes | Agreed | Agreed | Agreed | Agreed | Agreed | Agreed | Agreed | Agreed | Agreed | 10 | 9 | 1 |
| Banerjee et al. (2018)    2-Quantity purchased & price paid | Probably Yes/ Yes | Agreed | Agreed | Agreed | Agreed | Probably Yes/ Yes | Probably Yes/ Yes | Agreed | Agreed | Agreed | 10 | 7 | 3 |
| Rasolofosen et al. (2015)  1-Deforestation | NA | Agreed | Agreed | Agreed | Some concern/ Yes | Agreed | Agreed | Agreed | NA | NA | 7 | 6 | 1 |
| Rasolofosen et al. (2015)  2- Household consumption expenditures | NA | Agreed | Agreed | Agreed | Agreed but discussion led to change | Agreed | Agreed | Agreed | NA | NA | 7 | 7 | 0 |
| Persha & Meshack (2016)  1- Forest conditions | NA | Agreed | Agreed | Agreed | Yes/Some concern | Agreed | Yes/ Some concern | Some concern/ No = Probably No | NA | NA | 7 | 4 | 3 |
| Persha & Meshack (2016)  2- Governance and livelihood | NA | Agreed | Yes/ Probably Yes | Some concern/ Probably Yes | Yes/ Probably Yes | Agreed | Yes/ Some concern | Some concern/ Probably No | NA | NA | 7 | 2 | 5 |
|  |  |  |  |  |  |  |  |  |  | Total | 128 | 83 | 45 |

Appendix 6: Additional information for meta-analysis

Table A6.1 Citizen engagement outcome variabls

| Outcome | Detailed variable | Study |
| --- | --- | --- |
| Knowledge about processes | Correctly names local leader | Ananthpur et al., 2014 |
|  | Aware of who monitored facility performance | Björkman et al., 2017 |
| Knowledge about services | Had heard of program | Ananthpur et al., 2014 |
|  | Knows about own service user rights | Banerjee et al., 2018 |
|  | Knows about facilities available | Ravallion et al., 2013 |
| Participation | Attended any meeting in past year | Ananthpur et al., 2014 |
|  | Probability of participating in local government projects | Capuno and Garcia, 2010 |
|  | Attendance of non-elite at meetings | Olken, 2007 |
|  | Women participated in meeting | Ravallion et al., 2013 |
| Active participation | Contribute to preparing maintenance plan | Bandyopadhyay et al., 2010 |
|  | User engagement - used Raskin card | Banerjee et al., 2018 |
|  | Expressed views at meeting | Björkman et al., 2009, 2017 |
|  | Intensity of community monitoring training | Fiala et al., 2017 |
|  | Village requests for services | Grossman et al., 2017 |
|  | Maintenance expenditure | Huang et al., 2007 |
|  | Number who talk at meetings | Olken, 2007 |

Note: * synthetic effect calculated across individual outcomes reported.

Table A6.2 Provider response outcome variables

| Outcome | Detailed variable | Study |
| --- | --- | --- |
| Public spending | Central transfers per capita, spending on cultural institutions, administration, housing and utilities* | Beuermann & Amelina, 2014 |
|  | Funds received at the health facility | Björkman et al., 2017 |
|  | Per capita expenditure on administration and planning, housing and urbanism* | Goncalves et al., 2013 |
|  | Health care and sanitation spending | Touchton & Wampler, 2015 |
|  | Total funds received by health center | Grossman et al., 2017 |
|  | Development projects spending | Grossman & Michelitch, 2018 |
| Provider actions | Traditional Panchayat active, health, roads, electricity, sanitation transport, water, irrigation activity* | Ananthpur et al., 2014 |
|  | Provided subsidy card | Banerjee et al., 2018 |
|  | Participation - mean effects index | Beath et al., 2013 |
|  | Facility staff works closely with the community, visible staff duty roster, health facility receives monitoring visits from heads* | Björkman et al., 2017 |
|  | Civic engagement - mayors carry out council meetings, council meetings are open to citizens* | Diaz-Cayeros et al., 2014 |
|  | Health monitoring index | Grossman et al., 2017 |
|  | Effect on project selection – agriculture, watsan, transport, health, other* | Humphreys et al., 2014 |
|  | Adoption of participatory budgeting | Timmons, 2015 |
|  | Provider action - serious response taken | Olken, 2007 |
|  | Village council meeting occurred in past six months | Pandey et al., 2007 |
| Staff motivation | Overall staff motivation score | Alhassan et al., 2016 |
|  | Facility management: my opinion is valued by management, staff morale is high, management encourages training, supervisors help solve problems, supervisors help with training, feel part of a team, feel benefit from supervision* | Bradley et al., 2005 |
| Politician performance | Current mayor of the municipality is a woman, share of the municipal council made up by women* | Diaz-Cayeros et al., 2014 |
|  | Politician performance index | Grossman & Michelitch, 2018 |
|  | MP performance according to the scorecards | Humphreys & Weinstein, 2012 |
| Perceived response by user | Perceives President is responsive to needs | Ananthpur et al., 2014 |
|  | Perceived to have benefited vulnerable households, proportion of recipients reported ex-post to be vulnerable* | Beath et al., 2013 |
|  | Citizen satisfaction with public decision making in their settlement* | Beuermann & Amelina, 2014 |
|  | Perceives barangay councilors, captain, mayor respond to the needs of the barangay and attend to complaints* | Capuno and Garcia, 2009 |
|  | Perceived challenges in procurement process index, satisfaction with supplier index, satisfaction with district vet index* | Fiala et al., 2017 |

Note: * synthetic effect calculated across individual outcomes reported.

Table A6.3 Service access outcome variables

| Outcome | Detailed variable | Study |
| --- | --- | --- |
| Physical access to services | Water sources, roads, Anganwadis, below poverty line (BPL) cards provided* | Ananthpur et al., 2014 |
|  | Use of food subsidy card in past two months and amount (kg)* | Banerjee et al., 2018 |
|  | New health units constructed and amenity renovations undertaken* | Björkman et al., 2007 |
|  | Municipal household access to water, sanitation and electricity services* | Diaz-Cayeros et al., 2014 |
|  | Lady health worker assigned to community | Giné et al., 2018 |
|  | Water parts and services provided | Grossman et al., 2017 |
|  | Development projects provided | Grossman & Mitchelich, 2018 |
|  | Land irrigated | Huang et al., 2014 |
|  | Access to water | Humphreys & Weinstein, 2012 |
|  | Access to water | Barde et al., 2017 |
|  | Development work undertaken, visited by midwife* | Pandey et al., 2007 |
|  | Forest products harvested from reserve | Persha et al., 2016 |
| Cost of service | Service access price, subsidy* | Banerjee et al., 2018 |
|  | Amount of consultation fee paid at BHU | Giné et al., 2018 |
| Measured quality of service | Health facility performance index | Alhassan et al., 2015 |
|  | Employees in Anganwadi, Engineer visits, extension visits, roads with a drain, roads free of garbage* | Ananthpur et al., 2014 |
|  | Canal maintenance | Bandyopadhyay et al., 2010 |
|  | Condition of amenities at health centers, number of beds, drugs availability, weighing scale, bicycle equipment, use of equipment* | Björkman et al., 2017 |
|  | Index of quality of LHW services received, wait time at basic health unit* | Giné et al., 2018 |
|  | Num days facility is without ORS or antimalarials, outpatient referrals, frequency of events undertaken by clinic* | Grossman et al., 2017 |
|  | Outreach campaigns, new employees | Grossman & Mitchelich, 2018 |
|  | Timely water deliveries | Huang et al., 2014 |
|  | Respondent's average assessment of the quality of six government services | Humphreys & Weinstein, 2012 |
|  | Antenatal care, postnatal care by a community health worker* | Gullo et al., 2017 |
|  | Aggregate of three measures of road quality | Berman et al., 2017 |
|  | Forest cover, forest governance index | Persha et al., 2016 |
|  | Forest cover | Rasamoelina et al. 2015 |
|  | Interpersonal skills: politeness to client (offered a seat, maintained eye contact, greeted, explained well, records maintenance, gentle mannered, confirmed client understood, session uninterrupted)* | Bradley et al., 2005 |
|  | Forest cover (lopping index of forest averaged over plots) | Tachibana & Adhikari, 2004 |
|  | Overall score of indices measuring quality of training | Fiala et al., 2017 |
| Absenteeism | Workers not physically present | Björkman et al., 2009 |
|  | Unauthorized absent, health center has attendance register book* | Grossman et al., 2017 |
|  | Absenteeism | Grossman & Mitchelich, 2018 |
|  | Doctor was present at medical clinic | Humphreys & Weinstein, 2012 |
| Corruption/leakage | Embezzlement – food aid retained by leaders* | Beath et al., 2013 |
|  | Per cent missing roads, unskilled labor, materials* | Olken et al., 2007 |

Note: * synthetic effect calculated across individual outcomes reported.

Table A6.4 Service use and attitudes to services outcome variables

| Outcome | Detailed variable | Study |
| --- | --- | --- |
| Use of health service | Health insurance enrolment | Duku et al., 2018 |
|  | Child received immunization | Donato & Mosqueira, 2016 |
|  | Service use – pre- and post-pregnancy indexes, child immunization* | Giné et al., 2018 |
|  | Antenatal care, admissions to maternity unit, attendance at clinic, pregnant women received free bed nets, mother given vitamin A, child given vitamin A, child dewormed* | Grossman et al., 2017 |
|  | Antenatal, postnatal care received, went for HIV testing with husband/partner* | Gullo et al., 2017 |
|  | Ever heard a health talk at the site | Bradley et al., 2005 |
| Use employment service | Service use: participation, days, wages* | Ravallion et al., 2013 |
| User satisfaction | Perception of overall healthcare quality, health insurance scheme* | Duku et al., 2018 |
|  | Victim reports being 'satisfied' or 'completely satisfied' with police response | Banerjee, 2014 |
|  | Average over family of outcomes on citizen satisfaction with services | Beuermann & Amelina, 2014 |
|  | Desired changes in the delivery of public services | Capuno and Garcia, 2009 |
|  | User satisfaction basic health unit | Giné et al., 2018 |
|  | Satisfaction with governmental services | Kasim, 2016 |
|  | Perceived quality of services when last received: delivery care, family planning* | Gullo et al., 2017 |
|  | Overall satisfaction reported by citizen | Molina, 2014 |
|  | Villagers’ assessment of road quality | Berman et al., 2017 |
|  | Household satisfaction with village natural resource committee, household ranking of trajectory of forest reserve condition* | Persha et al., 2016 |
|  | Attitudes - public infrastructure improved in last year, migration decreased in village, benefits to participation in planning (Gram Sabha), work increased in village, wage increased in village, project increased employment, can get work when demanded, work opportunities increased, women treated well at worksite, women of hh would like to work on project, assets created by project are useful to women* | Ravallion et al., 2013 |
|  | Clients agree with positive statements about services and facility in general | Bradley et al., 2005 |
|  | Overall performance of the community project management committee | Fiala et al., 2017 |
|  | Complaints reported - protests reported, complaints about distribution process, complaints about list of beneficiaries* | Banerjee et al., 2018 |
| Perceived quality of staff | Public perception of police responsiveness to citizens, victim reports police became aware of victims' crimes, confidence in police* | Banerjee et al., 2014 |
|  | Able to convey concerns to service provider, treated well by the service provider, user satisfaction lady health worker* | Giné et al., 2018 |
|  | Perceived quality of family planning services | Gullo et al., 2017 |
|  | Citizen reported provider performance, politician performance* | Molina, 2014 |
|  | Positive views about staff performance* | Bradley et al., 2005 |
|  | Trust in leadership, management committee* | Fiala et al., 2017 |
| Perceived right to access services | Attitudes - women of hh would be allowed to work on NREGA, distance women would be willing to go to work, women paid equal wages as men* | Ravallion et al., 2013 |

Note: * synthetic effect calculated across individual outcomes reported.

Table A6.6 Wellbeing outcome variables

| Outcome | Detailed variable | Study |
| --- | --- | --- |
| Health: mortality | Under five-year-old deaths | Björkman et al., 2017  Donato & Mosqueira, 2016 |
|  | Neonatal mortality | Giné et al., 2018 |
|  | Infant Mortality | Touchton & Wampler, 2015 |
| Health: morbidity | Frequency of Illness | Duku et al., 2018 |
|  | Child diarrhea incidence in the last six months, respondents who fell ill in past month* | Giné et al., 2018 |
| Health: fertility | Average number of pregnancies per year, births per year* | Björkman et al., 2017 |
|  | Number of pregnancies, births | Donato & Mosqueira, 2016 |
| Nutrition | HAZ for children 0-12 months, WAZ for children 0-12 months* | Björkman et al., 2017 |
|  | Incidence of stunting | Giné et al., 2018 |
| Agriculture | Rice production - yield per hectare | Bandyopadhyay et al., 2010 |
|  | Rice production, wheat production (yield per unit land and per unit water)* | Huang et al., 2014 |
|  | All assets, number of cattle* | Fiala et al., 2017 |
| Crime | Household reports crime was committed | Banerjee et al., 2014 |
|  | Respondent reports crime committed | Palladium et al., 2015 |
| Empowerment | Leadership, decision-making, equality of rights, right to complain* | Humphreys et al., 2014 |
|  | Perceived individual influence in community, perceived collective power of community* | Fiala et al., 2017 |
| Social capital | NGO active in village | Ananthpur et al., 2014 |
|  | Member in local organization | Capuno and Garcia, 2010 |
|  | Number of CSOs per municipality | Touchton & Wampler, 2015 |
|  | General level of trust – fear of strangers, feel people are helpful, trust others* | Kasim et al., 2016 |
| Satisfaction with life | Subjective wellbeing | Kasim et al., 2016 |

Note: * synthetic effect calculated across individual outcomes reported.

Table A6.7 State society relations outcome variables

| Outcome | Detailed variable | Study |
| --- | --- | --- |
| Confidence in institutions | Trust in the federal government, civil service, district court, mosques* | Kasim et al., 2016 |
|  | Trust in leaders, local officials and politicians | Fiala et al., 2017 |
| Perceptions about corruption | Public perception of reduction in police corruption | Banerjee et al., 2014 |
|  | Low perceived level of corruption, payment was made to district officer* | Fiala et al., 2017 |
| Taxes paid | Paid tax last year, contributed last year* | Ananthpur et al., 2014 |
|  | Irrigation Service Fees (ISF) collection | Bandyopadhyay et al., 2010 |
|  | Local revenue per capita | Beuermann & Amelina, 2014 |
|  | Rate of water fee collected | Huang et al., 2014 |
|  | Property tax per capita | Timmons et al., 2015 |

Note: * synthetic effect calculated across individual outcomes reported.

Figure A6.1: Forest plots for service access by study design and risk of bias

Figure A6.2: Forest plots for service use and attitudes by study design and bias

Figure A6.3: Forest plots for wellbeing and state-society relations by study design and bias

Figure A6.4: Forest plots for user engagement by design and bias

Figure A6.5: Forest plots for provider response by design and bias

Figure A6.6: Intermediate outcomes by inclusion dimension of intervention

Figure A6.7: Final outcomes by inclusion dimension of intervention

Figure A6.6: Outcomes for female subgroups

Figure A6.7: Outcomes for male subgroups

Figure A6.6: Outcomes for poor subgroups

Figure A6.7: Outcomes by region: user engagement

Figure A6.8: Outcomes by region: provider response

Figure A6.9: Outcomes by region: service access

Figure A6.10: Outcomes by region: service use and attitudes


Figure A6.11: Outcomes by region: wellbeing

Figure A6.12: Outcomes by region: state-society relations

A6.13 Forest plots showing odds ratios

Appendix 7: Critical appraisal of cost data

| Study ID | Type of economic evaluation |  |  |
| --- | --- | --- | --- |
| Alhassan et. al. 2015 | Partial economic evaluation |  |  |
|  |  |  |  |
|  | Item | Yes | No |
| 1 | Is the study population clearly defined? | Yes |  |
| 2 | Are competing alternatives clearly described? | Yes |  |
| 3 | Is a well-defined research question posed in answerable form? | Yes |  |
| 4 | Is the economic study design appropriate to the stated objective? |  | No |
| 5 | Is the chosen time horizon appropriate to include relevant costs and consequences? |  |  |
| 6 | Is the actual perspective chosen appropriate? |  | No |
| 7 | Are all important and relevant costs for each alternative identified? |  | No |
| 8 | Are all costs measured appropriately in physical units? |  | No |
| 9 | Are costs valued appropriately? |  | No |
| 10 | Are all important and relevant outcomes for each alternative identified? |  |  |
| 11 | Are all outcomes measured appropriately? |  |  |
| 12 | Are outcomes valued appropriately? |  |  |
| 13 | Is an incremental analysis of costs and outcomes of alternatives performed? |  |  |
| 14 | Are all future costs and outcomes discounted appropriately? |  |  |
| 15 | Are all important variables, whose values are uncertain, appropriately subjected to sensitivity analysis? |  |  |
| 16 | Do the conclusions follow from the data reported? |  | No |
| 17 | Does the study discuss the generalizability of the results to other settings and patient/ client groups? |  | No |
| 18 | Does the article indicate that there is no potential conflict of interest of study researcher(s) and funder(s)? | Yes |  |
| 19 | Are ethical and distributional issues discussed appropriately? | Yes |  |
| Study ID | Type of economic evaluation |  |  |
| Ananthpur et al. 2014 | Partial economic evaluation |  |  |
|  |  |  |  |
|  | Item | Yes | No |
| 1 | Is the study population clearly defined? | Yes |  |
| 2 | Are competing alternatives clearly described? | Yes |  |
| 3 | Is a well-defined research question posed in answerable form? | Yes |  |
| 4 | Is the economic study design appropriate to the stated objective? |  | No |
| 5 | Is the chosen time horizon appropriate to include relevant costs and consequences? |  |  |
| 6 | Is the actual perspective chosen appropriate? |  | No |
| 7 | Are all important and relevant costs for each alternative identified? |  | No |
| 8 | Are all costs measured appropriately in physical units? |  | No |
| 9 | Are costs valued appropriately? |  | No |
| 10 | Are all important and relevant outcomes for each alternative identified? |  |  |
| 11 | Are all outcomes measured appropriately? |  |  |
| 12 | Are outcomes valued appropriately? |  |  |
| 13 | Is an incremental analysis of costs and outcomes of alternatives performed? |  |  |
| 14 | Are all future costs and outcomes discounted appropriately? |  |  |
| 15 | Are all important variables, whose values are uncertain, appropriately subjected to sensitivity analysis? |  |  |
| 16 | Do the conclusions follow from the data reported? |  | No |
| 17 | Does the study discuss the generalizability of the results to other settings and patient/ client groups? |  | No |
| 18 | Does the article indicate that there is no potential conflict of interest of study researcher(s) and funder(s)? |  | No |
| 19 | Are ethical and distributional issues discussed appropriately? |  | No |
| Study ID | Type of economic evaluation |  |  |
| Björkman et al. 2017 | Full economic evaluation (Cost-effectiveness analysis) |  |  |
|  |  |  |  |
|  | Item | Yes | No |
| 1 | Is the study population clearly defined? | Yes |  |
| 2 | Are competing alternatives clearly described? | Yes |  |
| 3 | Is a well-defined research question posed in answerable form? | Yes |  |
| 4 | Is the economic study design appropriate to the stated objective? | Yes |  |
| 5 | Is the chosen time horizon appropriate to include relevant costs and consequences? |  | No |
| 6 | Is the actual perspective chosen appropriate? |  | No |
| 7 | Are all important and relevant costs for each alternative identified? |  | No |
| 8 | Are all costs measured appropriately in physical units? |  | No |
| 9 | Are costs valued appropriately? |  | No |
| 10 | Are all important and relevant outcomes for each alternative identified? |  | No |
| 11 | Are all outcomes measured appropriately? |  | No |
| 12 | Are outcomes valued appropriately? |  | No |
| 13 | Is an incremental analysis of costs and outcomes of alternatives performed? |  | No |
| 14 | Are all future costs and outcomes discounted appropriately? |  | No |
| 15 | Are all important variables, whose values are uncertain, appropriately subjected to sensitivity analysis? |  | No |
| 16 | Do the conclusions follow from the data reported? |  | No |
| 17 | Does the study discuss the generalizability of the results to other settings and patient/ client groups? |  | No |
| 18 | Does the article indicate that there is no potential conflict of interest of study researcher(s) and funder(s)? | Yes |  |
| 19 | Are ethical and distributional issues discussed appropriately? | Yes |  |
| Study ID | Type of economic evaluation |  |  |
| Pandey et al. 2007 | Partial economic evaluation |  |  |
|  |  |  |  |
|  | Item | Yes | No |
| 1 | Is the study population clearly defined? | Yes |  |
| 2 | Are competing alternatives clearly described? | Yes |  |
| 3 | Is a well-defined research question posed in answerable form? | Yes |  |
| 4 | Is the economic study design appropriate to the stated objective? |  | No |
| 5 | Is the chosen time horizon appropriate to include relevant costs and consequences? |  |  |
| 6 | Is the actual perspective chosen appropriate? |  | No |
| 7 | Are all important and relevant costs for each alternative identified? |  | No |
| 8 | Are all costs measured appropriately in physical units? |  | No |
| 9 | Are costs valued appropriately? |  | No |
| 10 | Are all important and relevant outcomes for each alternative identified? |  |  |
| 11 | Are all outcomes measured appropriately? |  |  |
| 12 | Are outcomes valued appropriately? |  |  |
| 13 | Is an incremental analysis of costs and outcomes of alternatives performed? |  |  |
| 14 | Are all future costs and outcomes discounted appropriately? |  |  |
| 15 | Are all important variables, whose values are uncertain, appropriately subjected to sensitivity analysis? |  |  |
| 16 | Do the conclusions follow from the data reported? |  | No |
| 17 | Does the study discuss the generalizability of the results to other settings and patient/ client groups? |  | No |
| 18 | Does the article indicate that there is no potential conflict of interest of study researcher(s) and funder(s)? | Yes |  |
| 19 | Are ethical and distributional issues discussed appropriately? | Yes |  |
